# Synthesis and Functionalization of Tertiary Propargylic Boronic Esters by Alkynyllithium-Mediated 1,2-Metalate Rearrangement of Borylated Cyclopropanes

**DOI:** 10.1021/acs.orglett.2c03756

**Published:** 2022-11-29

**Authors:** Tereza Pavlíčková, Yannick Stöckl, Ilan Marek

**Affiliations:** Schulich Faculty of Chemistry, Technion − Israel Institute of Technology, Technion City, Haifa 3200009, Israel

## Abstract

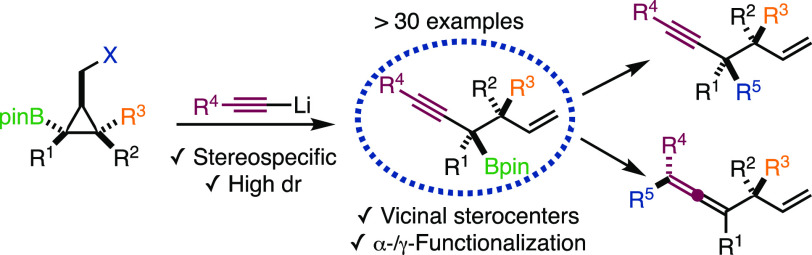

Implementing the use of alkynyllithium reagents in a stereospecific 1,2-metalate rearrangement-mediated ring opening of polysubstituted cyclopropyl boronic esters provides a variety of tertiary pinacol boranes bearing adjacent tertiary or quaternary carbon stereocenters with high levels of diastereomeric purity. The potential of this strategy was demonstrated through a selection of α- and γ-functionalization of the propargyl boronic esters.

Control over multiple vicinal carbon stereocenters in acyclic systems remains a challenging problem in stereoselective organic synthesis, notably when these centers are tertiary or quaternary.^1^ The main obstacles are conformational flexibility of acyclic frameworks along with distorted bond geometries around fully substituted carbon atoms.^2^ The majority of methods, which have appeared over the past decade to answer this synthetic riddle rely on reaction of two acyclic fragments, at least one of them stereodefined, where a double face differentiation is required to control the diastereoselectivity (Scheme 1A).^3^ On the other hand, triggering selective C–C bond cleavage of easily accessible stereodefined cyclopropanes by using simple reagents should provide an elegant alternative solution.^4^ Along these lines, our group has reported several examples of ring opening of polysubstituted cyclopropanes via a stereoinvertive nucleophilic substitution at quaternary carbon stereocenters (Scheme 1B),^5^ suggesting a nucleophilic attack at a cyclopropylcarbinyl cation intermediate as a common mechanistic denominator.^6^ Combination of heteroatom nucleophiles with Lewis acid-activated cyclopropyl carbinols, methyl ethers, and ketones gave rise to a range of tertiary alkyl halides, esters, and azides,^5^ whereas employing organoaluminum reagents allowed the stereospecific construction of quaternary carbon stereocenters.^7^ Even though all these structures were obtained with high degrees of diastereoselectivity, access to the latter was limited to the use of the restricted number of commercially available organoaluminum species as C-nucleophiles. In our quest for a more general approach, we have recently developed a highly regio- and diastereoselective synthesis of polysubstituted cyclopropyl boronic esters,^8^ suitable candidates for 1,2-metalate rearrangement-mediated ring opening using various alkyl- and aryllithium reagents (Scheme 1C).^8,9^

**Scheme 1:**
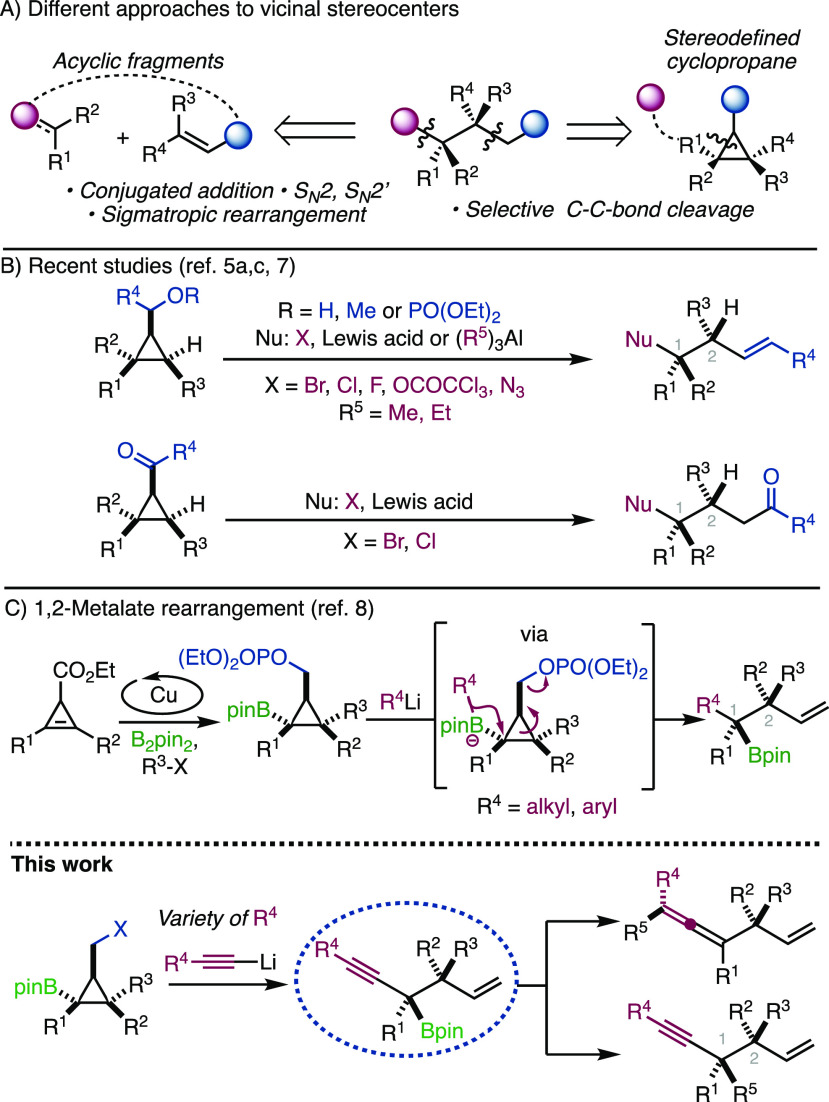
Stereoselective Synthesis of Vicinal Stereocenters

Corresponding acyclic tertiary boronic esters with a neighboring tertiary or quaternary carbon stereocenter were obtained in a highly stereospecific manner. Nevertheless, subsequent functionalization of the boronic ester and therefore access to more complex molecular architectures have remained elusive to this approach.

In contrast, expanding the scope of nucleophiles to C(sp)-hybridized organolithium reagents would give stereodefined tertiary propargylic boronic esters (Scheme 1C, bottom), which are underrepresented in the literature^10^ but have great potential for subsequent α- or γ-selective transformations.^11^ Moreover, lithium acetylides are readily accessible from a large variety of commercially and synthetically available terminal alkynes by simple deprotonation, allowing an easy diversification of the product scope.

Inspired by our recent work,^8^ we set out to synthesize a library of borylated cyclopropyl esters **1a**–**h** using the previously developed Cu-catalyzed hydro- and carboborylation (Scheme 2).^12^ The starting mono- and disubstituted cyclopropenyl esters were easily obtained by a well-described Rh-catalyzed decomposition of ethyl diazoacetate in the presence of the corresponding alkynes.^13^ Following this approach, diversity of substituents could be achieved by employing different cyclopropenes (R^1^ and R^2^) as well as by changing the nature of the electrophile (R^3^ = H or allyl). Based on our previous studies, ethyl esters **1a**–**d** were transformed to phosphates **2a**–**d** by a two-step reduction/phosphorylation sequence.^7,8,14^ In addition, we decided to explore the potentially more suitable, yet less stable, cyclopropyl methyl iodides **4**a–**g**, which were synthesized *ad hoc*, prior to the ring opening, from the corresponding bench-stable carbinols **3a**–**g** (see Supporting Information for details).^15^

**Scheme 2:**
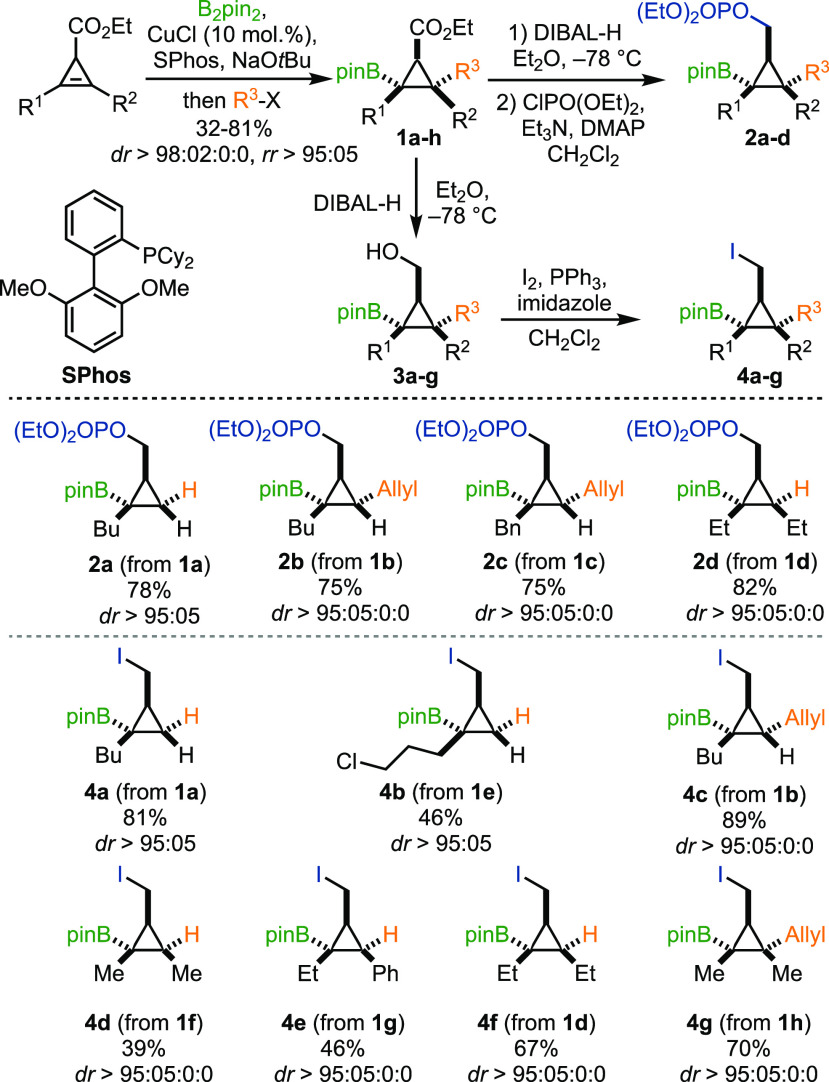
Synthesis of Starting Materials

It is noteworthy that potential minor regio- and/or diastereomers resulting from the Cu-catalyzed carboboration addition are fully separable by simple purification by column chromatography at the stage of phosphates or carbinols and therefore compounds **2a**–**d** and **4a**–**g** were thus obtained as single isomers with the pinacol borane conveniently oriented *trans* to the leaving group.

Subsequently, we focused on finding the optimal conditions for the alkynyllithium-mediated 1,2-metalate rearrangement. In this context, it should be noted that the C(sp)-hybridized organolithium derivatives are less nucleophilic than their alkyl or aryl counterparts and while lithium boronate complexes are favored at low temperatures (−78 °C), free alkynyllithium and boronic ester are observed at room temperature (Scheme 3).^10a,16^ The electronic properties of R^4^ of the parent alkynyllithium should similarly be reflected in the reactivity of the corresponding cyclopropyl boronate complex and might slow down the 1,2-rearrangement, giving space to competitive reaction pathways.^17^ As such, the reversible 1,3-shift of α-substituted propargylic boronic compounds (**B** to **C**) should be considered as well as the potential dissociation of **A** into its two parent reactants (Scheme 3). In the latter case, the free alkynyllithium can react with **B** to form a propargylic ate-complex **D** that could dissociate into propargyl lithium species **E** that should be prone to epimerization.^18^ The position of these equilibria should be fine-tunable by the steric and electronic nature of both reactants.^17b^ Thus, our set of borylated cyclopropanes with varying substitution pattern (R^1^, R^2^, R^3^, X) as well as the possibility to permute R^4^ in the acetylide provided a crucial starting point to explore the envisaged transformation.

**Scheme 3:**
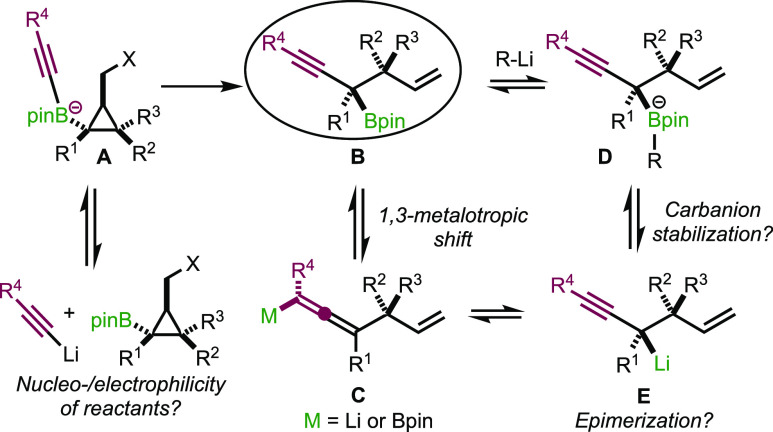
Potential Equilibrium-Associated Challenges in the Desired Transformation

We started our investigations on simple model substrates **2a** and **4a** by adding phenylacetylene as an acetylide source at low temperature and slowly raised the temperature of the reaction mixture to room temperature (Scheme 4). Indeed, when a two-fold excess of acetylide was added to **2a** at −78 °C and the mixture was slowly warmed to room temperature over 45 min, allene **5a** was formed as a single product in 58% yield, pointing to a possible boron/Li exchange with subsequent metalotropic shift (see Scheme 3). With the hope of slowing down the metalotropic equilibrium as well as potential transmetalation reaction (**D** to **E**), the same reaction was performed at a lower temperature (−95 °C) with 1.05 equiv of acetylide. Under this condition, formation of **5a** was mostly suppressed. However, in addition to the expected propargylic boronic ester **6a**, elimination product **7** was also obtained in a 1.7:1 ratio. Gratifyingly, when the same transformation was carried out with **4a**, we were pleased to mainly observe the formation of **6a** (65%, **6a**:**7** = 93:07). The same trend was observed when hexynyllithium was used as the nucleophile. With **2a**, **6b** was obtained in 72% with a ratio of **6b**:**7** = 80:20 whereas **4a** gave **6b** in 85% yield with an improved ratio of **6b**:**7** = 92:08 (see the Supporting Information for more details).

**Scheme 4:**
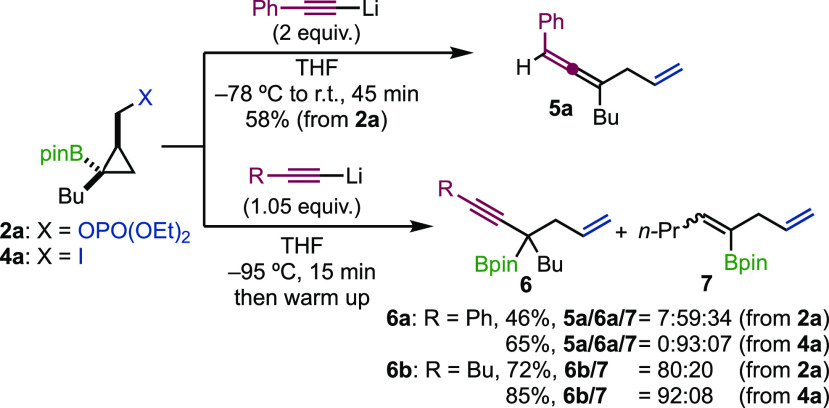
1,2-Metalate Rearrangement versus Elimination Reaction

With proper experimental conditions in hand, we initially focused on extending this transformation to various alkynes to determine the scope using **4a**–**g** as starting materials. First, disubstituted cyclopropyl iodides **4a**,**b** (R^1^ = alkyl, R^2^ = R^3^ = H) were treated with a variety of acetylides to give propargylic boronic esters **6a**–**m** containing a single tertiary stereocenter (Scheme 5A). The reaction proceeded smoothly for alkynes possessing linear, cyclic, or branched alkyl residues to give **6b**–**e** in good yields. Electron-rich aromatic substituents performed well, affording products **6a** and **6f**,**g** in 52–58% yield. Conversely, the *p*-CF_3_ group hampered the reaction to proceed satisfactorily, and the yield sharply dropped (not shown in Scheme 5). Silylated alkynyllithiums were well tolerated to give compounds **6h**,**i** with a protected terminal alkyne handle. A silyl ether moiety at a different position from the alkyne moiety was compatible under our reaction conditions (**6j**,**k**) as well as a chlorinated residue at both, R^1^ or R^4^ positions, giving functionalized products **6l** and **6m** in good to excellent yields.

**Scheme 5:**
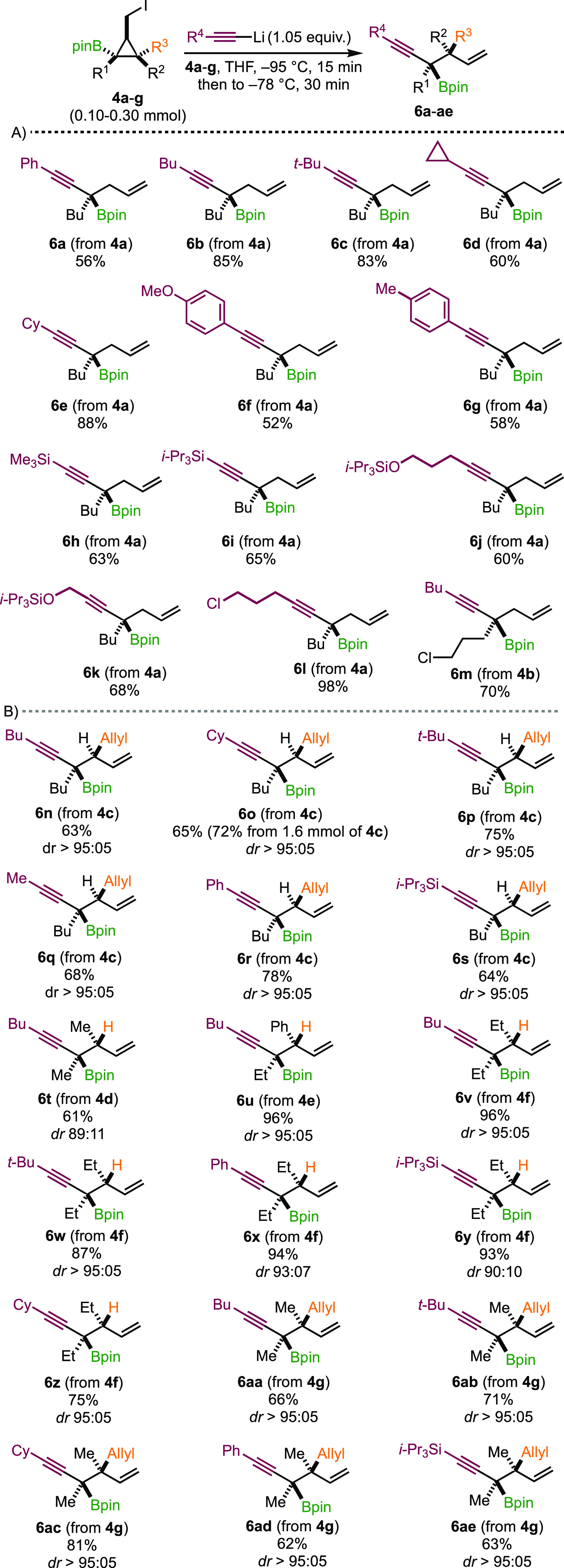
Products of Molecular Rearrangement

Having a rather large scope of potential substituents on the alkyne, we further explored the stereochemical outcome of the 1,2-metalate rearrangement (Scheme 5B). To our delight, a complete stereospecific molecular rearrangement of cyclopropane **4c** provided the corresponding acyclic products **6n**–**s** having an alkyl, phenyl, or silyl ethynyl group and two vicinal encumbered stereocenters in good yields with outstanding diastereoselectivities. Furthermore, reaction with cyclohexyl ethynyllithium was scaled to over 600 mg (1.6 mmol) of iodide **4c** without any significant impact on the yield (72%) or diastereoselectivity of the resulting product **6o**. Similarly, ring opening of substrates **4d**–**f** with two alkyl chains in a *cis*-relationship was successfully achieved in high yields and diastereospecificity with only a minor erosion of diastereomeric purity for compound **6t** (*dr* 89:11). Encouraged by these results, we finally treated pentasubstituted cyclopropane **4g** with an assortment of lithium acetylides, and we were pleased to find that a smooth transformation occurred in all cases. Compounds **6aa**–**ae** possessing a tertiary propargyl boronic ester next to a quaternary carbon stereocenter were thus produced with good yields and as single diastereomers (Scheme 5B).

Having access to a wide range of stereodefined tertiary boronic esters, we were keen to exploit the reactivity of the propargylic pinacol borane moiety in various subsequent transformations. First, we envisaged that oxidation of **6** with basic H_2_O_2_ would afford tertiary alcohols **8a**–**k** in a stereospecific manner. Indeed, using standard oxidation,^19^ corresponding alcohols **8** were obtained in generally good yields with a complete diastereospecificity (Scheme 6A).

**Scheme 6:**
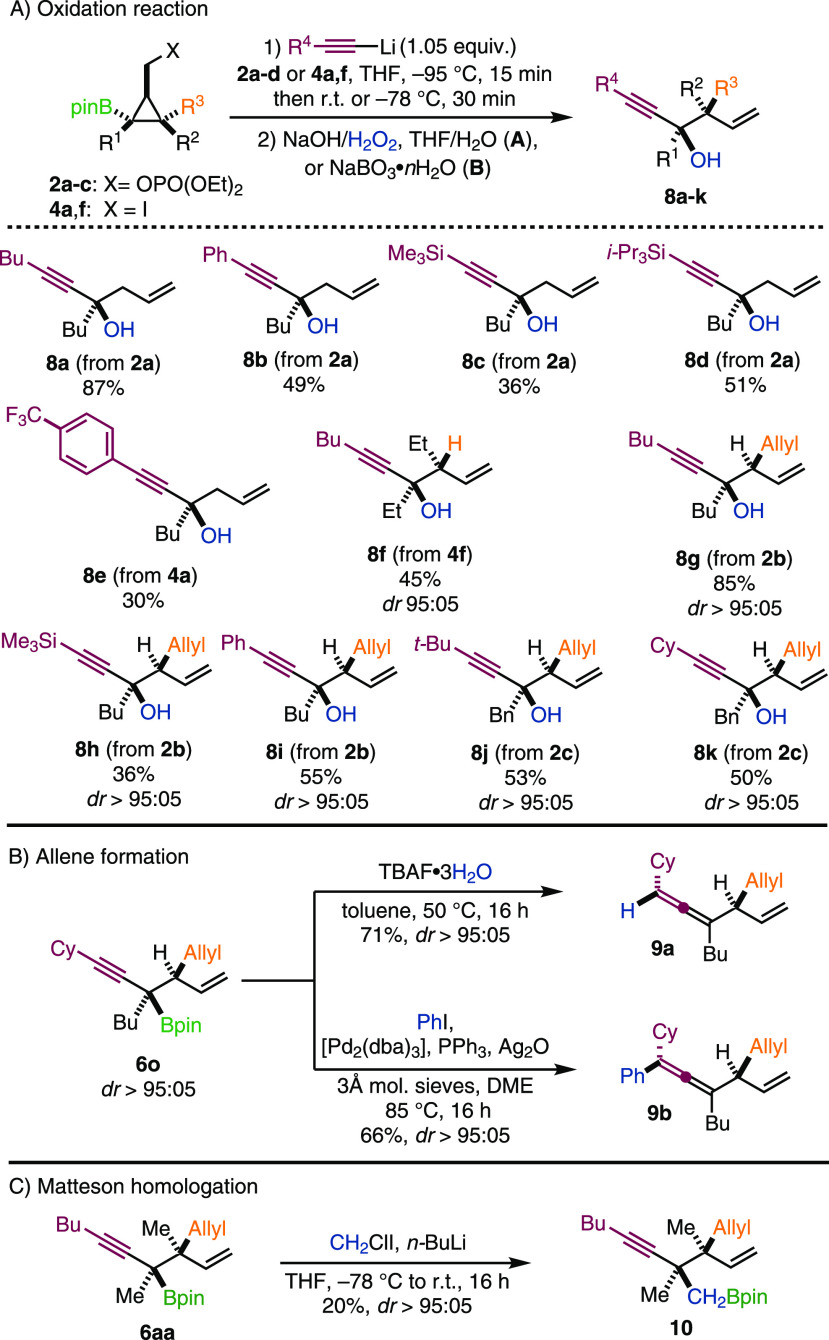
Postfunctionalization

The trimethylsilyl-protected alkynes were incompatible with these conditions, and we employed a modified oxidation reaction to provide **8c** and **8h**, albeit in moderate yields, by using NaBO_3_·*n*H_2_O in water.^1i,20^ It should be noted that the easy-to-handle cyclopropyl methyl phosphates **2a**–**c** proved to be generally more suitable substrates for a direct *in situ* oxidation of the products resulting from the 1,2-boronate rearrangement than the corresponding iodides due to the liberation of molecular iodine resulting from oxidation of LiI formed in the latter case.

Subsequently, we aimed to expand the array of postsynthetic modifications *en route* to fully substituted acyclic hydrocarbon frameworks. Inspired by the work of Aggarwal,^10a^ we subjected compound **6o** to TBAF-promoted protodeborylation and γ-selective Pd-catalyzed cross-coupling to provide tri- and tetrasubstituted allene **9a** and **9b** with 71% and 66% yields, respectively, without loss of stereochemical information (Scheme 6B).^10a,11a,11b^ Treating propargylic boronic esters with an excess of organolithium according to Scheme 4 also provided the corresponding allenes, but in a nonstereospecific manner (not shown in Scheme 6). Finally, Matteson-type homologation of **6aa** furnished boronic ester **10** bearing two adjacent quaternary carbon stereocenters, albeit in 20% yield (Scheme 6C).^21^ It should be emphasized that this transformation is sensitive to steric hindrance, and densely substituted tertiary pinacol boranes bearing branched substituents are usually out of scope.^1c^

In conclusion, we have developed a robust and modular approach to acyclic tertiary propargylic boronic esters from readily available cyclopropenes that are easily transformed into cyclopropyl boronic ester and subsequently treated with a large variety of terminal alkynes in a stereospecific manner. Acyclic fragments bearing vicinal tertiary or quaternary stereocenters have been synthesized with high diastereomeric ratios. We then highlighted the propargyl boronic ester moiety as a useful synthetic platform for a plethora of known postsynthetic modifications furnishing propargyl alcohols, tri- and tetrasubstituted allenes, and even acyclic frameworks with two adjacent quaternary carbon stereocenters. The development of new transformations to fully exploit the potential of these synthetic building blocks toward other types of acyclic architectures can be foreseen, and the investigations are underway.

## Data Availability

The data underlying this study are available in the published article and its online Supporting Information.

## Supporting Information Available

Experimental details, characterization data, and spectra (PDF)

## References

[ref1] aPeterson E. A.; Overman L. E. Contiguous stereogenic quaternary carbons: A daunting challenge in natural products synthesis. Proc. Natl. Acad. Sci. U. S. A. 2004, 101, 11943–11948. 10.1073/pnas.0402416101. 15232003 PMC514413

[ref2] Gonthier J. F.; Wodrich M. D.; Steinmann S. N.; Corminboeuf C. Branched Alkanes Have Contrasting Stabilities. Org. Lett. 2010, 12, 3070–3073. 10.1021/ol1010642. 20521826

[ref3] aDenmark S. E.; Fu J. Catalytic Enantioselective Addition of Allylic Organometallic Reagents to Aldehydes and Ketones. Chem. Rev. 2003, 103, 2763–2794. 10.1021/cr020050h. 12914480

[ref4] aMarek I.; Masarwa A.; Delaye P.-O.; Leibeling M. Selective Carbon–Carbon Bond Cleavage for the Stereoselective Synthesis of Acyclic Systems. Angew. Chem., Int. Ed. 2015, 54, 414–429. 10.1002/anie.201405067. 25266824

[ref5] aLanke V.; Marek I. Stereospecific nucleophilic substitution at tertiary and quaternary stereocentres. Chem. Sci. 2020, 11, 9378–9385. 10.1039/D0SC02562C. 34094203 PMC8161537

[ref6] Larmore S.; Champagne P. A. Non-classical-to-classical carbocation equilibria influence the stereospecificity in the nucleophilic substitution of cyclopropylcarbinols. ChemRxiv Preprint 2022, 10.26434/chemrxiv-2022-h9bq3. 37141426

[ref7] Patel K.; Lanke V.; Marek I. Stereospecific Construction of Quaternary Carbon Stereocenters from Quaternary Carbon Stereocenters. J. Am. Chem. Soc. 2022, 144, 7066–7071. 10.1021/jacs.2c01695. 35412821 PMC9052742

[ref8] Augustin A. U.; Di Silvio S.; Marek I. Borylated Cyclopropanes as Spring-Loaded Entities: Access to Vicinal Tertiary and Quaternary Carbon Stereocenters in Acyclic Systems. J. Am. Chem. Soc. 2022, 144, 16298–16302. 10.1021/jacs.2c07394. 36041738 PMC9479080

[ref9] aGregson C. H. U.; Ganesh V.; Aggarwal V. K. Strain Release of Donor–Acceptor Cyclopropyl Boronate Complexes. Org. Lett. 2019, 21, 3412–3416. 10.1021/acs.orglett.9b01152. 31017445

[ref10] aPartridge B. M.; Chausset-Boissarie L.; Burns M.; Pulis A. P.; Aggarwal V. K. Enantioselective Synthesis and Cross-Coupling of Tertiary Propargylic Boronic Esters Using Lithiation-Borylation of Propargylic Carbamates. Angew. Chem., Int. Ed. 2012, 51, 11795–11799. 10.1002/anie.201203198. 23076714

[ref11] aFandrick D. R.; Reeves J. T.; Tan Z.; Lee H.; Song J. J.; Yee N. K.; Senanayake C. H. Regioselective Allene Synthesis and Propargylations with Propargyl Diethanolamine Boronates. Org. Lett. 2009, 11, 5458–5461. 10.1021/ol9022529. 19877630

[ref12] aRubina M.; Rubin M.; Gevorgyan V. Catalytic Enantioselective Hydroboration of Cyclopropenes. J. Am. Chem. Soc. 2003, 125, 7198–7199. 10.1021/ja034210y. 12797792

[ref13] Petiniot N.; Anciaux A. J.; Noels A. F.; Hubert A. J.; Teyssié P. Rhodium catalysed cyclopropenation of acetylenes. Tetrahedron Lett. 1978, 19, 1239–1242. 10.1016/S0040-4039(01)94511-3.

[ref14] Cormier M.; de la Torre A.; Marek I. Total Synthesis of C30 Botryococcene and epi-Botryococcene by a Diastereoselective Ring Opening of Alkenylcyclopropanes. Angew. Chem., Int. Ed. 2018, 57, 13237–13241. 10.1002/anie.201808709. 30134044

[ref15] aLansbury P. T.; Pattison V. A.; Clement W. A.; Sidler J. D. Preparation and Properties of Cyclopropylcarbinyllithium. J. Am. Chem. Soc. 1964, 86, 2247–2251. 10.1021/ja01065a029.

[ref16] aBrown H. C.; Srebnik M. Organoboranes. 50. Preparation and characterization of organyl-1-alkynylborinic esters. Organometallics 1987, 6, 629–631. 10.1021/om00146a032.

[ref17] aFeeney K.; Berionni G.; Mayr H.; Aggarwal V. K. Structure and Reactivity of Boron-Ate Complexes Derived from Primary and Secondary Boronic Esters. Org. Lett. 2015, 17, 2614–2617. 10.1021/acs.orglett.5b00918. 25973673

[ref18] Bejjani J.; Botuha C.; Chemla F.; Ferreira F.; Magnus S.; Pérez-Luna A. Metallotropic Equilibrium and Configurational Stability of 3-Chloro-1-(trimethylsilyl)propargyl and -allenyl Metals: Comparative Study among Lithium, Titanium, and Zinc. Organometallics 2012, 31, 4876–4885. 10.1021/om300420q.

[ref19] aBrown H. C.; Zweifel G. Hydroboration. IX. The Hydroboration of Cyclic and Bicyclic Olefins—Stereochemistry of the Hydroboration Reaction. J. Am. Chem. Soc. 1961, 83, 2544–2551. 10.1021/ja01472a028.

[ref20] Kabalka G. W.; Shoup T. M.; Goudgaon N. M. Sodium perborate: A mild and convenient reagent for efficiently oxidizing trialkylboranes. Tetrahedron Lett. 1989, 30, 1483–1486. 10.1016/S0040-4039(00)99497-8.

[ref21] Sadhu K. M.; Matteson D. S. (Chloromethyl)lithium: efficient generation and capture by boronic esters and a simple preparation of diisopropyl (chloromethyl)boronate. Organometallics 1985, 4, 1687–1689. 10.1021/om00128a038.

