# Supplementary material for: Synthesis and Functionalization of Tertiary Propargylic Boronic Esters by Alkynyllithium-Mediated 1,2-Metalate Rearrangement of Borylated Cyclopropanes
Source: Org Lett. 2022 Nov 29;24(48):8901–6. doi: 10.1021/acs.orglett.2c03756 (PMC9791689; doi:10.1021/acs.orglett.2c03756)
Supplement: Supplementary file 1 — ol2c03756_si_001.pdf [file ol2c03756_si_001.pdf]

Supporting information

Experimental details and characterization data (PDF)

Synthesis and Functionalization of Tertiary Propargylic Boronic Esters by Alkynyllithium-Mediated 1,2-Metalate Rearrangement of Borylated Cyclopropanes

Tereza Pavlíčková, Yannick Stöckl and Ilan Marek*

ABSTRACT: Implementing the use of alkynyllithium reagents in a stereospecific 1,2-metalate rearrangement-mediated ring opening of polysubstituted cyclopropyl boronic esters provides a variety of tertiary pinacol boranes bearing adjacent tertiary or quaternary carbon stereocenters with high levels of diastereomeric purity. The potential of this strategy was demonstrated through a selection of α- and γ-functionalization of the propargyl boronic esters.

Table of Contents

1. General information....................3
2. Synthesis of starting materials....................4
   2.1 Cyclopropyl esters 1a-h....................4
   2.2 Phopshates 2a-d....................5
   2.3 Carbinols 3a-g....................7
   2.4 Iodides 4a-g....................11
3. New experimental procedures and analytical data....................16
   3.1 Optimization of the 1,2-metalate rearrangement....................16
   3.2 Propargylic boronic esters 6a-ae....................18
   3.3 Propargylic alcohols 8a-k....................37
   3.4 Other post-functionalization reactions....................44
4. NMR spectra of new compounds....................47
5. References....................166

1. General information

Air- and moisture-sensitive reactions were conducted in flame-dried glassware under a positive pressure of argon. Solvents were dried via distillation according to standard procedures (CH_2Cl_2, Et_3N) or used from a solvent purification system (THF, Et_2O; Pure-Solv.® Purification System) and stored at least 72 hours over activated 4Å molecular sieves before usage. Commercially available reagents were used as purchased unless otherwise stated. Commercially available organolithium reagents were titrated twice against *N*-benzylbenzamide before usage. Thin-layer chromatography (TLC) was conducted with E. Merck silica gel 60 F254 pre-coated plates (0.25 mm) and visualized by exposure to UV light (254 nm) or stained with acidic *p*-anisaldehyde, cerium molybdate, or potassium permanganate solutions. Column chromatography was performed using Fluka silica gel 60 Å (40-63 mm, 230-400 mesh). Petroleum ether (PE, 60-80 °C boiling range) was used for chromatographic separations. NMR spectra were recorded in $CDCl_3$ on a *Bruker Avance* AVIII400 instrument. ^{1}H NMR chemical shifts are provided using TMS as external standard (internal reference at δ = 7.26 ppm) and are reported as follows: chemical shift in ppm [multiplicity, coupling constant(s) *J* in Hz, integral]. The following abbreviations were used for peak multiplicities: br = broad, m = multiplet, s = singlet, d = doublet, t = triplet, q = quadruplet, quint = quintuplet, sext = sextuplet, sept = septuplet or combinations thereof. Carbon (^{13}C, APT) chemical shifts are referenced against the residual central solvent peak (δ = 77.16 ppm for $CDCl_3$) and are given in ppm. Boron (^{11}B), fluorine (^{19}F) and phosphorus (^{31}P) shifts are given in ppm using external calibration. High-resolution mass spectroscopy (HRMS) was carried out on a FTICR Bruker maXis impact™ high resolution QTOF mass spectrometer at the Mass Spectroscopy Unit of the Schulich Faculty of Chemistry at the Technion – Israel Institute of Technology. Diastereomeric ratios (*dr*) were determined either by crude ^{1}H NMR (relaxation delay D1 = 6 s) or by GC/FID analysis using an Agilent Technologies 7820A GC with an Agilent Technologies 19091J-413 (30 m × 0.3 mm) column.

2. Synthesis of starting materials

2.1 Cyclopropyl esters 1a-h

Cyclopropyl boronic esters **1a**, **1b**, **1e**, **1f**, **1g** and **1h** were prepared according to a literature procedure by Cu-mediated hydro- or carboboration of corresponding cyclopropenes;[1] the experimental results were in agreement with the literature report.[2]

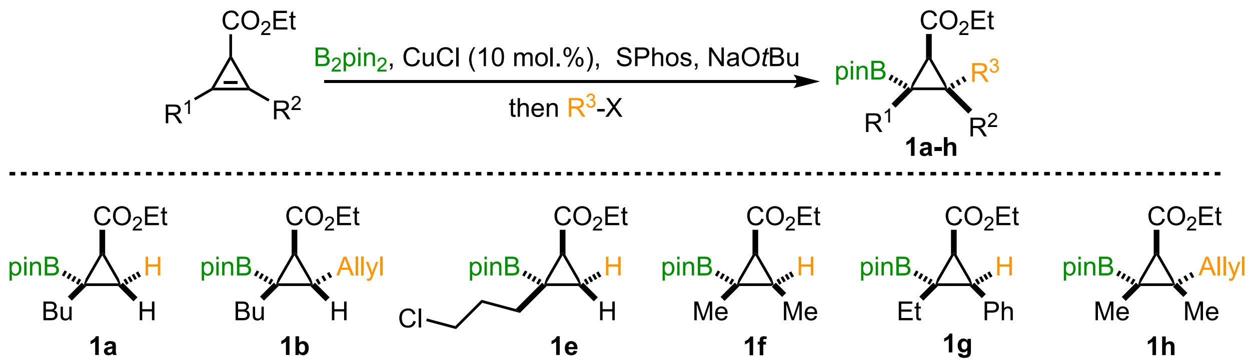

Ethyl (1*S,2*R**,3*S**)-3-allyl-2-benzyl-2-(4,4,5,5-tetramethyl-1,3,2-dioxaborolan-2-yl)cyclopropane-1-carboxylate (1c)**

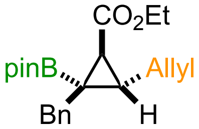

Chemical Formula: $C_{22}H_{31}BO_4$
Molecular Weight: 370.30

Prepared according to a literature procedure[2] from 1-benzyl-3-ethoxycarbonylcyclopropene[1e] (777 mg, 3.84 mmol), purified by flash column chromatography (silica gel, gradient PE/Et_2O 975:25 to 95:5).

Yield: 458 mg (2.19 mmol, 32%, *dr* > 95:05:0:0 as determined by ^{1}H NMR spectroscopy) as a pale yellow oil.

R*f* = 0.80 (PE/EtOAc = 90:10).

^{1}H NMR (400 MHz, $CDCl_3$): δ 7.20 (d, *J* = 4.4 Hz, 4H), 7.14-7.07 (m, 1H), 5.84 (ddt, *J* = 17.3, 10.3, 6.0 Hz, 1H), 5.08 (dq, *J* = 17.3, 1.8 Hz, 1H), 5.00 (dq, *J* = 10.3, 1.7 Hz, 1H), 4.07-3.96 (m, 2H), 3.22 (d, *J* = 15.7 Hz, 1H), 2.90 (d, *J* = 15.7 Hz, 1H), 2.29-2.18 (m, 2H), 2.03 (d, *J* = 5.5 Hz, 1H), 1.71 (td, *J* = 7.3, 5.6 Hz, 1H), 1.16 (t, *J* = 7.1 Hz, 3H), 1.13 (s, 6H), 1.05 (s, 6H) ppm.

^{13}C NMR (101 MHz, $CDCl_3$): δ 172.6, 141.7, 137.2, 128.6, 127.9, 125.6, 115.2, 83.9, 60.5, 35.0, 33.9, 31.9, 29.9, 25.0, 24.6, 14.4 ppm.
Note: Carbon atom attached to boron is not visible due to quadrupolar relaxation.

^{11}B NMR (128 MHz, $CDCl_3$): δ 32.3 ppm.

HRMS (APCI+) m/z: calcd. for $C_{22}H_{32}BO_4^+$ [*M*+H]$^+$: 371.2388, found: 371.2382.

Ethyl (1*S,2*R**,3*R**)-2,3-diethyl-2-(4,4,5,5-tetramethyl-1,3,2-dioxaborolan-2-yl)cyclopropane-1-carboxylate (1d)**

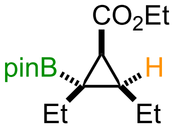

Chemical Formula: $C_{16}H_{29}BO_4$
Molecular Weight: 296.21

Prepared according to a literature procedure[2] from 1,2-diethyl-3-ethoxycarbonylcyclopropene[1d] (841 mg, 5.00 mmol), purified by flash column chromatography (silica gel, gradient PE/Et_2O 975:25 to 95:5).

Yield: 650 mg (2.19 mmol, 44%, *dr* > 95:05:0:0 as determined by ^1H NMR spectroscopy) as a colorless oil.

R*f* = 0.35 (PE/EtOAc = 25:1).

^{1}H NMR (400 MHz, $CDCl_3$): δ 4.17-4.01 (m, 2H), 1.80 (d, *J* = 8.4 Hz, 1H), 1.74-1.62 (m, 4H), 1.34 (td, *J* = 8.4, 6.3 Hz, 1H), 1.25 (t, *J* = 7.1 Hz, 3H), 1.22 (s, 6H), 1.21 (s, 6H), 0.93 (t, *J* = 7.4 Hz, 3H), 0.89 (t, *J* = 7.3 Hz, 3H) ppm.

^{13}C NMR (101 MHz, $CDCl_3$): δ 172.0, 83.5, 59.9, 32.1, 24.8, 24.64, 24.56, 16.5, 16.0, 14.5, 14.2, 13.7 ppm. *Note*: Carbon atom attached to boron is not visible due to quadrupolar relaxation.

^{11}B NMR (128 MHz, $CDCl_3$): δ 33.4 ppm.

HRMS (APCI+) m/z: calcd. for $C_{16}H_{30}BO_4^+$ $[M+H]^+$: 297.2232, found: 297.2245.

2.2 Phopshates **2a-d**

Phosphates **2a** and **2b** were prepared by a reduction/phosphorylation sequence from corresponding ethyl esters as described in the literature; experimental results were in agreement with the literature report.[2]

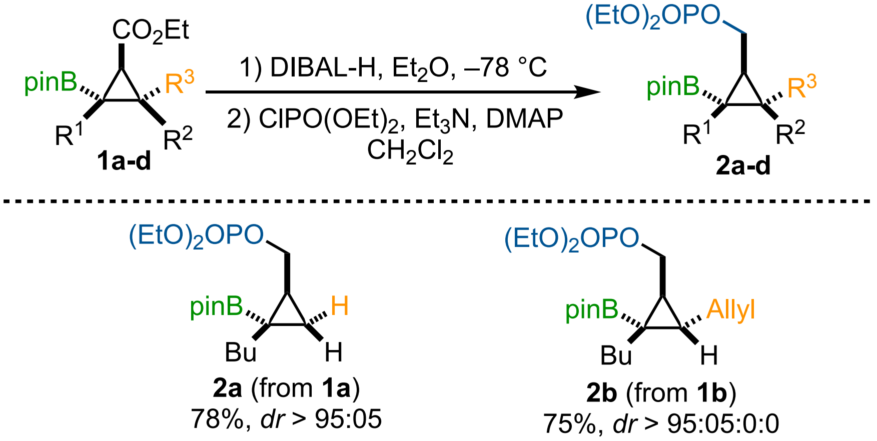

((1*S,2*R**,3*S**)-3-Allyl-2-benzyl-2-(4,4,5,5-tetramethyl-1,3,2-dioxaborolan-2-yl)cyclopropyl)methyl diethyl phosphate (2c)**

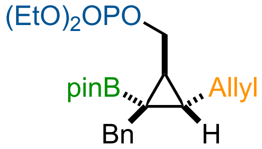

Chemical Formula: $C_{24}H_{38}BO_6P$
Molecular Weight: 464.35

Prepared according to a literature-described procedure[2] from **1c** (418 mg, 1.13 mmol), purified by flash column chromatography (silica gel, PE/EtOAc 70:30).

Yield: 392 mg (844 μmol, 75% over two steps, *dr* > 95:05:0:0 as determined by ^{1}H NMR spectroscopy) as a colorless oil.

R*f* = 0.20 (PE/EtOAc 70:30).

^{1}H NMR (400 MHz, $CDCl_3$): δ 7.26-7.20 (m, 4H), 7.15-7.10 (m, 1H), 5.83 (ddt, *J* = 16.6, 10.2, 6.1 Hz, 1H), 5.05 (dd, *J* = 17.2, 1.8 Hz, 1H), 4.96 (dd, *J* = 10.2, 1.8 Hz, 1H), 4.18 (dt, *J* = 11.0, 7.4 Hz, 1H), 4.13-4.00 (m, 5H), 3.07 (d, *J* = 16.1 Hz, 1H), 2.63 (d, *J* = 16.0 Hz, 1H), 2.23 (dt, *J* = 7.5, 1.7 Hz, 2H), 1.55 (td, *J* = 7.6, 5.6 Hz, 1H), 1.31 (t, *J* = 7.1 Hz, 3H), 1.29 (t, *J* = 7.1 Hz, 3H),1.12 (s, 6H), 1.05 (s, 6H), 0.93 (q, *J* = 7.3 Hz, 1H) ppm.

^{13}C NMR (101 MHz, $CDCl_3$): δ 141.8, 138.0, 128.6, 128.0, 125.7, 114.7, 83.5, 68.3 (d, $^2J_{C,P}$ = 5.7 Hz), 63.7 (d, $^2J_{C,P}$ = 5.6 Hz), 36.4, 34.5, 30.1, 28.5, 28.4, 24.9, 24.6, 16.3, 16.2 ppm.
Note: Carbon atom attached to boron is not visible due to quadrupolar relaxation.

^{11}B NMR (128 MHz, $CDCl_3$): δ 34.4 ppm.

^{31}P NMR (162 MHz, $CDCl_3$): δ –0.9 ppm.

HRMS (APCI+) m/z: calcd. for $C_{24}H_{39}BO_6P^+$ [*M*+H]$^+$: 465.2572, found: 465.2572.

((1*S,2*R**,3*R**)-2,3-Diethyl-2-(4,4,5,5-tetramethyl-1,3,2-dioxaborolan-2-yl)cyclopropyl)methyl diethyl phosphate (2d)**

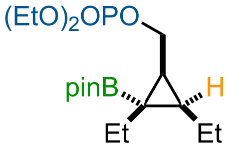

Chemical Formula: $C_{18}H_{36}BO_6P$
Molecular Weight: 390.26

Prepared according to a literature-described procedure[2] from **1d** (850 mg, 2.87 mmol), purified by flash column chromatography (silica gel, PE/EtOAc 70:30).

Yield: 912 mg (1.34 mmol, 82% over two steps, *dr* > 95:05:0:0 as determined by ^{1}H NMR spectroscopy) as a colorless oil.

R*f* = 0.25 (PE/EtOAc 70:30).

^{1}**H NMR** (400 MHz, CDCl3): δ 4.23-4.18 (m, 1H), 4.16-4.04 (m, 5H), 1.48 (dq, J = 14.7, 7.5 Hz, 2H), 1.40-1.35 (m, 2H), 1.32 (t, J = 7.0 Hz, 6H), 1.23 (dd, J = 7.1 Hz, 1H), 1.19 (s, 12H), 1.03 (q, J = 7.5 Hz, 1H), 0.97 (t, J = 7.4 Hz, 3H), 0.93 (t, J = 7.4 Hz, 3H) ppm.

^{13}**C NMR** (101 MHz, $CDCl_3$): δ 83.1, 65.5 (d, $^2J_{C,P}$ = 5.8 Hz), 63.72 (d, $^2J_{C,P}$ = 5.8 Hz), 63.67 (d, $^2J_{C,P}$ = 5.8 Hz), 27.0, 24.73, 24.66, 23.5, 23.4, 17.6, 16.9, 16.3, 16.2, 14.6, 14.1 ppm.
Note: Carbon atom attached to boron is not visible due to quadrupolar relaxation.

^{11}**B NMR** (128 MHz, $CDCl_3$): δ 33.4 ppm.

^{31}**P NMR** (162 MHz, $CDCl_3$): δ –0.8 ppm.

HRMS (APCI+) m/z: calcd. for $C_{18}H_{37}BO_6P^+$ $[M+H]^+$: 391.2415, found: 391.2427.

2.3 Carbinols **3a-g**

Reduction of cyclopropyl esters (GP1):

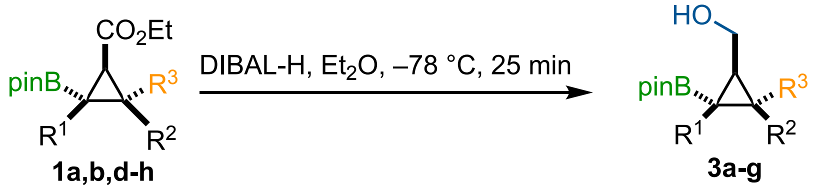

According to a literature procedure,[3] ester **1a,b,d-h** (1.0 equiv.) was dissolved in Et_2O (0.1 M), cooled to –78 °C and DIBAL-H (1.0 M in heptanes, 2.2 equiv.) was added dropwise over 10 min. The solution was stirred for 15 min at –78 °C, carefully quenched with wet MeOH until the evolution of the hydrogen gas ceased, allowed to warm to r.t. and stirred vigorously until the mixture became turbid and a gelous precipitate formed. Celite® (ca 2 g/mmol) and $MgSO_4$ (ca 2 g/mmol) were successively added, the mixture was stirred for further 5-10 min and subsequently filtered through a pad of silica gel (eluent: Et_2O). The filtrate was concentrated under reduced pressure and purified by flash column chromatography (silica gel, gradient PE/Et_2O 80:20 to 70:30) to give the title carbinols.

((1*S,2*R**)-2-Butyl-2-(4,4,5,5-tetramethyl-1,3,2-dioxaborolan-2-yl)cyclopropyl)methanol (3a)**

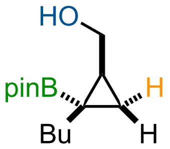

Chemical Formula: $C_{14}H_{27}BO_3$
Molecular Weight: 254.18

Prepared according to general procedure **GP1** from **1a** [2] (1.26 g, 4.14 mmol), purified by flash column chromatography (silica gel, gradient PE/Et_2O 80:20 to 70:30).

Yield: 972 mg (3.82 mmol, 92%, *dr* > 95:05 as determined by ^{1}H NMR spectroscopy) as a colorless oil.

R*f* = 0.25 (PE/Et_2O 1:1).

^{1}**H NMR** (400 MHz, $CDCl_3$): δ 3.70 (dd, J = 11.3, 6.8 Hz, 1H), 3.64 (dd, J = 11.4, 8.0 Hz, 1H), 1.52-1.24 (m, 8H), 1.202 (s, 6H), 1.198 (s, 6H), 0.94-0.78 (m, 1H), 0.88 (t, J = 7.1 Hz, 3H), 0.23 (dd, J = 5.5, 3.8 Hz, 1H) ppm.

^{13}C **NMR** (101 MHz, $CDCl_3$): δ 83.1, 63.2, 32.3, 29.6, 24.9, 24.6, 24.5, 23.2, 15.6, 14.3 ppm.
Note: Carbon atom attached to boron is not visible due to quadrupolar relaxation.

^{11}B **NMR** (128 MHz, $CDCl_3$): δ 33.8 ppm.

HRMS (APCI+) m/z: calcd. for $C_{14}H_{28}BO_3^+$ $[M+H]^+$: 255.2132, found: 255.2143.

((1*S,2*R**)-2-(3-Chloropropyl)-2-(4,4,5,5-tetramethyl-1,3,2-dioxaborolan-2-yl)cyclopropyl)methanol (3b)**

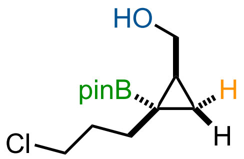

Chemical Formula: $C_{13}H_{24}BClO_3$
Molecular Weight: 274.59

Prepared according to general procedure **GP1** from **1e**[2] (286 mg, 903 μmol), purified by flash column chromatography (silica gel, gradient PE/Et_2O 80:20 to 70:30).

Yield: 165 mg (601 μmol, 67%, *dr* > 95:05 as determined by ^1H NMR spectroscopy) as a colorless oil.

R*f* = 0.25 (PE/Et_2O 1:1).

^1H **NMR** (400 MHz, $CDCl_3$): δ 3.76 (dt, *J* = 12.1, 6.2 Hz, 1H), 3.63 (ddd, *J* = 11.5, 8.2, 5.4 Hz, 1H), 3.55 (t, *J* = 6.7 Hz, 2H), 1.93 (dq, *J* = 8.2, 6.7 Hz, 2H), 1.63-1.53 (m, 1H), 1.48-1.39 (m, 1H), 1.39-1.31 (m, 1H), 1.20 (s, 12H), 0.93 (dd, *J* = 8.3, 3.8 Hz, 1H), 0.89-0.82 (m, 1H), 0.28 (dd, *J* = 5.6, 3.8 Hz, 1H) ppm.

^{13}C **NMR** (101 MHz, $CDCl_3$): δ 83.3, 62.9, 45.6, 32.8, 27.2, 24.9, 24.7, 24.6, 15.7 ppm.
Note: Carbon atom attached to boron is not visible due to quadrupolar relaxation.

^{11}B **NMR** (128 MHz, $CDCl_3$): δ 33.6 ppm.

HRMS (APCI+) m/z: calcd. for $C_{13}H_{23}BClO_2^+$ $[M–H_2O+H]^+$: 257.1480, found: 257.1489.

((1*S,2*R**,3*S**)-3-Allyl-2-butyl-2-(4,4,5,5-tetramethyl-1,3,2-dioxaborolan-2-yl)cyclopropyl)methanol (3c)**

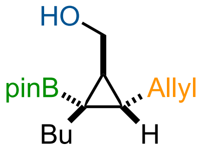

Chemical Formula: $C_{17}H_{31}BO_3$
Molecular Weight: 294.24

Prepared according to general procedure **GP1** from **1b**[2] (1.19 g, 3.54 mmol), purified by flash column chromatography (silica gel, gradient PE/Et_2O 80:20 to 70:30).

Yield: 921 mg (3.13 mmol, 89%, *dr* > 95:05:0:0 as determined by ^1H NMR spectroscopy) as a colorless oil.

R*f* = 0.35 (PE/Et_2O 1:1).

^{1}H NMR (400 MHz, $CDCl_3$): δ 5.85 (ddt, *J* = 16.7, 10.2, 6.3 Hz, 1H), 5.05 (dq, *J* = 17.1, 1.8 Hz, 1H), 4.96 (dd, *J* = 10.3, 2.0 Hz, 1H), 3.72 (ddd, *J* = 11.2, 7.5, 5.5 Hz, 1H), 3.67-3.55 (m, 1H), 2.26-2.09 (m, 2H), 1.63-1.51 (m, 1H), 1.39-1.12 (m, 6H), 1.22 (s, 6H), 1.20 (s, 6H), 0.87 (t, *J* = 7.0 Hz, 3H), 0.66 (q, *J* = 7.0 Hz, 1H) ppm.
Note: The O*H* resonance was not visible.

^{13}C NMR (101 MHz, $CDCl_3$): δ 138.7, 114.6, 83.2, 62.9, 34.6, 32.3, 31.4, 31.3, 30.3, 25.3, 24.6, 23.3, 14.3 ppm.
Note: Carbon atom attached to boron is not visible due to quadrupolar relaxation.

^{11}B NMR (128 MHz, $CDCl_3$): δ 33.9 ppm.

HRMS (APCI+) m/z: calcd. for $C_{17}H_{30}BO_2^+$ [*M*–H_2O+H]$^+$: 277.2339, found: 277.2351.

((1*S,2*R**,3*R**)-2,3-Dimethyl-2-(4,4,5,5-tetramethyl-1,3,2-dioxaborolan-2-yl)cyclopropyl)methanol (3d)**

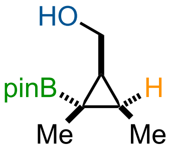

Chemical Formula: $C_{12}H_{23}BO_3$
Molecular Weight: 226.12

Prepared according to general procedure **GP1** from **1f**[2] (1.46 g, 5.45 mmol), purified by flash column chromatography (silica gel, gradient PE/Et_2O 80:20 to 70:30).

Yield: 1.11 g (4.90 mmol, 90%, *dr* > 95:05:0:0 as determined by ^{1}H NMR spectroscopy) as a colorless oil.

R*f* = 0.20 (PE/Et_2O 1:1).

^{1}H NMR (400 MHz, $CDCl_3$): δ 3.68 (d, *J* = 7.5 Hz, 2H), 1.29-1.10 (m, 2H), 1.19 (s, 12H), 1.03 (d, *J* = 6.4 Hz, 3H), 0.98 (s, 3H) ppm.
Note: The O*H* resonance was not visible.

^{13}C NMR (101 MHz, $CDCl_3$): δ 83.2, 59.4, 26.3, 24.8, 18.5, 8.2, 7.8 ppm.
Note: Carbon atom attached to boron is not visible due to quadrupolar relaxation.

^{11}B NMR (128 MHz, $CDCl_3$): δ 33.4 ppm.

HRMS (APCI+) m/z: calcd. for $C_{12}H_{22}BO_2^+$ [*M*–H_2O+H]$^+$: 209.1713, found: 209.1738.

((1*S,2*R**,3*R**)-2-Ethyl-3-phenyl-2-(4,4,5,5-tetramethyl-1,3,2-dioxaborolan-2-yl)cyclopropyl)methanol (3e)**

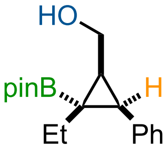

Chemical Formula: $C_{18}H_{27}BO_3$
Molecular Weight: 302.22

Prepared according to general procedure **GP1** from **1g**[2] (2.00 g, 5.81 mmol)), purified by flash column chromatography (silica gel, gradient PE/Et_2O 80:20 to 70:30).

Yield: 1.53 g (5.05 mmol, 86%, *dr* > 95:05:0:0 as determined by ^{1}H NMR spectroscopy) as a colorless oil.

R*f* = 0.30 (PE/Et_2O 1:1).

^{1}H NMR (400 MHz, $CDCl_3$): δ 7.33-7.22 (m, 4H), 7.23-7.15 (m, 1H), 3.78 (d, *J* = 6.8 Hz, 2H), 2.37 (d, *J* = 8.6 Hz, 1H), 1.74-1.58 (m, 2H), 1.35 (s, 1H), 1.25 (s, 6H), 1.23 (s, 6H), 0.97 (t, *J* = 7.0 Hz, 3H), 0.84 (dtd, *J* = 11.6, 6.7, 3.6 Hz, 1H) ppm.

^{13}C NMR (101 MHz, $CDCl_3$): δ 137.0, 130.9, 128.3, 126.2, 83.3, 60.1, 29.7, 27.5, 25.0, 24.5, 20.0, 14.0 ppm.
Note: Carbon atom attached to boron is not visible due to quadrupolar relaxation.

^{11}B NMR (128 MHz, $CDCl_3$): δ 34.0 ppm.

HRMS (APCI+) m/z: calcd. for $C_{18}H_{26}BO_2^+$ [*M*–H_2O+H]$^+$: 285.2026, found: 285.2054.

((1*S,2*R**,3*R**)-2,3-diethyl-2-(4,4,5,5-tetramethyl-1,3,2-dioxaborolan-2-yl)cyclopropyl)methanol (3f)**

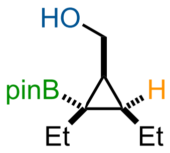

Chemical Formula: $C_{14}H_{27}BO_3$
Molecular Weight: 254.18

Prepared according to general procedure **GP1** from **2d** (650 mg, 2.19 mmol, purified by flash column chromatography (silica gel, gradient PE/Et_2O 80:20 to 70:30).

Yield: 520 mg (2.05 mmol, 93%, *dr* > 95:05:0:0 as determined by ^{1}H NMR spectroscopy) as a colorless oil.

R*f* = 0.35 (PE/Et_2O 1:1).

^{1}H NMR (400 MHz, $CDCl_3$): δ 3.81-3.65 (m, 2H), 2.17 (s, 1H), 1.58-1.30 (m, 4H), 1.27 (q, *J* = 7.8 Hz, 1H), 1.20 (s, 12H), 1.08-0.99 (m, 1H), 1.01-0.93 (m, 6H) ppm.

^{13}C NMR (101 MHz, $CDCl_3$): δ 83.0, 59.6, 26.8, 26.4, 24.74, 24.72, 17.7, 17.0, 14.8, 14.3 ppm.
Note: Carbon atom attached to boron is not visible due to quadrupolar relaxation.

^{11}B NMR (128 MHz, $CDCl_3$): δ 33.5 ppm.

HRMS (APCI+) m/z: calcd. for $C_{14}H_{26}BO_2^+$ [*M*–H_2O+H]$^+$: 237.2020, found: 237.2031.

((1*R,2*S**,3*S**)-2-Allyl-2,3-dimethyl-3-(4,4,5,5-tetramethyl-1,3,2-dioxaborolan-2-yl)cyclopropyl)methanol (3g)**

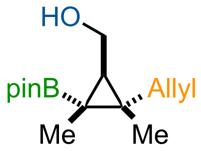

Chemical Formula: $C_{15}H_{27}BO_3$
Molecular Weight: 266.19

Prepared according to general procedure **GP1** from **1h**[2] (924 mg, 3.00 mmol) purified by flash column chromatography (silica gel, gradient PE/Et_2O 80:20 to 70:30).

Yield: 695 mg (2.61 mmol, 87%, *dr* > 95:05:0:0 as determined by ^1H NMR spectroscopy) as a colorless oil.

R*f* = 0.40 (PE/Et_2O 1:1).

^{1}H NMR (400 MHz, $CDCl_3$): δ 5.77 (ddt, *J* = 17.3, 10.5, 6.7 Hz, 1H), 5.06-4.98 (m, 2H), 3.70 (dd, *J* = 11.5, 7.5 Hz, 1H), 3.65 (dd, *J* = 11.5, 7.7 Hz, 1H), 2.17 (dt, *J* = 6.8, 1.4 Hz, 2H), 1.23 (s, 6H), 1.21 (s, 6H), 1.02 (s, 3H), 1.01 (s, 3H), 0.90-0.81 (m, 1H) ppm.
Note: The O*H* resonance was not visible.

^{13}C NMR (101 MHz, $CDCl_3$): δ 137.6, 115.9, 83.4, 60.1, 43.6, 32.8, 27.8, 25.3, 24.7, 13.1, 10.9 ppm.
Note: Carbon atom attached to boron is not visible due to quadrupolar relaxation.

^{11}B NMR (128 MHz, $CDCl_3$): δ 33.3 ppm.

HRMS (APCI+) m/z: calcd. for $C_{15}H_{26}BO_2^+$ [*M*–H_2O+H]$^+$: 249.2026, found: 249.2049.

2.4 Iodides **4a-g**

Appel reaction (GP2):

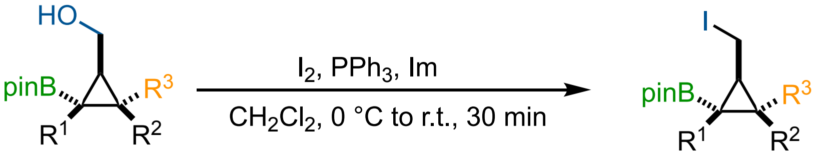

3a-g **4a-g**

Iodine (1.1 equiv.) and triphenylphosphine (1.15 equiv.) were dissolved in dry CH_2Cl_2 (0.2 M), the orange solution was cooled to 0 °C and imidazole (1.2 equiv.) was added. A solution of carbinol **3a-g** (1.0 equiv.) in dry CH_2Cl_2 (0.2 M) was added to the resulting yellow suspension and the reaction mixture was stirred for 15 min at 0 °C and then allowed to warm up to r.t. over 15 min. The mixture was diluted with PE (0.01 M), filtered through a plug of silica gel, which was washed with PE/Et_2O 95:05. The filtrate was concentrated under reduced pressure to give a crude product, which contained the desired iodide and residual impurities. To this residue was added *p*-xylene (0.25 equiv.), the mixture was dissolved in $CDCl_3$ (*ca* 1 mL), homogenized and the content of the title iodide was quantified by ^1H NMR spectroscopy (relaxation delay D1 = 6 s) using the characteristic C*H*2I resonance integration (δ 3.2-3.4 ppm) to determine the yield. The crude product was subsequently re-evaporated twice from toluene, dried in vacuo, and used promptly in the ring-opening step. The analytically pure sample of **4a** was obtained by small-scale column chromatography (silica gel, PE/Et_2O 99:1) and the characteristic resonances of the remaining iodides were assigned in analogy.

2-((1*R,2*S**)-1-Butyl-2-(iodomethyl)cyclopropyl)-4,4,5,5-tetramethyl-1,3,2-dioxaborolane (4a)**

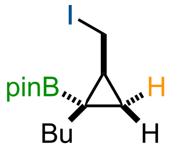

Chemical Formula: $C_{14}H_{26}BIO_2$
Molecular Weight: 364.07

Prepared according to general procedure **GP2** from **3a** (127 mg, 500 μmol).

Yield: 160 mg (440 μmol, 88%, 81% over two steps from **1a**; *dr* > 95:05 as determined by ^{1}H NMR spectroscopy) as a colorless oil.

^{1}H NMR (400 MHz, $CDCl_3$): δ 3.38 (dd, *J* = 9.6, 7.0 Hz, 1H), 3.26 (t, *J* = 9.6 Hz, 1H), 1.64-1.45 (m, 2H), 1.45-1.24 (m, 4H), 1.26-1.14 (m, 1H), 1.19 (s, 12H), 1.07 (dd, *J* = 8.1, 3.9 Hz, 1H), 0.88 (t, *J* = 7.1 Hz, 3H), 0.30-0.18 (m, 1H) ppm.

^{13}C NMR (101 MHz, $CDCl_3$): δ 83.3, 32.3, 29.2, 26.7, 24.8, 24.6, 23.2, 21.8, 14.3, 9.7 ppm.
Note: Carbon atom attached to boron is not visible due to quadrupolar relaxation.

^{11}B NMR (128 MHz, $CDCl_3$): δ 33.2 ppm.

HRMS (APCI+) m/z: calcd. for $C_{14}H_{27}BIO_2^+$ [*M*+H]$^+$: 365.1149, found: 365.1162.

2-((1*R,2*S**)-1-(3-Chloropropyl)-2-(iodomethyl)cyclopropyl)-4,4,5,5-tetramethyl-1,3,2-dioxaborolane (4b)**

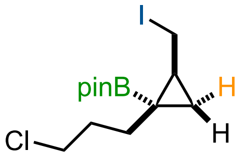

Chemical Formula: $C_{13}H_{23}BClIO_2$
Molecular Weight: 384.49

Prepared according to general procedure **GP2** from **3b** (69 mg, 250 μmol).

Yield: 65 mg (169 μmol, 68%, 46% over two steps from **1e**; *dr* > 95:05 as determined by ^{1}H NMR spectroscopy) as a yellow oil.

^{1}H NMR (400 MHz, $CDCl_3$): δ 3.54 (t, *J* = 6.6 Hz, 2H), 3.31 (dd, *J* = 8.4, 2.4 Hz, 2H), 1.98-1.87 (m, 2H), 1.73-1.65 (m, 1H), 1.64-1.56 (m, 1H), 1.38-1.28 (m, 1H), 1.19 (s, 12H), 1.10 (dd, *J* = 8.2, 4.0 Hz, 1H), 0.28 (dd, *J* = 5.6, 4.0 Hz, 1H) ppm.

^{13}C NMR (101 MHz, $CDCl_3$): δ 83.5, 45.4, 32.8, 26.8, 26.7, 24.8, 24.6, 21.8, 8.6 ppm.
Note: Carbon atom attached to boron is not visible due to quadrupolar relaxation.

^{11}B NMR (128 MHz, $CDCl_3$): δ 33.2 ppm.

HRMS (APCI+) m/z: calcd. for $C_{13}H_{24}BClIO_2^+$ [*M*+H]$^+$: 385.0603 found: 385.0599.

2-((1*R,2*S**,3*S**)-2-Allyl-1-butyl-3-(iodomethyl)cyclopropyl)-4,4,5,5-tetramethyl-1,3,2-dioxaborolane (4c)**

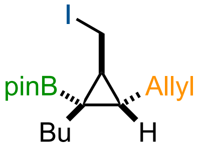

Chemical Formula: $C_{17}H_{30}BIO_2$
Molecular Weight: 404.14

Prepared according to general procedure **GP2** from **3c** (74 mg, 250 μmol).

Yield: 101 mg (150 μmol, 100%, 89% over two steps from **1b**; *dr* > 95:05:0:0 as determined by ^{1}H NMR spectroscopy) as a colorless oil.

^{1}H NMR (400 MHz, $CDCl_3$): δ 5.87 (ddt, *J* = 16.6, 10.2, 6.3 Hz, 1H), 5.03 (dq, *J* = 17.1, 1.7 Hz, 1H), 4.96 (dq, *J* = 10.1, 1.4 Hz, 1H), 3.46 (dd, *J* = 9.6, 6.4 Hz, 1H), 3.22 (t, *J* = 9.8 Hz, 1H), 2.26 (dtt, *J* = 14.8, 6.5, 1.5 Hz, 1H), 2.14 (dddt, *J* = 15.7, 7.9, 6.3, 1.6 Hz, 1H), 1.53 (dtd, *J* = 12.9, 6.6, 3.0 Hz, 2H), 1.39-1.21 (m, 5H), 1.21 (s, 6H), 1.19 (s, 6H), 0.87 (t, *J* = 6.9 Hz, 3H), 0.69-0.57 (m, 1H) ppm.

^{13}C NMR (101 MHz, $CDCl_3$): δ 138.4, 114.6, 83.3, 36.2, 34.5, 33.3, 32.4, 31.1, 25.2, 24.6, 23.3, 14.3, 9.8 ppm.
Note: Carbon atom attached to boron is not visible due to quadrupolar relaxation.

^{11}B NMR (128 MHz, $CDCl_3$): δ 32.6 ppm.

HRMS (APCI+) m/z: calcd. for $C_{17}H_{31}BIO_2^+$ [M+H]$^+$: 405.1462, found: 405.1485.

2-((1*R,2*S**,3*R**)-2-(Iodomethyl)-1,3-dimethylcyclopropyl)-4,4,5,5-tetramethyl-1,3,2-dioxaborolane (4d)**

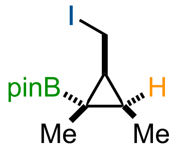

Chemical Formula: $C_{12}H_{22}BIO_2$
Molecular Weight: 336.02

Prepared according to general procedure **GP2** from **3d** (57 mg, 250 μmol).

Yield: 36 mg (107 μmol, 43%, 39% over two steps from **1f**; *dr* > 95:05:0:0 as determined by ^{1}H NMR spectroscopy) as a yellow oil.

^{1}H NMR (400 MHz, $CDCl_3$): δ 3.28 (dd, *J* = 9.9, 7.2 Hz, 1H), 3.17 (t, *J* = 9.7 Hz, 1H), 1.57 (ddd, *J* = 9.6, 8.5, 7.2 Hz, 1H), 1.27-1.20 (m, 1H), 1.19 (s, 12H), 0.97 (d, *J* = 6.6 Hz, 3H), 0.92 (s, 3H) ppm.

^{13}C NMR (101 MHz, $CDCl_3$): δ 83.3, 27.9, 24.82, 24.79, 21.6, 7.2, 6.5, 5.2 ppm.
Note: Carbon atom attached to boron is not visible due to quadrupolar relaxation.

^{11}B NMR (128 MHz, $CDCl_3$): δ 33.5 ppm.

HRMS (APCI+) m/z: calcd. for $C_{12}H_{23}BIO_2^+$ [M+H]$^+$: 337.0830, found: 337.0848.

2-((1*R,2*S**,3*R**)-1-Ethyl-2-(iodomethyl)-3-phenylcyclopropyl)-4,4,5,5-tetramethyl-1,3,2-dioxaborolane (4e)**

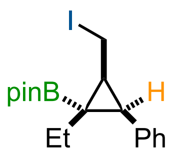

Chemical Formula: $C_{18}H_{26}BIO_2$
Molecular Weight: 412.12

Prepared according to general procedure **GP2** from **3e** (210 mg, 695 μmol).

Yield: 152 mg (368 μmol, 53%, 46% over two steps from **1g**; *dr* > 95:05:0:0 as determined by ^{1}H NMR spectroscopy) along with ring-fragmentation product **S1** (14 mg, 90 μmol, 13%, as determined by ^{1}H NMR spectroscopy) as a yellow oil.

^{1}H NMR (400 MHz, $CDCl_3$): δ 7.41-7.33 (m, 2H), 7.32-7.27 (m, 2H), 7.25-7.18 (m, 1H), 3.42 (dd, *J* = 9.8, 6.2 Hz, 1H), 3.29-3.12 (m, 1H), 2.44 (d, *J* = 8.5 Hz, 1H), 2.03 (td, *J* = 8.6, 6.2 Hz, 1H), 1.64-1.52 (m, 1H), 1.27 (s, 6H), 1.25 (s, 6H), 1.10-1.01 (m, 4H) ppm.

^{13}C NMR (101 MHz, $CDCl_3$): δ 136.1, 131.0, 128.4, 126.5, 83.5, 31.9, 29.9, 25.0, 24.5, 19.3, 14.1, 5.2 ppm. *Note*: Carbon atom attached to boron is not visible due to quadrupolar relaxation.

^{11}B NMR (128 MHz, $CDCl_3$): δ 32.7 ppm.

HRMS (APCI+) m/z: calcd. for $C_{18}H_{27}BIO_2^+$ [*M*+H]$^+$: 413.1143, found: 413.1148.

(2-Ethylbuta-1,3-dien-1-yl)benzene (S1)

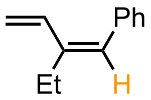

Chemical Formula: $C_{12}H_{14}$
Molecular Weight: 158.24

^{1}H NMR (400 MHz, $CDCl_3$): δ 7.34-7.22 (m, 4H), 7.24-7.09 (m, 1H), 6.42 (s, 1H), 6.37 (dd, *J* = 17.7, 10.9 Hz, 1H), 5.29 (d, *J* = 17.5 Hz, 1H), 5.09 (d, *J* = 10.7 Hz, 1H), 2.44 (q, *J* = 7.6 Hz, 2H), 1.14 (t, *J* = 7.5 Hz, 3H) ppm.

^{13}C NMR (101 MHz, $CDCl_3$): δ 142.3, 140.5, 137.8, 131.1, 128.9, 128.4, 126.8, 113.0, 20.0, 14.0 ppm.

HRMS (APCI+) m/z: calcd. for $C_{12}H_{15}^+$ [*M*+H]$^+$: 159.1168, found: 159.1160.

2-((1*R,2*R**,3*S**)-1,2-Diethyl-3-(iodomethyl)cyclopropyl)-4,4,5,5-tetramethyl-1,3,2-dioxaborolane (4f)**

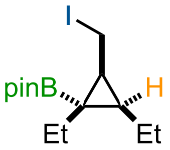

Chemical Formula: $C_{14}H_{26}BIO_2$
Molecular Weight: 364.07

Prepared according to general procedure **GP2** from **3f** (127 mg, 500 μmol).

Yield: 131 mg (360 μmol, 72%, 67% over two steps from **1d**; *dr* > 95:05:0:0 as determined by ^{1}H NMR spectroscopy) as a yellow oil.

^{1}H NMR (400 MHz, $CDCl_3$): δ 3.44 (dd, *J* = 9.8, 6.6 Hz, 1H), 3.23 (t, *J* = 10.0 Hz, 1H), 1.63 (ddd, *J* = 10.0, 8.5, 6.7 Hz, 1H), 1.52-1.33 (m, 4H), 1.20 (s, 12H), 1.12-1.04 (m, 1H), 1.04 (t, *J* = 7.3 Hz, 3H), 0.97 (t, *J* = 7.3 Hz, 3H) ppm.

^{13}C NMR (101 MHz, $CDCl_3$): δ 83.2, 29.6, 28.3, 24.72, 24.67, 17.0, 15.8, 14.8, 14.1, 5.6 ppm. *Note*: Carbon atom attached to boron is not visible due to quadrupolar relaxation.

^{11}B NMR (128 MHz, $CDCl_3$): δ 32.8 ppm.

HRMS (APCI+) m/z: calcd. for $C_{14}H_{27}BIO_2^+$ [*M*+H]$^+$: 365.1149, found: 365.1168.

2-((1*S,2*S**,3*R**)-2-Allyl-3-(iodomethyl)-1,2-dimethylcyclopropyl)-4,4,5,5-tetramethyl-1,3,2-dioxaborolane (4g)**

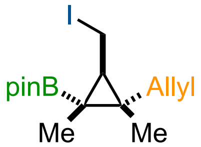

Chemical Formula: $C_{15}H_{26}BIO_2$
Molecular Weight: 376.09

Prepared according to general procedure **GP2** from **3g** (160 mg, 600 μmol).

Yield: 181 mg (480 μmol, 80%, 70% over two steps from **1h**; *dr* > 95:05:0:0 as determined by ^{1}H NMR spectroscopy) as a colorless oil.

^{1}H NMR (400 MHz, $CDCl_3$): δ 5.85-5.73 (m, 1H), 5.03 (h, *J* = 1.0 Hz, 1H), 5.02-4.99 (m, 1H), 3.34 (dd, *J* = 9.8, 7.2 Hz, 1H), 3.20 (t, *J* = 9.7 Hz, 1H), 2.22-2.16 (m, 2H), 1.58 (dd, *J* = 9.6, 7.2 Hz, 1H), 1.24 (s, 6H), 1.22 (s, 6H), 0.98 (s, 3H), 0.97 (s, 3H) ppm.

^{13}C NMR (101 MHz, $CDCl_3$): δ 137.3, 115.9, 83.5, 43.2, 34.5, 31.0, 25.2, 24.7, 11.9, 10.0, 6.2 ppm.
Note: Carbon atom attached to boron is not visible due to quadrupolar relaxation.

^{11}B NMR (128 MHz, $CDCl_3$): δ 33.1 ppm.

HRMS (APCI+) m/z: calcd. for $C_{15}H_{27}BIO_2^+$ [*M*+H]$^+$: 377.1149, found: 377.1167.

3. New experimental procedures and analytical data

3.1 Optimization of the 1,2-metalate rearrangement

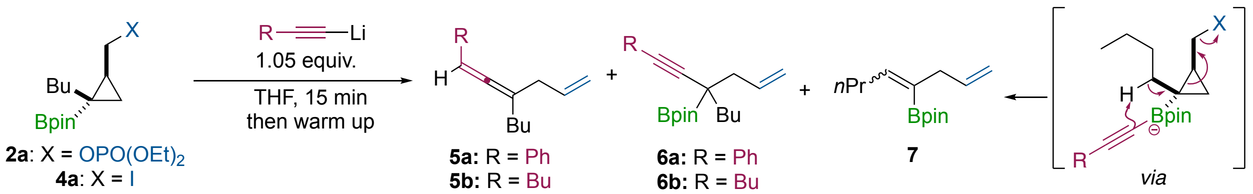

Entry	X	R	Temperature	Time	**5/6/7**[a]	**6a/b** (%)[b]
**1**	OPO(OEt)$_2$	Ph[c]	–78 °C to r.t.	30 min	100:0:0	0
**2**	OPO(OEt)$_2$	Ph	–78 °C to r.t.	30 min	3:52:45	44
**3**	OPO(OEt)$_2$	Ph	–95 °C to r.t.	30 min	7:59:34	46
**4**	I	Ph	–95 °C to –78 °C	30 min	0:93:07	65
**5**	OPO(OEt)$_2$	Bu	–78 °C to r.t.	30 min	0:63:37	51
**6**	OPO(OEt)$_2$	Bu	–95 °C to r.t.	30 min	0:80:20	72
**7**	OPO(OEt)$_2$	Bu	–95 °C to r.t.[d]	30 min	0:41:59	NA
**8**	OPO(OEt)$_2$	Bu	–95 °C to r.t.	2 h	0:64:36	58
**9**	OPO(OEt)$_2$	Bu	–95 °C to r.t.	5 min	0:62:38[e]	33
**10**	OPO(OEt)$_2$	Bu	–95 °C to r.t. [f]	30 min	0:43:57	27
**11**	I	Bu	–95 °C to –78 °C	30 min	0:92:08	85

Table 1. Optimization of the metalate rearrangement-mediated ring opening. [a] Molar ratio according as determined by ^{1}H NMR spectroscopy of the crude mixture. [b] Yield of **6a**/**b** as determined by ^{1}H NMR spectroscopy using an internal standard. [c] With 2.0 equiv. of acetylide. [d] In an excess of **2a** (1.5 equiv. to hexynyllithium). [e] Conversion (**2a**) = 62% according to ^{1}H NMR spectroscopy. [f] In Et_2O instead of THF.

(3-Allylhepta-1,2-dien-1-yl)benzene (5a)

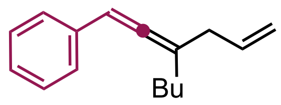

Chemical Formula: $C_{16}H_{20}$
Molecular Weight: 212.34

Physical description: colorless oil.

R*f* = 0.55 (PE 100%).

^{1}H NMR (400 MHz, $CDCl_3$) δ 7.31-7.26 (m, 4H), 7.20-7.14 (m, 1H), 6.14 (quint, *J* = 2.9 Hz, 1H), 5.87 (ddt, *J* = 17.0, 10.0, 6.6 Hz, 1H), 5.11 (dd, *J* = 17.0, 1.7 Hz, 1H), 5.06-5.03 (m, 1H), 2.86 (d, *J* = 6.6 Hz, 2H), 2.11-2.06 (m, 2H), 1.49-1.44 (m, 2H), 1.39-1.33 (m, 2H), 0.89 (t, *J* = 7.2 Hz, 3H) ppm.

^{13}C NMR (101 MHz, $CDCl_3$): δ 202.8, 136.0, 128.6, 126.61, 126.60, 116.2, 107.3, 95.6, 37.8, 32.1, 29.88, 29.86, 22.6, 14.1 ppm.

HRMS (APCI+) m/z: calcd. for $C_{16}H_{21}^+$ [*M*+H]$^+$: 213.1649, found: 213.1643.

4,4,5,5-Tetramethyl-2-(octa-1,4-dien-4-yl)-1,3,2-dioxaborolane (7)

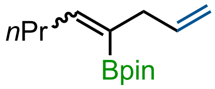

Chemical Formula: $C_{14}H_{25}BO_2$
Molecular Weight: 236.16

R*f* = 0.20 (PE/EtOAc = 50:1).

E-**7**:

^{1}H NMR (400 MHz, $CDCl_3$): δ 6.80-6.66 (m, 1H), 6.37 (t, *J* = 7.1 Hz, 1H), 5.43-5.13 (m, 2H), 2.88 (d, *J* = 5.8 Hz, 2H), 2.20-2.04 (m, 2H), 1.59-1.33 (m, 1H), 1.30-1.14 (m, 1H), 1.22 (s, 12H), 0.86 (t, *J* = 7.3 Hz, 3H) ppm.

^{13}C NMR (101 MHz, $CDCl_3$): δ 147.2, 136.4, 116.0, 83.0, 38.6, 32.8, 25.0, 23.7, 13.9 ppm.
Note: Carbon atom attached to boron is not visible due to quadrupolar relaxation.

Z-**7**:

^{1}H NMR (400 MHz, $CDCl_3$): δ 6.80-6.66 (m, 1H), 6.01 (t, *J* = 7.4 Hz, 1H), 5.43-5.13 (m, 2H), 2.83 (d, *J* = 6.4 Hz, 2H), 2.20-2.04 (m, 2H), 1.59-1.33 (m, 1H), 1.30-1.14 (m, 1H), 1.22 (s, 12H), 0.86 (t, *J* = 7.3 Hz, 3H) ppm.

^{13}C NMR (101 MHz, $CDCl_3$): δ 147.0, 137.6, 113.9, 83.2, 39.3, 30.8, 24.9, 23.7, 14.3 ppm.
Note: Carbon atom attached to boron is not visible due to quadrupolar relaxation.

^{11}B NMR (128 MHz, $CDCl_3$): δ = 30.6 ppm.

HRMS (APCI+) m/z: calcd. for $C_{14}H_{26}BO_2^+$ [*M*+H]$^+$: 237.2021, found: 237.2031.

3.2 Propargylic boronic esters **6a-ae**

Ring opening of borylated cyclopropyl iodides with alkynyl lithium reagents (GP3)

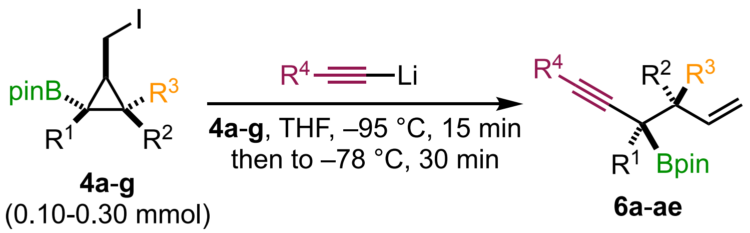

The respective alkyne (1.1-2 equiv. to **4**) was dissolved in dry THF (0.2 M), the solution was cooled to –78 °C (acetone/dry ice) and *n*-BuLi (1.05 equiv. to **4**, 1.2-1.6 M in hexanes) was added. After stirring the solution for 30 min at this temperature, the mixture was cooled to –95 °C (acetone/liquid N_2) and a cold (–78 °C) solution of iodide **4a-g** (1.0 equiv.) in THF (0.2 M), was added over 15 min using a cannula. The solution was subsequently warmed to –78 °C and stirred at this temperature for 30 min. Wet Et_2O (0.02 M) was added, the turbid mixture was allowed to warm up to r.t. and filtered through a plug of silica gel, which was washed with Et_2O. The filtrate was concentrated under reduced pressure and the crude residue was purified by silica gel column chromatography (gradient PE/Et_2O) to give the title tertiary propargylic boronic esters.

4,4,5,5-Tetramethyl-2-(4-(phenylethynyl)oct-1-en-4-yl)-1,3,2-dioxaborolane (6a)

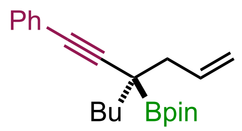

Chemical Formula: $C_{22}H_{31}BO_2$
Molecular Weight: 338.30

Prepared according to general procedure **GP3** from **4a** (93 mg, 163 μmol) and phenylacetylene (21 μL, 196 μmol), purified by flash column chromatography (silica gel, gradient PE/Et_2O 996:4 to 99:1).

Yield: 31 mg (123 μmol, 56%) as a yellow oil.

R*f* = 0.55 (PE/Et_2O 97:3).

^{1}H NMR (400 MHz, $CDCl_3$): δ 7.52-7.35 (m, 2H), 7.34-7.14 (m, 3H), 5.98 (ddt, *J* = 17.2, 10.1, 7.2 Hz, 1H), 5.13 (ddd, *J* = 17.1, 2.5, 1.3 Hz, 1H), 5.06 (ddt, *J* = 10.2, 2.3, 1.1 Hz, 1H), 2.43 (ddt, *J* = 13.5, 7.3, 1.2 Hz, 1H), 2.35 (ddt, *J* = 13.5, 7.1, 1.3 Hz, 1H), 1.72-1.46 (m, 3H), 1.48-1.27 (m, 3H), 1.26 (s, 12H), 0.91 (t, *J* = 7.1 Hz, 3H) ppm.

^{13}C NMR (101 MHz, $CDCl_3$): δ 135.9, 131.9, 128.1, 127.2, 124.9, 117.0, 94.6, 84.0, 82.2, 41.4, 36.4, 28.7, 24.9, 24.8, 23.3, 14.3 ppm.
Note: Carbon atom attached to boron is not visible due to quadrupolar relaxation.

^{11}B NMR (128 MHz, $CDCl_3$): δ 32.5 ppm.

HRMS (APCI+) m/z: calcd. for $C_{22}H_{32}BO_2^+$ [*M*+H]$^+$: 339.2490, found: 339.2498.

2-(5-Allylundec-6-yn-5-yl)-4,4,5,5-tetramethyl-1,3,2-dioxaborolane (6b)

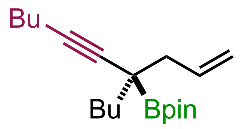

Chemical Formula: $C_{20}H_{35}BO_2$
Molecular Weight: 318.31

Prepared according to general procedure **GP3** from **4a** (81 mg, 222 μmol) and 1-hexyne (31 μL, 266 μmol), purified by flash column chromatography (silica gel, gradient PE/Et_2O 996:4 to 993:7).

Yield: 60 mg (188 μmol, 85%) as a yellow oil.

R*f* = 0.55 (PE/Et_2O 97:3).

^{1}H NMR (400 MHz, $CDCl_3$): δ 5.91 (ddt, *J* = 17.2, 10.1, 7.2 Hz, 1H), 5.06 (ddt, *J* = 17.1, 2.8, 1.4 Hz, 1H), 5.05-4.97 (m, 1H), 2.31 (ddt, *J* = 13.6, 7.4, 1.0 Hz, 1H), 2.22 (ddt, *J* = 13.6, 7.1, 1.1 Hz, 1H), 2.19 (t, *J* = 6.8 Hz, 2H), 1.54-1.37 (m, 6H), 1.35-1.26 (m, 4H), 1.24 (s, 12H), 0.89 (t, *J* = 6.8 Hz, 6H) ppm.

^{13}C NMR (101 MHz, $CDCl_3$): δ 136.3, 116.6, 84.0, 83.8, 81.8, 41.7, 36.6, 31.6, 28.6, 24.9, 24.8, 23.3, 21.9, 18.9, 14.3, 13.8 ppm.
Note: Carbon atom attached to boron is not visible due to quadrupolar relaxation.

^{11}B NMR (128 MHz, $CDCl_3$): δ 33.6 ppm.

HRMS (APCI+) m/z: calcd. for $C_{20}H_{36}BO_2^+$ [*M*+H]$^+$: 319.2803, found: 319.2826.

2-(5-Allyl-2,2-dimethylnon-3-yn-5-yl)-4,4,5,5-tetramethyl-1,3,2-dioxaborolane (6c)

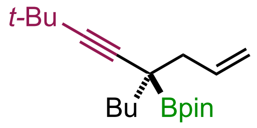

Chemical Formula: $C_{20}H_{35}BO_2$
Molecular Weight: 318.31

Prepared according to general procedure **GP3** from **4a** (65 mg, 178 μmol) and *tert*-butylacetylene (44 μL, 356 μmol), purified by flash column chromatography (silica gel, PE/Et_2O 997:3).

Yield: 47 mg (148 μmol, 83%) as a colorless oil.

R*f* = 0.60 (PE/Et_2O 97:3).

^{1}H NMR (400 MHz, $CDCl_3$): δ 5.88 (ddt, *J* = 17.2, 10.1, 7.2 Hz, 1H), 5.05 (ddt, *J* = 17.2, 2.3, 1.2 Hz, 1H), 4.99 (ddt, *J* = 10.0, 2.3, 0.8 Hz, 1H), 2.29 (ddt, *J* = 13.6, 7.0, 1.2 Hz, 1H), 2.22 (ddt, *J* = 13.5, 7.3, 1.2 Hz, 1H), 1.56-1.38 (m, 2H), 1.36-1.27 (m, 4H), 1.23 (s, 12H), 1.18 (s, 9H), 0.88 (t, *J* = 7.0 Hz, 3H) ppm.

^{13}C NMR (101 MHz, $CDCl_3$): δ 136.3, 116.4, 90.5, 83.6, 82.1, 40.8, 35.6, 31.7, 28.2, 27.6, 24.74, 24.72, 23.3, 14.3 ppm.
Note: Carbon atom attached to boron is not visible due to quadrupolar relaxation.

^{11}B NMR (128 MHz, $CDCl_3$): δ 32.7 ppm.

HRMS (APCI+) m/z: calcd. for $C_{20}H_{36}BO_2^+$ [*M*+H]$^+$: 319.2803, found: 319.2810.

2-(4-(Cyclopropylethynyl)oct-1-en-4-yl)-4,4,5,5-tetramethyl-1,3,2-dioxaborolane (6d)

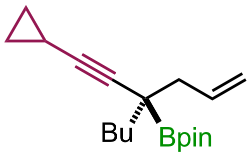

Chemical Formula: $C_{19}H_{31}BO_2$
Molecular Weight: 302.27

Prepared according to general procedure **GP3** from **4a** (54 mg, 148 μmol) and cyclopropylacetylene (14 μL, 180 μmol), purified by flash column chromatography (silica gel, gradient PE/Et_2O 996:4 to 99:1).

Yield: 27 mg (89 μmol, 60%) as a colorless oil.

R*f* = 0.35 (PE/Et_2O 97:3).

^{1}H NMR (400 MHz, $CDCl_3$): δ 5.87 (ddt, *J* = 17.2, 10.1, 7.2 Hz, 1H), 5.18-4.91 (m, 2H), 2.28 (ddt, *J* = 13.5, 7.3, 1.2 Hz, 1H), 2.19 (ddt, *J* = 13.4, 7.1, 1.3 Hz, 1H), 1.56-1.34 (m, 3H), 1.34-1.17 (m, 4H), 1.23 (s, 12H), 0.88 (t, *J* = 7.0 Hz, 3H), 0.72-0.64 (m, 2H), 0.61-0.55 (m, 2H) ppm.

^{13}C NMR (101 MHz, $CDCl_3$): δ 136.2, 116.6, 85.0, 83.8, 79.2, 41.6, 36.6, 28.6, 24.83, 24.80, 23.3, 14.2, 8.6, 0.2 ppm.
Note: Carbon atom attached to boron is not visible due to quadrupolar relaxation.

^{11}B NMR (128 MHz, $CDCl_3$): δ 32.6 ppm.

HRMS (APCI+) m/z: calcd. for $C_{19}H_{32}BO_2^+$ [*M*+H]$^+$: 303.2490, found: 303.2517.

2-(4-(Cyclohexylethynyl)oct-1-en-4-yl)-4,4,5,5-tetramethyl-1,3,2-dioxaborolane (6e)

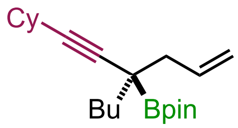

Chemical Formula: $C_{22}H_{37}BO_2$
Molecular Weight: 344.35

Prepared according to general procedure **GP3** from **4a** (80 mg, 220 μmol) and cyclohexylacetylene (33 μL, 253 μmol), purified by flash column chromatography (silica gel, gradient PE/Et_2O 996:4 to 99:1).

Yield: 27 mg (195 μmol, 88%) as a colorless oil.

R*f* = 0.55 (PE/Et_2O 97:3).

^{1}H NMR (400 MHz, $CDCl_3$): δ 5.90 (ddt, *J* = 17.3, 10.1, 7.2 Hz, 1H), 5.05 (ddt, *J* = 17.1, 2.6, 1.4 Hz, 1H), 5.00 (ddt, *J* = 10.1, 2.3, 1.0 Hz, 1H), 2.39 (tt, *J* = 7.8, 3.2 Hz, 1H), 2.30 (ddt, *J* = 13.5, 7.1, 1.3 Hz, 1H), 2.22 (ddt, *J* = 13.5, 7.1, 1.3 Hz, 1H), 1.83-1.61 (m, 4H), 1.58-1.24 (m, 12H), 1.25 (s, 12H), 0.88 (t, *J* = 7.0 Hz, 3H) ppm.

^{13}C NMR (101 MHz, $CDCl_3$): δ 136.3, 116.5, 86.1, 84.2, 83.7, 41.4, 36.3, 33.4, 29.2, 28.5, 26.3, 24.81, 24.79, 24.7, 23.3, 14.3 ppm.

Note: Carbon atom attached to boron is not visible due to quadrupolar relaxation.

^{11}B NMR (128 MHz, $CDCl_3$): δ 32.6 ppm.

HRMS (APCI+) m/z: calcd. for $C_{22}H_{38}BO_2^+$ [*M*+H]$^+$: 345.2965, found: 345.2972.

2-(4-((*p*-Methoxyphenyl)ethynyl)oct-1-en-4-yl)-4,4,5,5-tetramethyl-1,3,2-dioxaborolane (6f)

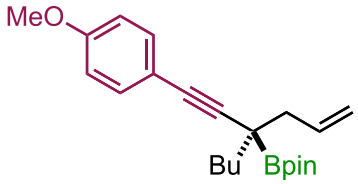

Chemical Formula: $C_{23}H_{33}BO_3$
Molecular Weight: 368.32

Prepared according to general procedure **GP3** from **4a** (77 mg, 210 μmol) and *p*-methoxyphenylacetylene (32 mg, 242 μmol), purified by flash column chromatography (silica gel, gradient PE/Et_2O 996:4 to 98:2).

Yield: 40 mg (109 μmol, 52%) as a light yellow oil.

R*f* = 0.48 (PE/Et_2O 97:3).

^{1}H NMR (400 MHz, $CDCl_3$): δ 7.34 (d, *J* = 8.7 Hz, 2H), 6.79 (d, *J* = 8.7 Hz, 2H), 6.07-5.88 (m, 1H), 5.12 (d, *J* = 17.1 Hz, 1H), 5.06 (d, *J* = 10.1 Hz, 1H), 3.79 (s, 3H), 2.42 (dd, *J* = 13.4, 7.3 Hz, 1H), 2.34 (dd, *J* = 13.5, 7.1 Hz, 1H), 1.70-1.45 (m, 3H), 1.46-1.28 (m, 3H), 1.26 (s, 12H), 0.91 (t, *J* = 7.1 Hz, 3H) ppm.

^{13}C NMR (101 MHz, $CDCl_3$): δ 158.8, 136.0, 133.1, 117.2, 116.9, 113.7, 92.8, 84.0, 81.8, 55.4, 41.4, 36.4, 28.7, 24.9, 24.8, 23.3, 14.2 ppm.
Note: Carbon atom attached to boron is not visible due to quadrupolar relaxation.

^{11}B NMR (128 MHz, $CDCl_3$): δ 32.4 ppm.

HRMS (APCI+) m/z: calcd. for $C_{23}H_{34}BO_3^+$ [*M*+H]$^+$: 369.2596, found: 369.2629.

4,4,5,5-Tetramethyl-2-(4-(*p*-tolylethynyl)oct-1-en-4-yl)-1,3,2-dioxaborolane (6g)

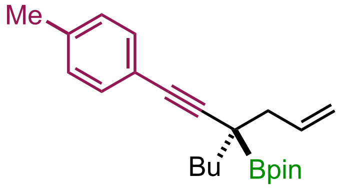

Chemical Formula: $C_{23}H_{33}BO_2$
Molecular Weight: 352.33

Prepared according to general procedure **GP3** from **4a** (107 mg, 293 μmol) and *p*-tolylacetylene (45 μL, 355 μmol), purified by flash column chromatography (silica gel, gradient PE/Et_2O 996:4 to 98:2).

Yield: 60 mg (170 μmol, 58%) as a yellow oil.

R*f* = 0.45 (PE/Et_2O 97:3).

^{1}**H NMR** (400 MHz, $CDCl_3$): δ 7.30 (d, *J* = 8.0 Hz, 2H), 7.06 (d, *J* = 7.8 Hz, 2H), 5.98 (ddt, *J* = 17.1, 10.0, 7.1 Hz, 1H), 5.13 (ddt, *J* = 17.1, 2.6, 1.3 Hz, 1H), 5.06 (dd, *J* = 10.1, 2.3 Hz, 1H), 2.43 (dd, *J* = 13.6, 7.2 Hz, 1H), 2.39-2.29 (m, 1H), 2.32 (s, 3H), 1.72-1.46 (m, 3H), 1.45-1.29 (m, 3H), 1.27 (s, 12H), 0.92 (t, *J* = 7.1 Hz, 3H) ppm.

^{13}**C NMR** (101 MHz, $CDCl_3$): δ 137.1, 136.0, 131.7, 128.8, 121.8, 116.9, 93.7, 84.0, 82.2, 41.4, 36.4, 28.7, 24.9, 24.8, 23.3, 21.5, 14.3 ppm.
Note: Carbon atom attached to boron is not visible due to quadrupolar relaxation.

^{11}**B NMR** (128 MHz, $CDCl_3$): δ 33.4 ppm.

HRMS (APCI+) m/z: calcd. for $C_{23}H_{34}BO_2^+$ [*M*+H]$^+$: 353.2646, found: 353.2648.

4,4,5,5-Tetramethyl-2-(4-((trimethylsilyl)ethynyl)oct-1-en-4-yl)-1,3,2-dioxaborolane (6h)

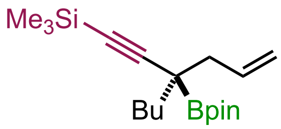

Chemical Formula: $C_{19}H_{35}BO_2Si$
Molecular Weight: 334.38

Prepared according to general procedure **GP3** from **4a** (71 mg, 195 μmol) and trimethylsilylacetylene (55 μL, 390 μmol), purified by flash column chromatography (silica gel, PE/Et_2O 997:3).

Yield: 41 mg (123 μmol, 63%) as a colorless oil.

R*f* = 0.55 (PE/Et_2O 97:3).

^{1}**H NMR** (400 MHz, $CDCl_3$): δ 5.95-5.80 (m, 1H), 5.12-4.99 (m, 2H), 2.33 (dd, *J* = 13.5, 7.1 Hz, 1H), 2.26 (dd, *J* = 13.5, 7.3 Hz, 1H), 1.58-1.41 (m, 2H), 1.41-1.26 (m, 4H), 1.24 (s, 12H), 0.89 (t, *J* = 7.0 Hz, 3H), 0.13 (s, 9H) ppm.

^{13}**C NMR** (101 MHz, $CDCl_3$): δ 135.7, 116.9, 111.6, 85.5, 83.9, 40.7, 35.6, 28.3, 24.8, 24.7, 23.2, 14.2, 0.6 ppm.
Note: Carbon atom attached to boron is not visible due to quadrupolar relaxation.

^{11}**B NMR** (128 MHz, $CDCl_3$): δ 32.4 ppm.

HRMS (APCI+) m/z: calcd. for $C_{19}H_{36}BO_2Si^+$ [*M*+H]$^+$: 335.2572, found: 335.2577.

4,4,5,5-Tetramethyl-2-(4-((triisopropylsilyl)ethynyl)oct-1-en-4-yl)-1,3,2-dioxaborolane (6i)

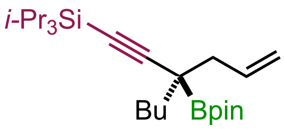

Chemical Formula: $C_{25}H_{47}BO_2Si$
Molecular Weight: 418.54

Prepared according to general procedure **GP3** from **4a** (92 mg, 245 μmol) and triisopropylsilylacetylene (66 μL, 295 μmol), purified by flash column chromatography (silica gel, PE/Et_2O 997:3).

Yield: 67 mg (160 μmol, 65%) as a colorless oil.

R*f* = 0.80 (PE/Et_2O 97:3).

^{1}H NMR (400 MHz, $CDCl_3$): δ 5.90 (ddt, *J* = 17.2, 10.1, 7.2 Hz, 1H), 5.11-5.03 (m, 1H), 5.03-4.97 (m, 1H), 2.38-2.21 (m, 2H), 1.58-1.25 (m, 6H), 1.25 (s, 12H), 1.12-0.94 (m, 21H), 0.88 (t, *J* = 7.1 Hz, 3H) ppm.

^{13}C NMR (101 MHz, $CDCl_3$): δ 136.0, 116.7, 113.2, 83.8, 80.9, 40.9, 35.7, 28.4, 24.8, 23.3, 18.8, 14.3, 11.5 ppm.
Note: Carbon atom attached to boron is not visible due to quadrupolar relaxation.

^{11}B NMR (128 MHz, $CDCl_3$): δ 33.1 ppm.

HRMS (APCI+) m/z: calcd. for $C_{25}H_{48}BO_2Si^+$ [*M*+H]$^+$: 419.3517, found: 419.3518.

2-(5-Allyl-10-(triisopropylsilyl)oxydec-6-yn-5-yl)-4,4,5,5-tetramethyl-1,3,2-dioxaborolane (6j)

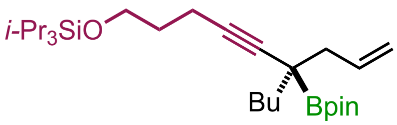

Chemical Formula: $C_{28}H_{53}BO_3Si$
Molecular Weight: 476.62

Prepared according to general procedure **GP3** from **4a** (69 mg, 189 μmol) and 5-(triisopropylsilyl)oxypent-1-yne (52 mg, 217 μmol), purified by flash column chromatography (silica gel, gradient PE/Et_2O 995:5 to 95:5).

Yield: 54 mg (113 μmol, 60%) as a colorless oil.

R*f* = 0.40 (PE/Et_2O 97:3).

^{1}H NMR (400 MHz, $CDCl_3$): δ 5.91 (ddt, *J* = 17.3, 10.2, 7.2 Hz, 1H), 5.05 (ddt, *J* = 17.2, 2.7, 1.4 Hz, 1H), 5.04-4.96 (m, 1H), 3.77 (t, *J* = 6.3 Hz, 2H), 2.35-2.25 (m, 1H), 2.29 (t, *J* = 6.9 Hz, 2H), 2.21 (ddt, *J* = 13.5, 7.0, 1.3 Hz, 1H), 1.71 (p, *J* = 6.6 Hz, 2H), 1.58-1.34 (m, 3H), 1.32-1.14 (m, 3H), 1.23 (s, 12H), 1.16-0.95 (m, 21H), 0.88 (t, *J* = 7.0 Hz, 3H) ppm.

^{13}C NMR (101 MHz, $CDCl_3$): δ 136.3, 116.6, 84.1, 83.8, 81.2, 62.3, 41.8, 36.8, 32.8, 28.7, 24.9, 24.8, 23.3, 18.2, 15.7, 14.2, 12.1 ppm.
Note: Carbon atom attached to boron is not visible due to quadrupolar relaxation.

^{11}B NMR (128 MHz, $CDCl_3$): δ 33.4 ppm.

HRMS (APCI+) m/z: calcd. for $C_{28}H_{54}BO_3Si^+$ [*M*+H]$^+$: 477.3930, found: 477.3948.

2-(5-Allyl-10-(triisopropylsilyl)oxyoct-6-yn-5-yl)-4,4,5,5-tetramethyl-1,3,2-dioxaborolane (6k)

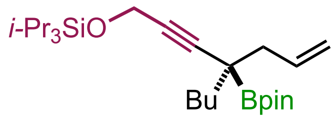

Chemical Formula: $C_{26}H_{49}BO_3Si$
Molecular Weight: 448.57

Prepared according to general procedure **GP3** from **4a** (75 mg, 206 μmol) and 3-(triisopropylsilyl)oxyprop-1-yne (50 mg, 237 μmol), purified by flash column chromatography (silica gel, gradient PE/Et_2O 995:5 to 95:5).
.

Yield: 63 mg (140 μmol, 68%) as a yellow oil.

R*f* = 0.44 (PE/Et_2O 97:3).

^{1}H NMR (400 MHz, $CDCl_3$): δ 5.91 (ddt, *J* = 17.2, 10.1, 7.2 Hz, 1H), 5.11-5.03 (m, 1H), 5.01 (dd, *J* = 10.2, 2.3 Hz, 1H), 4.41 (s, 2H), 2.32 (dd, *J* = 13.5, 7.5 Hz, 1H), 2.25 (dd, *J* = 13.4, 7.0 Hz, 1H), 1.59-1.36 (m, 3H), 1.34-1.18 (m, 3H), 1.23 (s, 12H), 1.17-0.98 (m, 21H), 0.88 (t, *J* = 7.0 Hz, 3H) ppm.

^{13}C NMR (101 MHz, $CDCl_3$): δ 136.0, 116.8, 88.9, 83.9, 80.3, 52.7, 41.6, 36.7, 28.7, 24.88, 24.85, 23.3, 18.2, 14.2, 12.2 ppm.
Note: Carbon atom attached to boron is not visible due to quadrupolar relaxation.

^{11}B NMR (128 MHz, $CDCl_3$): δ 32.7 ppm.

HRMS (APCI+) m/z: calcd. for $C_{26}H_{50}BO_3Si^+$ [*M*+H]$^+$: 449.3617, found: 449.3602.

2-(5-Allyl-10-chlorodec-6-yn-5-yl)-4,4,5,5-tetramethyl-1,3,2-dioxaborolane (6l)

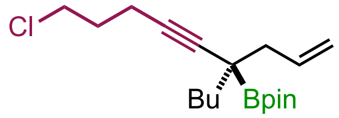

Chemical Formula: $C_{19}H_{32}BClO_2$
Molecular Weight: 338.72

Prepared according to general procedure **GP3** from **4a** (65 mg, 184 μmol) and 5-chloropent-1-yne (24 μL, 210 μmol), purified by flash column chromatography (silica gel, gradient PE/Et_2O 996:4 to 98:2).

Yield: 61 mg (180 μmol, 98%) as a colorless oil.

R*f* = 0.35 (PE/Et_2O 97:3).

^{1}H NMR (400 MHz, $CDCl_3$): δ 5.88 (ddt, *J* = 17.2, 10.1, 7.2 Hz, 1H), 5.11-4.97 (m, 2H), 3.69 (t, *J* = 6.6 Hz, 2H), 2.36 (t, *J* = 6.6 Hz, 2H), 2.30 (ddt, *J* = 13.5, 7.3, 1.2 Hz, 1H), 2.21 (ddt, *J* = 13.4, 7.1, 1.3 Hz, 1H), 1.92 (quint, *J* = 6.6 Hz, 2H), 1.59-1.34 (m, 3H), 1.36-1.24 (m, 3H), 1.25 (s, 12H), 0.89 (t, *J* = 7.0 Hz, 3H) ppm.

^{13}C NMR (101 MHz, $CDCl_3$): δ 136.0, 116.8, 85.6, 83.9, 79.6, 44.0, 41.7, 36.7, 32.3, 28.7, 24.9, 24.8, 23.3, 16.7, 14.2 ppm.
Note: Carbon atom attached to boron is not visible due to quadrupolar relaxation.

^{11}B NMR (128 MHz, $CDCl_3$): δ 33.4 ppm.

HRMS (APCI+) m/z: calcd. for $C_{19}H_{33}BClO_2^+$ [*M*+H]$^+$: 339.2257, found: 339.2241.

2-(4-(3-Chloropropyl)dec-1-en-5-yn-4-yl)-4,4,5,5-tetramethyl-1,3,2-dioxaborolane (6m)

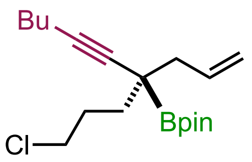

Chemical Formula: $C_{19}H_{32}BClO_2$
Molecular Weight: 338.72

Prepared according to general procedure **GP3** from **4b** (47 mg, 122 μmol) and 1-hexyne (17 μL, 147 μmol), purified by flash column chromatography (silica gel, gradient PE/Et_2O 996:4 to 98:2).

Yield: 29 mg (86 μmol, 70%) as a light yellow oil.

R*f* = 0.45 (PE/Et_2O 97:3).

^{1}H NMR (400 MHz, $CDCl_3$): δ 5.88 (ddt, *J* = 17.3, 10.1, 7.2 Hz, 1H), 5.13-4.98 (m, 2H), 3.54 (t, *J* = 6.8 Hz, 2H), 2.37-2.29 (m, 1H), 2.29-2.22 (m, 1H), 2.18 (t, *J* = 6.7 Hz, 2H), 1.96 (dddd, *J* = 13.7, 11.8, 6.9, 2.0 Hz, 1H), 1.91-1.81 (m, 1H), 1.65 (ddd, *J* = 13.0, 11.5, 4.7 Hz, 1H), 1.57-1.51 (m, 1H), 1.50-1.35 (m, 4H), 1.24 (s, 12H), 0.89 (t, *J* = 7.1 Hz, 3H) ppm.

^{13}C NMR (101 MHz, $CDCl_3$): δ 135.7, 117.1, 84.0, 83.1, 82.5, 45.7, 41.7, 34.0, 31.5, 29.8, 24.86, 24.83, 22.0, 18.8, 13.8 ppm.
Note: Carbon atom attached to boron is not visible due to quadrupolar relaxation.

^{11}B NMR (128 MHz, $CDCl_3$): δ 32.9 ppm.

HRMS (APCI+) m/z: calcd. for $C_{19}H_{33}BClO_2^+$ [*M*+H]$^+$: 339.2257, found: 339.2244.

2-((4*S,5*S**)-5-Butyl-4-vinylundec-1-en-6-yn-5-yl)-4,4,5,5-tetramethyl-1,3,2-dioxaborolane (6n)**

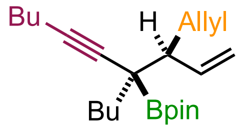

Chemical Formula: $C_{23}H_{39}BO_2$
Molecular Weight: 358.37

Prepared according to general procedure **GP3** from **4c** (93 mg, 230 μmol) and 1-hexyne (32 μL, 276 μmol), purified by flash column chromatography (silica gel, gradient PE/Et_2O 996:4 to 99:1).

Yield: 52 mg (145 μmol, 63%, *dr* > 95:05 as determined by ^{1}H NMR spectroscopy) as a colorless oil.

R*f* = 0.55 (PE/Et_2O 97:3).

^{1}H NMR (400 MHz, $CDCl_3$): δ 5.80-5.64 (m, 2H), 5.03 (dd, *J* = 10.3, 2.3 Hz, 1H), 5.02-4.90 (m, 3H), 2.60-2.47 (m, 1H), 2.21 (t, *J* = 6.6 Hz, 2H), 2.19-2.02 (m, 2H), 1.57-1.36 (m, 8H), 1.35-1.23 (m, 2H), 1.24 (s, 6H), 1.23 (s, 6H), 0.91 (t, *J* = 7.1 Hz, 3H), 0.89 (t, *J* = 7.1 Hz, 3H) ppm.

^{13}C NMR (101 MHz, $CDCl_3$): δ 140.2, 138.4, 116.4, 115.1, 83.8, 83.2, 82.4, 51.1, 35.8, 35.2, 31.7, 29.0, 25.1, 24.9, 23.4, 22.0, 19.0, 14.3, 13.8 ppm.

Note: Carbon atom attached to boron is not visible due to quadrupolar relaxation.

^{11}B NMR (128 MHz, $CDCl_3$): δ 32.4 ppm.

HRMS (APCI+) m/z: calcd. for $C_{23}H_{40}BO_2^+$ [*M*+H]$^+$: 359.3116, found: 359.3127.

2-((4*S,5*S**)-5-(cyclohexylethynyl)-4-vinylnon-1-en-5-yl)-4,4,5,5-tetramethyl-1,3,2-dioxaborolane (6o)**

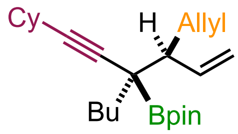

Chemical Formula: $C_{25}H_{41}BO_2$
Molecular Weight: 384.41

Prepared according to general procedure **GP3** from **4c** (75 mg, 186 μmol) and cyclohexylacetylene (38 μL, 291 μmol), purified by flash column chromatography (silica gel, gradient PE/Et_2O 996:4 to 99:1).

Yield: 46 mg (120 μmol, 65%, *dr* > 95:05 as determined by ^{1}H NMR spectroscopy) as a colorless oil.

In a scale-up procedure, prepared according to general procedure **GP3** from **4f** (647 mg, 1.60 mmol) and cyclohexylacetylene (251 μL, 1.92 mmol).

Yield: 440 mg (1.15 mmol, 72%, *dr* > 95:05 as determined by ^{1}H NMR spectroscopy) as a colorless oil.

R*f* = 0.60 (PE/Et_2O 97:3).

^{1}H NMR (400 MHz, $CDCl_3$): δ 5.80-5.65 (m, 2H), 5.02 (dd, *J* = 10.2, 2.3 Hz, 1H), 5.00-4.89 (m, 3H), 2.61-2.49 (m, 1H), 2.44 (tt, *J* = 8.5, 3.2 Hz, 1H), 2.19-2.04 (m, 2H), 1.72 (quintt, *J* = 7.0, 3.0 Hz, 4H), 1.59-1.39 (m, 6H), 1.40-1.24 (m, 6H), 1.231 (s, 6H), 1.229 (s, 6H), 0.89 (t, *J* = 7.0 Hz, 3H) ppm.

^{13}C NMR (101 MHz, $CDCl_3$): δ 140.2, 138.5, 116.3, 115.1, 86.8, 83.7, 83.4, 51.1, 35.6, 35.3, 33.4, 29.2, 28.8, 26.3, 25.1, 24.9, 24.6, 23.4, 14.3 ppm.
Note: Carbon atom attached to boron is not visible due to quadrupolar relaxation.

^{11}B NMR (128 MHz, $CDCl_3$): δ 32.4 ppm.

HRMS (APCI+) m/z: calcd. for $C_{25}H_{42}BO_2^+$ [*M*+H]$^+$: 385.3272, found: 385.3305.

2-((4*S,5*S**)-5-Butyl-8,8-dimethyl-4-vinylnon-1-en-6-yn-5-yl)-4,4,5,5-tetramethyl-1,3,2-dioxaborolane (6p)**

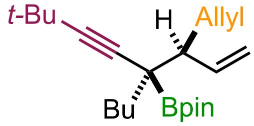

Chemical Formula: $C_{23}H_{39}BO_2$
Molecular Weight: 358.37

Prepared according to general procedure **GP3** from **4c** (95 mg, 236 μmol) and *tert*-butylacetylene (58 μL, 472 μmol), purified by flash column chromatography (silica gel, gradient PE/Et_2O 996:4 to 997:3).

Yield: 63 mg (176 μmol, 75%, *dr* > 95:05 as determined by ^{1}H NMR spectroscopy) as a colorless oil.

R*f* = 0.75 (PE/Et_2O 97:3).

^{1}H NMR (400 MHz, $CDCl_3$): δ 5.80-5.62 (m, 2H), 5.02 (dd, *J* = 10.2, 1.9 Hz, 1H), 4.99-4.87 (m, 3H), 2.51 (dt, *J* = 15.6, 7.8 Hz, 1H), 2.19-2.05 (m, 2H), 1.55-1.37 (m, 3H), 1.34-1.24 (m, 3H), 1.22 (s, 12H), 1.20 (s, 9H), 0.89 (t, *J* = 7.0 Hz, 3H) ppm.

^{13}C NMR (101 MHz, $CDCl_3$): δ 140.2, 138.7, 116.3, 114.9, 91.4, 83.6, 81.4, 50.9, 35.5, 35.1, 31.7, 28.6, 27.7, 25.0, 24.9, 23.4, 14.3 ppm.
Note: Carbon atom attached to boron is not visible due to quadrupolar relaxation.

^{11}B NMR (128 MHz, $CDCl_3$): δ 33.1 ppm.

HRMS (APCI+) m/z: calcd. for $C_{23}H_{40}BO_2^+$ [*M*+H]$^+$: 359.3116, found: 359.3129.

4,4,5,5-Tetramethyl-2-((4*S,5*S**)-5-(prop-1-yn-1-yl)-4-vinylnon-1-en-5-yl)-1,3,2-dioxaborolane (6q)**

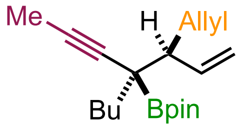

Chemical Formula: $C_{20}H_{33}BO_2$
Molecular Weight: 316.29

Prepared according to general procedure **GP3** from **4c** (95 mg, 234 μmol) and propyne (3-4% w/w in heptane, 625 mg, 0.46-0.62 mmol), purified by flash column chromatography (silica gel, gradient PE/Et_2O 996:4 to 98:2).

Yield: 50 mg (158 μmol, 68%, *dr* > 95:05 as determined by ^{1}H NMR spectroscopy) as a colorless oil.

R*f* = 0.40 (PE/Et_2O 97:3).

^{1}H NMR (400 MHz, $CDCl_3$): δ 5.79-5.63 (m, 2H), 5.02 (dd, *J* = 10.2, 2.2 Hz, 1H), 5.00-4.86 (m, 3H), 2.57-2.47 (m, 1H), 2.19-2.00 (m, 2H), 1.84 (s, 3H), 1.61-1.41 (m, 3H), 1.35-1.22 (m, 3H), 1.24 (s, 6H), 1.23 (s, 6H), 0.89 (t, *J* = 7.2 Hz, 3H) ppm.

^{13}C NMR (101 MHz, $CDCl_3$): δ 140.1, 138.2, 116.5, 115.2, 83.9, 82.1, 77.4, 51.2, 36.0, 35.0, 29.1, 25.1, 24.9, 23.4, 14.3, 4.2 ppm.
Note: Carbon atom attached to boron is not visible due to quadrupolar relaxation.

^{11}B NMR (128 MHz, $CDCl_3$): δ 32.5 ppm.

HRMS (APCI+) m/z: calcd. for $C_{20}H_{34}BO_2^+$ [*M*+H]$^+$: 317.2646, found: 317.2656.

4,4,5,5-Tetramethyl-2-((4*S,5*S**)-5-(phenylethynyl)-4-vinylnon-1-en-5-yl)-1,3,2-dioxaborolane (6r)**

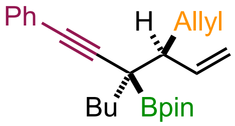

Chemical Formula: $C_{25}H_{35}BO_2$
Molecular Weight: 378.36

Prepared according to general procedure **GP3** from **4c** (95 mg, 235 μmol) and phenylacetylene (31 μL, 282 μmol) using HMPA (250 μL) as a cosolvent, purified by flash column chromatography (silica gel, gradient PE/Et_2O 996:4 to 98:2).

Yield: 69 mg (182 μmol, 78%, *dr* > 95:05 as determined by ^{1}H NMR spectroscopy) as a colorless oil.

R*f* = 0.58 (PE/Et_2O 97:3).

^{1}H NMR (400 MHz, $CDCl_3$): δ 7.43 (dt, *J* = 8.3, 2.1 Hz, 2H), 7.32-7.20 (m, 3H), 5.88-5.69 (m, 2H), 5.13-4.93 (m, 4H), 2.63 (ddt, *J* = 13.8, 7.1, 1.3 Hz, 1H), 2.29 (td, *J* = 10.4, 2.5 Hz, 1H), 2.25-2.15 (m, 1H), 1.72-1.58 (m, 3H), 1.47-1.31 (m, 3H), 1.27 (s, 12H), 0.93 (t, *J* = 7.1 Hz, 3H) ppm.

^{13}C NMR (101 MHz, $CDCl_3$): δ 139.8, 138.1, 131.9, 128.1, 127.2, 125.0, 116.8, 115.4, 94.0, 84.0, 82.9, 51.1, 35.5, 35.4, 29.0, 25.1, 25.0, 23.4, 14.3 ppm.
Note: Carbon atom attached to boron is not visible due to quadrupolar relaxation.

^{11}B NMR (128 MHz, $CDCl_3$): δ 32.3 ppm.

HRMS (APCI+) m/z: calcd. for $C_{25}H_{36}BO_2^+$ [*M*+H]$^+$: 379.2803, found: 379.2803.

4,4,5,5-Tetramethyl-2-((4*S,5*S**)-5-(triisopropylsilyl(ethynyl))-4-vinylnon-1-en-5-yl)-1,3,2-dioxaborolane (6s)**

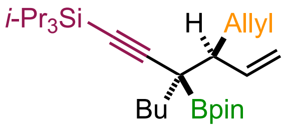

Chemical Formula: $C_{28}H_{51}BO_2Si$
Molecular Weight: 458.61

Prepared according to general procedure **GP3** from **4c** (68 mg, 168 μmol) and triisopropylsilylacetylene (45 μL, 203 μmol), purified by flash column chromatography (silica gel, gradient PE/Et_2O 997:3 to 99:1).

Yield: 49 mg (107 μmol, 64%, *dr* > 95:05 as determined by ^{1}H NMR spectroscopy) as a colorless oil.

R*f* = 0.85 (PE/Et_2O 97:3).

^{1}H NMR (400 MHz, $CDCl_3$): δ 5.80-5.62 (m, 2H), 5.03 (dd, *J* = 10.2, 2.2 Hz, 1H), 5.03-4.90 (m, 3H), 2.68-2.52 (m, 1H), 2.21-2.07 (m, 2H), 1.64-1.41 (m, 3H), 1.41-1.23 (m, 3H), 1.22 (s, 12H), 1.13-0.98 (m, 21H), 0.88 (t, *J* = 7.1 Hz, 3H) ppm.

^{13}C NMR (101 MHz, $CDCl_3$): δ 139.8, 138.3, 116.6, 115.2, 112.4, 83.7, 82.0, 50.9, 35.6, 35.1, 28.7, 25.1, 25.0, 23.4, 18.8, 14.3, 11.6 ppm.
Note: Carbon atom attached to boron is not visible due to quadrupolar relaxation.

^{11}B NMR (128 MHz, $CDCl_3$): δ 32.2 ppm.

HRMS (APCI+) m/z: calcd. for $C_{28}H_{52}BO_2Si^+$ [*M*+H]$^+$: 459.3830, found: 459.3817.

2-(3,4-Dimethyldec-1-en-5-yn-4-yl)-4,4,5,5-tetramethyl-1,3,2-dioxaborolane (6t)

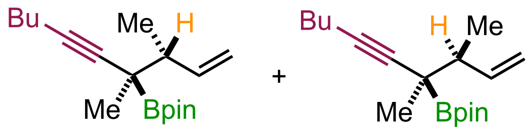

dr 89:11
Chemical Formula: $C_{18}H_{31}BO_2$
Molecular Weight: 290.25

Prepared according to general procedure **GP3** from **4d** (54 mg, 162 μmol) and 1-hexyne (22 μL, 194 μmol), purified by flash column chromatography (silica gel, gradient PE/Et_2O 996:4 to 99:1).

Yield: 30 mg (103 μmol, 61%) as a partially separable mixture of diastereomers (*dr* = 89:11 as determined by ^{1}H NMR spectroscopy) as a colorless oil.

R*f* = 0.53 (PE/Et_2O 97:3).

***(3S*,4R*)*-6t** *(major)*:

^{1}H NMR (400 MHz, $CDCl_3$): δ 5.80 (ddd, *J* = 17.1, 10.3, 8.5 Hz, 1H), 5.06-4.94 (m, 2H), 2.34 (dq, *J* = 13.4, 6.8 Hz, 1H), 2.18 (t, *J* = 6.7 Hz, 2H), 1.52-1.34 (m, 4H), 1.25 (s, 12H), 1.13-1.02 (m, 6H), 0.89 (t, *J* = 7.1 Hz, 3H) ppm.

^{13}C NMR (101 MHz, $CDCl_3$): δ 141.5, 114.8, 83.8, 83.6, 81.6, 44.0, 31.6, 24.8, 24.66, 22.0, 20.8, 18.9, 17.4, 13.8 ppm.
Note: Carbon atom attached to boron is not visible due to quadrupolar relaxation.

***(3S*,4R*)*-6t** *(minor)*:

^{1}H NMR (400 MHz, $CDCl_3$): δ 5.93 (ddd, *J* = 17.2, 10.4, 7.8 Hz, 1H), 5.06-4.94 (m, 2H), 2.34 (dq, *J* = 13.4, 6.8 Hz, 1H), 2.18 (t, *J* = 6.7 Hz, 2H), 1.52-1.34 (m, 4H), 1.24 (s, 12H), 1.13-1.02 (m, 6H), 0.89 (t, *J* = 7.1 Hz, 3H) ppm.

^{13}C NMR (101 MHz, $CDCl_3$): δ 142.1, 114.3, 83.7, 83.6, 81.7, 44.1, 31.6, 24.8, 24.70, 22.0, 20.5, 18.9, 16.0, 13.8 ppm.
Note: Carbon atom attached to boron is not visible due to quadrupolar relaxation.

^{11}B NMR (128 MHz, $CDCl_3$): δ 32.7 ppm.

HRMS (APCI+) m/z: calcd. for $C_{18}H_{32}BO_2^+$ [*M*+H]$^+$: 291.2490, found: 291.2480.

2-((3*R,4*S**)-4-ethyl-3-phenyldec-1-en-5-yn-4-yl)-4,4,5,5-tetramethyl-1,3,2-dioxaborolane (6u)**

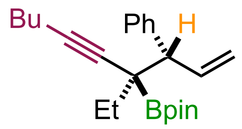

Chemical Formula: $C_{24}H_{35}BO_2$
Molecular Weight: 366.35

Prepared according to general procedure **GP3** from **4e** (95 mg, 231 μmol) and 1-hexyne (32 μL, 277 μmol), purified by flash column chromatography (silica gel, gradient PE/Et_2O 996:4 to 98:2).

Yield: 83 mg (227 μmol, 98%, *dr* > 95:05 as determined by ^{1}H NMR spectroscopy) as a colorless oil.

R*f* = 0.50 (PE/Et_2O 97:3).

^{1}H NMR (400 MHz, $CDCl_3$): δ 7.65-7.47 (m, 2H), 7.31-7.19 (m, 2H), 7.21-7.11 (m, 1H), 6.28 (dt, *J* = 17.0, 9.8 Hz, 1H), 5.06 (dd, *J* = 10.0, 2.1 Hz, 1H), 5.03 (dd, *J* = 16.4, 2.1 Hz, 1H), 3.28 (d, *J* = 9.5 Hz, 1H), 2.30 (t, *J* = 6.9 Hz, 2H), 1.78-1.41 (m, 6H), 1.03 (s, 6H), 1.02 (t, *J* = 7.5 Hz, 3H), 0.94 (s, 6H), 0.93 (t, *J* = 7.2 Hz, 3H) ppm.

^{13}C NMR (101 MHz, $CDCl_3$): δ 143.5, 140.0, 129.0, 128.0, 126.5, 115.7, 83.7 (3C), 82.8, 56.3, 31.6, 31.1, 25.0, 24.5, 22.0, 19.1, 13.8, 11.3 ppm.
Note: Carbon atom attached to boron is not visible due to quadrupolar relaxation.

^{11}B NMR (128 MHz, $CDCl_3$): δ 32.6 ppm.

HRMS (APCI+) m/z: calcd. for $C_{24}H_{36}BO_2^+$ $[M+H]^+$: 367.2803, found: 367.2838.

2-((3*R*,*4*S)-3,4-Diethyldec-1-en-5-yn-4-yl)-4,4,5,5-tetramethyl-1,3,2-dioxaborolane (6v)**

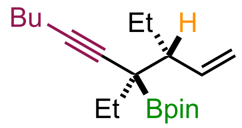

Chemical Formula: $C_{20}H_{35}BO_2$
Molecular Weight: 318.31

Prepared according to general procedure **GP3** from **4f** (97 mg, 266 μmol) and 1-hexyne (37 μL, 321 μmol), purified by flash column chromatography (silica gel, gradient PE/Et_2O 996:4 to 99:1).

Yield: 81 mg (254 μmol, 96%, *dr* > 95:05 as determined by ^{1}H NMR spectroscopy) as a colorless oil.

R*f* = 0.48 (PE/Et_2O 97:3).

^{1}H NMR (400 MHz, $CDCl_3$): δ 5.66 (dt, *J* = 17.2, 10.0 Hz, 1H), 5.07 (dd, *J* = 10.2, 2.4 Hz, 1H), 4.96 (dd, *J* = 17.2, 2.3 Hz, 1H), 2.20 (t, *J* = 6.8 Hz, 2H), 1.95 (td, *J* = 8.8, 5.4 Hz, 1H), 1.57-1.35 (m, 6H), 1.262 (s, 6H), 1.256 (s, 6H), 1.24-1.19 (m, 2H), 0.94 (t, *J* = 7.5 Hz, 3H), 0.90 (t, *J* = 7.2 Hz, 3H), 0.83 (t, *J* = 7.4 Hz, 3H) ppm.

^{13}C NMR (101 MHz, $CDCl_3$): δ 139.7, 116.4, 83.8, 82.5, 82.2, 52.2, 31.8, 29.6, 25.8, 25.1, 24.9, 22.0, 19.0, 13.8, 12.5, 11.4 ppm.
Note: Carbon atom attached to boron is not visible due to quadrupolar relaxation.

^{11}B NMR (128 MHz, $CDCl_3$): δ 32.9 ppm.

HRMS (APCI+) m/z: calcd. for $C_{20}H_{36}BO_2^+$ [M+H]$^+$: 319.2808, found: 319.2824.

2-((3*R,4*S**)-3,4-Diethyl-7,7-dimethyloct-1-en-5-yn-4-yl)-4,4,5,5-tetramethyl-1,3,2-dioxaborolane (6w)**

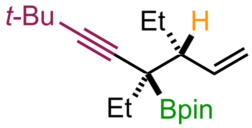

Chemical Formula: $C_{20}H_{35}BO_2$
Molecular Weight: 318.31

Prepared according to general procedure **GP3** from **4f** (79 mg, 217 μmol) and *tert*-butylacetylene (53 μL, 434 μmol), purified by flash column chromatography (silica gel, gradient PE/Et_2O 997:3 to 995:5).

Yield: 60 mg (189 μmol, 87%, *dr* > 95:05 as determined by ^{1}H NMR spectroscopy) as a colorless oil.

R*f* = 0.65 (PE/Et_2O 97:3).

^{1}H NMR (400 MHz, $CDCl_3$): δ 5.65 (dt, *J* = 17.2, 10.0 Hz, 1H), 5.04 (dd, *J* = 10.2, 2.5 Hz, 1H), 4.95 (dd, *J* = 17.1, 2.5 Hz, 1H), 1.96 (td, *J* = 10.2, 2.9 Hz, 1H), 1.56-1.34 (m, 4H), 1.245 (s, 6H), 1.239 (s, 6H), 1.19 (s, 9H), 0.92 (t, *J* = 7.4 Hz, 3H), 0.82 (t, *J* = 7.4 Hz, 3H) ppm.

^{13}C NMR (101 MHz, $CDCl_3$): δ 140.0, 116.2, 91.1, 83.6, 80.7, 51.9, 31.8, 28.8, 27.7, 25.3, 25.0, 24.8, 12.6, 11.2 ppm.
Note: Carbon atom attached to boron is not visible due to quadrupolar relaxation.

^{11}B NMR (128 MHz, $CDCl_3$): δ 32.9 ppm.

HRMS (APCI+) m/z: calcd. for $C_{20}H_{36}BO_{2+}$ [M+H]$^+$: 319.2808, found: 319.2809.

2-(3,4-Diethyl-1-phenylhex-5-en-1-yn-3-yl)-4,4,5,5-tetramethyl-1,3,2-dioxaborolane (6x)

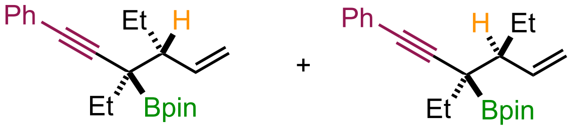

dr 93:07
Chemical Formula: $C_{22}H_{31}BO_2$
Molecular Weight: 338.30

Prepared according to general procedure **GP3** from **4f** (66 mg, 180 μmol) and phenylacetylene (23 μL, 207 μmol), purified by flash column chromatography (silica gel, gradient PE/Et_2O 996:4 to 98:2).

Yield: 57 mg (168 μmol, 94%) as an inseparable mixture of diastereomers (*dr* = 93:07 as determined by ^{1}H NMR spectroscopy) as a colorless oil.

R*f* = 0.40 (PE/Et_2O 97:3).

***(3S*,4R*)*-6x** *(major)*:

^{1}H NMR (400 MHz, $CDCl_3$): δ 7.45-7.39 (m, 2H), 7.30-7.19 (m, 3H), 5.75 (dt, *J* = 17.1, 10.0 Hz, 1H), 5.12 (dd, *J* = 10.2, 2.4 Hz, 1H), 5.03 (dd, *J* = 17.1, 2.3 Hz, 1H), 2.10 (td, *J* = 9.6, 4.3 Hz, 1H), 1.75-1.47 (m, 4H), 1.295 (s, 6H), 1.289 (s, 6H), 1.04 (t, *J* = 7.5 Hz, 3H), 0.88 (t, *J* = 7.4 Hz, 3H) ppm.

^{13}C NMR (101 MHz, $CDCl_3$): δ 139.5, 131.9, 128.1, 127.1, 125.1, 116.8, 93.4, 84.0, 82.7, 52.3, 29.4, 25.9, 25.1, 24.9, 12.5, 11.4 ppm.
Note: Carbon atom attached to boron is not visible due to quadrupolar relaxation.

***(3S*,4S*)*-6x** *(minor)*:

^{1}H NMR (400 MHz, $CDCl_3$): δ 7.46-7.38 (m, 2H), 7.31-7.18 (m, 3H), 5.76 (ddd, *J* = 16.4, 10.2, 6.5 Hz, 1H), 5.09 (dd, *J* = 10.4, 2.5 Hz, 1H), 5.05 (d, *J* = 2.3 Hz, 1H), 2.07-2.02 (m, 1H), 1.75-1.46 (m, 4H), 1.295 (s, 6H), 1.289 (s, 6H), 1.08 (t, *J* = 7.4 Hz, 3H), 0.86 (t, *J* = 7.4 Hz, 3H) ppm.

^{13}C NMR (101 MHz, $CDCl_3$): δ 140.1, 131.9, 128.1, 127.1, 125.1, 116.7, 93.4, 84.0, 82.7, 53.2, 29.8, 25.1, 25.0, 23.4, 12.6, 11.2 ppm.
Note: Carbon atom attached to boron is not visible due to quadrupolar relaxation.

^{11}B NMR (128 MHz, $CDCl_3$): δ 33.4 ppm.

HRMS (APCI+): calcd. for $C_{22}H_{32}BO_2$ $[M+H]^+$: 339.2490, found: 339.2500.

2-(3,4-Diethyl-1-(triisopropylsilyl-5-en-1-yn-3-yl)-4,4,5,5-tetramethyl-1,3,2-dioxaborolane (6y)

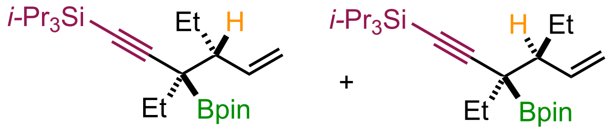

dr 90:10
Chemical Formula: $C_{25}H_{47}BO_2Si$
Molecular Weight: 418.54

Prepared according to general procedure **GP3** from **4f** (66 mg, 180 μmol) and triisopropylsilylacetylene (46 μL, 207 μmol), purified by flash column chromatography (silica gel, gradient PE/Et_2O 997:3 to 99:1).

Yield: 70 mg (167 μmol, 93%) as an inseparable mixture of diastereomers (*dr* = 90:10 as determined by ^{1}H NMR spectroscopy) as a colorless oil.

R*f* = 0.80 (PE/Et_2O 97:3).

***(3S*,4R*)*-6y** *(major)*:

^{1}H NMR (400 MHz, $CDCl_3$): δ 5.69 (dt, *J* = 17.1, 10.0 Hz, 1H), 5.07 (dd, *J* = 10.2, 2.4 Hz, 1H), 4.97 (dd, *J* = 17.0, 2.4 Hz, 1H), 1.97 (td, *J* = 9.2, 4.8 Hz, 1H), 1.59-1.37 (m, 4H), 1.241 (s, 6H), 1.236 (s, 6H), 1.07 (d, *J* = 5.4 Hz, 21H), 0.97 (t, *J* = 7.5 Hz, 3H), 0.84 (t, *J* = 7.4 Hz, 3H) ppm.

^{13}C NMR (101 MHz, $CDCl_3$): δ 139.7, 116.5, 111.8, 83.8, 81.7, 51.8, 28.9, 25.6, 25.1, 24.9, 18.9, 12.5, 11.6, 11.3 ppm.
Note: Carbon atom attached to boron is not visible due to quadrupolar relaxation.

***(3S*,4S*)*-6y** *(minor)*:

^{1}H NMR (400 MHz, $CDCl_3$): δ 5.67 (dt, *J* = 16.8, 10.0 Hz, 1H), 5.03 (dd, *J* = 10.5, 2.7 Hz, 1H), 4.98 (dd, *J* = 16.9, 2.4 Hz, 1H), 1.98-1.90 (m, 1H), 1.59-1.37 (m, 4H), 1.241 (s, 6H), 1.236 (s, 6H), 1.07 (d, *J* = 5.4 Hz, 21H), 0.97 (t, *J* = 7.5 Hz, 3H), 0.82 (t, *J* = 7.1 Hz, 3H) ppm.

^{13}C NMR (101 MHz, $CDCl_3$): δ 140.2, 116.4, 111.8, 83.8, 81.7, 52.9, 28.2, 25.1, 24.9, 23.6, 18.9, 12.6, 11.6, 10.9 ppm.
Note: Carbon atom attached to boron is not visible due to quadrupolar relaxation.

^{11}B NMR (128 MHz, $CDCl_3$): δ 32.7 ppm.

HRMS (APCI+) m/z: calcd. for $C_{25}H_{48}BO_2Si^+$ [*M*+H]$^+$: 419.3511, found: 419.3521.

2-(1-Cyclohexyl-3,4-diethylhex-5-en-1-yn-3-yl)-4,4,5,5-tetramethyl-1,3,2-dioxaborolane (6z)

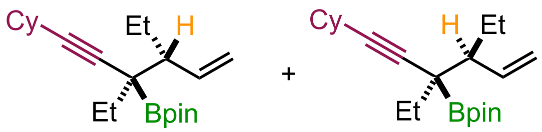

dr 95:05
Chemical Formula: $C_{22}H_{37}BO_2$
Molecular Weight: 344.35

Prepared according to general procedure **GP3** from **4f** (49 mg, 135 μmol) and cyclohexylacetylene (20 μL, 155 μmol), purified by flash column chromatography (silica gel, gradient PE/Et_2O 996:4 to 99:1).

Yield: 35 mg (102 μmol, 75%) as an inseparable mixture of diastereomers (*dr* = 95:05 as determined by ^{1}H NMR spectroscopy) as a colorless oil.

R*f* = 0.55 (PE/Et_2O 97:3).

***(3S*,4R*)*-6z** *(major)*:

^{1}H NMR (400 MHz, $CDCl_3$): δ 5.67 (dt, *J* = 17.1, 10.0 Hz, 1H), 5.06 (dd, *J* = 10.2, 2.5 Hz, 1H), 4.96 (dd, *J* = 17.2, 2.5 Hz, 1H), 2.42 (tt, *J* = 8.1, 3.3 Hz, 1H), 1.96 (ddd, *J* = 9.6, 7.8, 5.6 Hz, 1H), 1.80-1.63 (m, 4H), 1.56-1.36 (m, 6H), 1.39-1.19 (m, 4H), 1.254 (s, 6H), 1.247 (s, 6H), 0.94 (t, *J* = 7.4 Hz, 3H), 0.83 (t, *J* = 7.4 Hz, 3H) ppm.

^{13}C NMR (101 MHz, $CDCl_3$): δ 139.9, 116.3, 86.5, 83.7, 82.7, 52.1, 33.4, 29.4, 29.3, 26.3, 25.7, 25.03, 24.9, 24.7, 12.5, 11.3 ppm.
Note: Carbon atom attached to boron is not visible due to quadrupolar relaxation.

***(3S*,4S*)*-6z** *(minor)*:

^{1}H NMR (400 MHz, $CDCl_3$): δ 5.67 (dt, *J* = 17.1, 10.0 Hz, 1H), 5.01 (dd, *J* = 10.7, 2.8 Hz, 1H), 4.97 (dd, *J* = 16.6, 2.5 Hz, 1H), 2.42 (tt, *J* = 8.1, 3.3 Hz, 1H), 1.97-1.87 (m, 1H), 1.80-1.63 (m, 4H), 1.45 (ddtd, *J* = 13.0, 8.3, 5.7, 2.6 Hz, 6H), 1.39-1.19 (m, 16H), 0.94 (t, *J* = 7.4 Hz, 3H), 0.83 (t, *J* = 7.4 Hz, 3H) ppm.

^{13}C NMR (101 MHz, $CDCl_3$): δ 140.5, 116.2, 86.5, 83.7, 82.7, 53.2, 33.4, 29.8, 29.3, 26.3, 25.7, 25.03, 24.96, 24.7, 12.6, 11.0 ppm.
Note: Carbon atom attached to boron is not visible due to quadrupolar relaxation.

^{11}B NMR (128 MHz, $CDCl_3$): δ 33.1 ppm.

HRMS (APCI+) m/z: calcd. for $C_{22}H_{38}BO_2^+$ [*M*+H]$^+$: 345.2959, found: 345.2972.

2-((4*S,5*S**)-4,5-Dimethyl-4-vinylundec-1-en-6-yn-5-yl)-4,4,5,5-tetramethyl-1,3,2-dioxaborolane (6aa)**

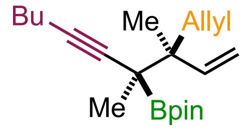

Chemical Formula: $C_{21}H_{35}BO_2$
Molecular Weight: 330.32

Prepared according to general procedure **GP3** from **4g** (92 mg, 245 μmol) and 1-hexyne (34 μL, 294 μmol), purified by flash column chromatography (silica gel, gradient PE/Et_2O 996:4 to 99:1).

Yield: 53 mg (160 μmol, 66%, *dr* > 95:05 as determined by ^{1}H NMR spectroscopy) as a colorless oil.

R*f* = 0.60 (PE/Et_2O 97:3).

^{1}H NMR (400 MHz, $CDCl_3$): δ 5.92 (dd, *J* = 17.5, 10.9 Hz, 1H), 5.69 (dddd, *J* = 16.9, 10.3, 8.7, 5.8 Hz, 1H), 5.08 (dd, *J* = 10.9, 1.6 Hz, 1H), 5.02-4.92 (m, 2H), 4.91 (dd, *J* = 17.5, 1.7 Hz, 1H), 2.51 (dd, *J* = 13.9, 5.8 Hz, 1H), 2.33 (ddt, *J* = 13.8, 8.7, 1.0 Hz, 1H), 2.19 (t, *J* = 6.7 Hz, 2H), 1.53-1.36 (m, 4H), 1.24 (s, 12H), 1.13 (s, 3H), 1.06-1.01 (m, 3H), 0.90 (t, *J* = 7.1 Hz, 3H) ppm.

^{13}C NMR (101 MHz, $CDCl_3$): δ 144.2, 136.6, 116.3, 113.9, 84.1, 83.6, 82.4, 44.5, 42.0, 31.6, 24.74, 24.71, 22.0, 19.8, 18.9, 17.6, 13.8 ppm.
Note: Carbon atom attached to boron is not visible due to quadrupolar relaxation.

^{11}B NMR (128 MHz, $CDCl_3$): δ 32.5 ppm.

HRMS (APCI+) m/z: calcd. for $C_{21}H_{36}BO_2^+$ [*M*+H]$^+$: 331.2803, found: 331.2824.

2-((3*R,4*S**)-3,4-Dimethyl-7,7-dimethyloct-1-en-5-yn-4-yl)-4,4,5,5-tetramethyl-1,3,2-dioxaborolane (6ab)**

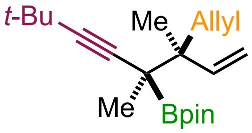

Chemical Formula: $C_{21}H_{35}BO_2$
Molecular Weight: 330.32

Prepared according to general procedure **GP3** from **4g** (77 mg, 204 μmol) and *tert*-butylacetylene (50 μL, 408 μmol), purified by flash column chromatography (silica gel, gradient PE/Et_2O 997:3 to 995:5).

Yield: 48 mg (145 μmol, 71%, *dr* > 95:05 as determined by ^{1}H NMR spectroscopy) as a colorless oil.

R*f* = 0.60 (PE/Et_2O 97:3).

^{1}H NMR (400 MHz, $CDCl_3$): δ 5.88 (dd, *J* = 17.5, 10.8 Hz, 1H), 5.71 (dddd, *J* = 16.4, 10.2, 8.6, 5.9 Hz, 1H), 5.07 (dd, *J* = 10.8, 1.6 Hz, 1H), 5.01-4.94 (m, 2H), 4.90 (dd, *J* = 17.5, 1.6 Hz, 1H), 2.57 (dd, *J* = 14.0, 5.8 Hz, 1H), 2.32 (dd, *J* = 13.9, 8.7 Hz, 1H), 1.23 (s, 12H), 1.19 (s, 9H), 1.10 (s, 3H), 1.03 (s, 3H) ppm.

^{13}C NMR (101 MHz, $CDCl_3$): δ 144.4, 136.9, 116.2, 113.8, 91.1, 83.4, 82.3, 44.5, 42.0, 31.6, 27.7, 24.7, 24.6, 19.6, 17.6 ppm.

Note: Carbon atom attached to boron is not visible due to quadrupolar relaxation.

^{11}B NMR (128 MHz, $CDCl_3$): δ 32.1 ppm.

HRMS (APCI+) m/z: calcd. for $C_{21}H_{36}BO_2^+$ [*M*+H]$^+$: 331.2803, found: 331.2815.

2-((3*S,4*S**)-1-Cyclohexyl-3,4-dimethyl-4-vinylhept-6-en-1-yn-3-yl)-4,4,5,5-tetramethyl-1,3,2-dioxaborolane (6ac)**

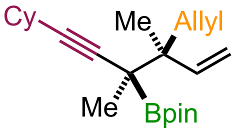

Chemical Formula: $C_{23}H_{37}BO_2$
Molecular Weight: 356.36

Prepared according to general procedure **GP3** from **4g** (92 mg, 245 μmol) and cyclohexylacetylene (38 μL, 294 μmol), purified by flash column chromatography (silica gel, gradient PE/Et_2O 996:4 to 98:2).

Yield: 71 mg (199 μmol, 81%, *dr* > 95:05 as determined by ^{1}H NMR spectroscopy) as a colorless oil.

R*f* = 0.70 (PE/Et_2O 97:3).

^{1}H NMR (400 MHz, $CDCl_3$): δ 5.91 (dd, *J* = 17.5, 10.8 Hz, 1H), 5.78-5.59 (m, 1H), 5.08 (dd, *J* = 10.8, 1.6 Hz, 1H), 5.01-4.94 (m, 2H), 4.91 (dd, *J* = 17.5, 1.6 Hz, 1H), 2.55 (dd, *J* = 13.9, 5.8 Hz, 1H), 2.42 (quint, *J* = 5.7 Hz, 1H), 2.34 (ddt, *J* = 13.9, 8.7, 0.9 Hz, 1H), 1.80-1.66 (m, 4H), 1.45 (q, *J* = 8.9 Hz, 4H), 1.38-1.28 (m, 2H), 1.24 (s, 12H), 1.12 (s, 3H), 1.04 (s, 3H) ppm.

^{13}C NMR (101 MHz, $CDCl_3$): δ 144.3, 136.8, 116.3, 113.9, 86.6, 84.3, 83.6, 44.5, 42.0, 33.3, 29.2, 26.3, 24.7, 24.6, 19.8, 17.6 ppm.
Note: Carbon atom attached to boron is not visible due to quadrupolar relaxation.

^{11}B NMR (128 MHz, $CDCl_3$): δ 32.4 ppm.

HRMS (APCI+) m/z: calcd. for $C_{23}H_{38}BO_2^+$ [*M*+H]$^+$: 357.2959, found: 357.2983.

2-((3*S,4*S**)-3,4-Dimethyl-1-phenyl-4-vinylhept-6-en-1-yn-3-yl)-4,4,5,5-tetramethyl-1,3,2-dioxaborolane (6ad)**

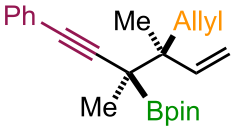

Chemical Formula: $C_{23}H_{31}BO_2$
Molecular Weight: 350.31

Prepared according to general procedure **GP3** from **4g** (90 mg, 240 μmol) and phenylacetylene (30 μL, 276 μmol), purified by flash column chromatography (silica gel, gradient PE/Et_2O 996:4 to 97:3).

Yield: 52 mg (148 μmol, 62%, *dr* > 95:05 as determined by ^{1}H NMR spectroscopy) as a colorless oil.

R*f* = 0.55 (PE/Et_2O 97:3).

^{1}H NMR (400 MHz, $CDCl_3$): δ 7.45-7.37 (m, 2H), 7.32-7.18 (m, 3H), 6.00 (dd, *J* = 17.4, 10.8 Hz, 1H), 5.73 (dddd, *J* = 17.1, 10.2, 8.6, 5.8 Hz, 1H), 5.14 (dd, *J* = 10.8, 1.6 Hz, 1H), 5.06-4.98 (m, 1H), 4.99 (dd, *J* = 17.5, 1.6 Hz, 2H), 2.61 (dd, *J* = 14.0, 5.9 Hz, 1H), 2.43 (ddt, *J* = 13.8, 8.8, 0.9 Hz, 1H), 1.28 (s, 12H), 1.27 (s, 3H), 1.14 (s, 3H) ppm.

^{13}C NMR (101 MHz, $CDCl_3$): δ 143.9, 136.4, 131.8, 128.1, 127.2, 124.9, 116.6, 114.3, 94.8, 83.9, 82.8, 44.8, 42.2, 24.8, 24.7, 19.4, 17.9 ppm.
Note: Carbon atom attached to boron is not visible due to quadrupolar relaxation.

^{11}B NMR (128 MHz, $CDCl_3$): δ 32.8 ppm.

HRMS (APCI+) m/z: calcd. for $C_{23}H_{32}BO_2^+$ [*M*+H]$^+$: 351.2490, found: 351.2476.

2-((3*S,4*S**)-3,4-Dimethyl-1-triisopropylsilyl-4-vinylhept-6-en-1-yn-3-yl)-4,4,5,5-tetramethyl-1,3,2-dioxaborolane (6ae)**

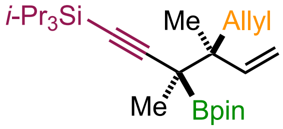

Chemical Formula: $C_{26}H_{47}BO_2Si$
Molecular Weight: 430.56

Prepared according to general procedure **GP3** from **4g** (90 mg, 240 μmol) and triisopropylsilylacetylene (62 μL, 276 μmol), purified by flash column chromatography (silica gel, gradient PE/Et_2O 996:3 to 99:1).

Yield: 65 mg (151 μmol, 63%, *dr* > 95:05 as determined by ^{1}H NMR spectroscopy) as a colorless oil.

R*f* = 0.75 (PE/Et_2O 97:3).

^{1}H NMR (400 MHz, $CDCl_3$): δ 5.89 (dd, *J* = 17.5, 10.8 Hz, 1H), 5.81-5.60 (m, 1H), 5.09 (dd, *J* = 10.8, 1.6 Hz, 1H), 5.02-4.87 (m, 3H), 2.58 (dd, *J* = 14.0, 5.8 Hz, 1H), 2.41 (dd, *J* = 13.9, 8.8 Hz, 1H), 1.23 (s, 12H), 1.17 (s, 3H), 1.12-0.94 (m, 24H) ppm.

^{13}C NMR (101 MHz, $CDCl_3$): δ 144.0, 136.6, 116.4, 114.2, 113.2, 83.6, 81.7, 44.4, 42.2, 24.8, 24.7, 19.5, 18.9, 17.5, 11.6 ppm.
Note: Carbon atom attached to boron is not visible due to quadrupolar relaxation.

^{11}B NMR (128 MHz, $CDCl_3$): δ 32.1 ppm.

HRMS (APCI+) m/z: calcd. for $C_{26}H_{48}BO_2Si^+$ [*M*+H]$^+$: 431.3511, found: 431.3529.

3.3 Propargylic alcohols **8a-k**

Ring opening of borylated cyclopropyl phosphates with alkynyl lithium reagents and *in-situ* oxidation (GP4)

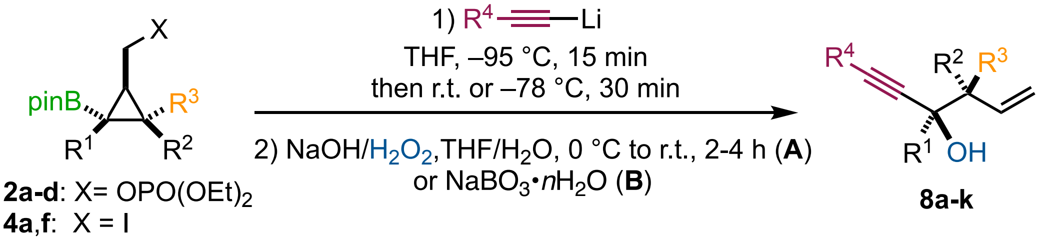

2a-d: X= $OPO(OEt)_2$
4a,**f**: X = I

8a-k

The respective alkyne (1.1 equiv.) was dissolved in THF (0.2 M), the solution was cooled to –78 °C (acetone/liquid N_2) and *n*-BuLi (1.05 equiv., 1.2–1.6 M in hexanes) was added. The solution was stirred for 30 min, cooled to –95 °C and phosphate **2a-d** (1.0 equiv.) in THF (0.2 M), precooled to –95 °C, was quickly added via syringe. The solution was stirred for 15 min at –95 °C and subsequently warmed to room temperature over 30 min. TLC analysis indicated full consumption of phosphate and formation of the intermediate propargylic boronates (R*f*(boronates) ~ 0.5 (PE/Et_2O 97:3)).
Oxidative workup A: After cooling to 0 °C, a mixture of NaOH (3 M aqueous solution, 2 mL/mmol) and H_2O_2 (30%, 1.2 mL/mmol) was added at once. The resulting mixture was vigorously stirred for 2-4 h at room temperature until TLC analysis indicated full consumption of the intermediate boronate. The reaction mixture was diluted with an aqueous solution of saturated Na_2SO_3 and extracted with Et_2O. Combined organic layers were dried over $MgSO_4$, filtered, and concentrated under reduced pressure. The crude residue was purified by flash column chromatography on silica gel to give propargylic alcohols **8**.
Oxidative workup B: After cooling to 0 °C, water (0.1 M) and sodium perborate (5.0 equiv.) were successively added and the mixture was stirred for 4-8 h at 40 °C until TLC analysis indicated full consumption of the intermediate boronate. The reaction mixture was diluted with an aqueous solution of saturated Na_2SO_3 and extracted with Et_2O. Combined organic layers were dried over $MgSO_4$, filtered, and concentrated under reduced pressure. The crude residue was purified by flash column chromatography on silica gel (gradient PE/Et_2O) to give propargylic alcohols **8**.

5-Allylundec-6-yn-5-ol (8a)

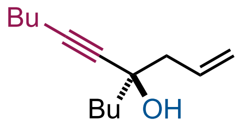

Chemical Formula: $C_{14}H_{24}O$
Molecular Weight: 208.35

Prepared according to general procedure **GP4**, oxidative workup **A**, from **2a** (150 mg, 384 μmol) and 1-hexyne (54 μL, 461 μmol), purified by flash column chromatography (silica gel, gradient PE/Et_2O 90:10 to 80:20).

Yield: 70 mg (336 μmol, 87%) as a colorless oil.

R*f* = 0.30 (PE/EtOAc 50:1).

^{1}H NMR (400 MHz, $CDCl_3$): δ 5.97 (dddd, *J* = 16.9, 10.3, 8.1, 6.5 Hz, 1H), 5.19-5.16 (m, 1H), 2.46 (ddt, *J* = 13.5, 6.5, 1.4 Hz, 1H), 2.31 (ddt, *J* = 13.4, 8.5, 1.4 Hz, 1H), 2.20 (t, *J* = 6.9 Hz, 2H), 2.01 (s, 1H), 1.62-1.61 (m, 2H), 1.50-1.47 (m, 4H), 1.36-1.33 (m, 5H), 0.93 (t, *J* = 7.3 Hz, 3H), 0.91 (t, *J* = 7.2 Hz, 3H) ppm.

^{13}**C NMR** (101 MHz, $CDCl_3$): δ 133.9, 119.2, 85.3, 82.8, 70.3, 47.2, 41.8, 31.0, 26.6, 23.0, 22.0, 18.4, 14.2, 13.7 ppm.

HRMS (APCI+) m/z: calcd. for $C_{14}H_{25}O^+$ [*M*+H]$^+$: 209.1900, found: 209.1909.

4-(Phenylethynyl)oct-1-en-4-ol (8b)

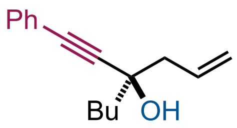

Chemical Formula: $C_{16}H_{20}O$
Molecular Weight: 228.34

Prepared according to general procedure **GP4**, oxidative workup **A**, from **2a** (171 mg, 438 μmol) and phenylacetylene (53 μL, 482 μmol), purified by flash column chromatography (silica gel, gradient PE/Et_2O 90:10 to 70:30).

Yield: 63 mg (215 μmol, 49%) as a colorless oil.

In a scale-up experiment, prepared according to general procedure **GP4**, oxidative workup **A**, from **2a** (933 mg, 2.39 mmol) and phenylacetylene (289 μL, 2.63 mmol).

Yield: 323 mg (1.42 mmol, 59%) as a colorless oil.

R*f* = 0.30 (PE/EtOAc 50:1).

^{1}**H NMR** (400 MHz, $CDCl_3$): δ 7.42 (ddd, *J* = 6.4, 4.5, 2.7 Hz, 2H), 7.31-7.29 (m, 3H), 6.06 (dddd, *J* = 16.8, 10.6, 8.2 Hz, 1H), 5.27-5.20 (m, 2H), 2.60 (ddt, *J* = 13.5, 6.4, 1.3 Hz, 1H), 2.25 (s, 1H), 2.31-2.24 (m, 1H), 1.78-1.75 (m, 1H), 1.61-1.56 (m, 2H), 1.42-1.38 (m, 2H), 1.32-1.28 (m, 1H), 0.87 (t, *J* = 7.3 Hz, 3H) ppm.

^{13}**C NMR** (101 MHz, $CDCl_3$): δ 133.5, 131.8, 128.39, 128.37, 122.9, 119.7, 91.8, 84.7, 70.6, 46.9, 41.6, 26.6, 23.0, 14.2 ppm.

HRMS (APCI+) m/z: calcd. for $C_{16}H_{19}^+$ [*M*–H_2O+H]$^+$: 211.1481, found: 211.1496.

4-((Trimethylsilyl)ethynyl)oct-1-en-4-ol (8c)

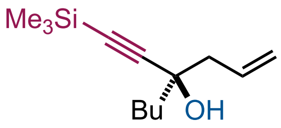

Chemical Formula: $C_{13}H_{24}OSi$
Molecular Weight: 224.42

Prepared according to general procedure **GP4**, oxidative workup **B**, from **2a** (98 mg, 250 μmol) and trimethylsilylacetylene (42 μL, 300 μmol), purified by flash column chromatography (silica gel, gradient PE/Et_2O 95:5 to 90:10).

Yield: 20 mg (89 μmol, 36%) as a colorless oil.

R*f* = 0.35 (PE/EtOAc 50:1).

^{1}**H NMR** (400 MHz, CDCl$_3$): δ 5.96 (ddt, *J* = 16.8, 8.3, 6.4 Hz, 1H), 5.20 (d, *J* = 8.3 Hz, 1H), 5.18 (d, *J* = 16.8 Hz, 1H), 2.48 (dd, *J* = 13.6, 6.4 Hz, 1H), 2.32 (dd, *J* = 13.6, 8.2 Hz, 1H), 2.09 (s, 1H), 1.68-1.61 (m, 2H), 1.51-1.45 (m, 2H), 1.35 (quint, *J* = 7.0 Hz, 2H), 0.92 (t, *J* = 7.3 Hz, 3H), 0.16 (s, 9H) ppm.

^{13}**C NMR** (101 MHz, CDCl$_3$): δ 133.4, 119.6, 108.4, 88.9, 70.4, 46.7, 41.3, 26.5, 22.9, 14.2, 0.1 ppm.

HRMS (APCI+) m/z: calcd. for $C_{13}H_{25}OSi^+$ 225.1669 [*M*+H]$^+$, found: 225.1669.

4-((Triisopropylsilyl)ethynyl)oct-1-en-4-ol (8d)

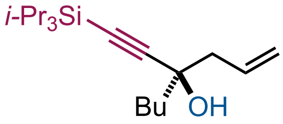

Chemical Formula: $C_{19}H_{36}OSi$
Molecular Weight: 308.58

Prepared according to general procedure **GP4**, oxidative workup **A**, from **2a** (183 mg, 469 µmol) and TIPS-acetylene (126 µL, 563 µmol), purified by flash column chromatography (silica gel, gradient PE/Et$_2$O 95:5 to 90:10).

Yield: 74 mg (240 µmol, 51%) as a colorless oil.

R*f* = 0.35 (PE/EtOAc 50:1).

^{1}**H NMR** (400 MHz, CDCl$_3$): δ 5.99 (dddd, *J* = 16.8, 10.3, 8.2, 6.4 Hz, 1H), 5.20-5.11 (m, 2H), 2.49 (ddt, *J* = 13.5, 6.4, 1.3 Hz, 1H), 2.33 (ddt, *J* = 13.4, 8.2, 1.1 Hz, 1H), 2.09 (s, 1H), 1.67-1.63 (m, 2H), 1.55-1.49 (m, 2H), 1.40-1.34 (m, 2H), 1.08-1.04 (m, 21H), 0.91 (t, *J* = 7.3 Hz, 3H) ppm.

^{13}**C NMR** (101 MHz, CDCl$_3$): δ 133.6, 119.5, 110.4, 84.9, 70.6, 47.1, 41.6, 26.7, 23.0, 18.7, 14.2, 11.3 ppm.

HRMS (APCI+) m/z: calcd. for $C_{19}H_{37}OSi^+$ [*M*+H]$^+$: 309.2608, found: 309.2610.

4-((4-(Trifluoromethyl)phenyl)ethynyl)oct-1-en-4-ol (8e)

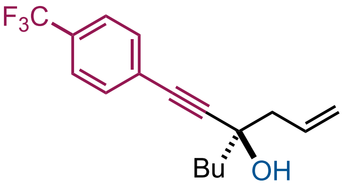

Chemical Formula: $C_{17}H_{19}F_3O$
Molecular Weight: 296.33

Prepared according to general procedure **GP3** from **4a** (103 mg, 284 µmol) and (*p*-trifluoromethyl)phenylacetylene (53 µL, 327 µmol). Thus obtained crude propargylic boronic ester was dissolved in THF (2.8 mL), treated according to oxidative workup **A** described in **GP4** and purified by flash column chromatography (silica gel, gradient PE/Et$_2$O 100:0 to 70:30). This procedure also gave corresponding allene **S2**.

Yield: 25 mg (84 µmol, 30% over two steps) as a colorless oil.

R*f* = 0.55 (PE/Et$_2$O 9:1).

^{1}**H NMR** (400 MHz, $CDCl_3$): δ 7.56 (d, *J* = 8.3 Hz, 2H), 7.51 (d, *J* = 8.3 Hz, 2H), 6.03 (dddd, *J* = 16.8, 10.3, 8.2, 6.5 Hz, 1H), 5.29-5.19 (m, 2H), 2.60 (dd, *J* = 13.6, 6.4 Hz, 1H), 2.44 (dd, *J* = 13.6, 8.2 Hz, 1H), 2.25 (s, 1H), 1.81-1.72 (m, 2H), 1.64-1.51 (m, 2H), 1.39 (h, *J* = 7.3 Hz, 2H), 0.95 (t, *J* = 7.3 Hz, 3H) ppm.

^{13}**C NMR** (101 MHz, $CDCl_3$): δ 133.1, 132.1, 130.2 (q, $J_{C,F}$ = 32.6 Hz), 126.7 (q, $J_{C,F}$ = 1.4 Hz), 125.3 (q, $J_{C,F}$ = 3.8 Hz), 124.0 (q, $J_{C,F}$ = 272.2 Hz), 120.1, 94.4, 83.5, 70.6, 46.8, 41.5, 26.6, 23.0, 14.2 ppm.

^{19}**F NMR** (377 MHz, $CDCl_3$): δ –62.8 ppm.

HRMS (APCI+) m/z: calcd. for $C_{17}H_{18}F_3^+$ [*M*–H_2O+H]$^+$: 279.1361, found: 279.1352.

1-(3-Allyl-hepta-1,2-dien-1-yl)-4-(trifluoromethyl)benzene (S2)

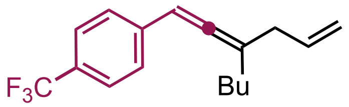

Chemical Formula: $C_{17}H_{19}F_3$
Molecular Weight: 280.33

Yield: 15 mg (54 μmol, 19% over two steps) as a colorless oil.

R*f* = 0.80 (PE 100%).

^{1}**H NMR** (400 MHz, $CDCl_3$): δ 7.53 (d, *J* = 8.1 Hz, 2H), 7.36 (d, *J* = 8.1 Hz, 2H), 6.16 (quint, *J* = 3.0 Hz, 1H), 5.84 (ddt, *J* = 16.9, 10.0, 6.9 Hz, 1H), 5.12 (dq, *J* = 17.0, 1.7 Hz, 1H), 5.04 (dd, *J* = 10.1, 1.8 Hz, 1H), 2.90-2.82 (m, 2H), 2.19-2.03 (m, 2H), 1.52-1.41 (m, 2H), 1.41-1.28 (m, 2H), 0.89 (t, *J* = 7.2 Hz, 3H) ppm.

^{13}**C NMR** (101 MHz, $CDCl_3$): δ 203.8, 140.0, 135.6, 128.5 (q, $J_{C,F}$ = 32.1 Hz), 126.6, 125.6 (q, $J_{C,F}$ = 3.9 Hz), 124.5 (q, $J_{C,F}$ = 271.9 Hz), 116.5, 108.2, 94.9, 37.6, 32.0, 29.8, 22.6, 14.1.

^{19}**F NMR** (377 MHz, $CDCl_3$): δ –62.3 ppm.

HRMS (APCI+) m/z: calcd. for $C_{17}H_{20}F_3^+$ [*M*+H]$^+$: 281.1512, found: 281.1496.

3,4-Diethyldec-1-en-5-yn-4-ol (8f)

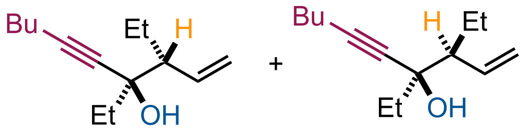

Chemical Formula: $C_{14}H_{24}O$
Molecular Weight: 208.35

Prepared according to general procedure **GP3** from **4f** (93 mg, 256 μmol) and 1-hexyne (34 μL, 294 μmol). Thus obtained crude propargylic boronic ester was dissolved in THF (2.6 mL), treated according to oxidative workup **A** described in **GP4** and purified by flash column chromatography (silica gel, gradient PE/Et_2O 90:10 to 70:30).

Yield: 24 mg (115 μmol, 45% over two steps) as a separable mixture of diastereomers (*dr* = 95:05 as determined by ^{1}H NMR spectroscopy) as a colorless oil.

Synthesis according to general procedure **GP4** from **2d** (197 mg, 505 μmol) and 1-hexyne (64 μL, 555 μmol) gave **8f** (67 mg, 322 μmol, 64% over two steps) as a separable mixture of diastereomers (*dr* = 56:44 as determined by ^{1}H NMR spectroscopy) as colorless oil.

***(3R*,4R*)*-8f** *(major)*:

R*f* = 0.30 (PE/Et_2O 9:1).

^{1}H NMR (400 MHz, $CDCl_3$): δ 5.59 (dt, *J* = 17.1, 10.0 Hz, 1H), 5.13 (dd, *J* = 10.3, 2.2 Hz, 1H), 5.06 (dd, *J* = 17.1, 2.0 Hz, 1H), 2.20 (t, *J* = 6.9 Hz, 2H), 2.04-1.96 (m, 1H), 1.85 (dqd, *J* = 15.0, 7.5, 2.8 Hz, 2H), 1.69 (dq, *J* = 14.9, 7.5 Hz, 1H), 1.58-1.34 (m, 5H), 1.31 (ddt, *J* = 14.7, 11.2, 5.7 Hz, 1H), 1.01 (t, *J* = 7.4 Hz, 3H), 0.91 (t, *J* = 7.2 Hz, 3H), 0.86 (t, *J* = 7.4 Hz, 3H) ppm.

^{13}C NMR (101 MHz, $CDCl_3$): δ 138.6, 117.8, 85.9, 81.6, 73.6, 56.9, 33.4, 31.0, 22.1, 21.8, 18.4, 13.7, 12.3, 8.2 ppm.

HRMS (APCI+) m/z: calcd. for $C_{14}H_{25}O^{+}$ [*M*+H]$^{+}$: 209.1900, found: 209.1901.

***(3R*,4S*)*-8f** *(minor)*:

R*f* = 0.35 (PE/Et_2O 9:1).

^{1}H NMR (400 MHz, $CDCl_3$): δ 5.70 (dt, *J* = 17.2, 10.0 Hz, 1H), 5.24 (dd, *J* = 10.2, 2.2 Hz, 1H), 5.12 (dd, *J* = 17.2, 2.1 Hz, 1H), 2.20 (t, *J* = 6.9 Hz, 2H), 2.07 (s, 1H), 1.95 (td, *J* = 11.2, 2.9 Hz, 1H), 1.82-1.66 (m, 2H), 1.60 (dq, *J* = 14.4, 7.3 Hz, 1H), 1.54-1.30 (m, 5H), 1.02 (t, *J* = 7.4 Hz, 3H), 0.91 (t, *J* = 7.2 Hz, 3H), 0.86 (t, *J* = 7.4 Hz, 3H) ppm.

^{13}C NMR (101 MHz, $CDCl_3$): δ 138.4, 119.3, 85.6, 82.0, 72.9, 56.5, 33.0, 31.1, 22.5, 22.1, 18.5, 13.7, 12.5, 8.4 ppm.

HRMS (APCI+) m/z: calcd. for $C_{14}H_{25}O^{+}$ [*M*+H]$^{+}$: 209.1900, found: 209.1901.

(4*S,5*R**)-5-Butyl-4-vinylundec-1-en-6-yn-5-ol (8g)**

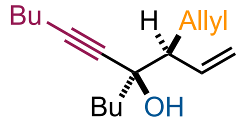

Chemical Formula: $C_{17}H_{28}O$
Molecular Weight: 248.41

Prepared according to general procedure **GP4**, oxidative workup **A**, from **2b** (112 mg, 260 μmol) and 1-hexyne (33 μL, 286 μmol), purified by flash column chromatography (silica gel, gradient PE/Et_2O 90:10 to 70:30).

Yield: 55 mg (221 μmol, 85%, *dr* > 95:05 as determined by ^{1}H NMR spectroscopy) as a colorless oil.

R*f* = 0.30 (PE/EtOAc 25:1).

^{1}H NMR (400 MHz, $CDCl_3$): δ 5.80-5.68 (m, 2H), 5.23 (dd, *J* = 10.2, 2.1 Hz, 1H), 5.11 (dd, *J* = 17.2, 2.1 Hz, 1H), 5.03-4.98 (m, 2H), 2.50-2.45 (m, 1H), 2.24-2.17 (m, 4H), 2.11 (s, 1H), 1.73-1.67 (m, 1H), 1.61-1.56 (m, 1H), 1.50-1.44 (m, 6H), 1.38-1.31 (m, 2H), 0.93 (t, *J* = 7.2 Hz, 3H), 0.90 (t, *J* = 7.2 Hz, 3H) ppm.

^{13}C NMR (101 MHz, $CDCl_3$): δ 137.9, 137.4, 119.3, 115.8, 85.9, 82.0, 72.5, 54.6, 39.9, 34.8, 31.0, 26.2, 23.1, 22.1, 18.4, 14.2, 13.7 ppm.

HRMS (APCI+) m/z: calcd. for $C_{17}H_{29}O^+$ [*M*+H]$^+$: 249.2213, found: 249.2195.

(4*S,5*R**)-5-((Trimethylsilyl)ethynyl)-4-vinylnon-1-en-5-ol (8h)**

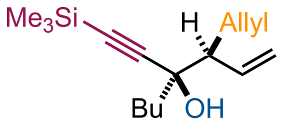

Chemical Formula: $C_{16}H_{28}OSi$
Molecular Weight: 264.48

Prepared according to general procedure **GP4**, oxidative workup **B**, from **2b** (108 mg, 250 μmol) and trimethylsilylacetylene (71 μL, 500 μmol), purified by flash column chromatography (silica gel, gradient PE/Et_2O 95:5 to 80:20).

Yield: 24 mg (91 μmol, 36%, *dr* > 95:05 as determined by ^{1}H NMR spectroscopy) as a colorless oil.

R*f* = 0.50 (PE/EtOAc 25:1).

^{1}H NMR (400 MHz, $CDCl_3$): δ 5.84-5.66 (m, 2H), 5.26 (dd, *J* = 10.2, 2.0 Hz, 1H), 5.12 (dd, *J* = 17.2, 1.9 Hz, 1H), 5.07-4.95 (m, 2H), 2.53-2.41 (m, 1H), 2.31-2.12 (m, 3H), 1.71 (td, *J* = 12.2, 4.5 Hz, 1H), 1.64-1.55 (m, 1H), 1.55-1.41 (m, 2H), 1.34 (h, *J* = 7.2 Hz, 2H), 0.92 (t, *J* = 7.3 Hz, 3H), 0.17 (s, 9H) ppm.

^{13}C NMR (101 MHz, $CDCl_3$): δ 137.5, 137.2, 119.7, 116.0, 107.7, 89.8, 72.7, 54.2, 39.5, 34.8, 26.1, 23.0, 14.2, 0.1 ppm.

HRMS (APCI+) m/z: calcd. for $C_{13}H_{19}^+$ [*M*–TMSOH+H]$^+$: 175.1482, found: 175.1496.

(4*S,5*R**)-5-(Phenylethynyl)-4-vinylnon-1-en-5-ol (8i)**

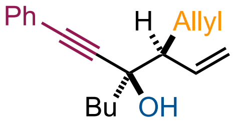

Chemical Formula: $C_{19}H_{24}O$
Molecular Weight: 268.40

Prepared according to general procedure **GP4**, oxidative workup **A**, from **2b** (142 mg, 330 μmol) and phenylacetylene (40 μL, 363 μmol), purified by flash column chromatography (silica gel, gradient PE/Et_2O 90:10 to 70:30). This procedure also gave corresponding allene **S3**.

Yield: 49 mg (183 μmol, 55%, *dr* > 95:05 as determined by ^{1}H NMR spectroscopy) as a colorless oil.

R*f* = 0.25 (PE/EtOAc 25:1).

^{1}H NMR (400 MHz, $CDCl_3$): δ 7.43 (ddd, *J* = 6.7, 2.9, 1.6 Hz, 2H), 7.33-7.30 (m, 3H), 5.84-5.75 (m, 2H), 5.30 (dd, *J* = 10.2, 2.0 Hz, 1H), 5.18 (dd, *J* = 17.2, 2.0 Hz, 1H), 5.07 (dd, *J* = 17.0, 1.7 Hz, 1H), 5.03-5.01 (m, 1H), 2.56 (dddd, *J* = 10.4, 7.5, 4.5, 1.9 Hz, 1H), 2.37-2.34 (m, 1H), 2.31 (t, *J* = 3.9 Hz, 2H), 1.84 (ddd, *J* = 12.8, 11.1, 4.8 Hz, 1H), 1.71 (ddd, *J* = 12.8, 10.6, 5.4 Hz, 1H), 1.59 (dddd, *J* = 12.4, 11.2, 6.2, 3.9 Hz, 2H), 1.39 (quint, *J* = 7.3, 7.3 Hz, 2H), 0.95 (t, *J* = 7.3 Hz, 3H) ppm.

^{13}C NMR (101 MHz, $CDCl_3$): δ 137.6, 137.1, 131.8, 128.42, 128.41, 122.9, 119.8, 116.1, 91.1, 85.5, 72.8, 54.6, 39.7, 34.9, 26.3, 23.1, 14.2 ppm.

HRMS (APCI+) m/z: calcd. for $C_{19}H_{25}O^+$ [*M*+H]$^+$: 269.1900, found: 269.1913.

(3-butyl-4-vinylhepta-1,2,6-trien-1-yl)benzene (S3)

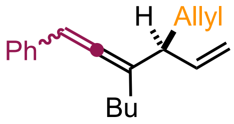

dr 1.5:1
Chemical Formula: $C_{19}H_{24}$
Molecular Weight: 252.40

Yield: 9 mg (36 µmol, 11%) as an inseparable mixture of diastereomers (*dr* = 1.5:1 as determined by ^{1}H NMR spectroscopy) as a colorless oil.

R*f* = 0.80 (PE 100%).

Major diastereomer:

^{1}H NMR (400 MHz, $CDCl_3$): 7.33-7.27 (m, 4H), 7.17 (dt, *J* = 8.7, 4.1 Hz, 1H), 6.21 (td, *J* = 3.1, 1.8 Hz, 1H), 5.78 (ddt, *J* = 13.8, 6.9, 3.5 Hz, 1H), 5.74-5.63 (m, 1H), 5.13-4.95 (m, 4H), 2.71 (tdd, *J* = 8.5, 6.0, 2.1 Hz, 1H), 2.37-2.31 (m, 1H), 2.26-2.15 (m, 1H), 2.14-1.99 (m, 2H), 1.48-1.39 (m, 2H), 1.38-1.31 (m, 2H), 0.872 (t, *J* = 7.2 Hz, 3H) ppm.

^{13}C NMR (101 MHz, $CDCl_3$): δ 202.4, 140.6, 137.0, 135.8, 128.6, 126.64, 126.5, 116.1, 115.19, 111.1, 97.1, 48.0, 38.1, 31.2, 30.0, 22.71, 14.1 ppm.

Minor diastereomer:

^{1}H NMR (400 MHz, $CDCl_3$): 7.33-7.27 (m, 4H), 7.22-7.16 (m, 1H), 6.23 (td, *J* = 3.1, 1.9 Hz, 1H), 5.80 (ddt, *J* = 13.7, 6.9, 3.3 Hz, 1H), 5.74-5.63 (m, 1H), 5.13-4.95 (m, 4H), 2.70-2.61 (m, 1H), 2.37 (ddd, *J* = 7.4, 4.8, 1.3 Hz, 1H), 2.26-2.15 (m, 1H), 2.14-1.99 (m, 2H), 1.48-1.39 (m, 2H), 1.38-1.31 (m, 2H), 0.866 (t, *J* = 7.2 Hz, 3H) ppm.

^{13}C NMR (101 MHz, $CDCl_3$): δ 202.4, 141.0, 137.1, 136.0, 128.7, 126.7, 126.61, 116.0, 115.23, 111.0, 97.2, 47.9, 37.9, 31.1, 29.9, 22.68, 14.1.

HRMS (APCI+) m/z: calcd. for $C_{19}H_{25}^+$ [*M*+H]$^+$: 253.1956, found: 253.1943.

(4*S,5*R**)-5-benzyl-8,8-dimethyl-4-vinylnon-1-en-6-yn-5-ol (8j)**

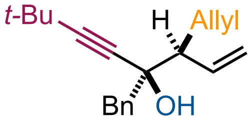

Chemical Formula: $C_{20}H_{26}O$
Molecular Weight: 282.43

Prepared according to general procedure **GP4**, oxidative workup **A**, from **2c** (181 mg, 390 µmol) and *tert*-butylacetylene (96 µL, 780 µmol), purified by flash column chromatography (silica gel, gradient PE/Et_2O 90:10 to 70:30).

Yield: 58 mg (205 µmol, 53%, *dr* > 95:05 as determined by ^{1}H NMR spectroscopy) as a colorless oil.

R*f* = 0.30 (PE/EtOAc 25:1).

^{1}H NMR (400 MHz, $CDCl_3$): δ 7.33-7.26 (m, 5H), 5.84-5.74 (m, 2H), 5.26 (dd, *J* = 10.3, 2.1 Hz, 1H), 5.08-5.01 (m, 3H), 2.93 (q, *J* = 13.3 Hz, 2H), 2.62 (dd, *J* = 12.3, 6.0 Hz, 1H), 2.29-2.20 (m, 2H), 2.03 (s, 1H), 1.19 (s, 9H) ppm.

^{13}C NMR (101 MHz, $CDCl_3$): δ 137.7, 137.4, 136.7, 131.2, 127.9, 126.8, 119.1, 115.9, 95.7, 80.0, 72.4, 54.4, 46.1, 35.0, 31.0, 27.5 ppm.

HRMS (APCI+) m/z: calcd. for $C_{20}H_{25}^+$ [*M*–H_2O+H]$^+$: 265.1951, found: 265.1949.

(3*R,4*S**)-3-benzyl-1-cyclohexyl-4-vinylhept-6-en-1-yn-3-ol (8k)**

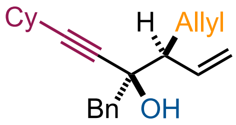

Chemical Formula: $C_{22}H_{28}O$
Molecular Weight: 308.47

Prepared according to general procedure **GP4**, oxidative workup **A**, from **2c** (217 mg, 467 μmol) and cyclohexylacetylene (73 μL, 560 μmol), purified by flash column chromatography (silica gel, gradient PE/Et_2O 90:10 to 70:30).

Yield: 72 mg (233 μmol, 50%, *dr* > 95:05 as determined by ^{1}H NMR spectroscopy) as a colorless oil.

R*f* = 0.25 (PE/EtOAc 25:1).

^{1}H NMR (400 MHz, $CDCl_3$): δ 7.37-7.24 (m, 5H), 5.85-5.75 (m, 2H), 5.26 (dd, *J* = 10.3, 2.1 Hz, 1H), 5.09-4.97 (m, 3H), 2.95 (q, *J* = 13.3 Hz, 2H), 2.63 (dd, *J* = 14.6, 7.9 Hz, 1H), 2.42-2.37 (m, 1H), 2.29-2.22 (m, 2H), 2.06 (s, 1H), 1.79-1.74 (m, 2H), 1.68-1.63 (m, 2H), 1.48-1.39 (m, 3H), 1.30-1.26 (m, 3H) ppm.

^{13}C NMR (101 MHz, $CDCl_3$): δ 137.7, 137.3, 136.7, 131.1, 127.9, 126.7, 119.2, 115.9, 91.5, 81.5, 72.5, 54.4, 46.1, 35.0, 32.7, 32.6, 29.0, 26.0, 24.9 ppm.

HRMS (APCI+) m/z: calcd. for $C_{22}H_{29}O^+$ [*M*+H]$^+$: 309.2213, found: 309.2230.

3.4 Other post-functionalization reactions

((2*S,4*S**)-3-butyl-4-vinylhepta-1,2,6-trien-1-yl)cyclohexane (9a)**

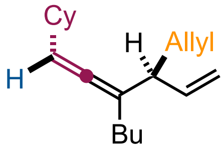

Chemical Formula: $C_{19}H_{30}$
Molecular Weight: 258.45

According to a literature procedure,[5] **6o** (103 mg, 250 μmol) was dissolved in toluene (630 μL) in a dry test tube equipped with a screw cap. Water (20 μL, 1.13 mmol) was added, followed by TBAF (375 μL, 1.0 M in THF, 375 μmol), the tube was sealed and the reaction mixture was stirred at 50 °C overnight. After cooling to r.t., PE (2.0 mL) and water (2.0 mL) were added, the layers were separated and the aqueous one was extracted with PE (2×2 mL) and Et_2O (2 mL). Combined organic layers were washed with water

and brine, dried over Na_2SO_4, filtered, and concentrated under reduced pressure. The crude residue was purified by flash column chromatography (20 mL silica gel, gradient PE/Et_2O 100:0 to 95:5) to give title allene **9a** (46 mg, 180 µmol, 71%, *dr* > 95:05 as determined by ^{1}H NMR spectroscopy) as a colorless oil.

***R*f** = 0.75 (PE 100%).

^{1}H NMR (400 MHz, $CDCl_3$): δ 5.79 (ddt, *J* = 17.1, 10.1, 6.9 Hz, 1H), 5.68-5.55 (m, 1H), 5.15 (dq, *J* = 5.9, 3.5 Hz, 1H), 5.08-4.92 (m, 4H), 2.52 (q, *J* = 7.6 Hz, 1H), 2.31 (dt, *J* = 13.6, 6.3 Hz, 1H), 2.14 (dt, *J* = 15.1, 7.7 Hz, 1H), 2.01-1.82 (m, 3H), 1.82-1.60 (m, 4H), 1.46-1.00 (m, 10H), 0.90 (t, *J* = 7.0 Hz, 3H) ppm.

^{13}C NMR (101 MHz, $CDCl_3$): δ 199.4, 141.1, 137.6, 115.6, 114.5, 107.4, 99.8, 47.5, 38.3, 38.2, 33.6, 31.1, 30.1, 26.39, 26.38, 26.36, 22.7, 14.2 ppm.

HRMS (APCI+): calcd. for $C_{19}H_{31}^+$ [*M*+H]$^+$: 259.2420, found: 259.2432.

((2*R,4*S**)-3-Butyl-1-cyclohexyl-4-vinylhepta-1,2,6-trien-1-yl)benzene (9b)**

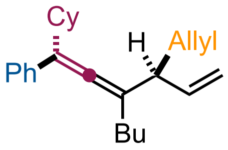

Chemical Formula: $C_{25}H_{34}$
Molecular Weight: 334.55

According to a literature procedure,[5] a flame-dried Schlenk flask was charged with activated 3 Å molecular sieves (100 mg), tris(dibenzylideneacetone)dipalladium(0) (2 mg, 3 μmol), PPh_3 (1 mg, 5 μmol), Ag_2O (35 mg, 150 μmol), and boronic ester **6o** (46 mg, 110 μmol) in dry DME (1 mL) under an argon atmosphere. Iodobenzene (11 μL, 100 μmol) was added and the mixture was stirred at refluxing temperature (85 °C) overnight. After cooling to r.t., the mixture was filtered through a plug of Celite® and washed with CH_2Cl_2. The filtrate was adsorbed on Celite® and purified by flash column chromatography (10 mL silica gel, gradient pentane/Et_2O 1000:0 to 999:1) to give tetrasubstituted allene **9b** (22 mg, 66 μmol, 66% *dr* > 95:05 as determined by ^{1}H NMR spectroscopy) as a colorless oil. Protodeborylation product **9a** (5 mg, 19 μmol, 19%) was also isolated.

***R*f** = 0.70 (PE 100%).

^{1}H NMR (400 MHz, $CDCl_3$): δ 7.39 (d, *J* = 7.3 Hz, 2H), 7.29 (t, *J* = 7.7 Hz, 2H), 7.17 (t, *J* = 7.3 Hz, 1H), 5.80 (ddt, *J* = 17.1, 10.1, 6.9 Hz, 1H), 5.75-5.61 (m, 1H), 5.12-4.95 (m, 4H), 2.68 (td, *J* = 8.7, 5.6 Hz, 1H), 2.51-2.35 (m, 2H), 2.18 (dd, *J* = 14.2, 7.4 Hz, 1H), 2.12-1.97 (m, 2H), 1.98-1.86 (m, 2H), 1.86-1.64 (m, 3H), 1.50-1.25 (m, 6H), 1.28-1.08 (m, 3H), 0.86 (t, *J* = 7.1 Hz, 3H) ppm.

^{13}C NMR (101 MHz, $CDCl_3$): δ 200.3, 141.0, 137.9, 137.5, 128.3, 126.3, 126.2, 115.8, 115.1, 111.2, 48.3, 38.4, 38.1, 33.6, 33.3, 31.2, 30.1, 27.0, 26.6, 22.9, 14.2 ppm.

HRMS (APCI+): calcd. for $C_{25}H_{35}^+$ [*M*+H]$^+$: 335.2733, found: 335.2720.

4,4,5,5-Tetramethyl-2-((*R)-2-methyl-2-((*S**)-3-methylhexa-1,5-dien-3-yl)oct-3-yn-1-yl)-1,3,2-dioxaborolane (10)**

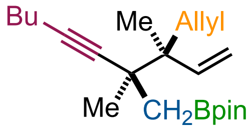

Chemical Formula: $C_{22}H_{37}BO_2$
Exact Mass: 344.29

According to a literature procedure,[6] CH_2ClI (19 μL, 260 μmol) was added to a solution of boronic ester **6aa** (71 mg, 220 μmol) in dry THF (860 μL), the solution was cooled to –78 °C and *n*-BuLi (1.22 M in hexanes; 210 μL, 260 μmol, 1.2 equiv.) was added dropwise. The pale yellow, slightly turbid reaction mixture was stirred for 15 min at this temperature, warmed to r.t. and stirred overnight while becoming dark yellow and homogenous. The mixture was diluted with wet Et_2O (10 mL), filtered through a plug of silica gel and concentrated under reduced pressure. The crude residue was purified by flash column chromatography (10 mL SiO_2, gradient PE/ Et_2O 997:3 to 996:4) to give primary boronic ester **10** (15 mg, 44 μmol, 20%, *dr* > 95:05 as determined by ^{1}H NMR spectroscopy) as a yellow oil.

***R*f** = 0.50 (PE/Et_2O 97:3).

^{1}H NMR (400 MHz, $CDCl_3$): δ 5.96 (dd, *J* = 17.5, 10.9 Hz, 1H), 5.74-5.59 (m, 1H), 5.11 (dd, *J* = 10.9, 1.6 Hz, 1H), 5.01-4.91 (m, 2H), 4.89 (dd, *J* = 17.7, 1.7 Hz, 1H), 2.50 (dd, *J* = 13.7, 5.8 Hz, 1H), 2.20 (dd, *J* = 13.5, 8.7 Hz, 1H), 2.15 (t, *J* = 6.9 Hz, 2H), 1.52-1.36 (m, 4H), 1.28-1.22 (m, 15H), 0.93 (s, 3H), 0.90 (t, *J* = 7.1 Hz, 3H), 0.79 (s, 1H), 0.75 (s, 1H) ppm.

^{13}C NMR (101 MHz, $CDCl_3$): δ 143.7, 137.0, 116.2, 114.5, 86.0, 83.0, 82.3, 46.3, 40.5, 39.4, 31.4, 25.2, 24.8, 23.6, 22.2, 18.7, 16.3, 13.8 ppm.
Note: Carbon atom attached to boron is not visible due to quadrupolar relaxation.

^{11}B NMR (128 MHz, $CDCl_3$): δ 33.6 ppm.

HRMS (APCI+) m/z: calcd. for $C_{22}H_{38}BO_2^+$ [*M*+H]$^+$: 345.2959, found: 345.2967.

4. NMR spectra of new compounds

Ethyl (1*S,2*R**,3*S**)-3-allyl-2-benzyl-2-(4,4,5,5-tetramethyl-1,3,2-dioxaborolan-2-yl)cyclopropane-1-carboxylate (1c)**

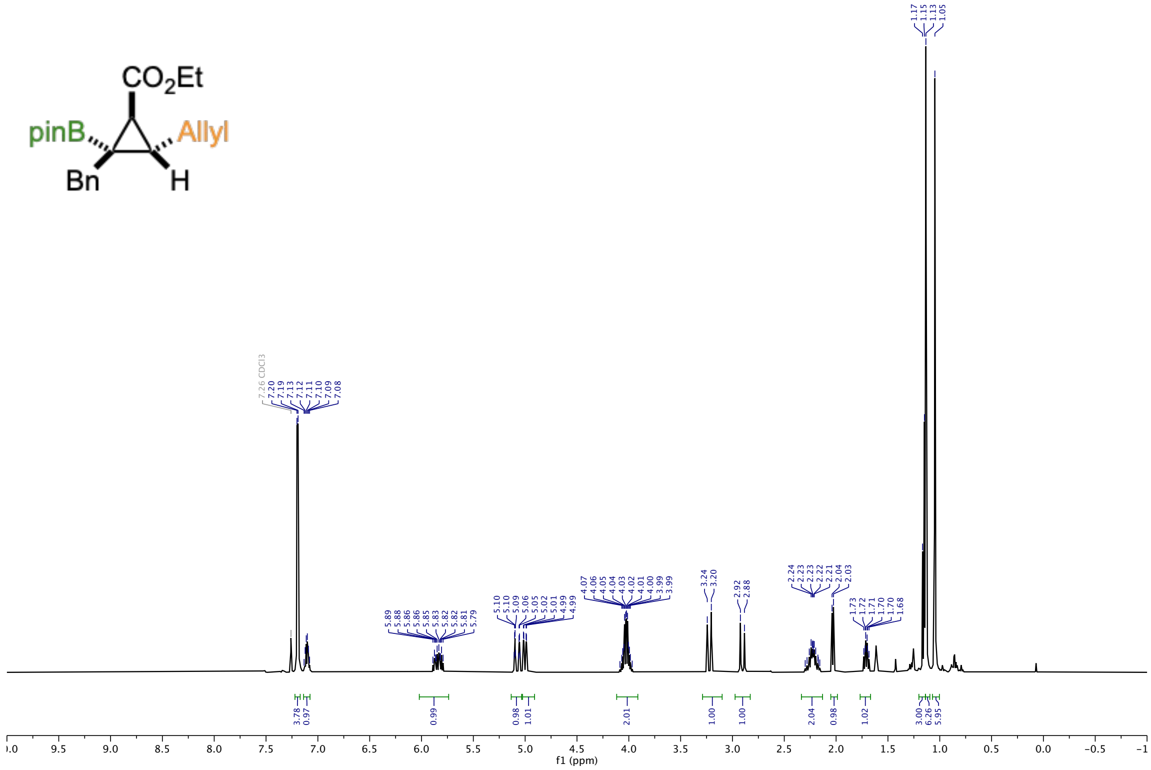

^{1}H NMR spectrum (400 MHz, $CDCl_3$)

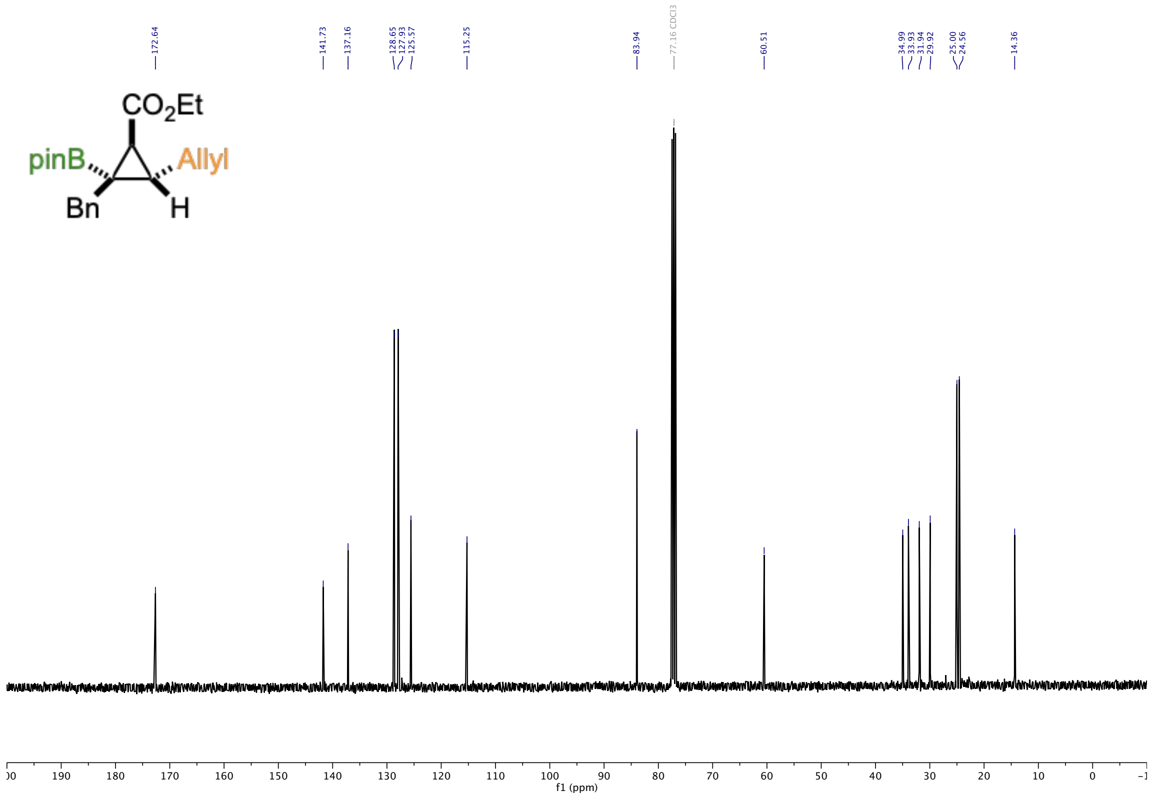

^{13}C NMR spectrum (101 MHz, $CDCl_3$)

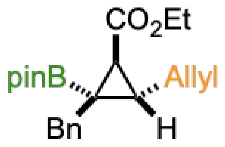

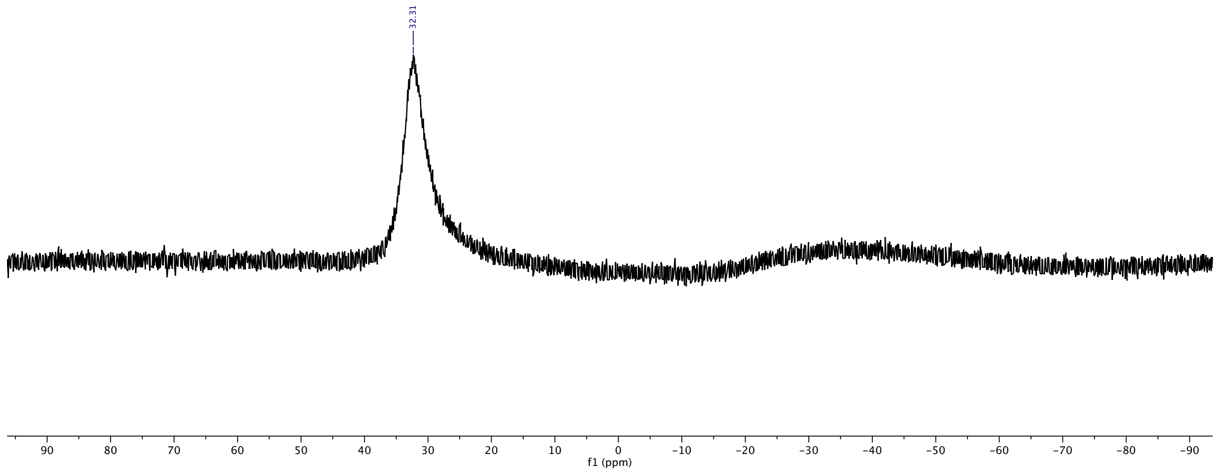

^{11}B NMR spectrum (128 MHz, $CDCl_3$)

Ethyl (1*S,2*R**,3*R**)-2,3-diethyl-2-(4,4,5,5-tetramethyl-1,3,2-dioxaborolan-2-yl)cyclopropane-1-carboxylate (1d)**

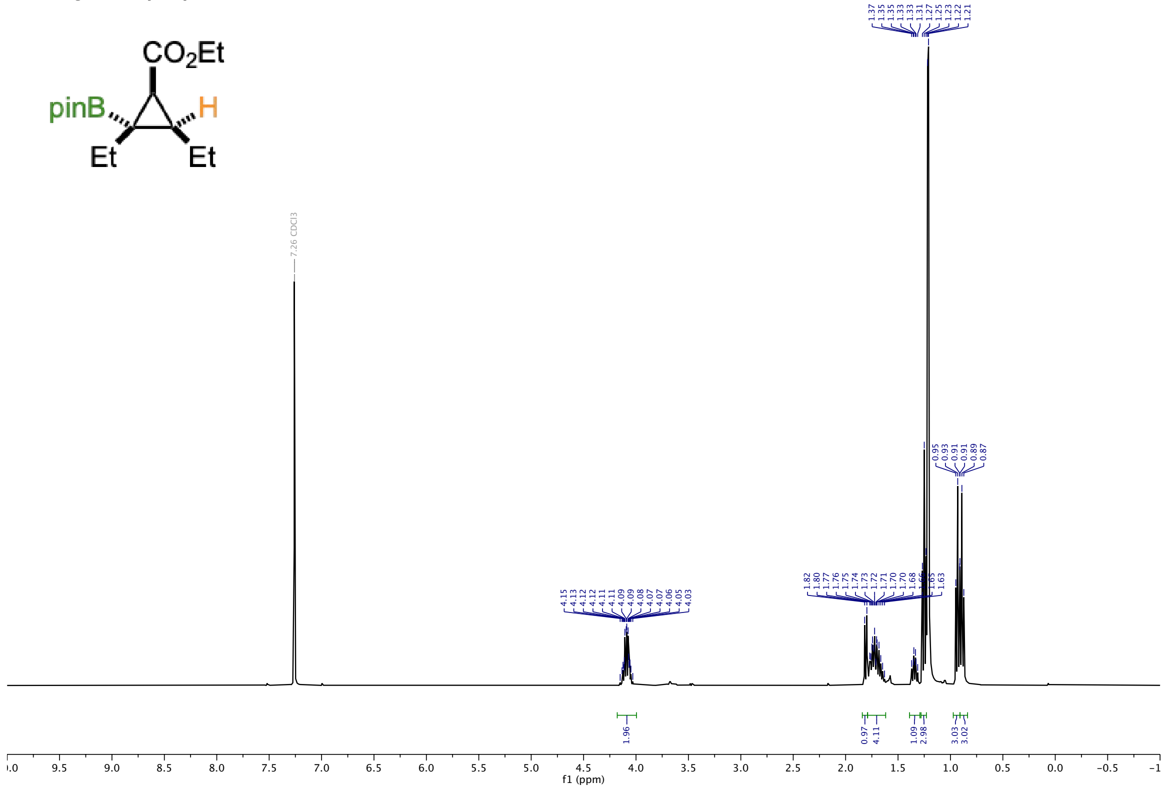

^{1}H NMR spectrum (400 MHz, $CDCl_3$)

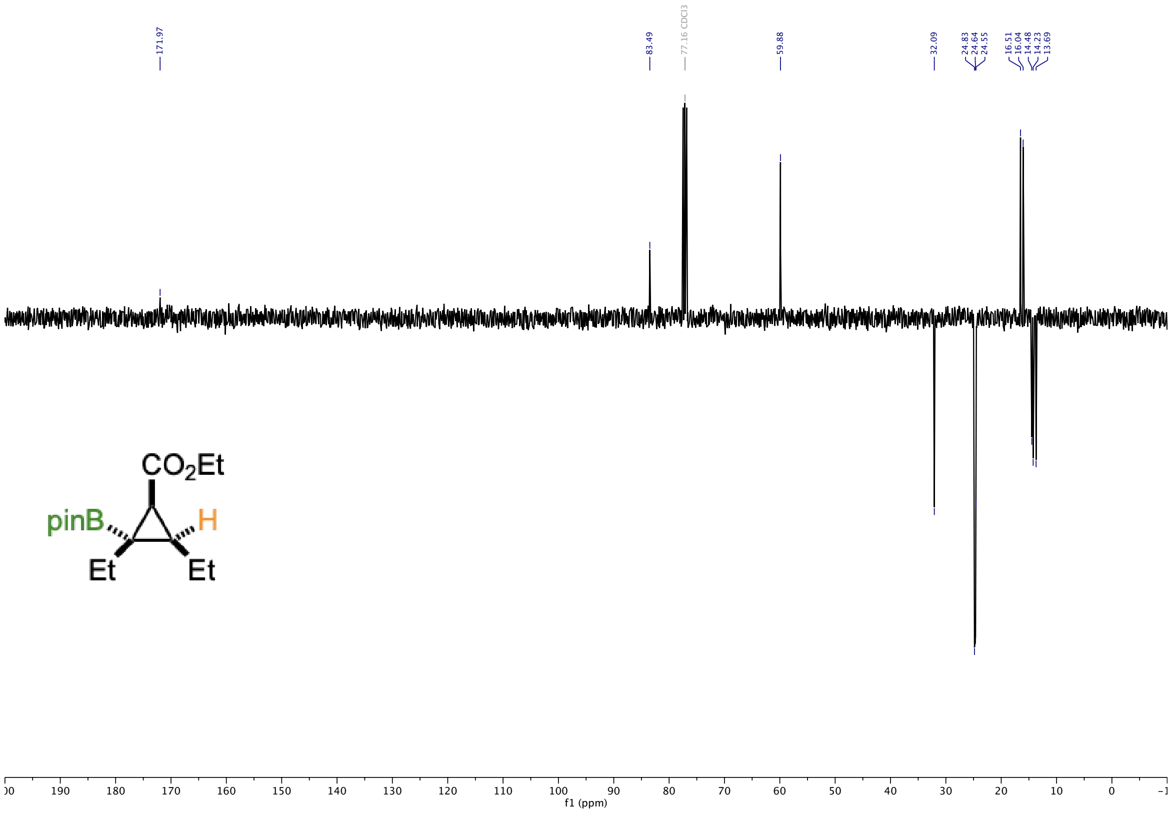

^{13}C NMR (APT) spectrum (101 MHz, $CDCl_3$)

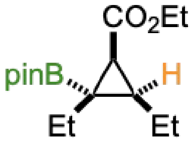

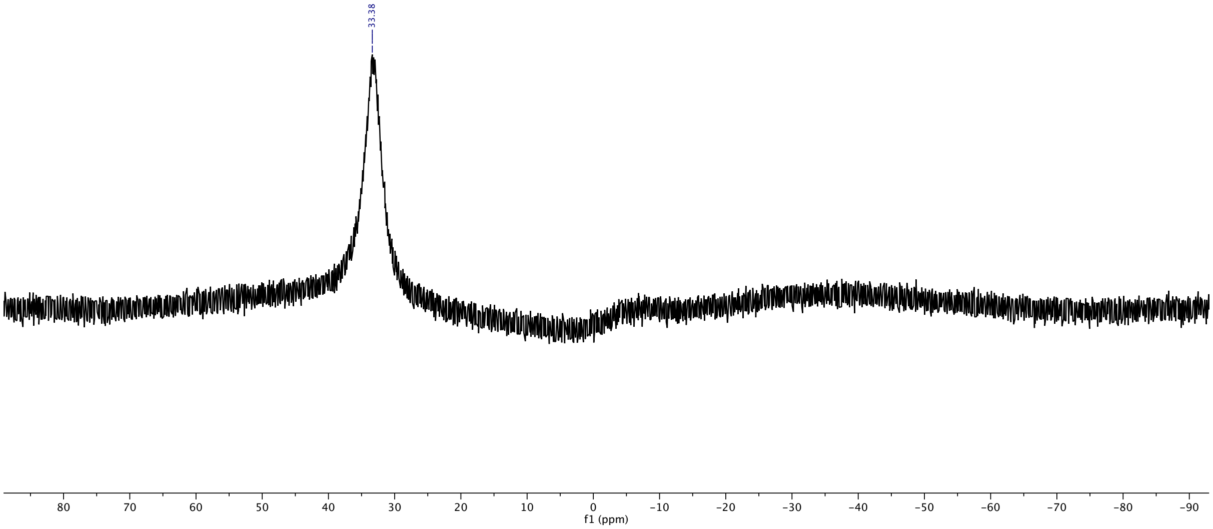

^{11}**B NMR** spectrum (128 MHz, $CDCl_3$)

((1*S,2*R**,3*S**)-3-Allyl-2-benzyl-2-(4,4,5,5-tetramethyl-1,3,2-dioxaborolan-2-yl)cyclopropyl)methyl diethyl phosphate (2c)**

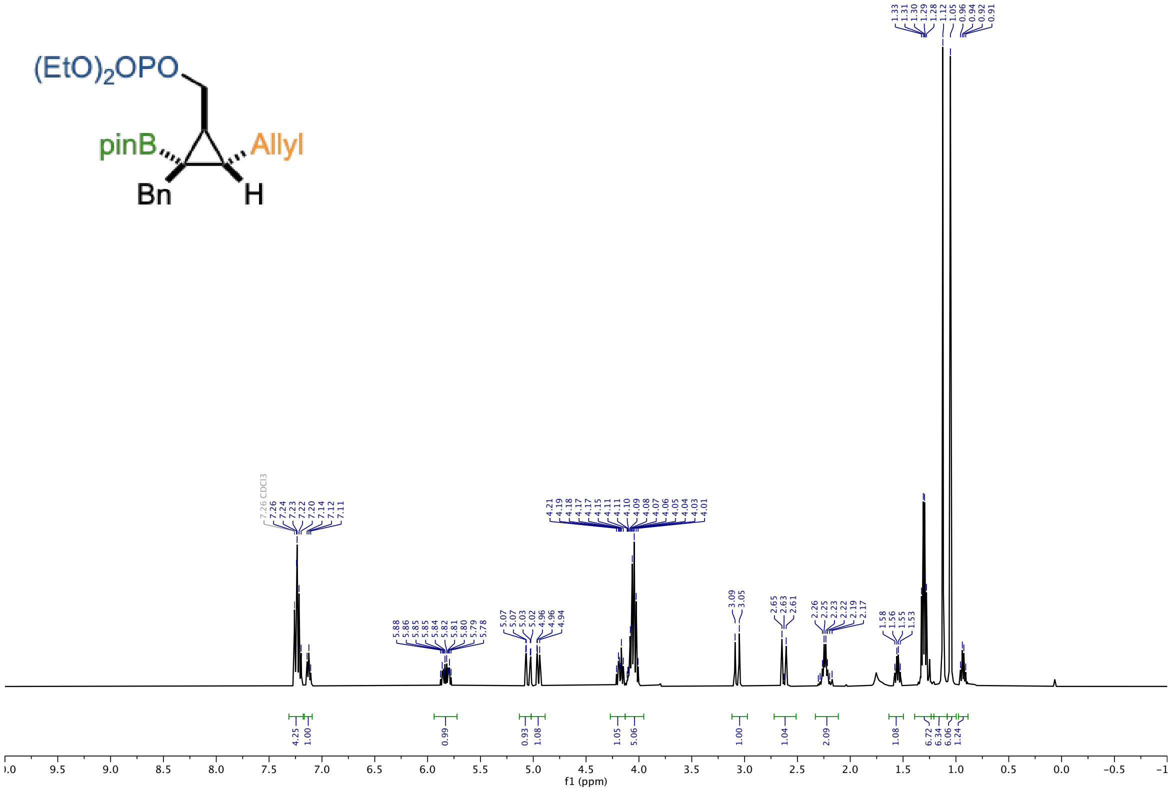

¹H NMR spectrum (400 MHz, CDCl₃)

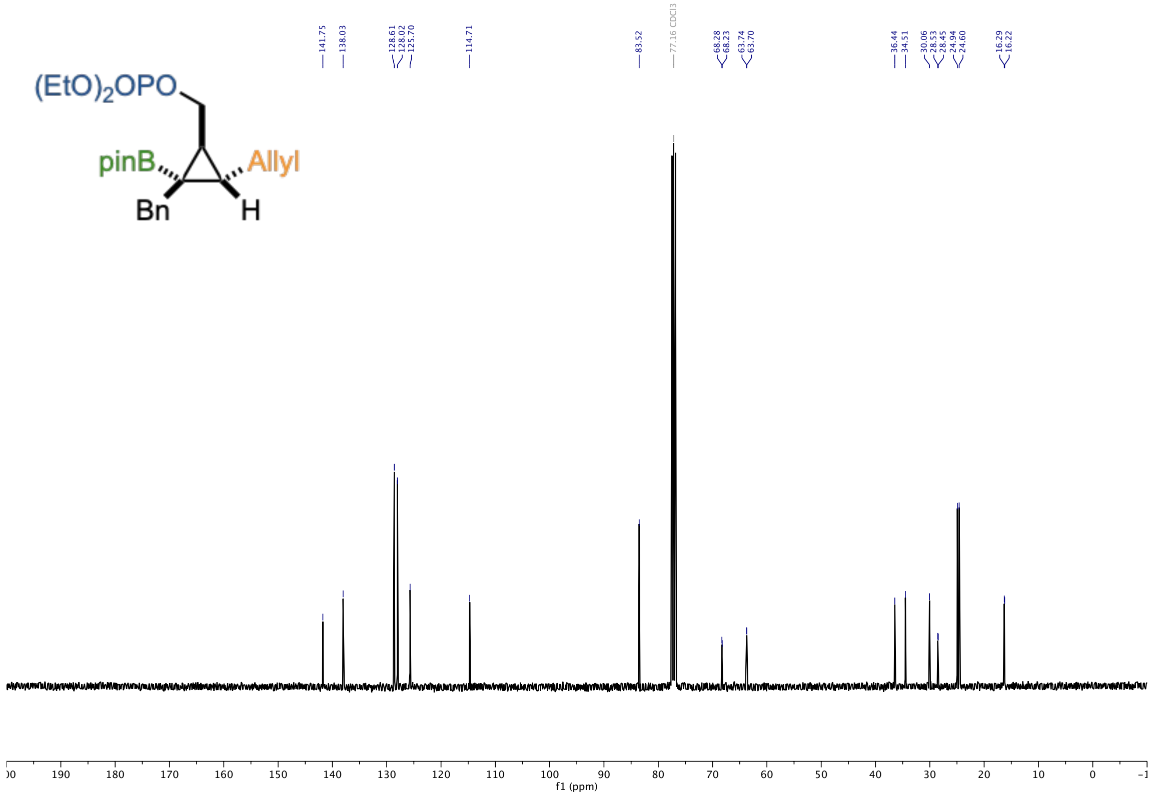

¹³C NMR spectrum (101 MHz, CDCl₃)

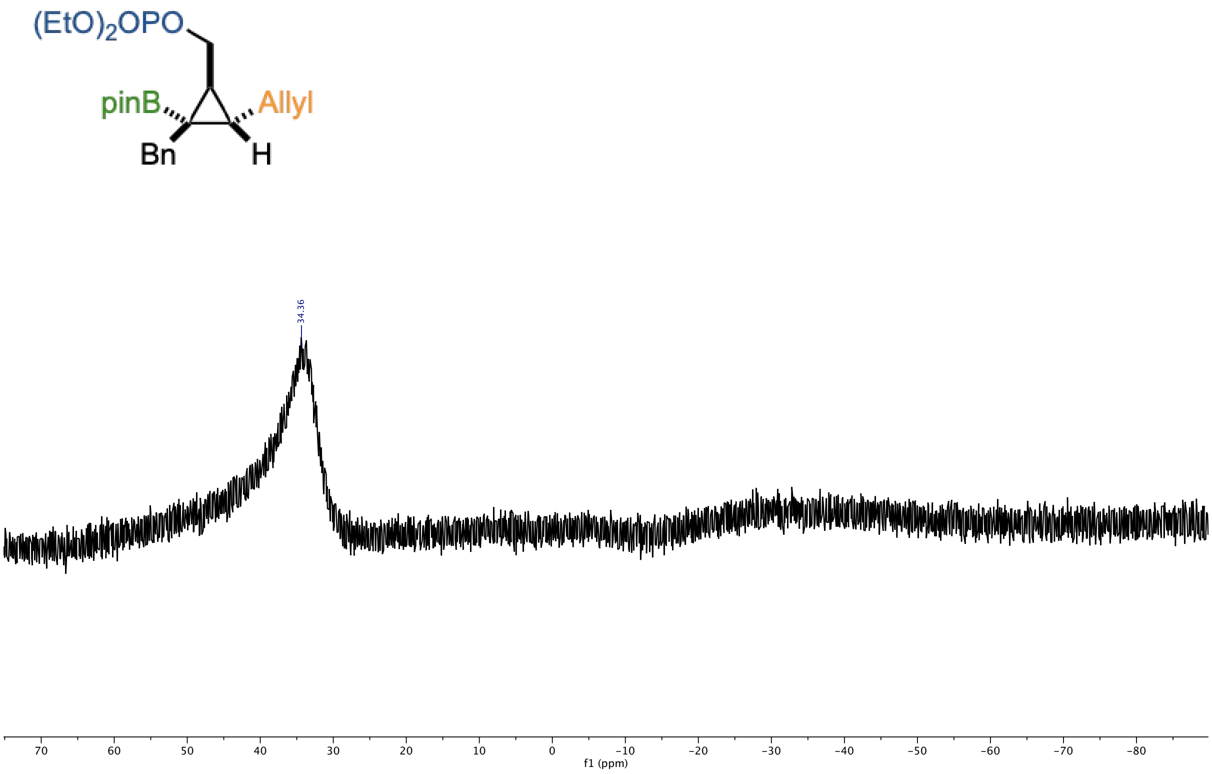

^{11}B NMR spectrum (128 MHz, $CDCl_3$)

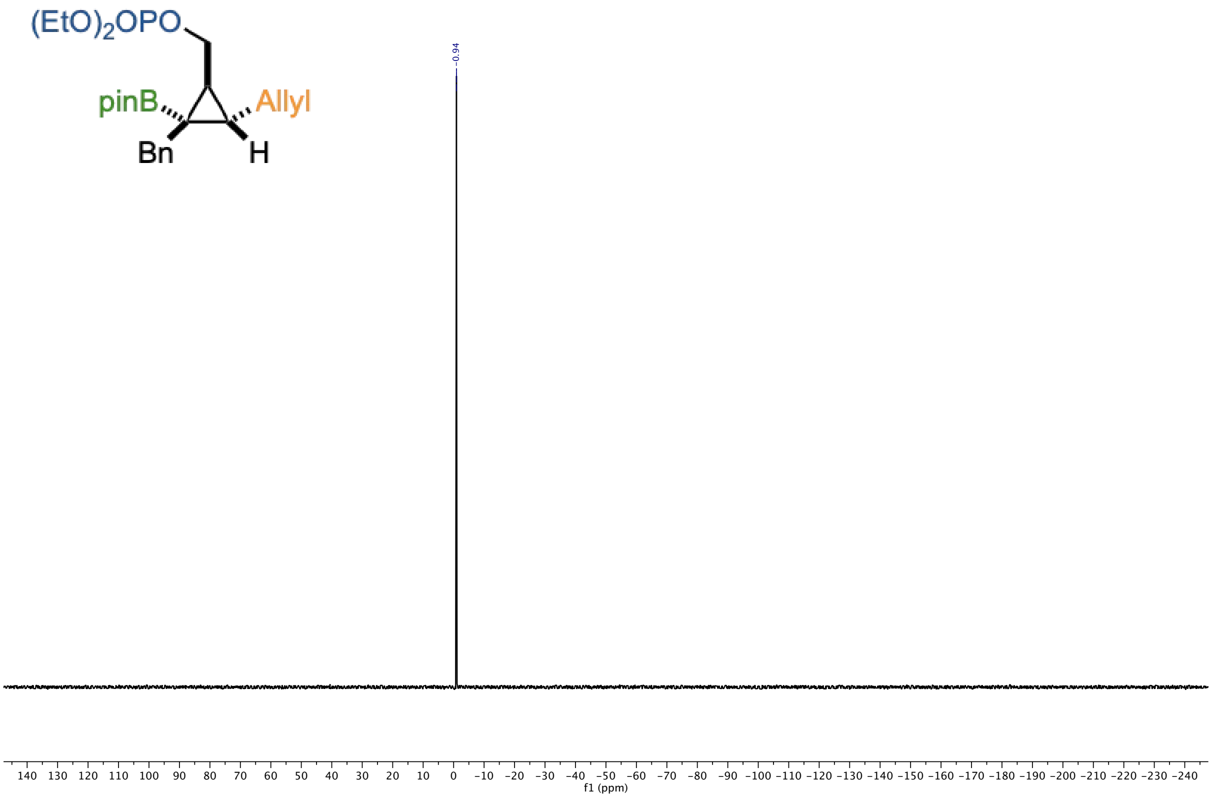

^{31}P NMR spectrum (162 MHz, $CDCl_3$)

((1*S,2*R**,3*R**)-2,3-Diethyl-2-(4,4,5,5-tetramethyl-1,3,2-dioxaborolan-2-yl)cyclopropyl)methyl diethyl phosphate (2d)**

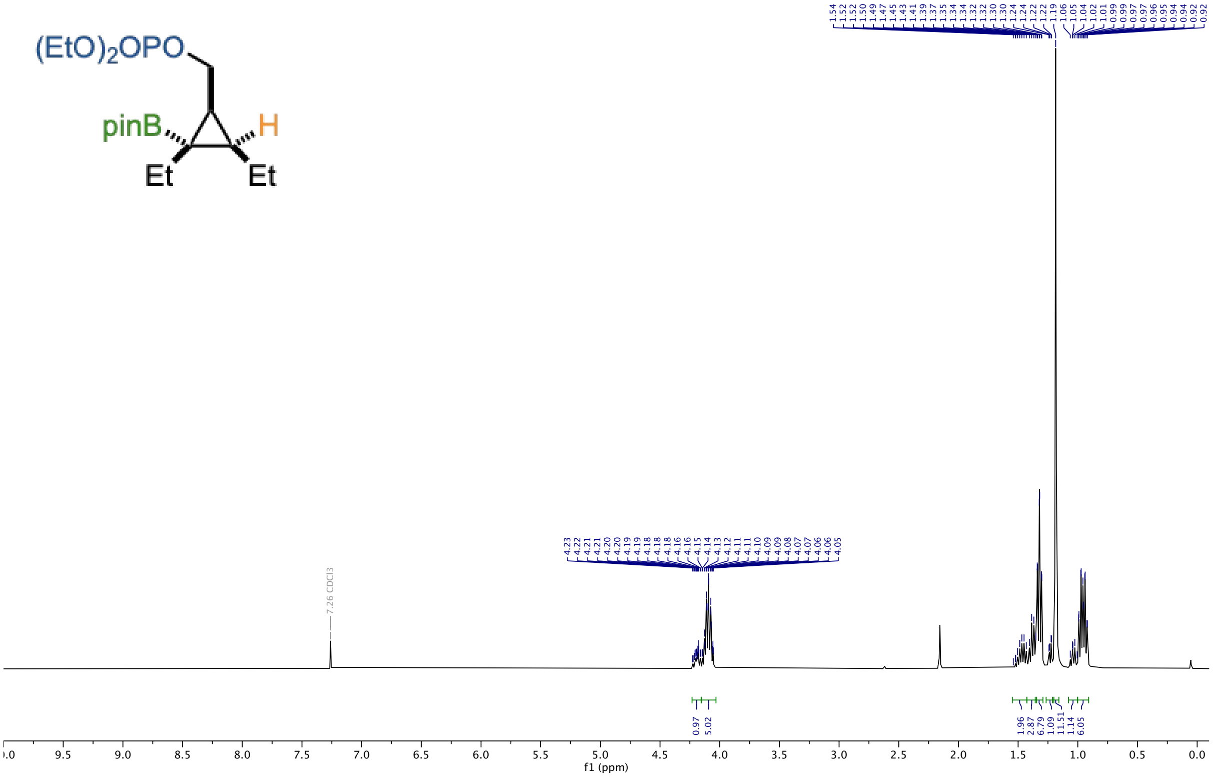

^{1}H NMR spectrum (400 MHz, $CDCl_3$)

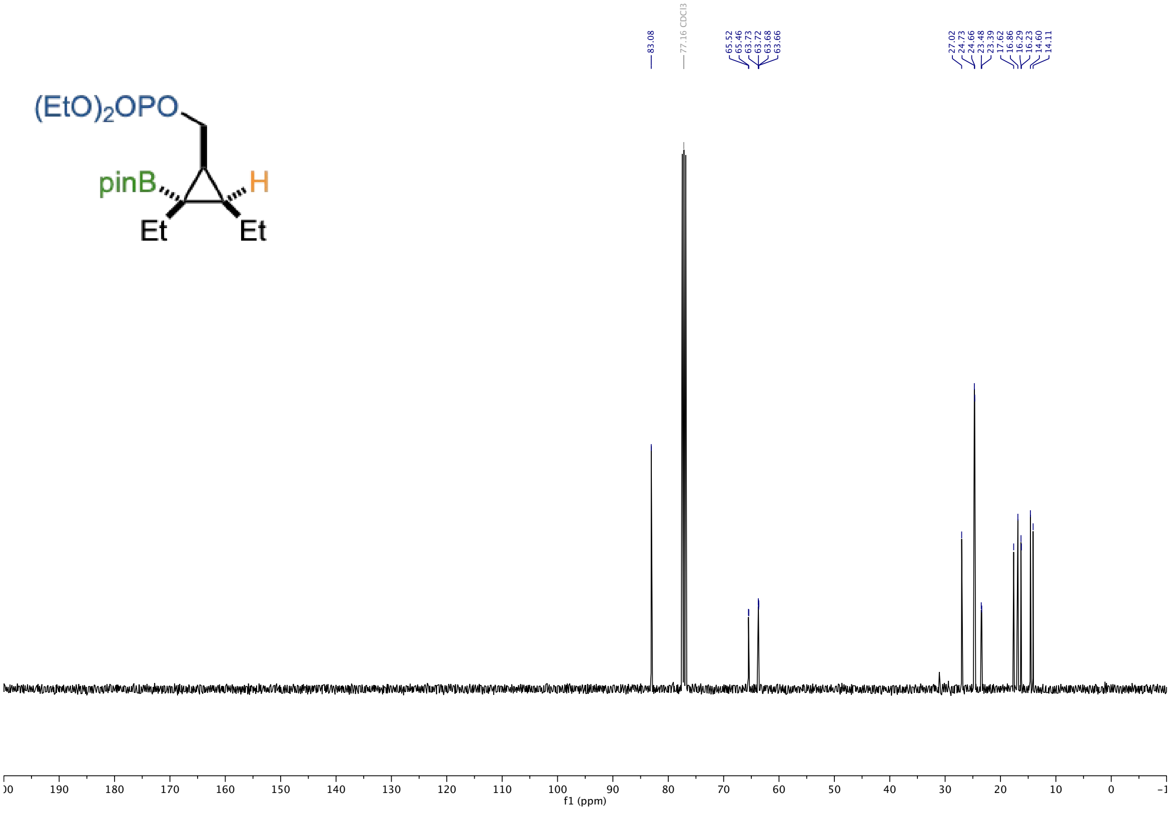

^{13}C NMR spectrum (101 MHz, $CDCl_3$)

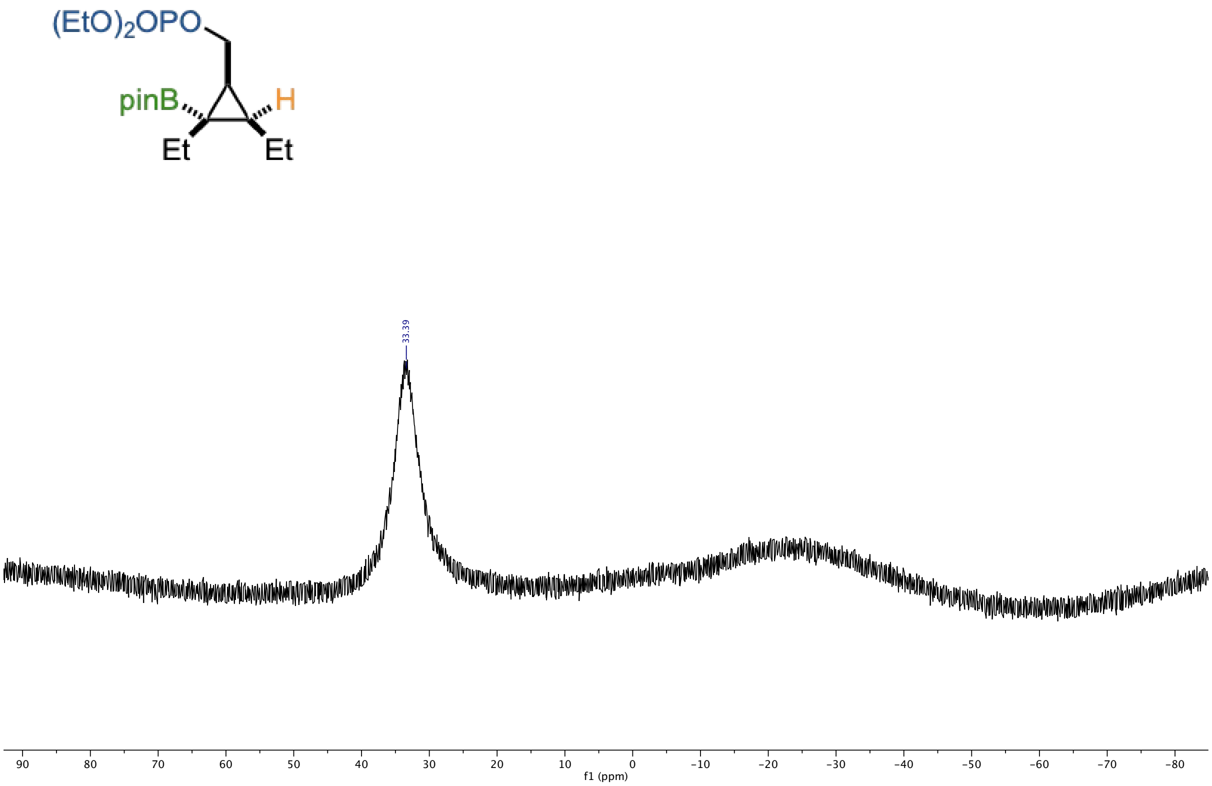

^{11}B NMR spectrum (128 MHz, $CDCl_3$)

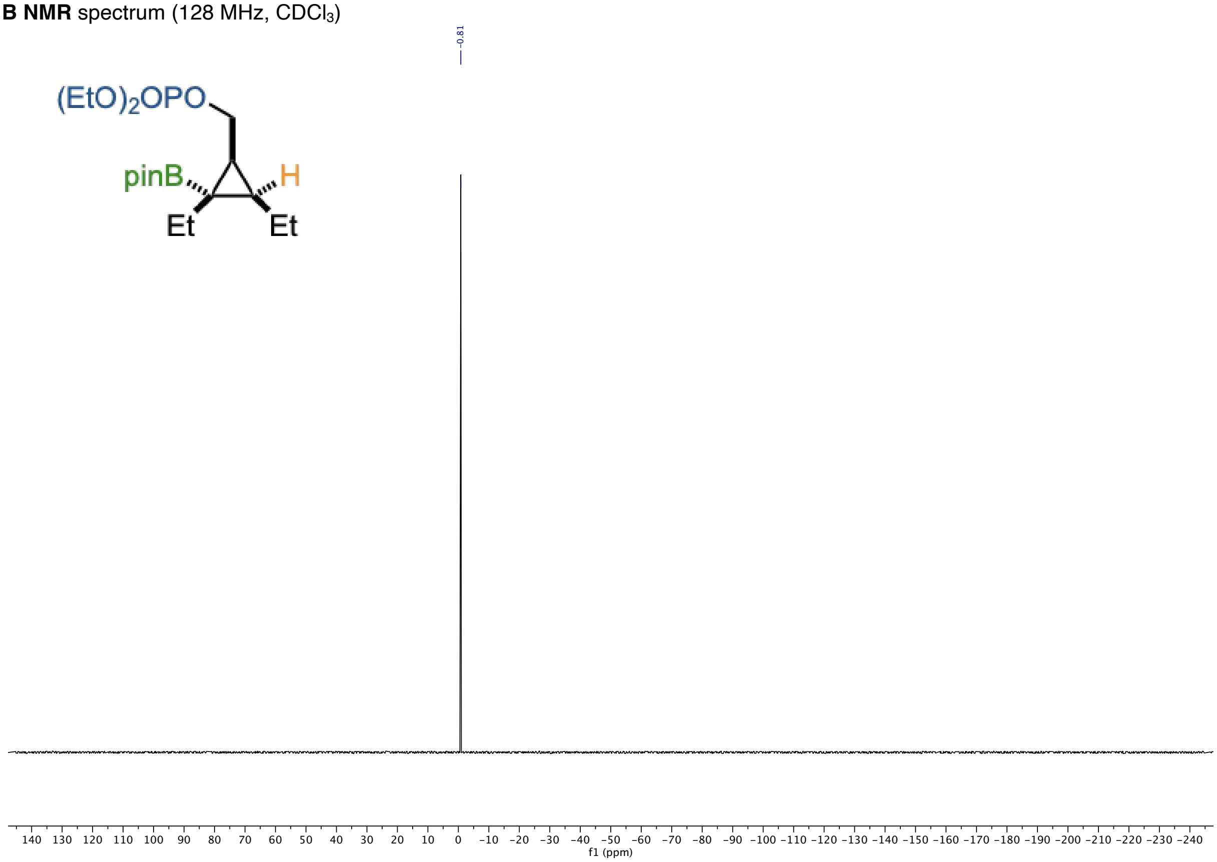

^{31}P NMR spectrum (162 MHz, $CDCl_3$)

((1*S**,2*R**)-2-Butyl-2-(4,4,5,5-tetramethyl-1,3,2-dioxaborolan-2-yl)cyclopropyl)methanol (3a)

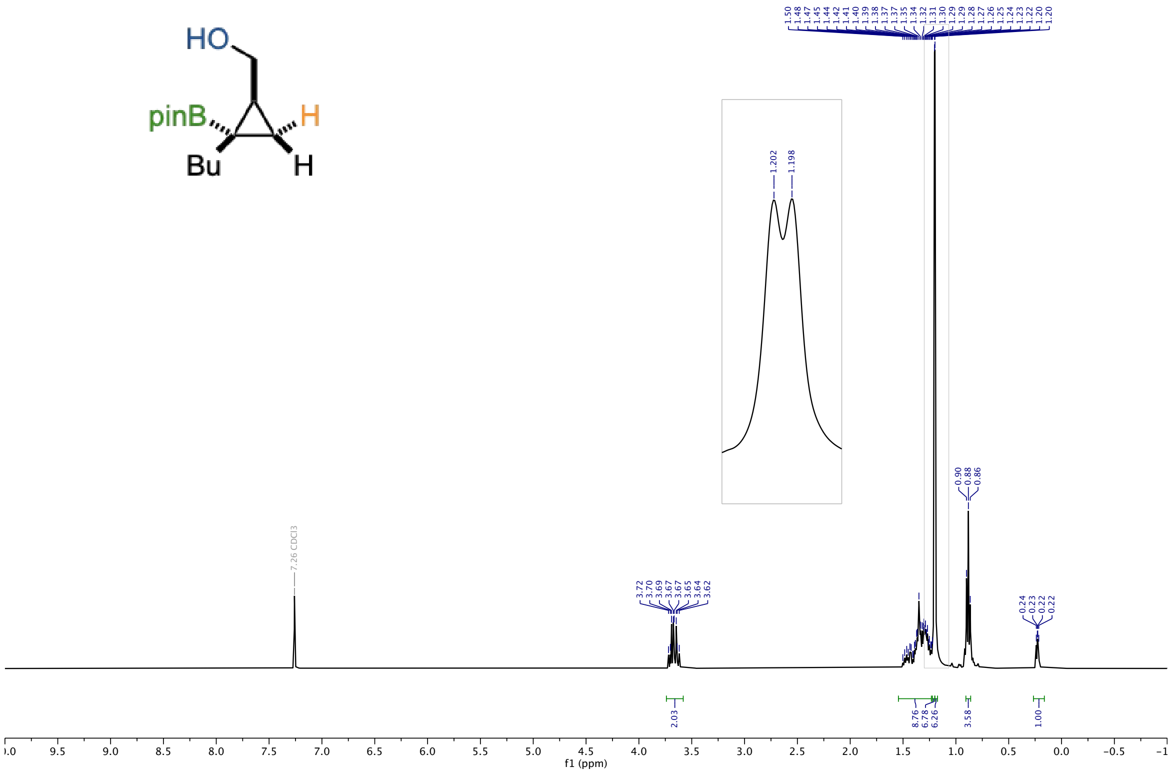

^{1}H NMR spectrum (400 MHz, $CDCl_3$)

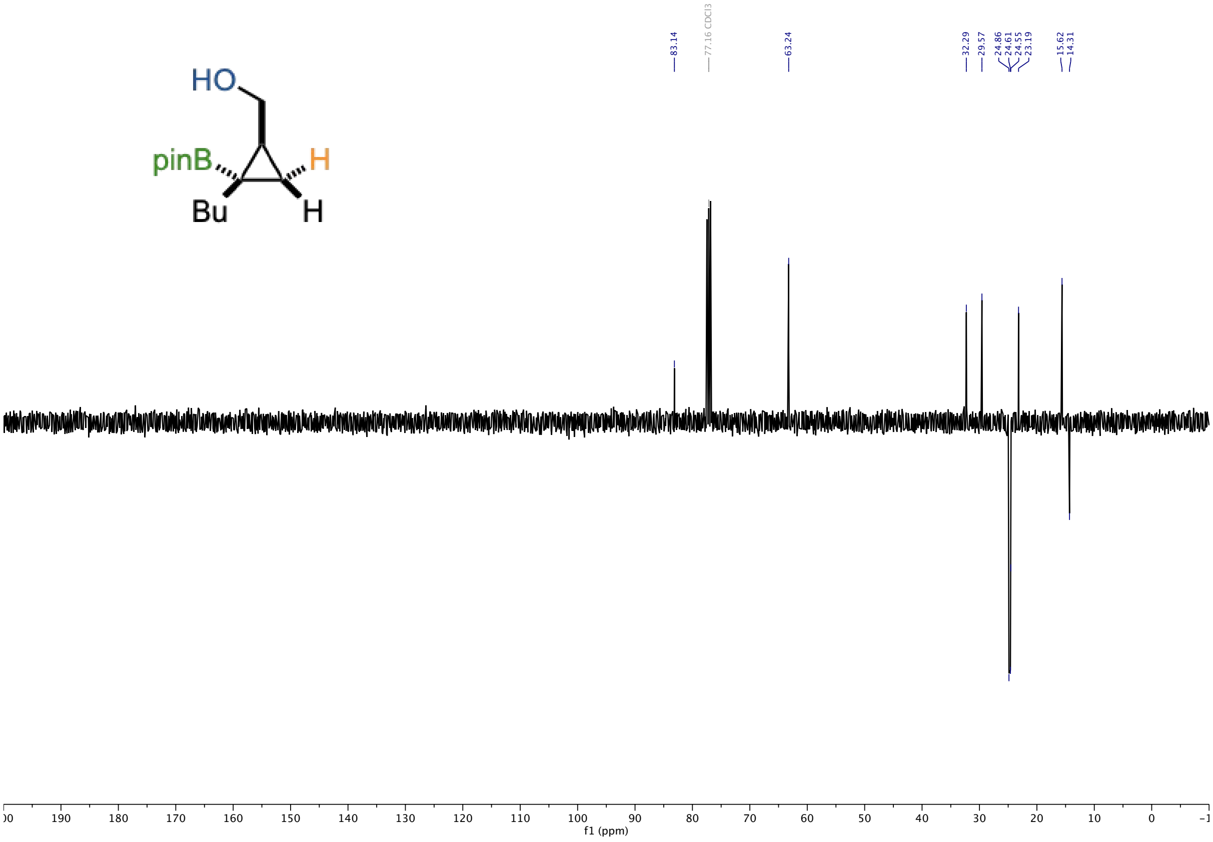

^{13}C NMR (APT) spectrum (101 MHz, $CDCl_3$)

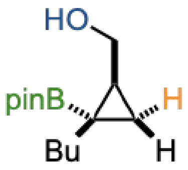

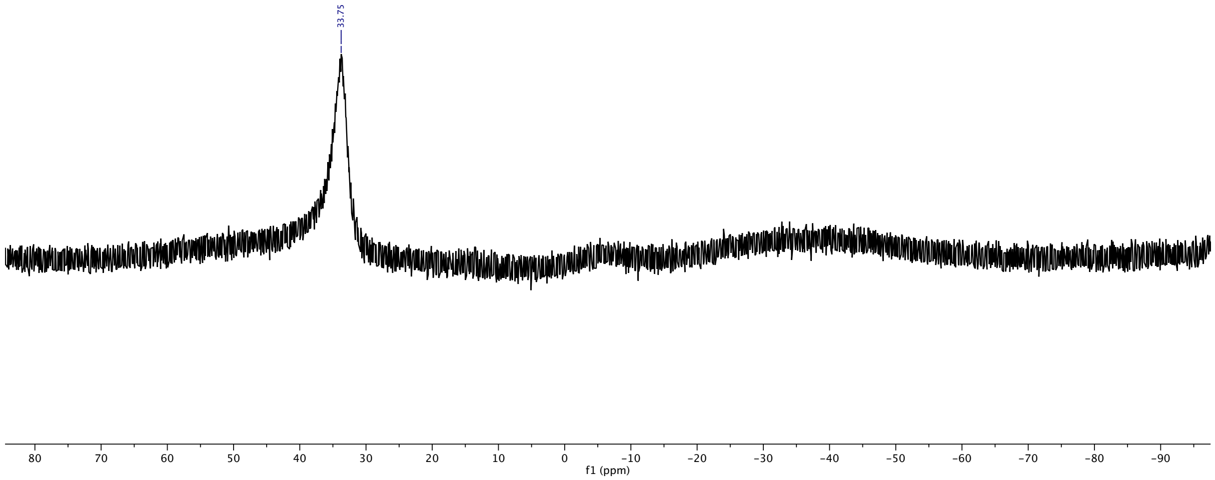

^{11}B NMR spectrum (128 MHz, $CDCl_3$)

((1*S**,2*R**)-2-(3-Chloropropyl)-2-(4,4,5,5-tetramethyl-1,3,2-dioxaborolan-2-yl)cyclopropyl)methanol (3b)

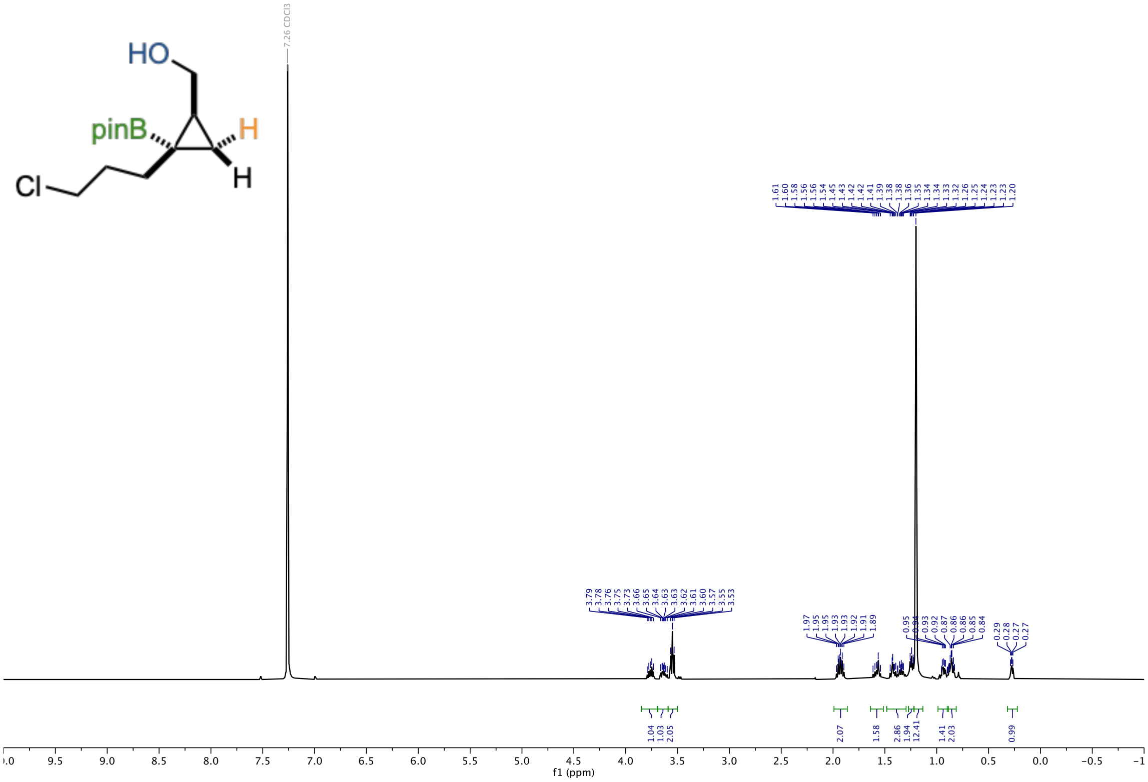

^{1}H NMR spectrum (400 MHz, $CDCl_3$)

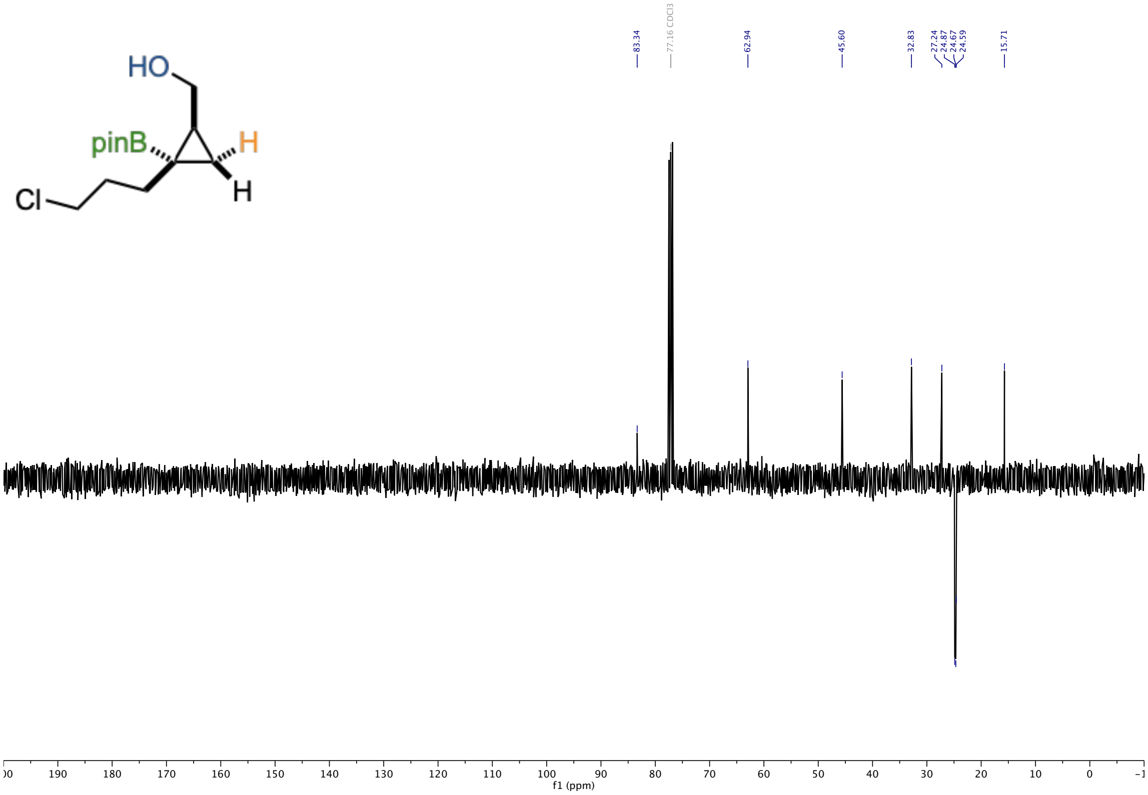

^{13}C NMR (APT) spectrum (101 MHz, $CDCl_3$)

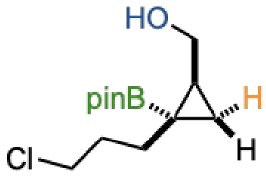

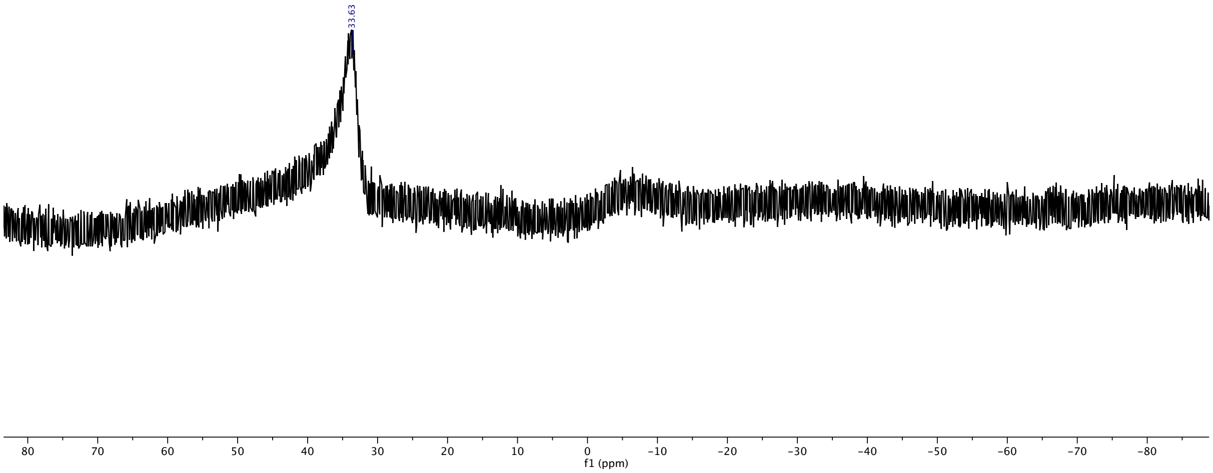

^{11}B NMR spectrum (128 MHz, $CDCl_3$)

((1*S,2*R**,3*S**)-3-Allyl-2-butyl-2-(4,4,5,5-tetramethyl-1,3,2-dioxaborolan-2-yl)cyclopropyl)methanol (3c)**

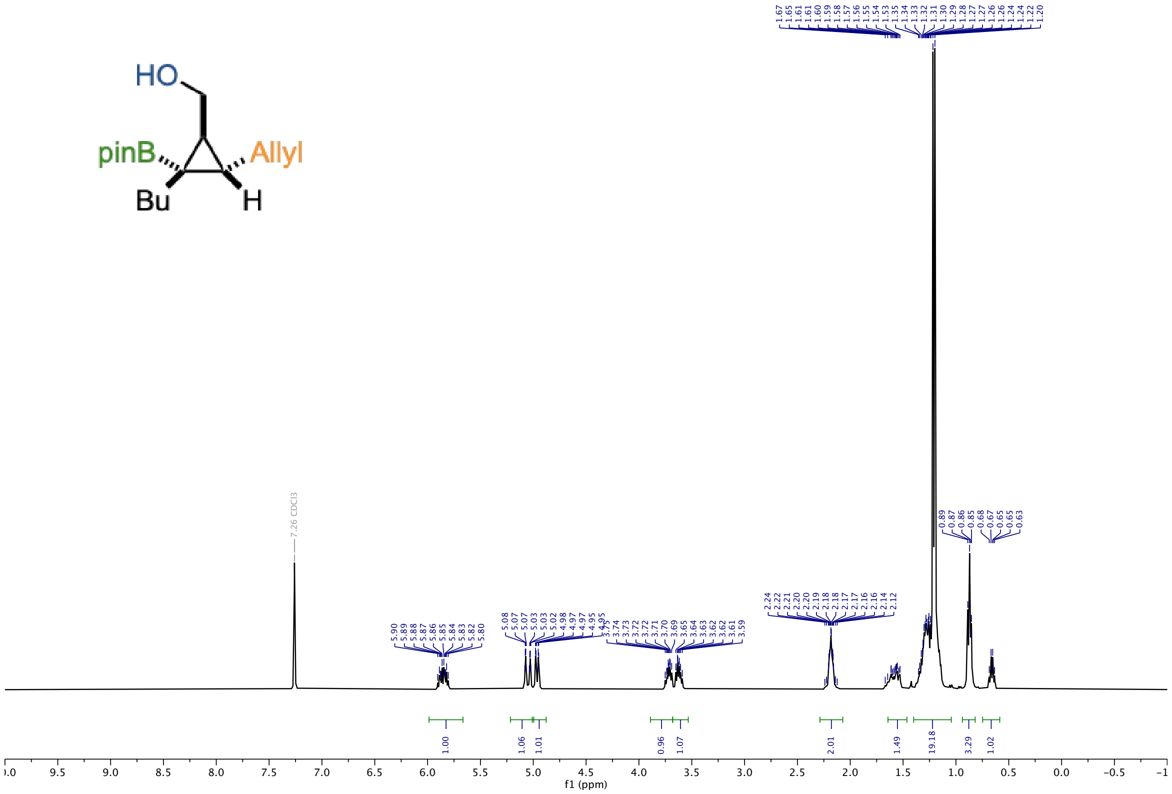

^{1}H NMR spectrum (400 MHz, $CDCl_3$)

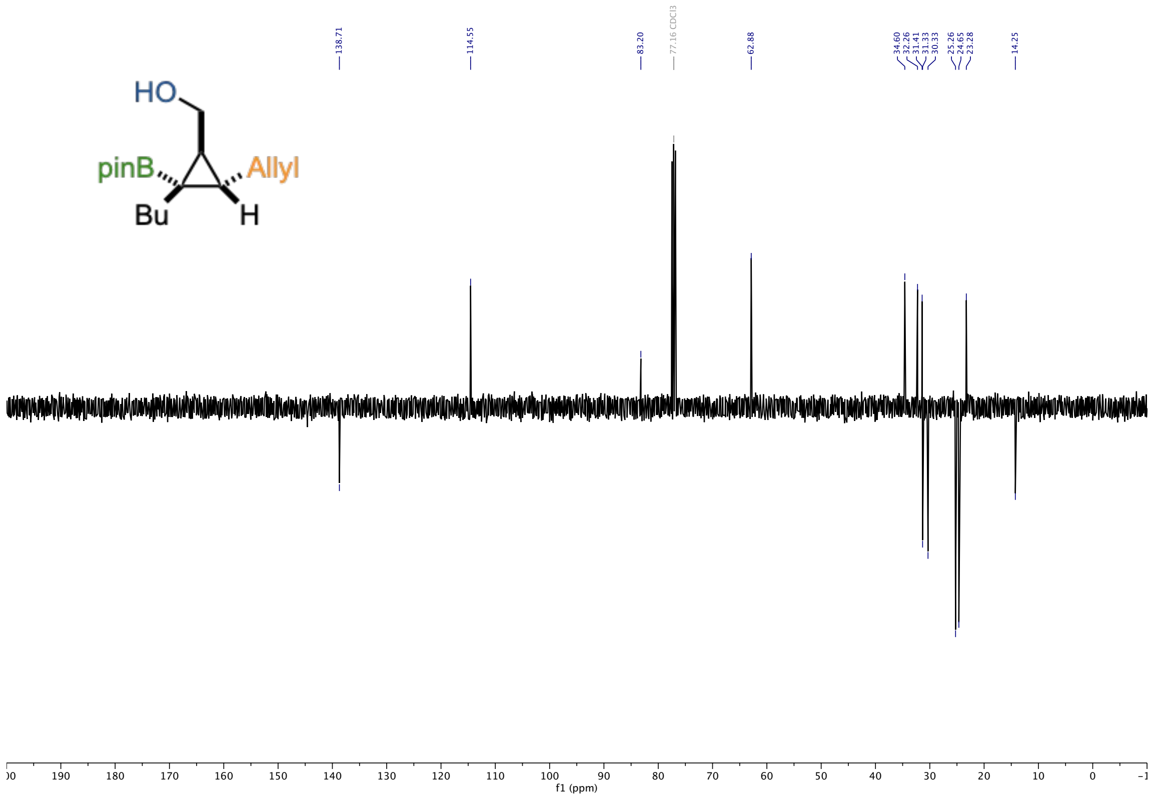

^{13}C NMR (APT) spectrum (101 MHz, $CDCl_3$)

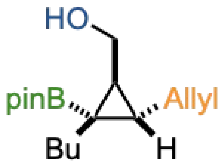

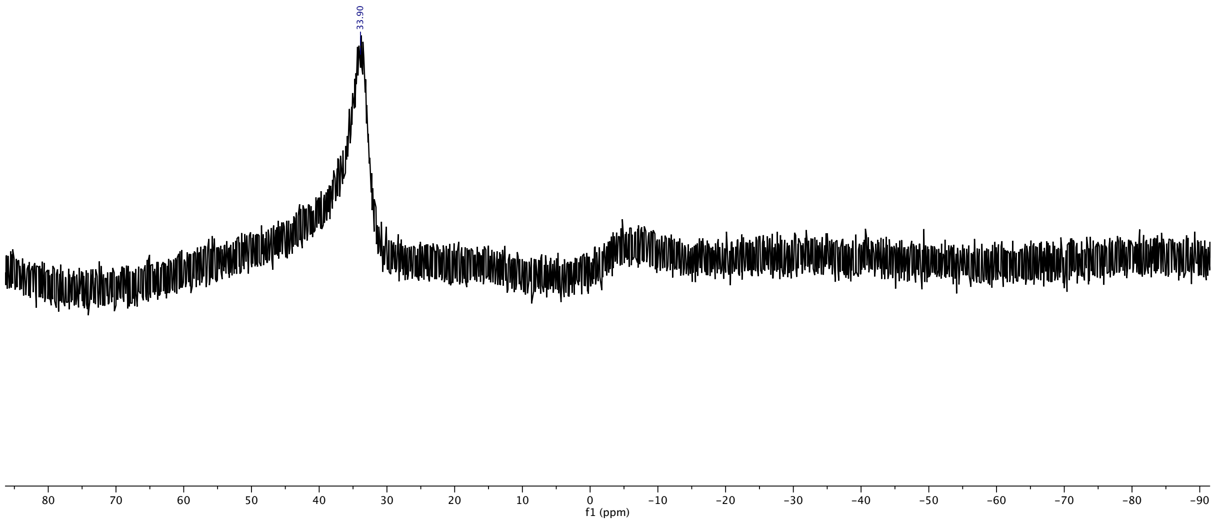

^{11}B NMR spectrum (128 MHz, $CDCl_3$)

((1*S,2*R**,3*R**)-2,3-Dimethyl-2-(4,4,5,5-tetramethyl-1,3,2-dioxaborolan-2-yl)cyclopropyl)methanol (3d)**

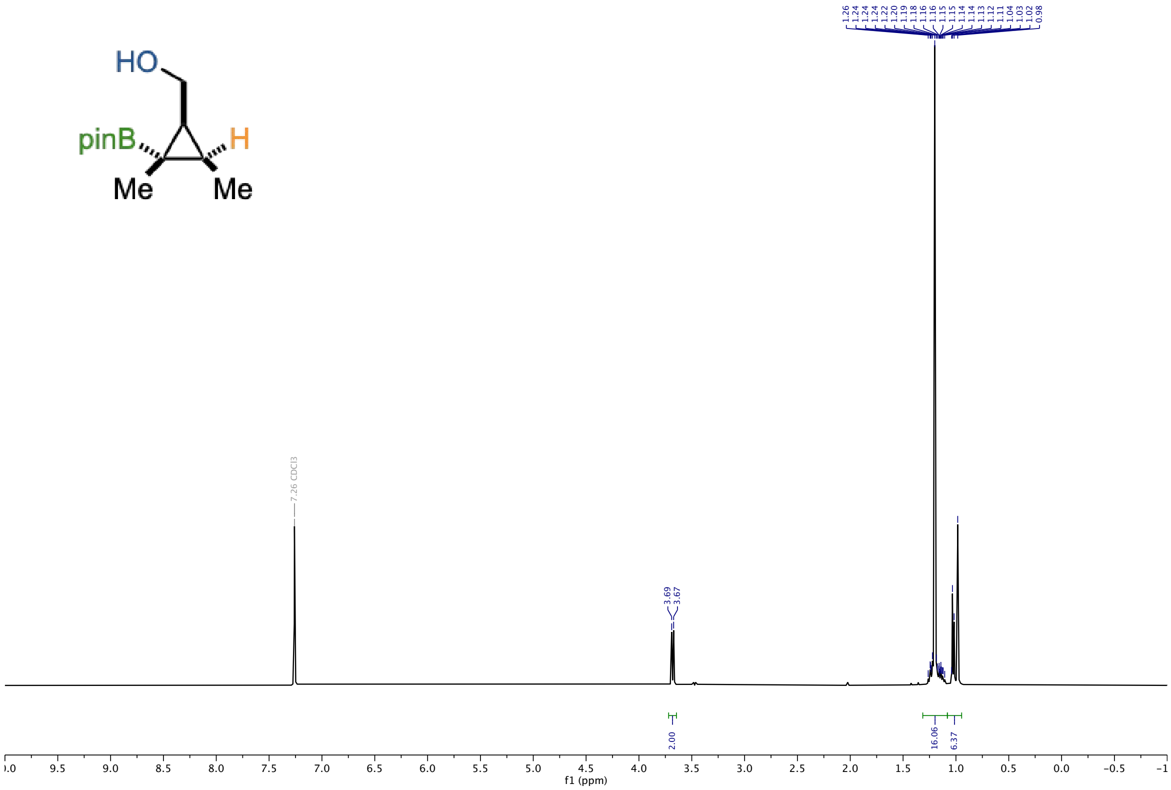

^{1}**H NMR** spectrum (400 MHz, $CDCl_3$)

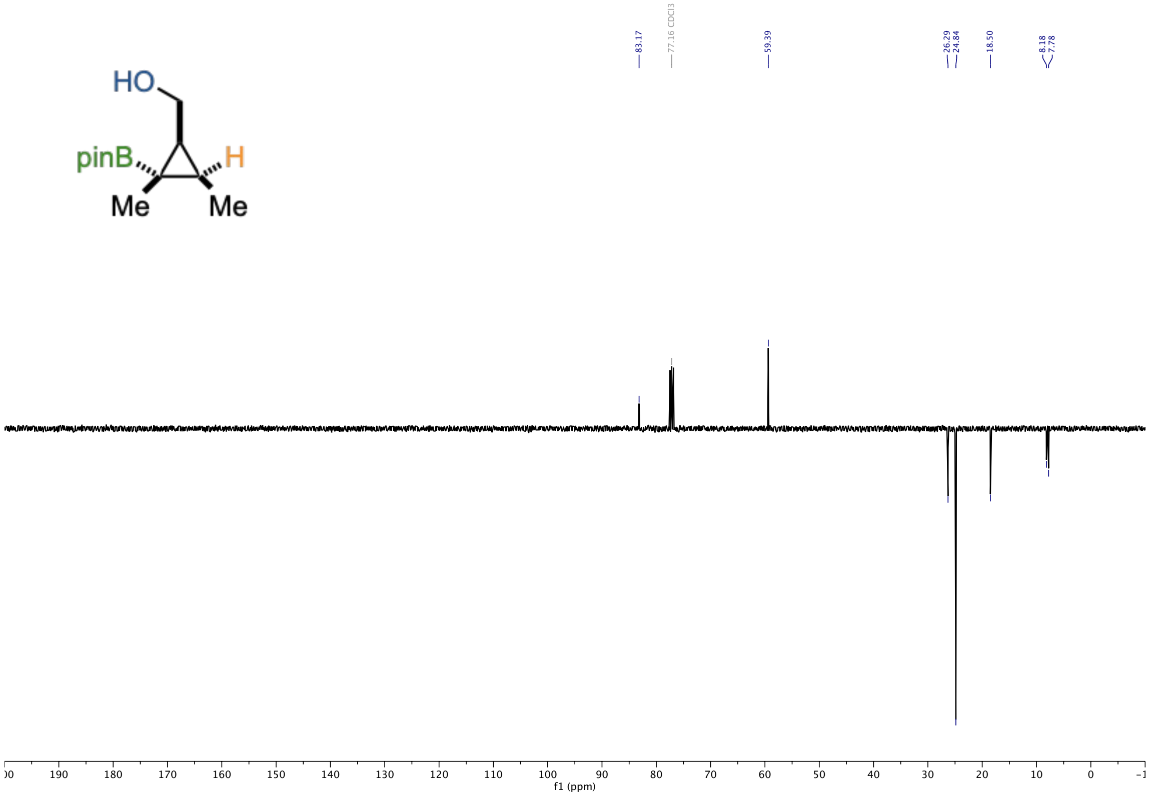

^{13}**C NMR** (APT) spectrum (101 MHz, $CDCl_3$)

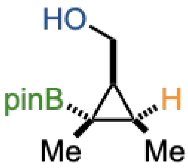

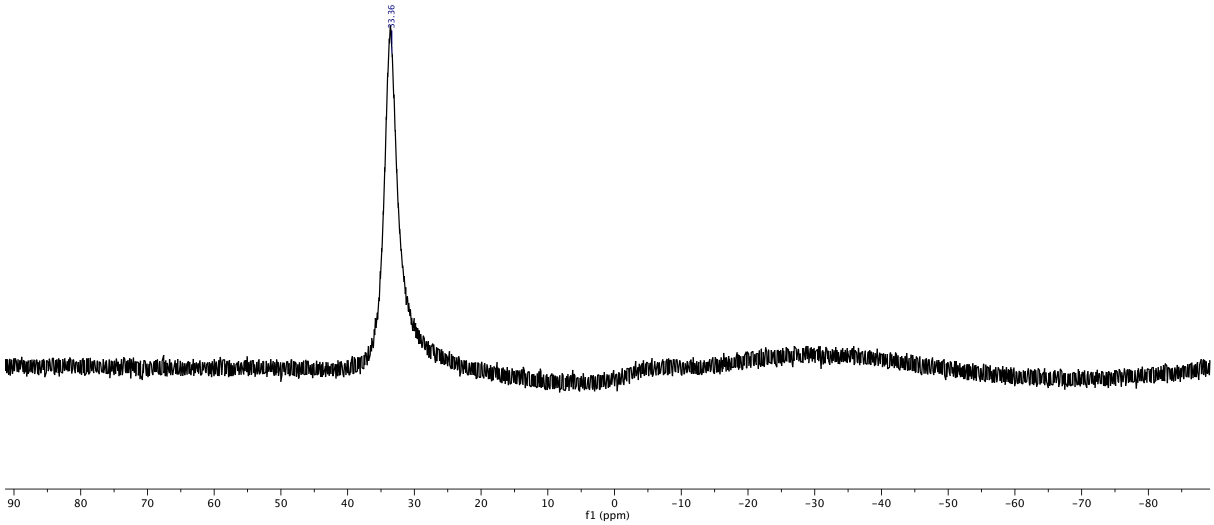

^{11}B NMR spectrum (128 MHz, $CDCl_3$)

((1*S**,2*R**,3*R**)-2-Ethyl-3-phenyl-2-(4,4,5,5-tetramethyl-1,3,2-dioxaborolan-2-yl)cyclopropyl)methanol (3e)

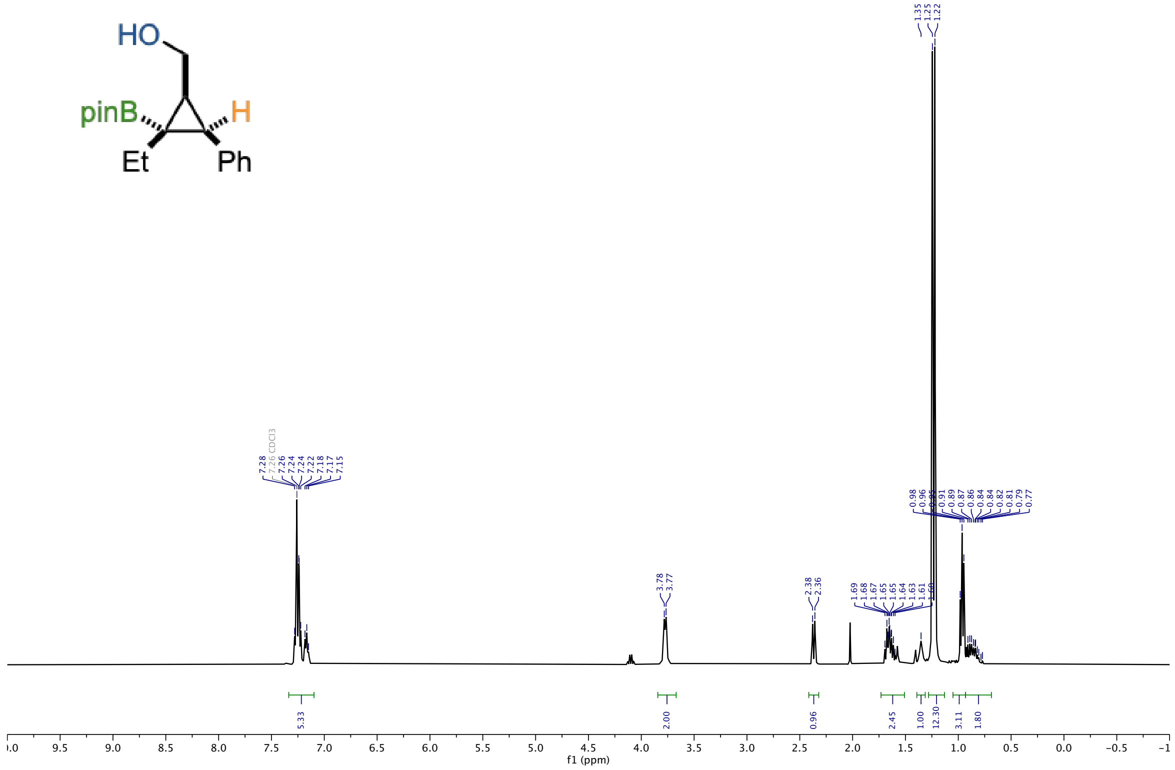

^{1}H NMR spectrum (400 MHz, $CDCl_3$)

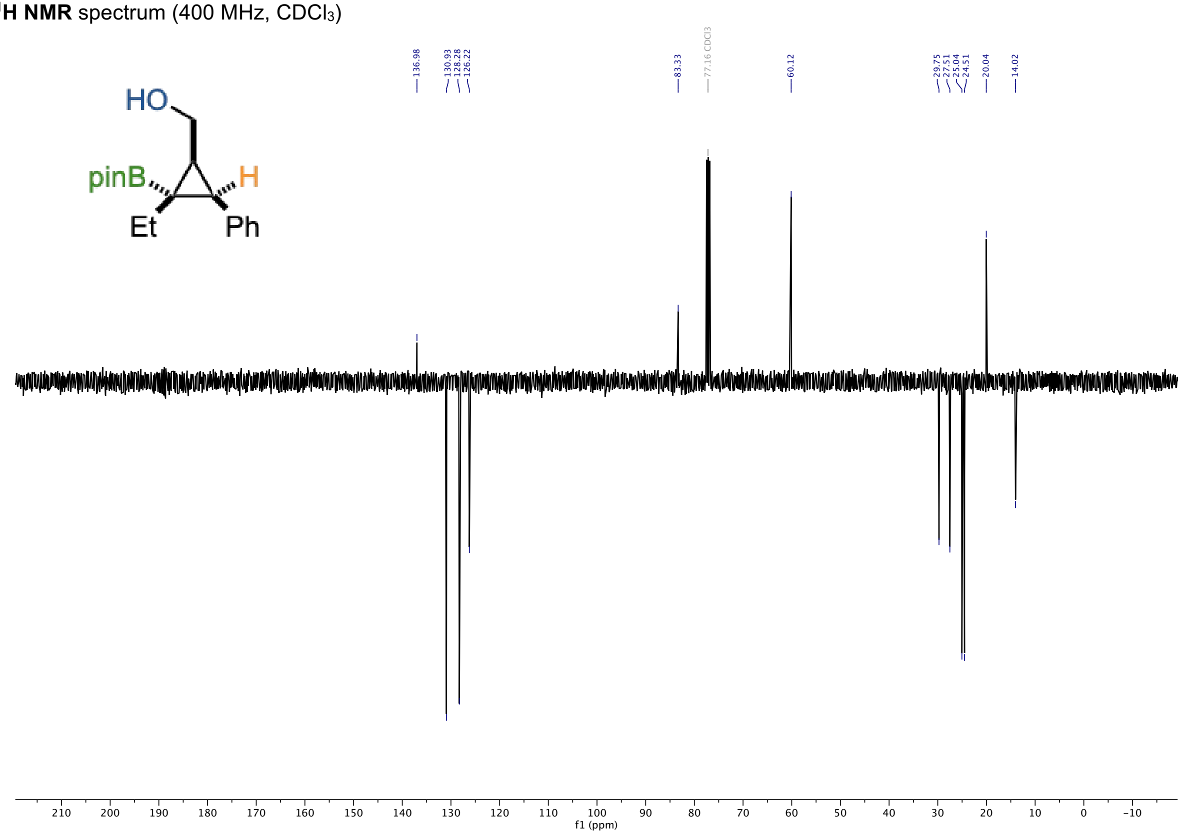

^{13}C (APT) NMR spectrum

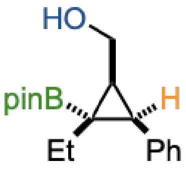

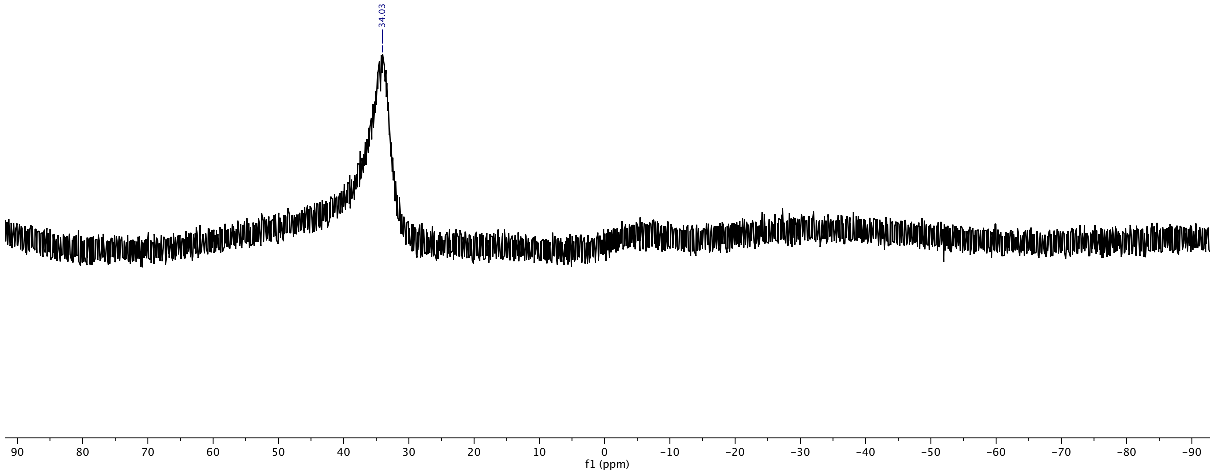

^{11}B NMR spectrum (128 MHz, $CDCl_3$)

((1*S**,2*R**,3*R**)-2,3-diethyl-2-(4,4,5,5-tetramethyl-1,3,2-dioxaborolan-2-yl)cyclopropyl)methanol (3f)

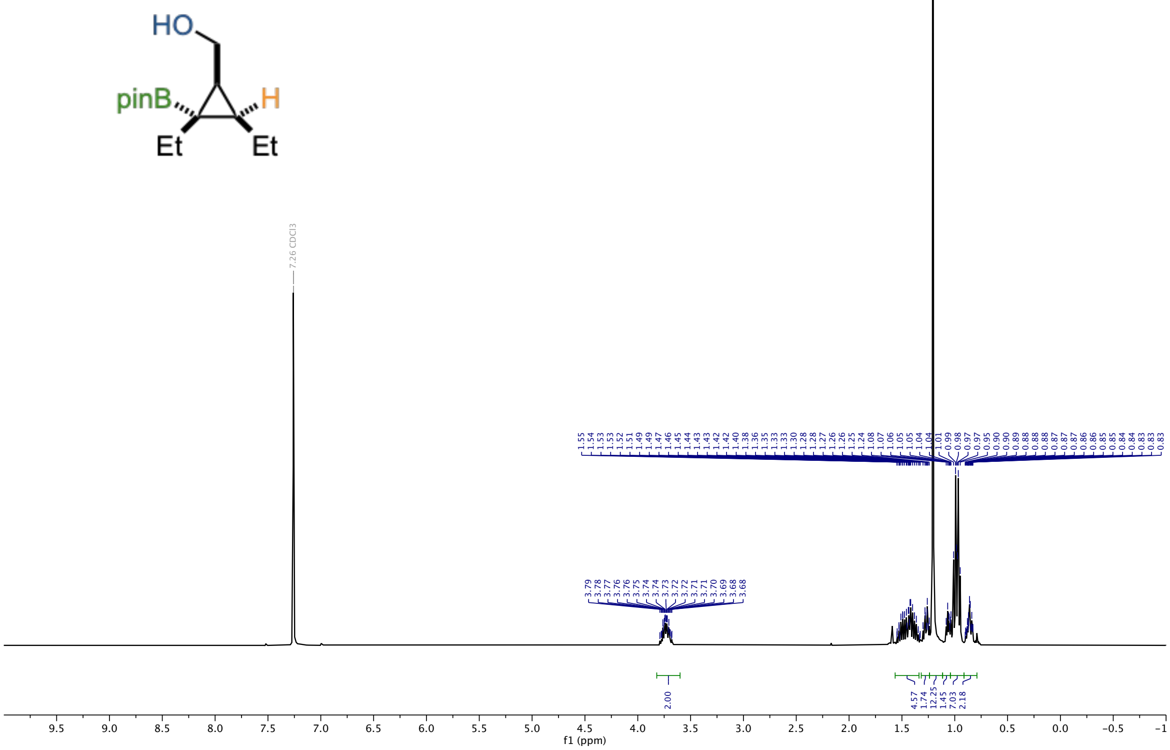

^{1}H NMR spectrum (400 MHz, $CDCl_3$)

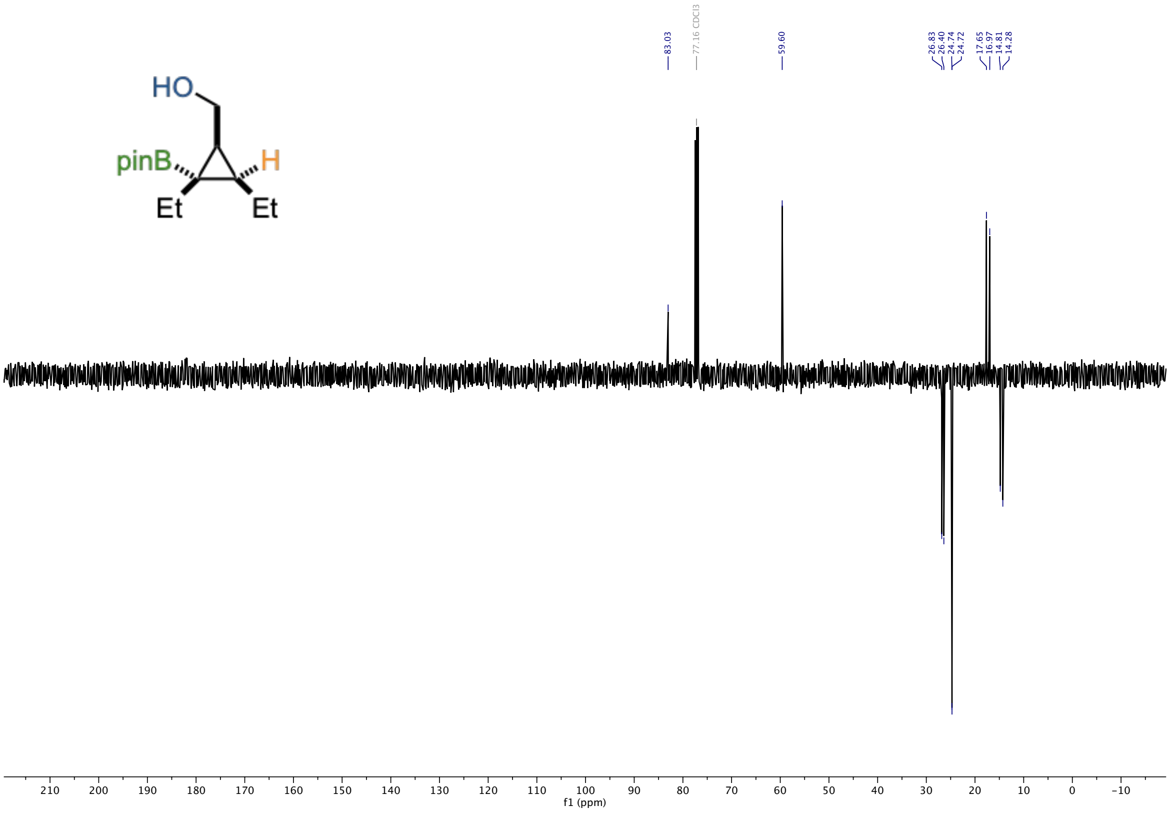

^{13}C NMR (APT) spectrum (101 MHz, $CDCl_3$)

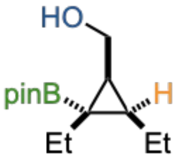

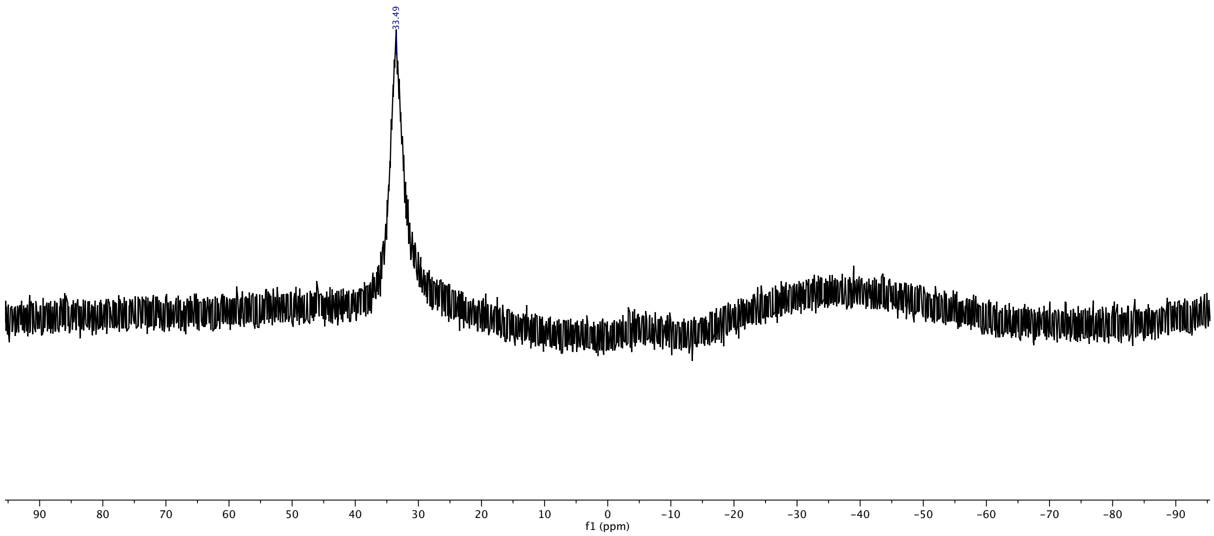

^{11}B NMR spectrum (128 MHz, $CDCl_3$)

((1*R,2*S**,3*S**)-2-Allyl-2,3-dimethyl-3-(4,4,5,5-tetramethyl-1,3,2-dioxaborolan-2-yl)cyclopropyl)methanol (3g)**

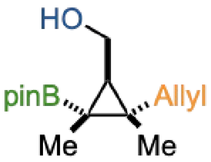

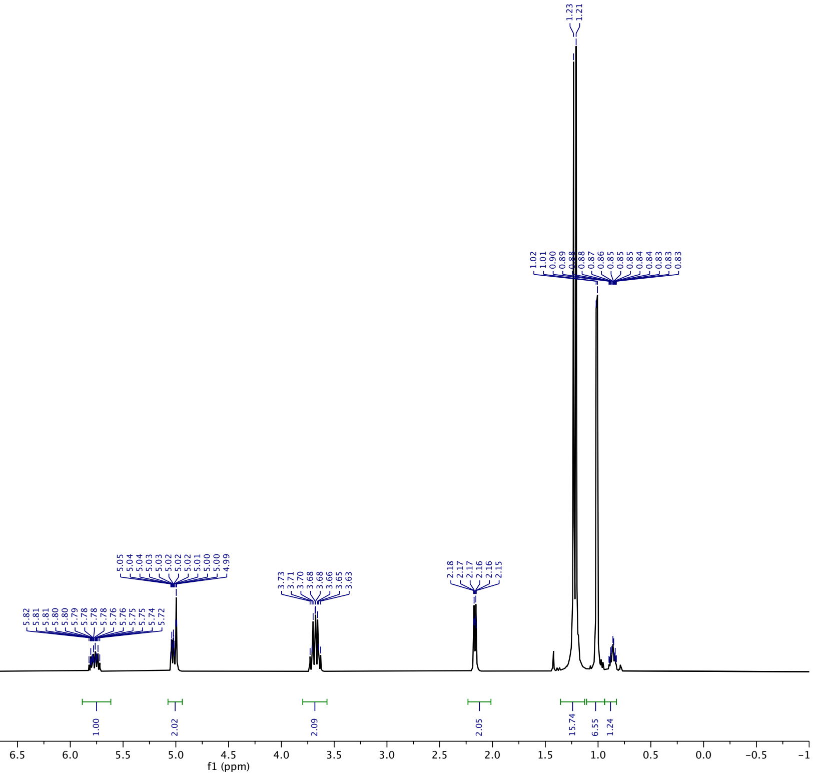

^{1}H NMR spectrum (400 MHz, $CDCl_3$)

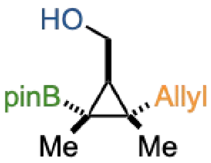

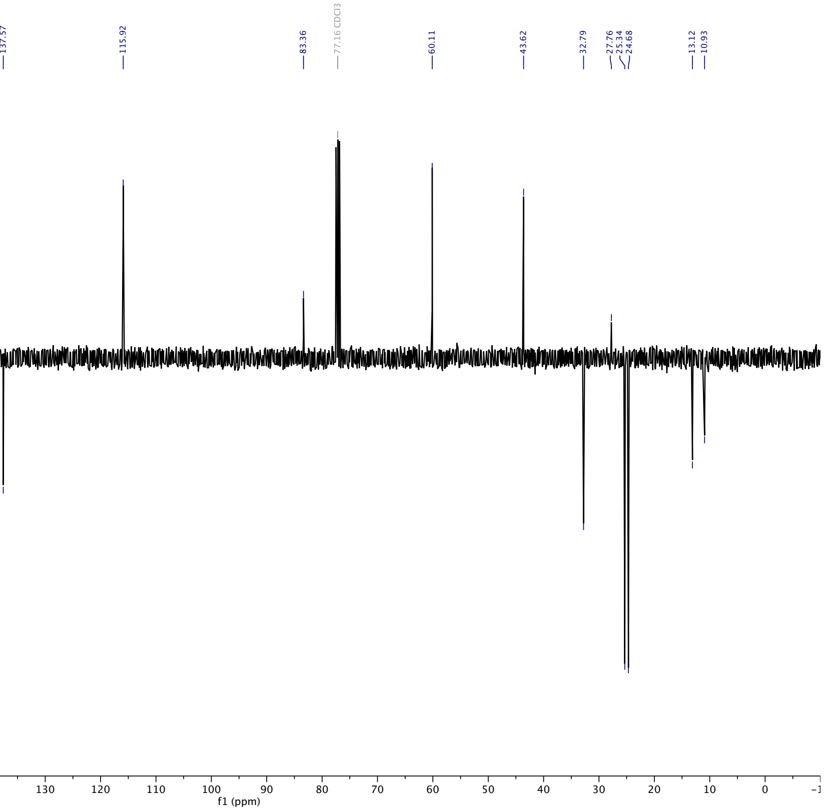

^{13}C NMR (APT) spectrum (101 MHz, $CDCl_3$)

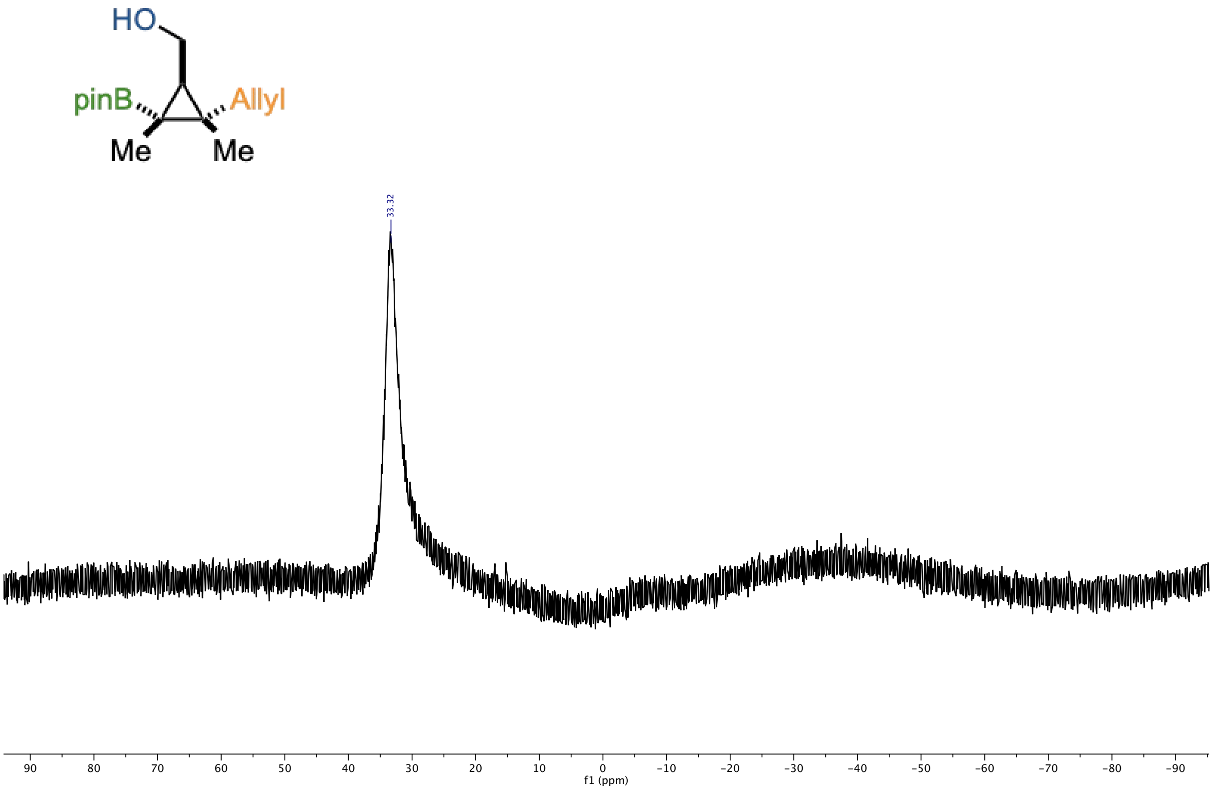

^{11}B NMR spectrum (128 MHz, $CDCl_3$)

2-((1*R,2*S**)-1-Butyl-2-(iodomethyl)cyclopropyl)-4,4,5,5-tetramethyl-1,3,2-dioxaborolane (4a)**

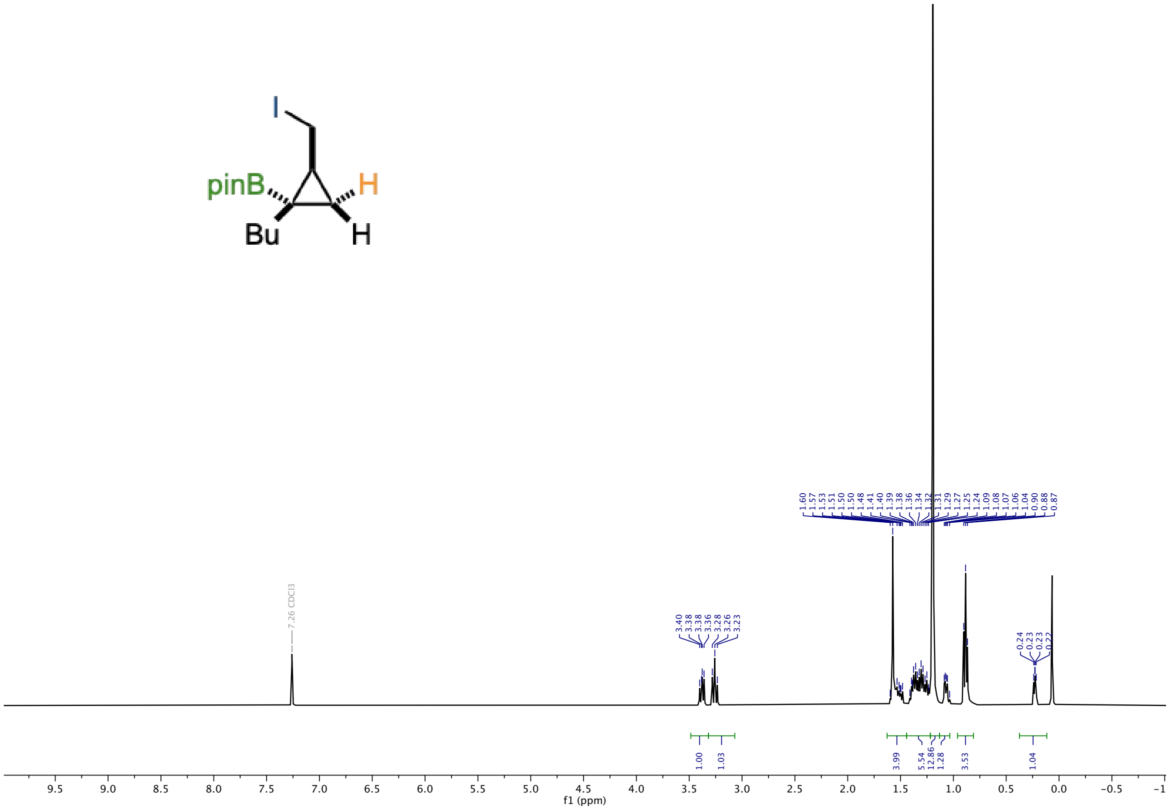

¹H NMR spectrum (400 MHz, $CDCl_3$)

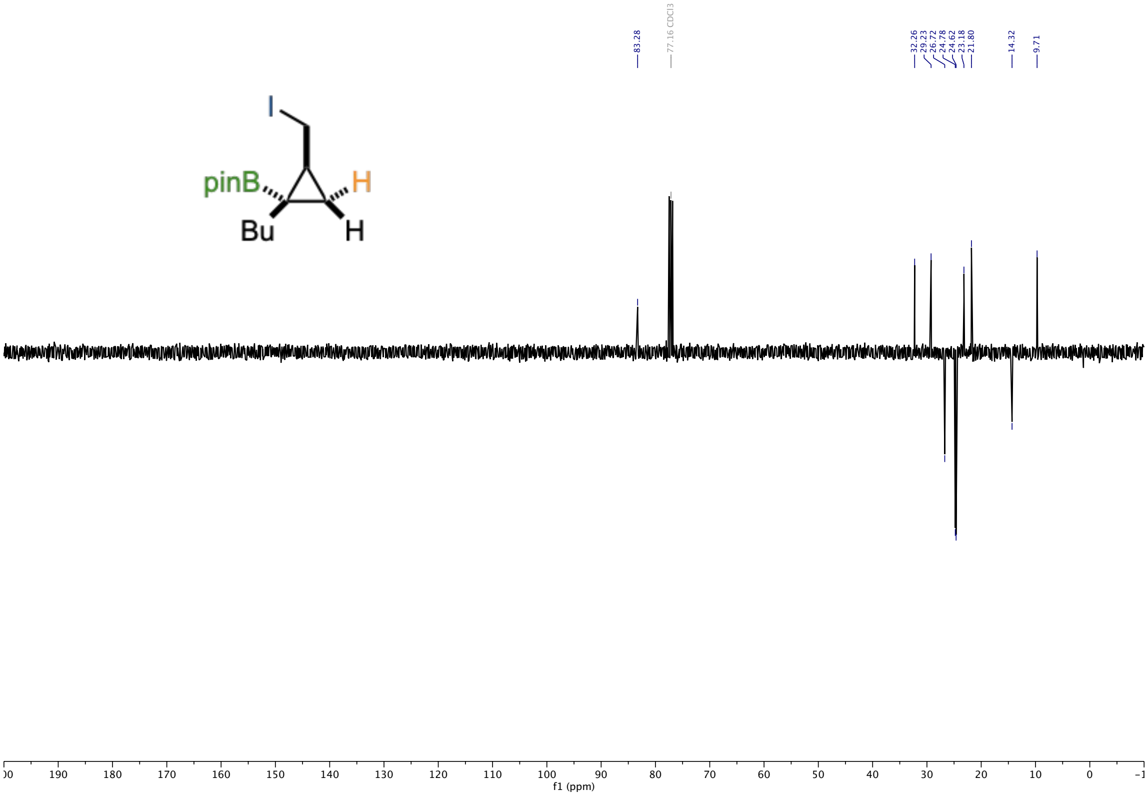

¹³C NMR (APT) spectrum (101 MHz, $CDCl_3$)

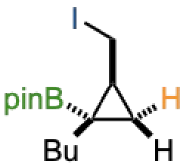

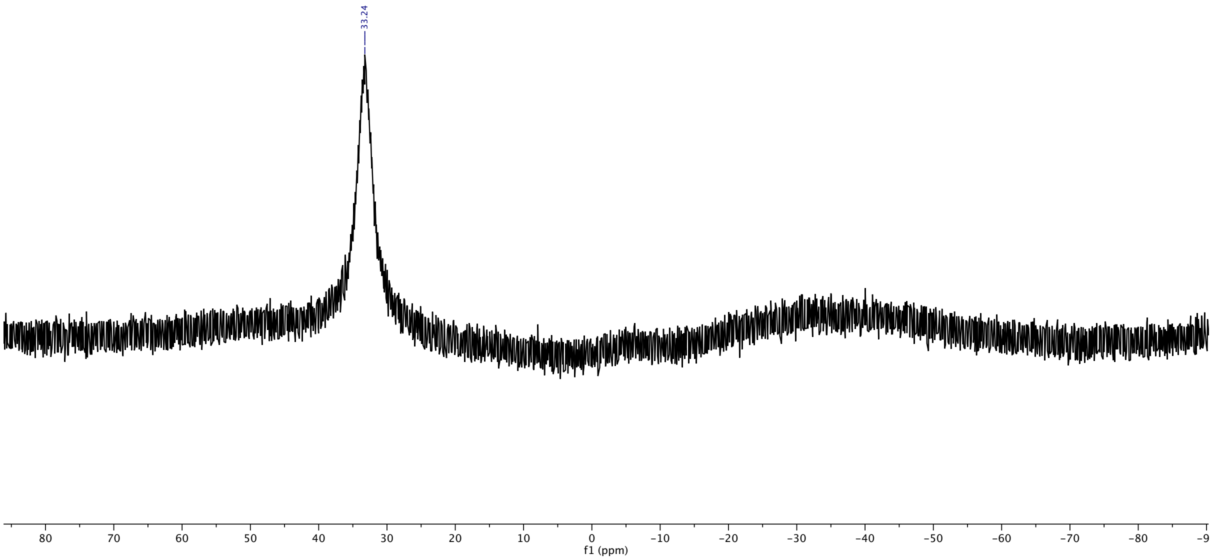

^{11}B NMR spectrum (128 MHz, $CDCl_3$)

2-((1*R,2*S**)-1-(3-Chloropropyl)-2-(iodomethyl)cyclopropyl)-4,4,5,5-tetramethyl-1,3,2-dioxaborolane (4b)**

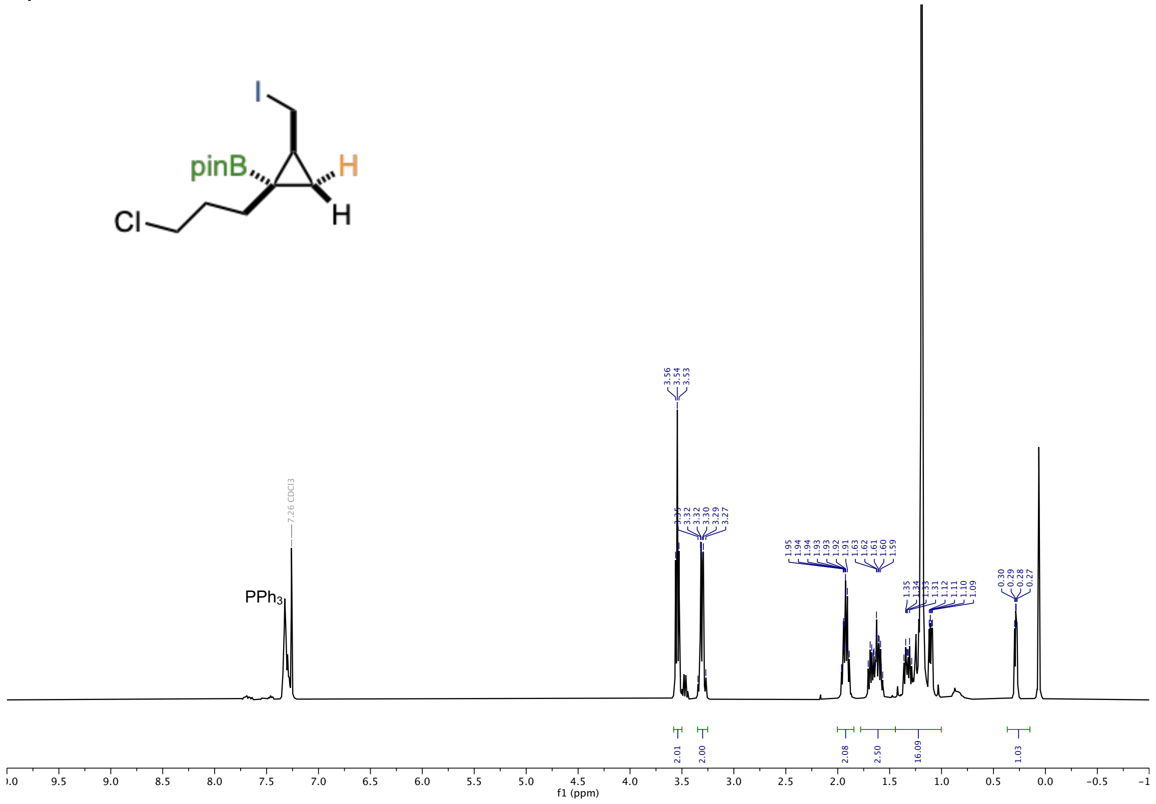

^{1}**H NMR** spectrum (400 MHz, $CDCl_3$)

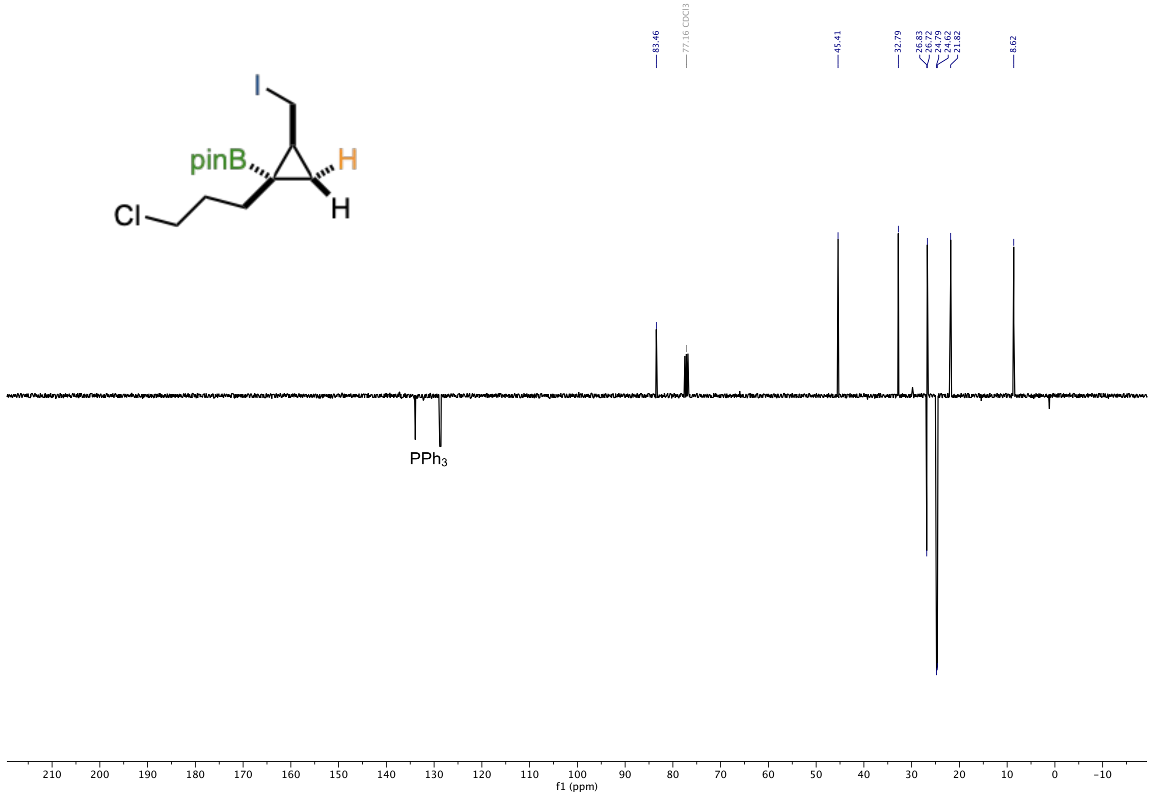

^{13}**C NMR** (APT) spectrum (101 MHz, $CDCl_3$)

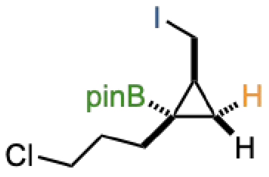

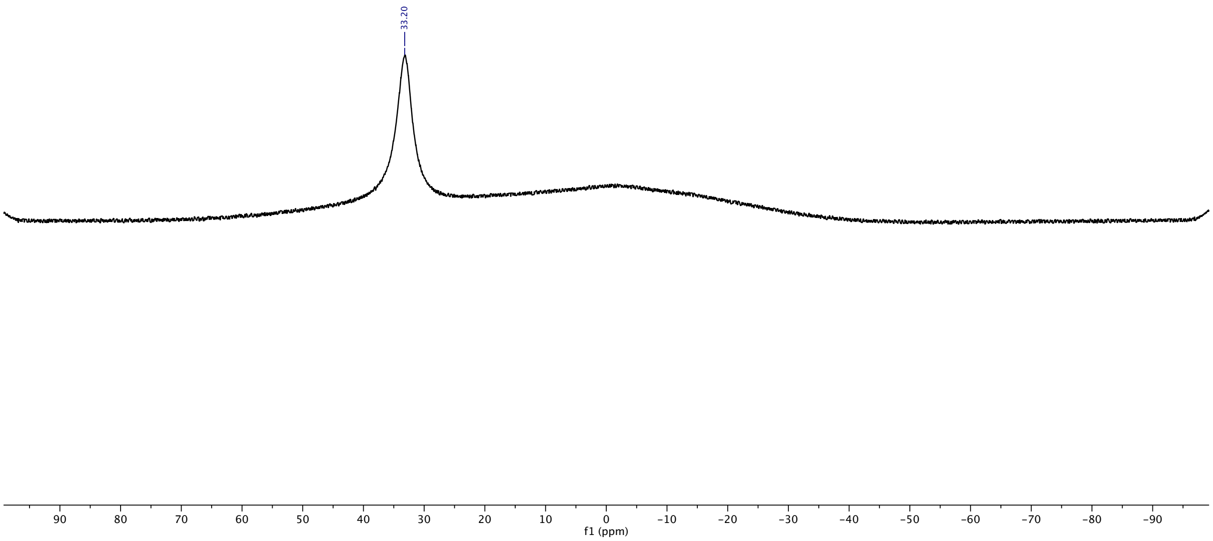

^{11}B NMR spectrum (128 MHz, $CDCl_3$)

2-((1*R,2*S**,3*S**)-2-Allyl-1-butyl-3-(iodomethyl)cyclopropyl)-4,4,5,5-tetramethyl-1,3,2-dioxaborolane (4c)**

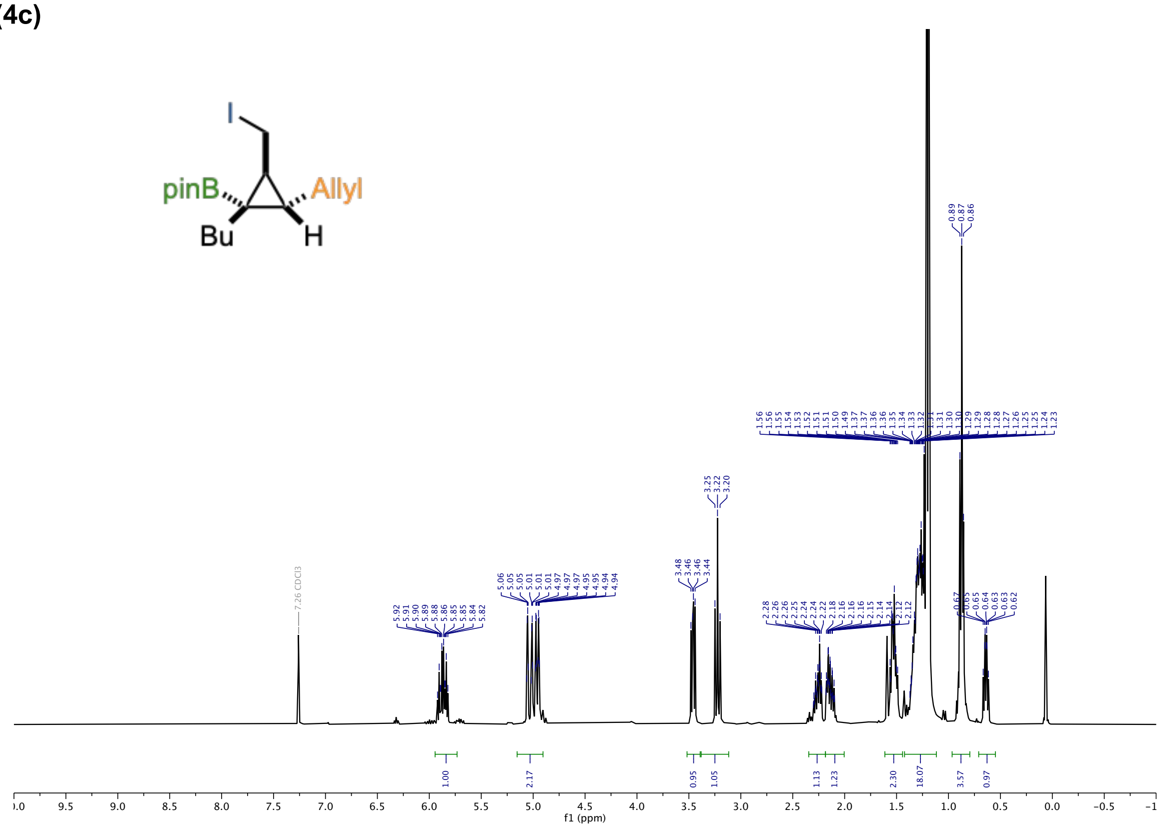

^{1}H NMR spectrum (400 MHz, CDCl$_3$)

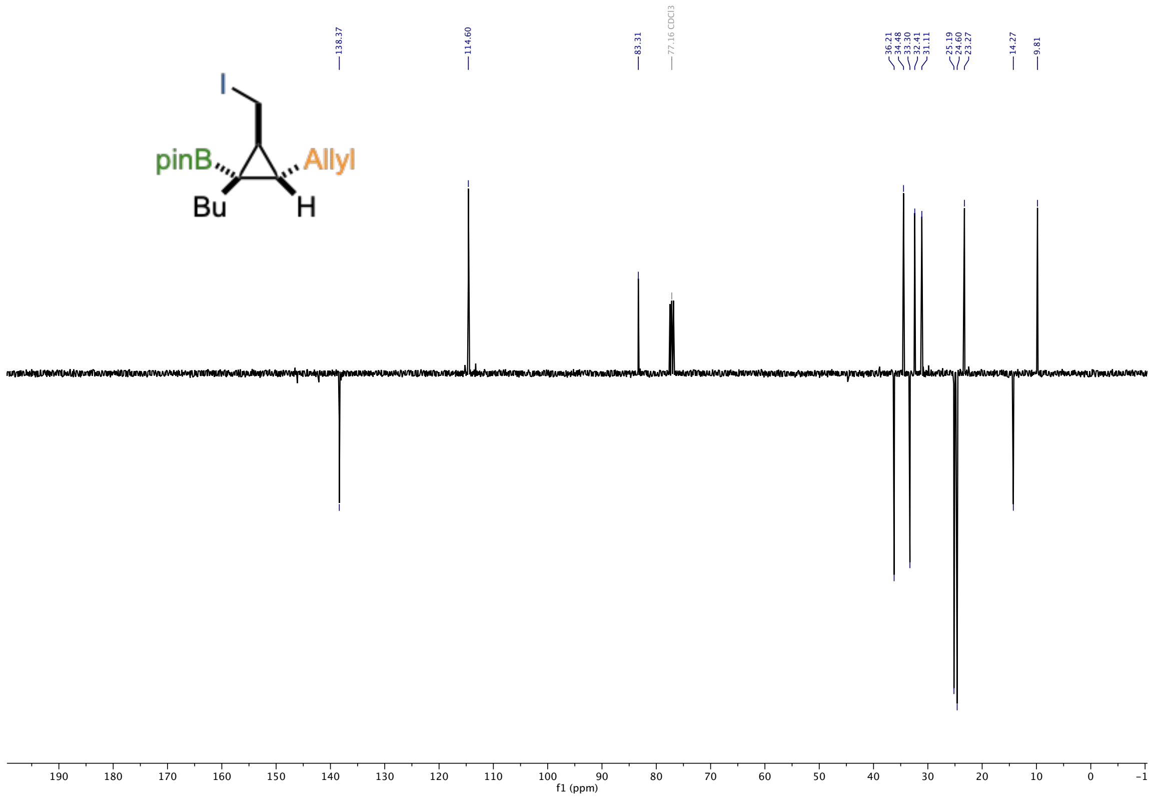

^{13}C NMR (APT) spectrum (101 MHz, CDCl$_3$)

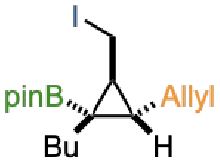

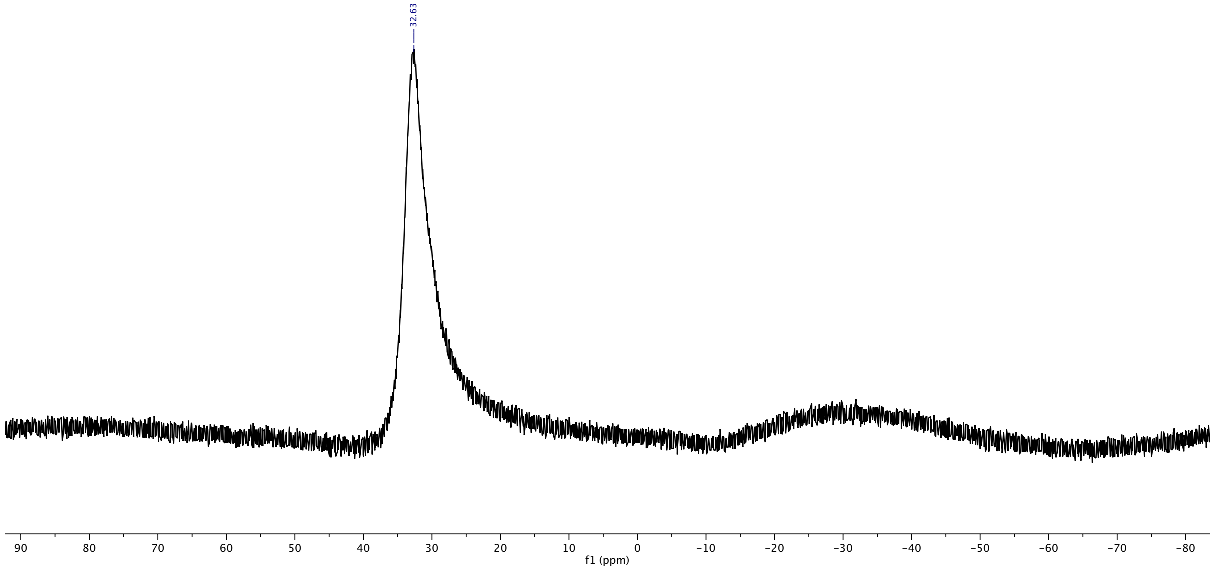

^{11}B NMR spectrum (128 MHz, $CDCl_3$)

2-((1*R,2*S**,3*R**)-2-(Iodomethyl)-1,3-dimethylcyclopropyl)-4,4,5,5-tetramethyl-1,3,2-dioxaborolane (4d)**

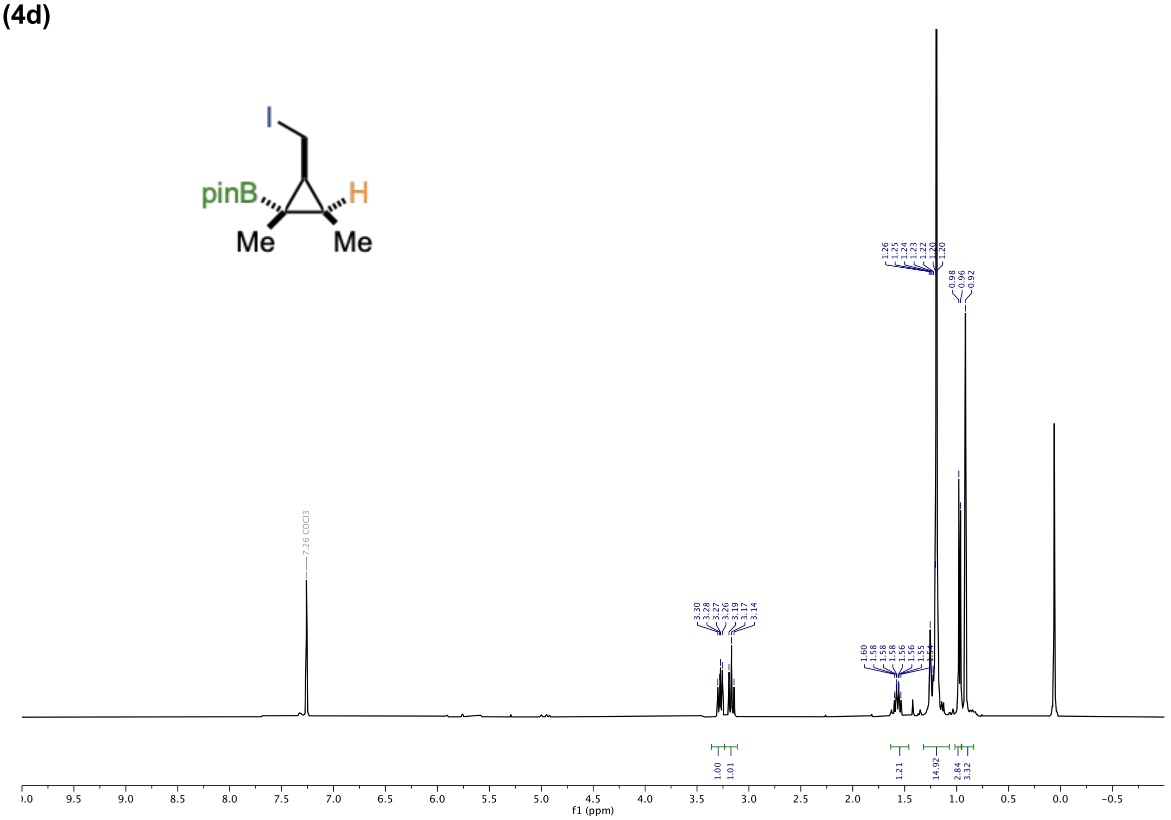

[1]**H NMR** spectrum (400 MHz, $CDCl_3$)

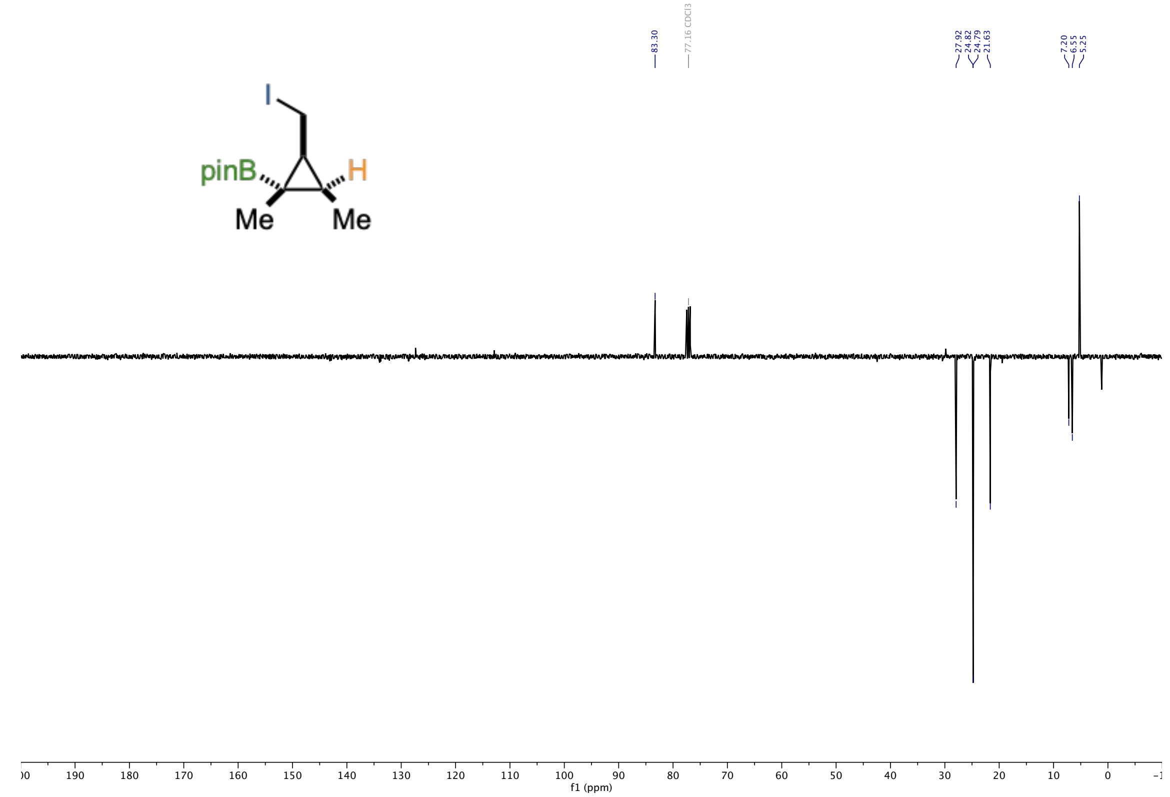

[13]**C NMR** (APT) spectrum (101 MHz, $CDCl_3$)

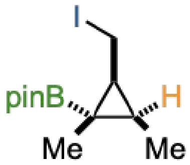

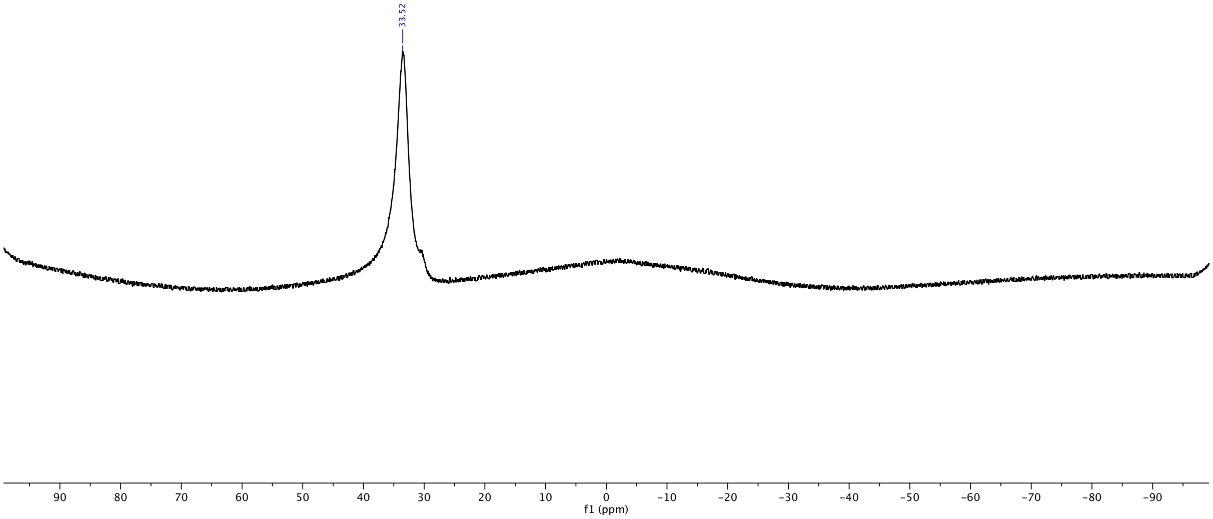

^{11}B NMR spectrum (128 MHz, $CDCl_3$)

2-((1*R**,2*S**,3*R**)-1-Ethyl-2-(iodomethyl)-3-phenylcyclopropyl)-4,4,5,5-tetramethyl-1,3,2-dioxaborolane (4e)

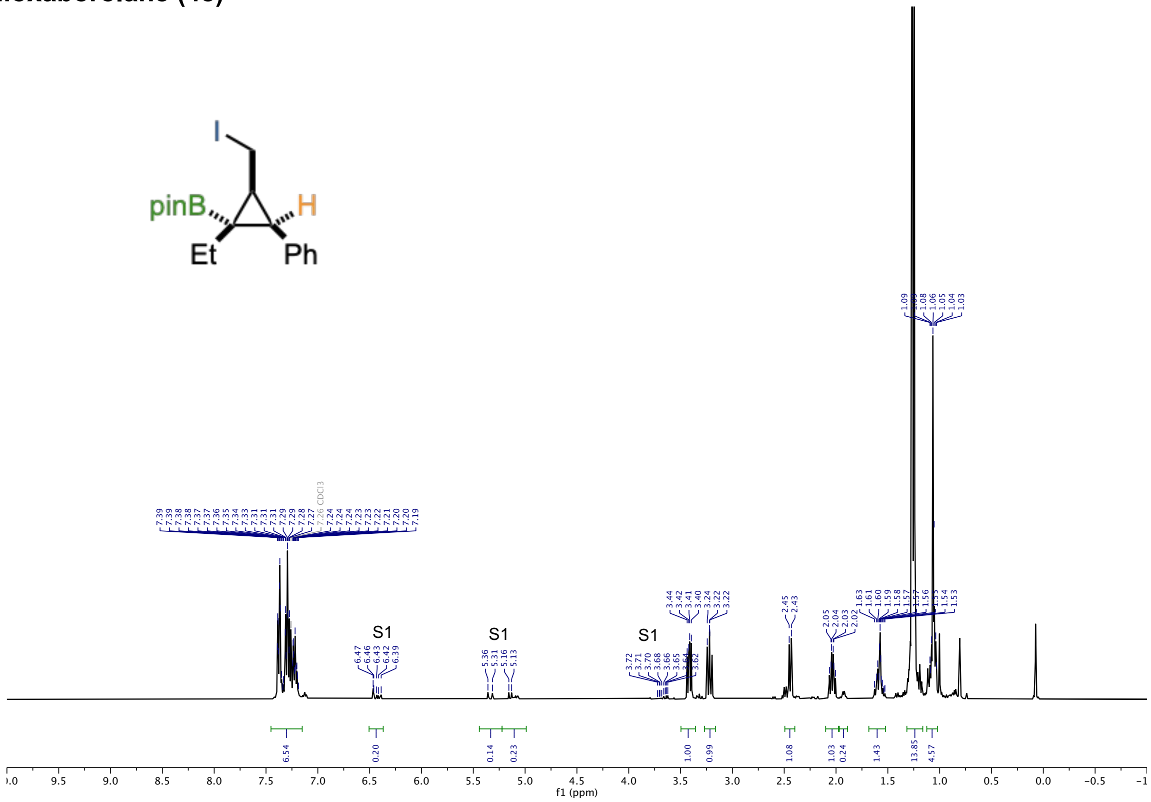

^{1}H NMR spectrum (400 MHz, $CDCl_3$)

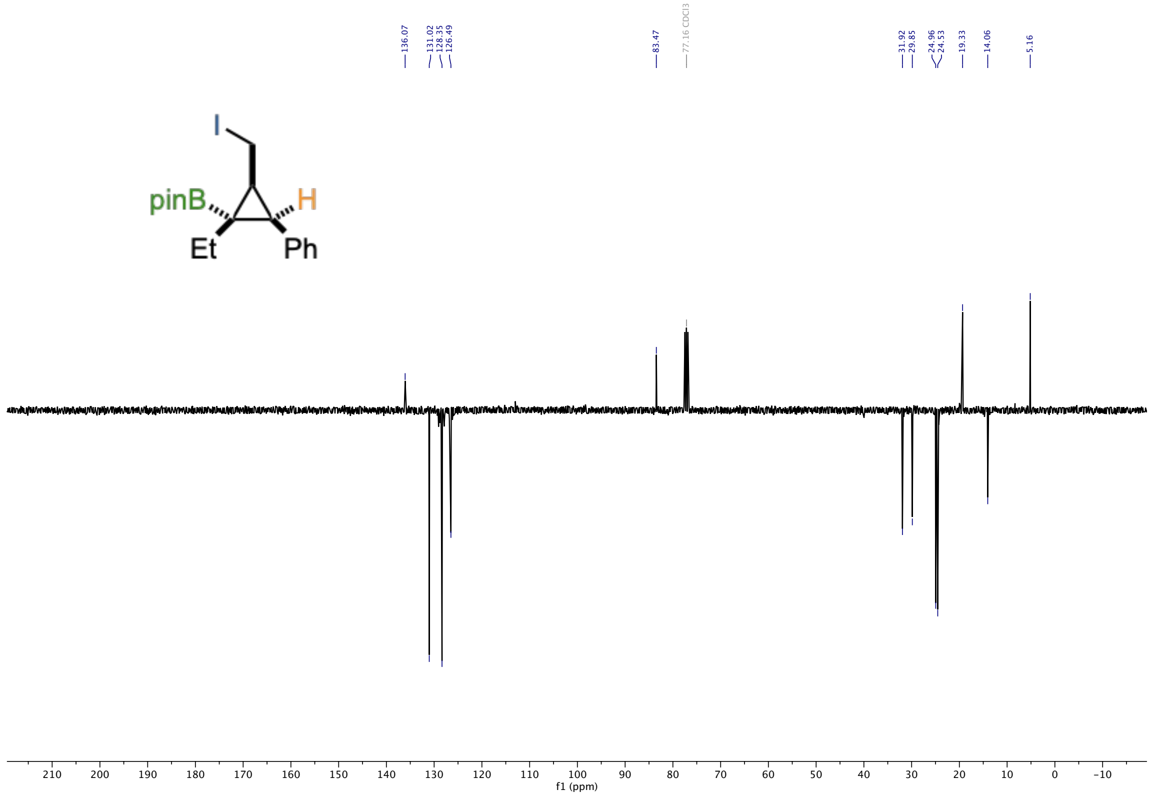

^{13}C NMR (APT) spectrum (101 MHz, $CDCl_3$)

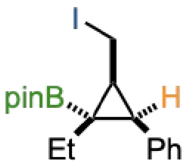

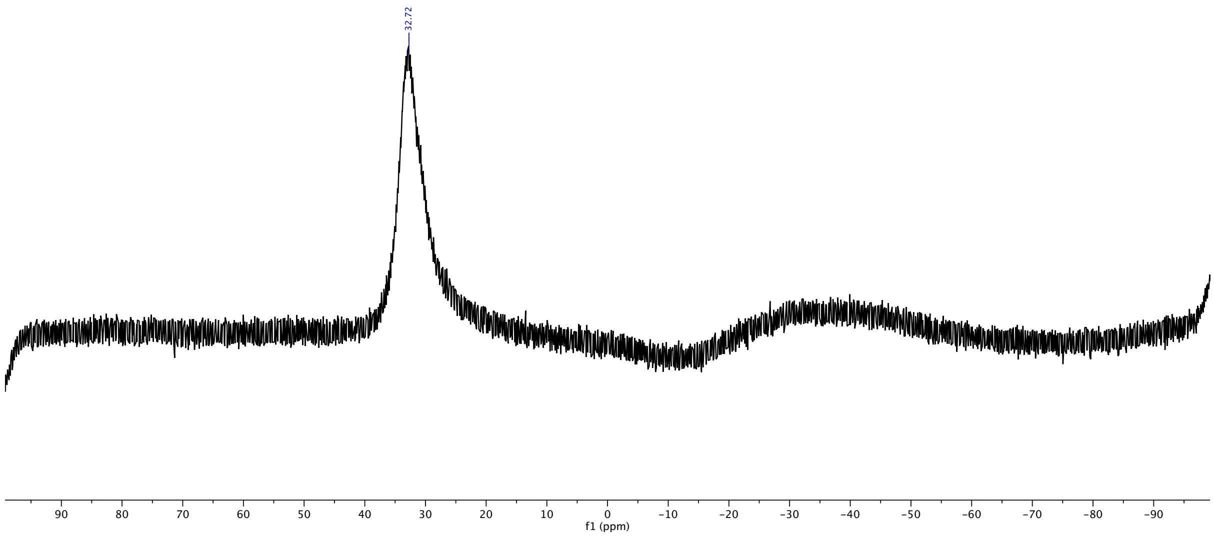

^{11}B NMR spectrum (128 MHz, $CDCl_3$)

2-((1*R**,2*R**,3*S**)-1,2-Diethyl-3-(iodomethyl)cyclopropyl)-4,4,5,5-tetramethyl-1,3,2-dioxaborolane (4f)

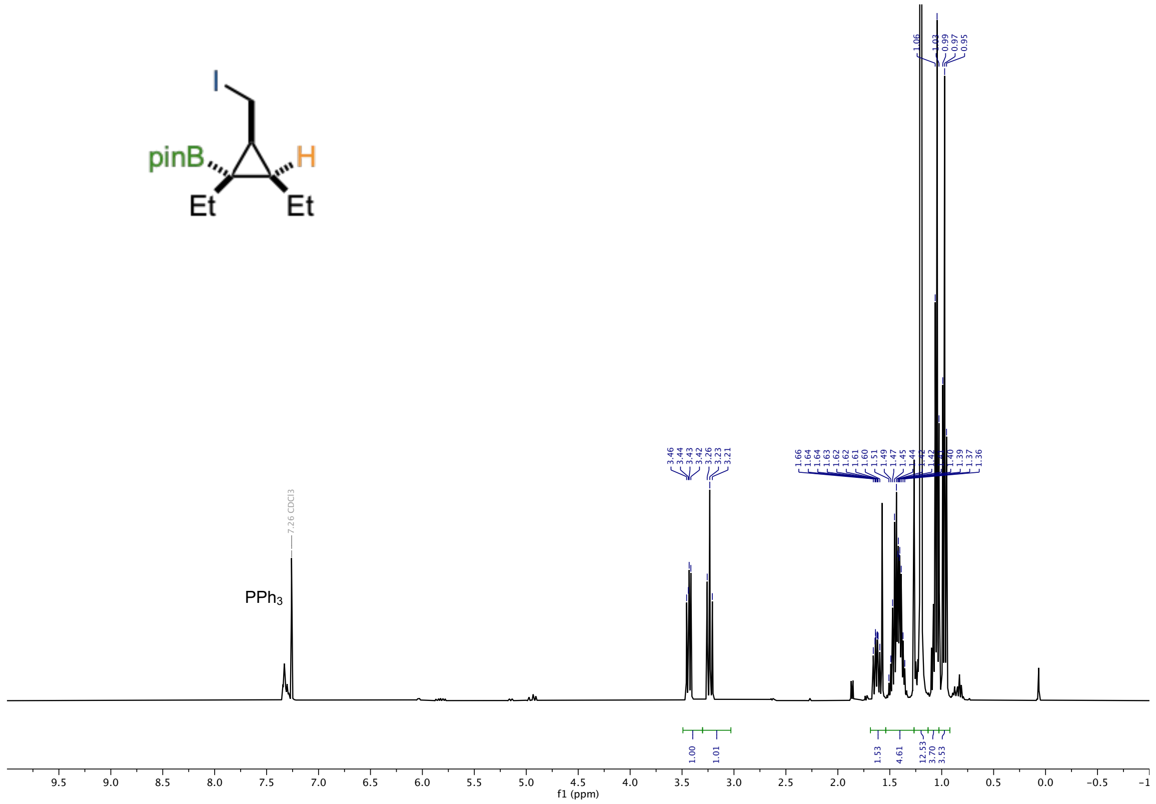

^{1}**H NMR** spectrum (400 MHz, $CDCl_3$)

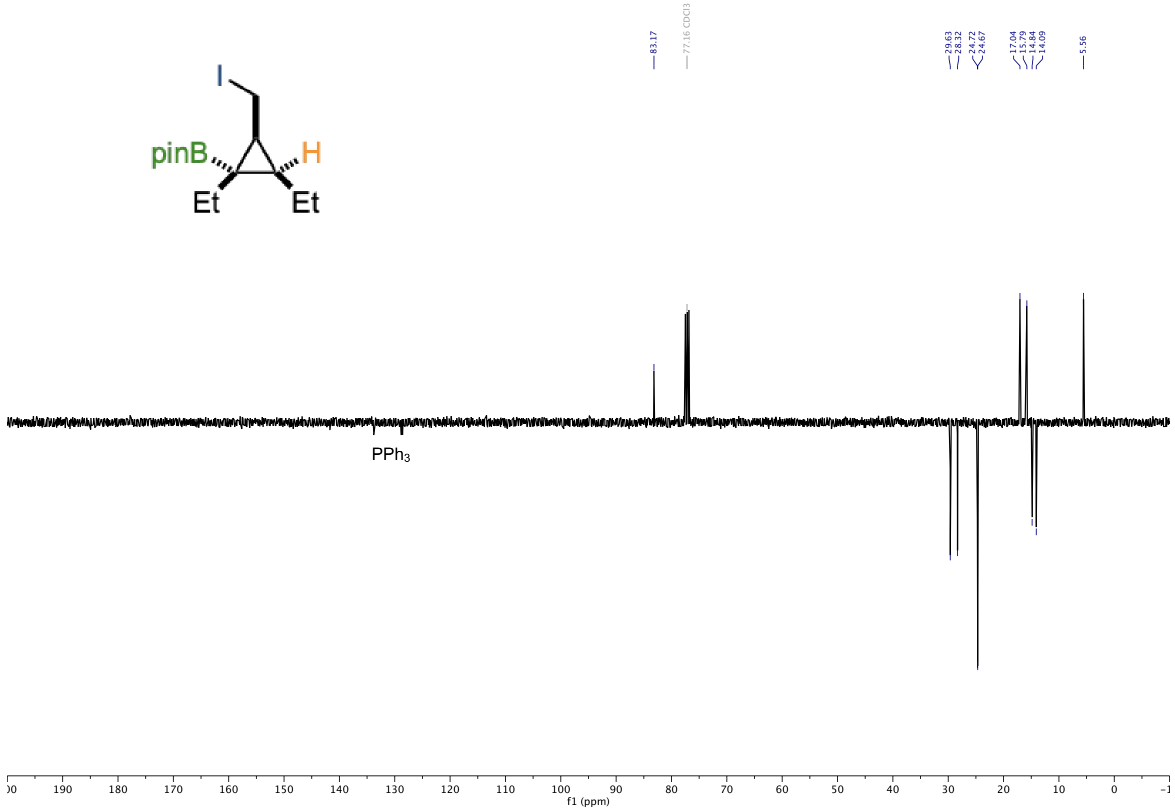

^{13}**C NMR** (APT) spectrum (101 MHz, $CDCl_3$)

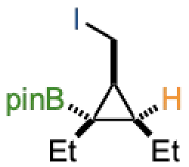

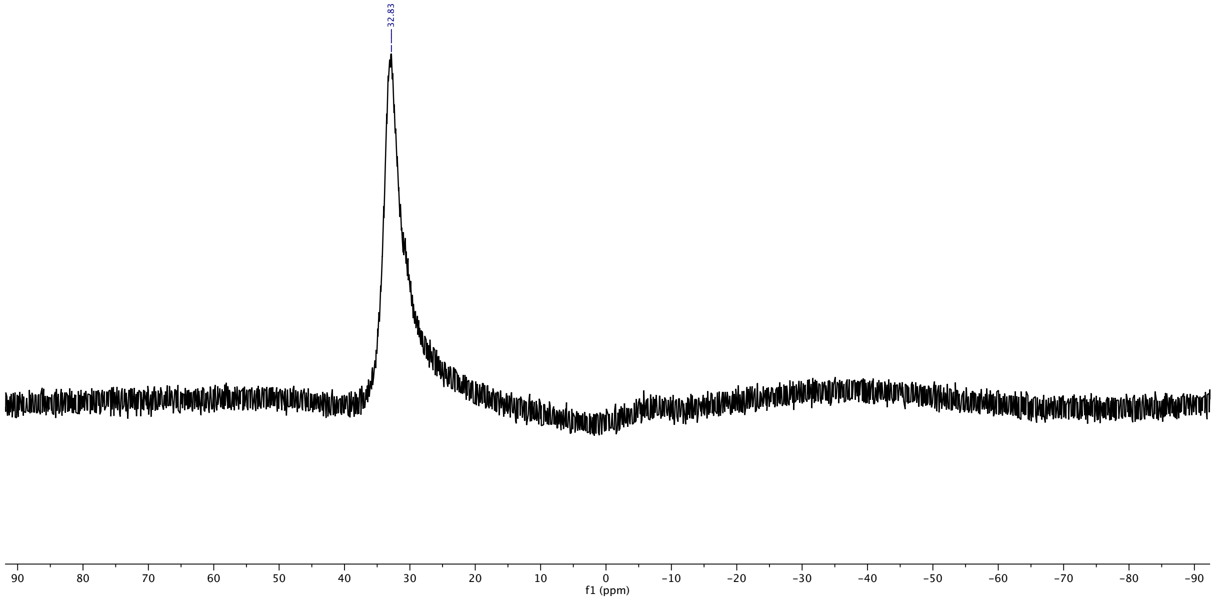

¹¹B NMR spectrum (128 MHz, $CDCl_3$)

2-((1*S**,2*S**,3*R**)-2-Allyl-3-(iodomethyl)-1,2-dimethylcyclopropyl)-4,4,5,5-tetramethyl-1,3,2-dioxaborolane (4g)

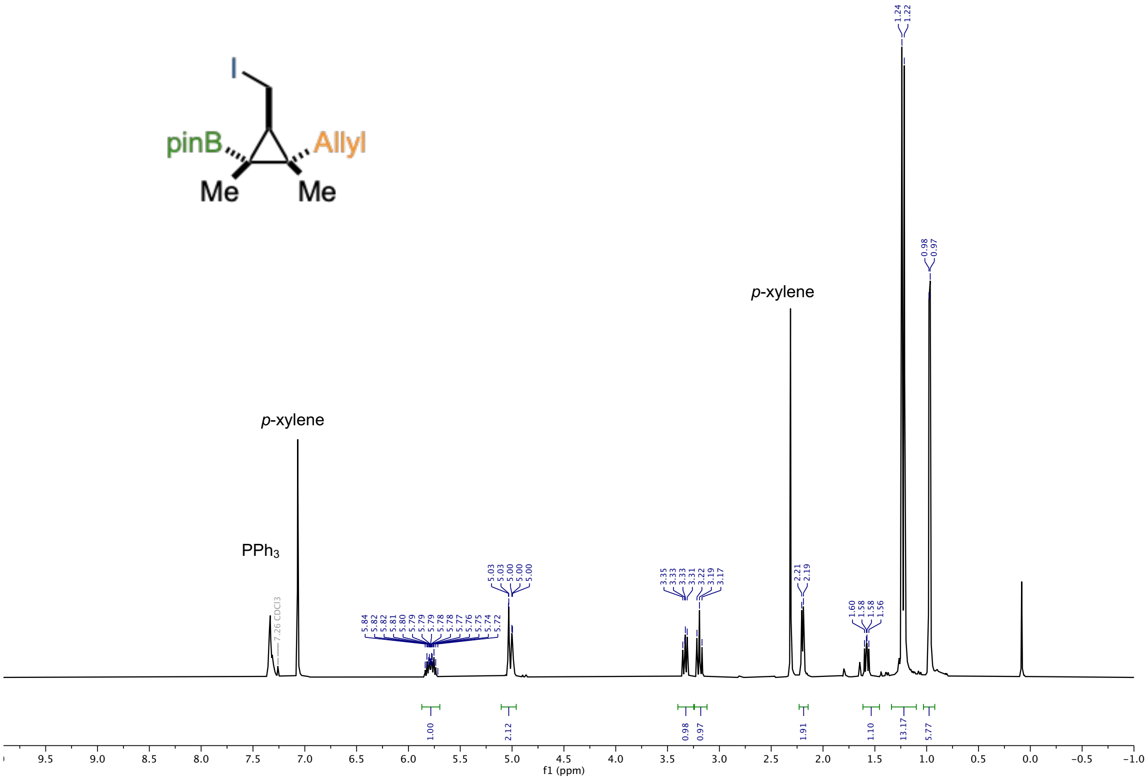

¹H NMR spectrum (400 MHz, $CDCl_3$)

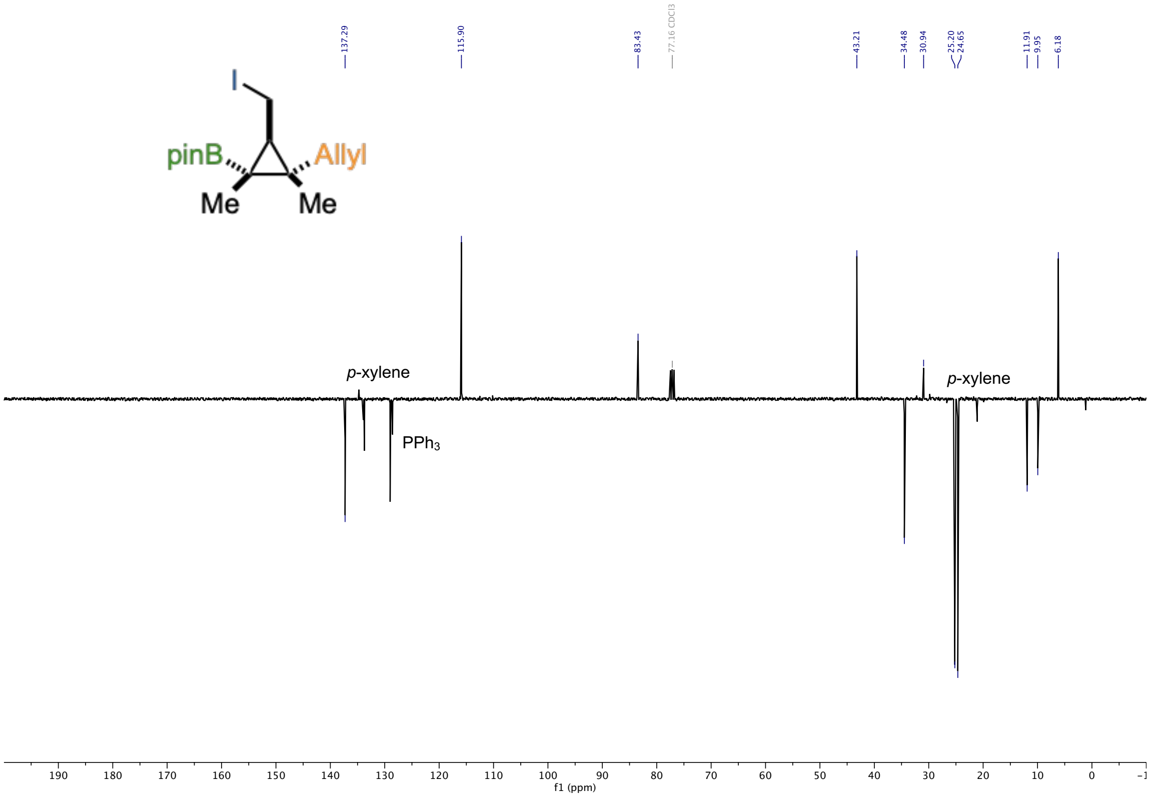

¹³C NMR (APT) spectrum (101 MHz, $CDCl_3$)

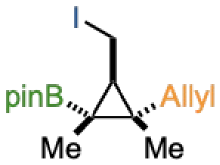

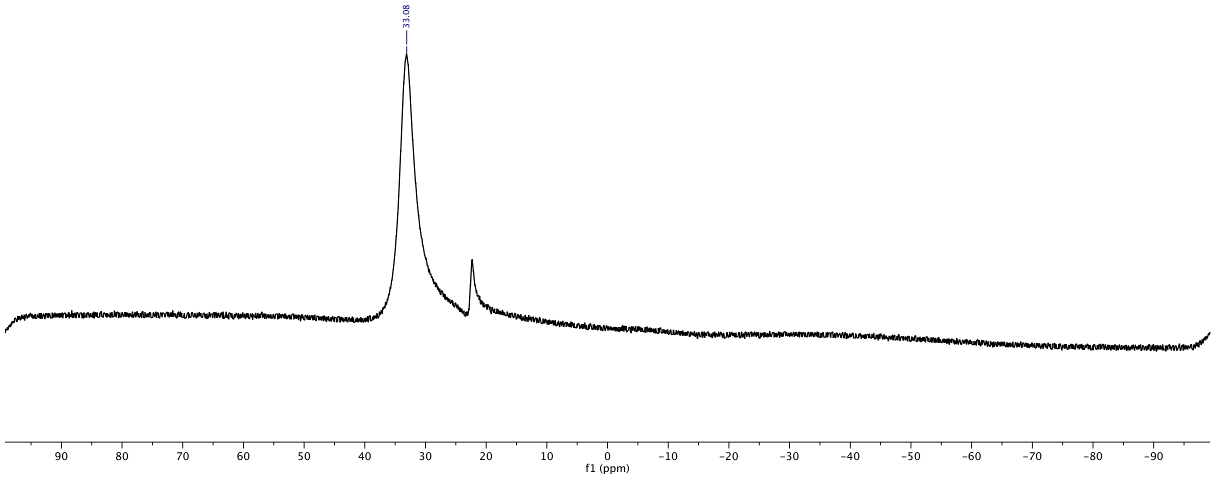

^{11}B NMR spectrum (128 MHz, $CDCl_3$)

(3-Allylhepta-1,2-dien-1-yl)benzene (5a)

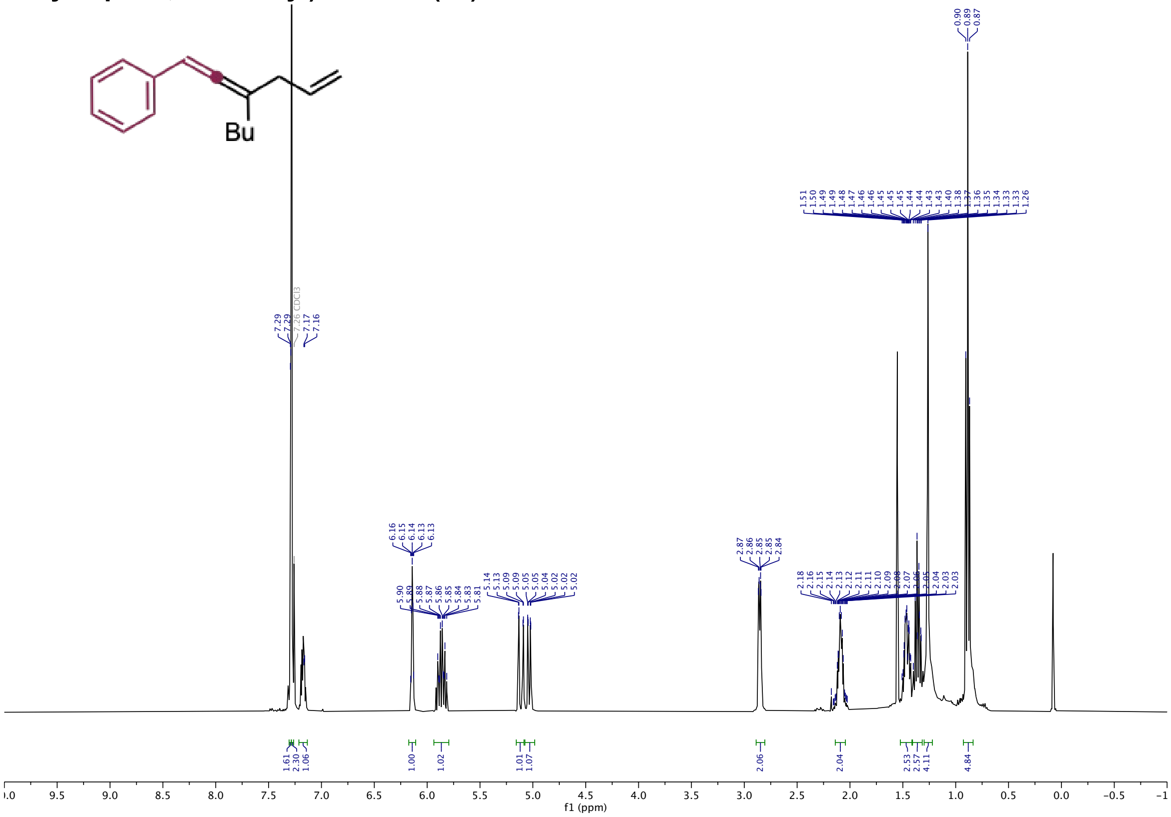

^{1}**H NMR** spectrum (400 MHz, $CDCl_3$)

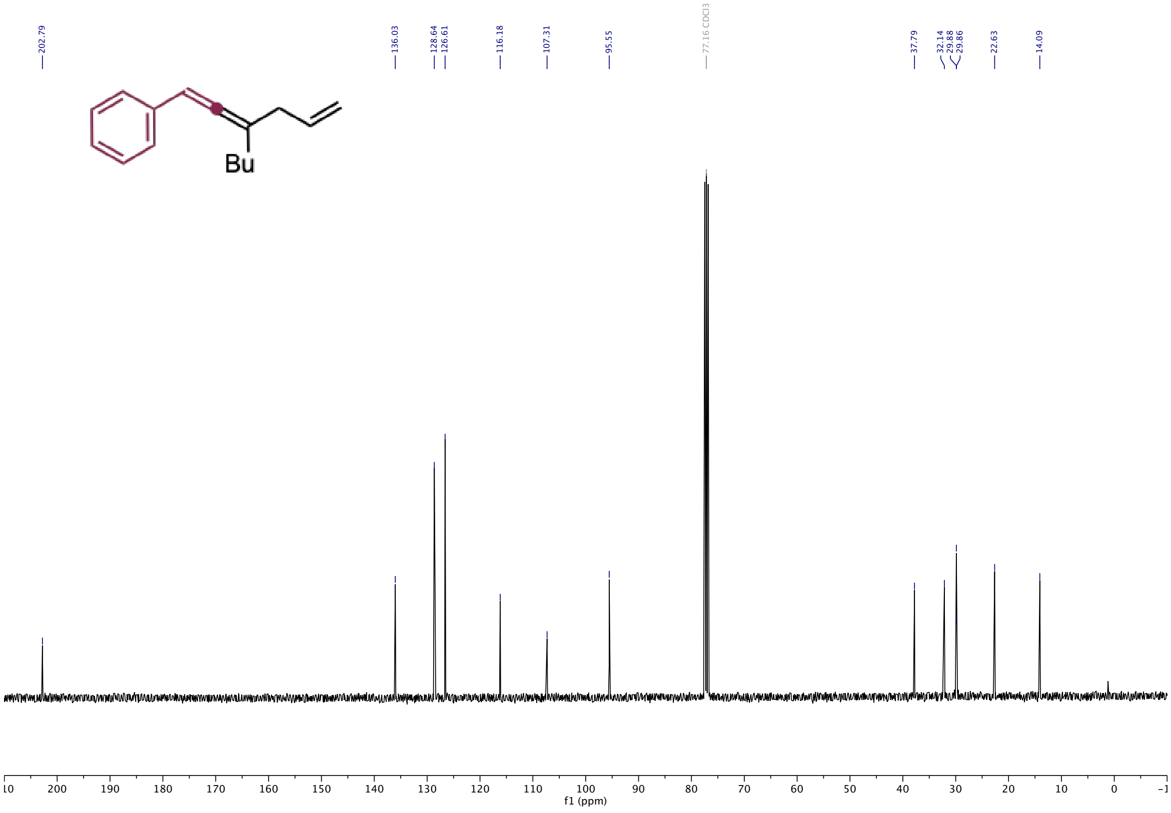

^{13}**C NMR** spectrum (101 MHz, $CDCl_3$)

4,4,5,5-Tetramethyl-2-(4-(phenylethynyl)oct-1-en-4-yl)-1,3,2-dioxaborolane (6a)

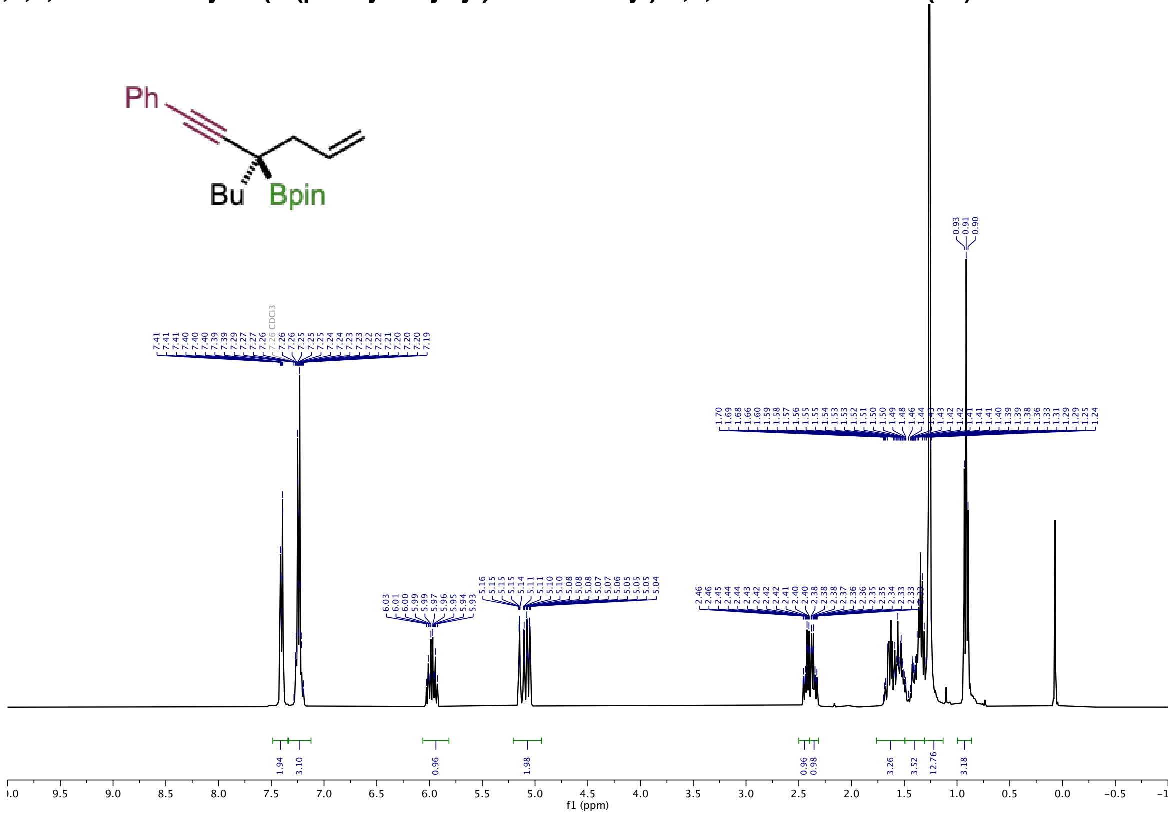

¹H NMR spectrum (400 MHz, $CDCl_3$)

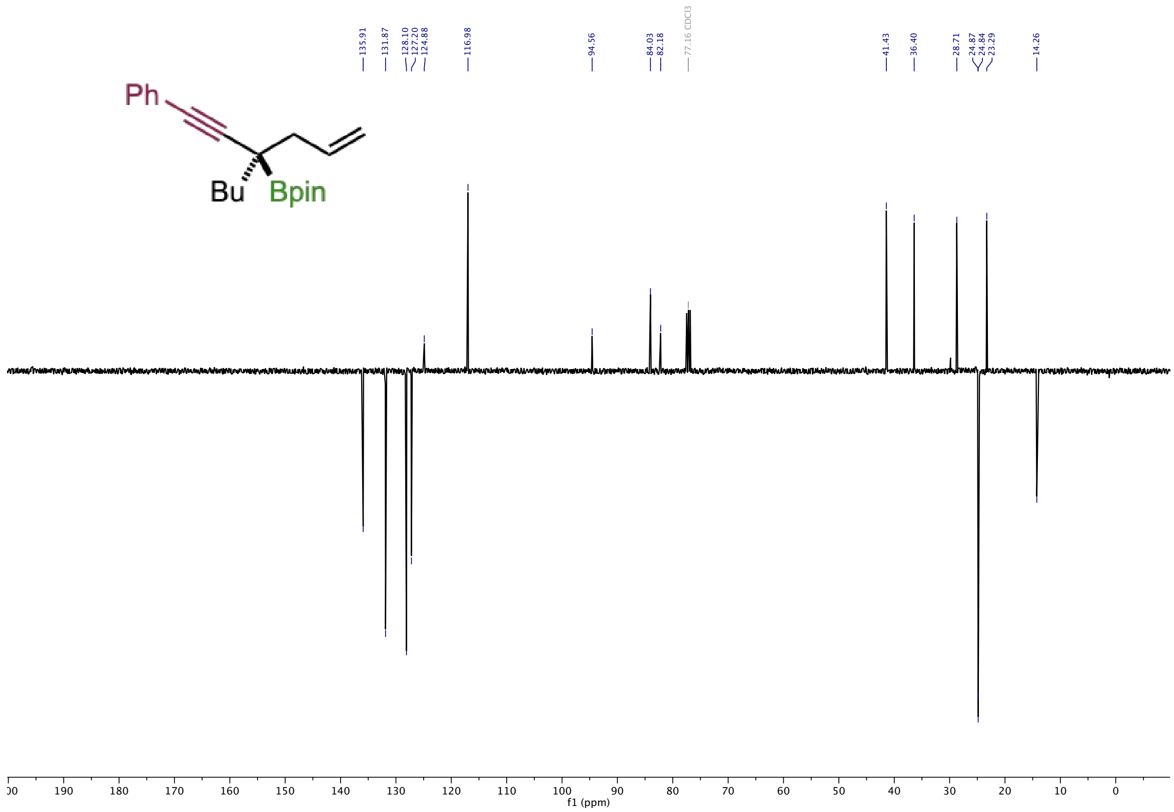

¹³C NMR (APT) spectrum (101 MHz, $CDCl_3$)

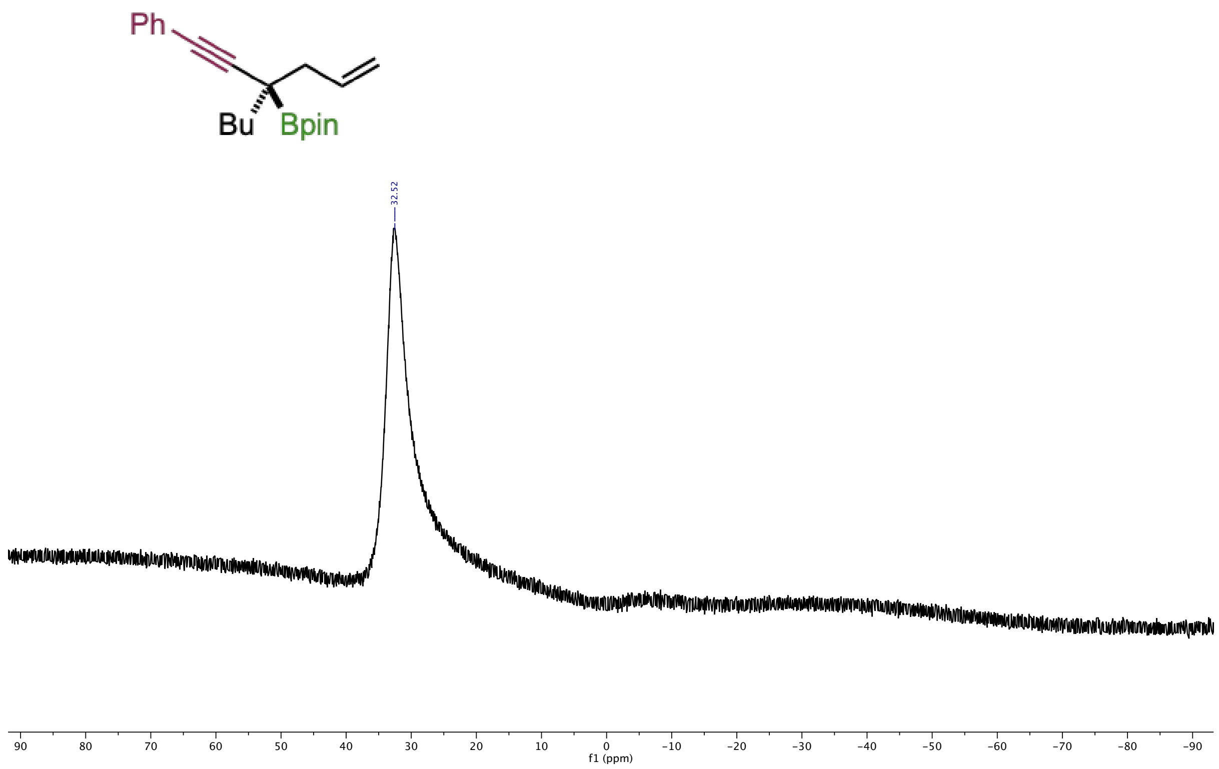

^{11}B NMR spectrum (128 MHz, $CDCl_3$)

2-(5-Allylundec-6-yn-5-yl)-4,4,5,5-tetramethyl-1,3,2-dioxaborolane (6b)

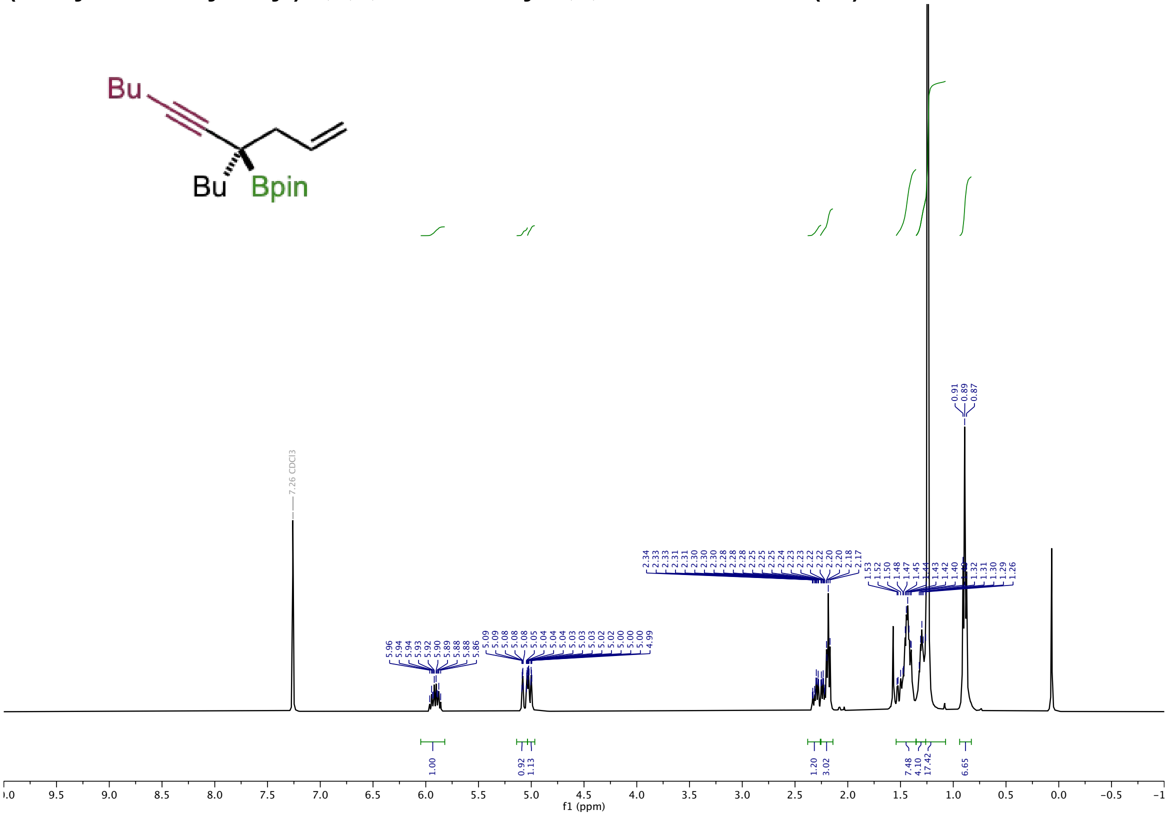

¹H NMR spectrum (400 MHz, $CDCl_3$)

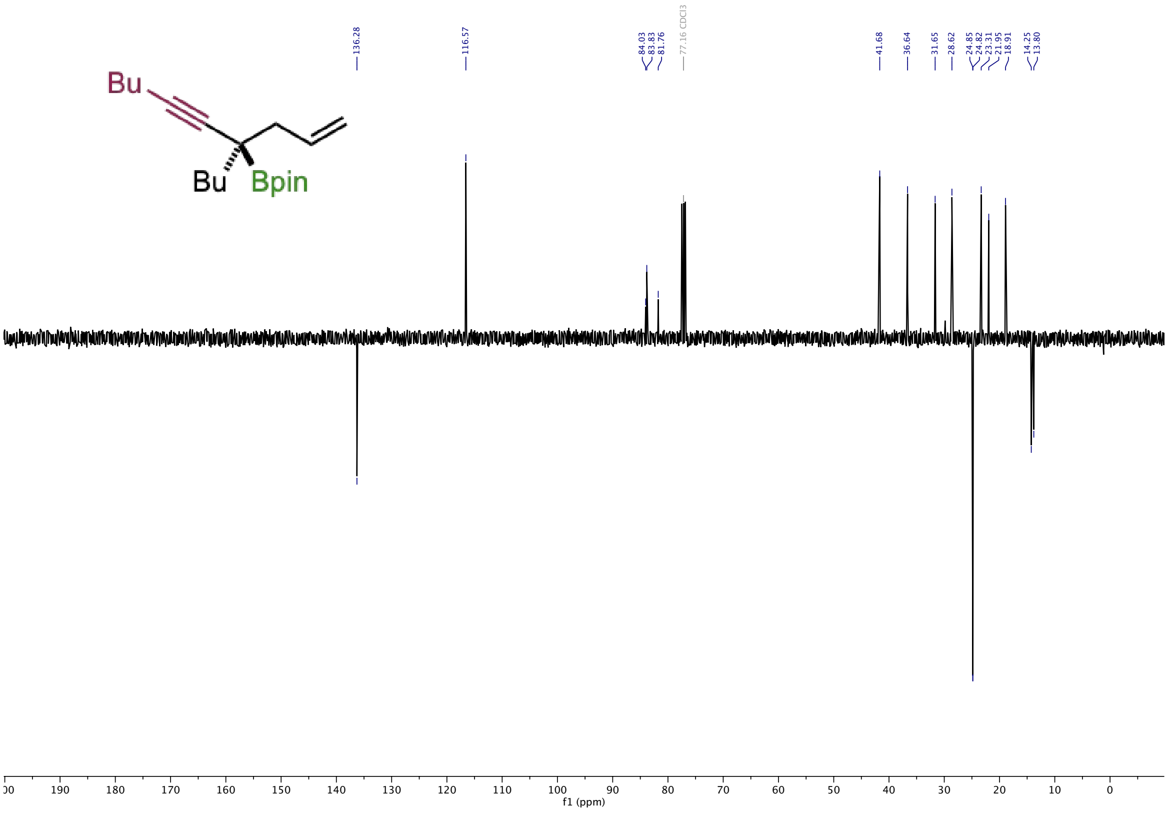

¹³C NMR (APT) spectrum (101 MHz, $CDCl_3$)

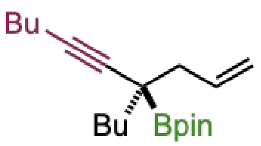

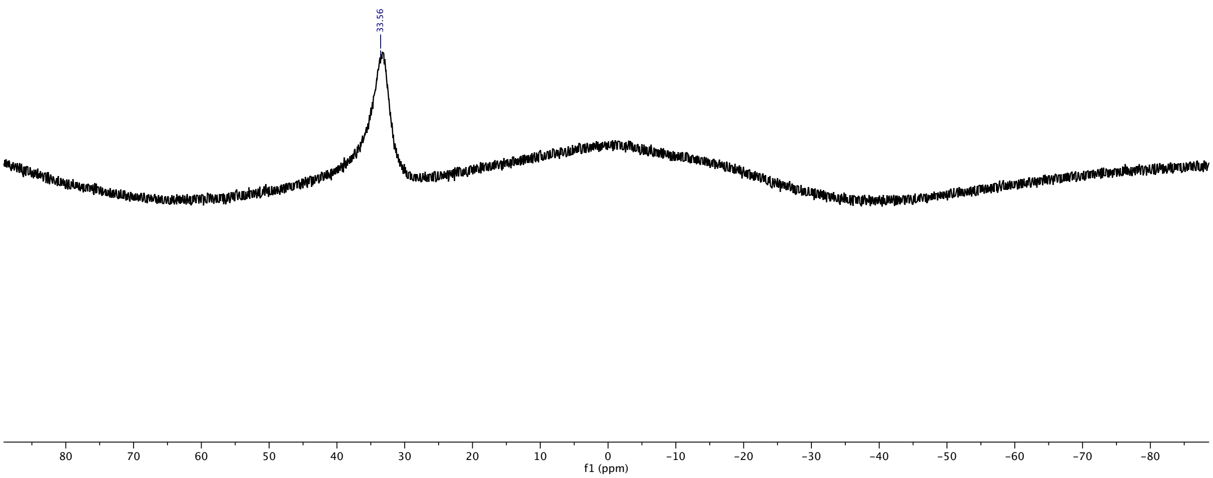

^{11}B NMR spectrum (128 MHz, $CDCl_3$)

2-(5-Allyl-2,2-dimethylnon-3-yn-5-yl)-4,4,5,5-tetramethyl-1,3,2-dioxaborolane (6c)

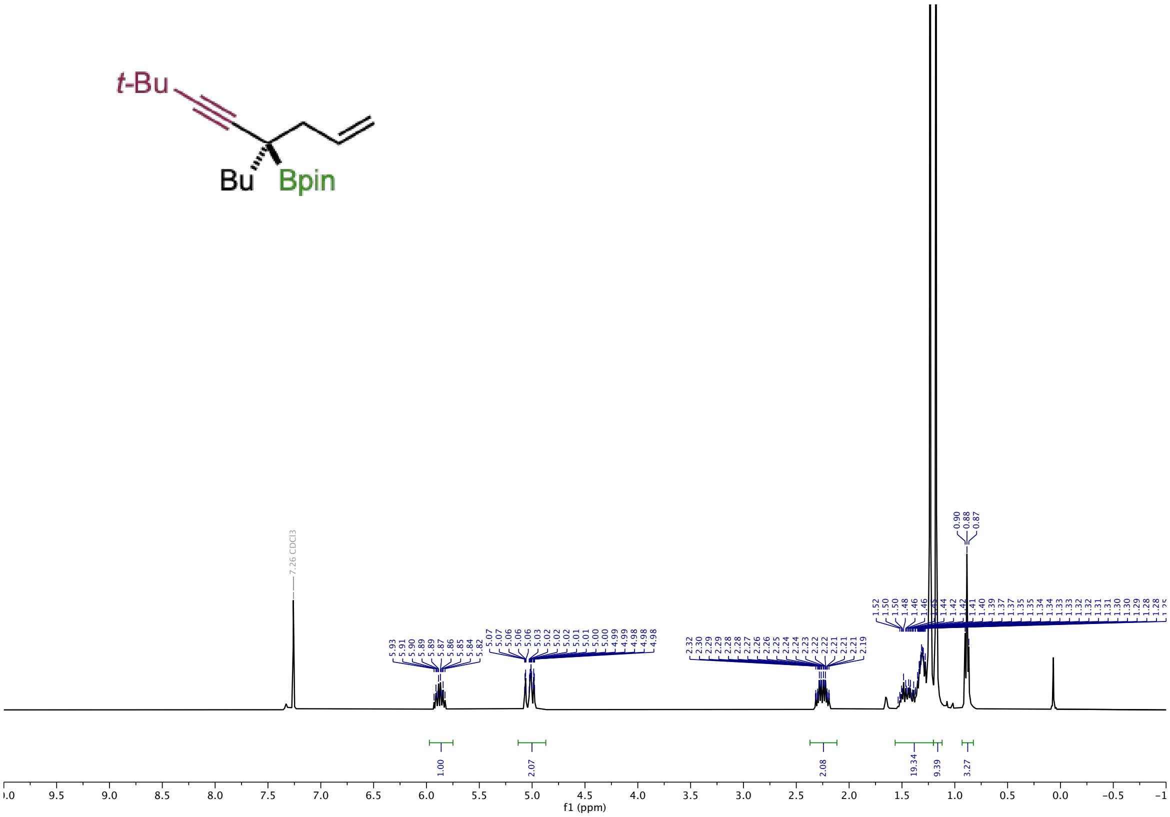

¹H NMR spectrum (400 MHz, $CDCl_3$)

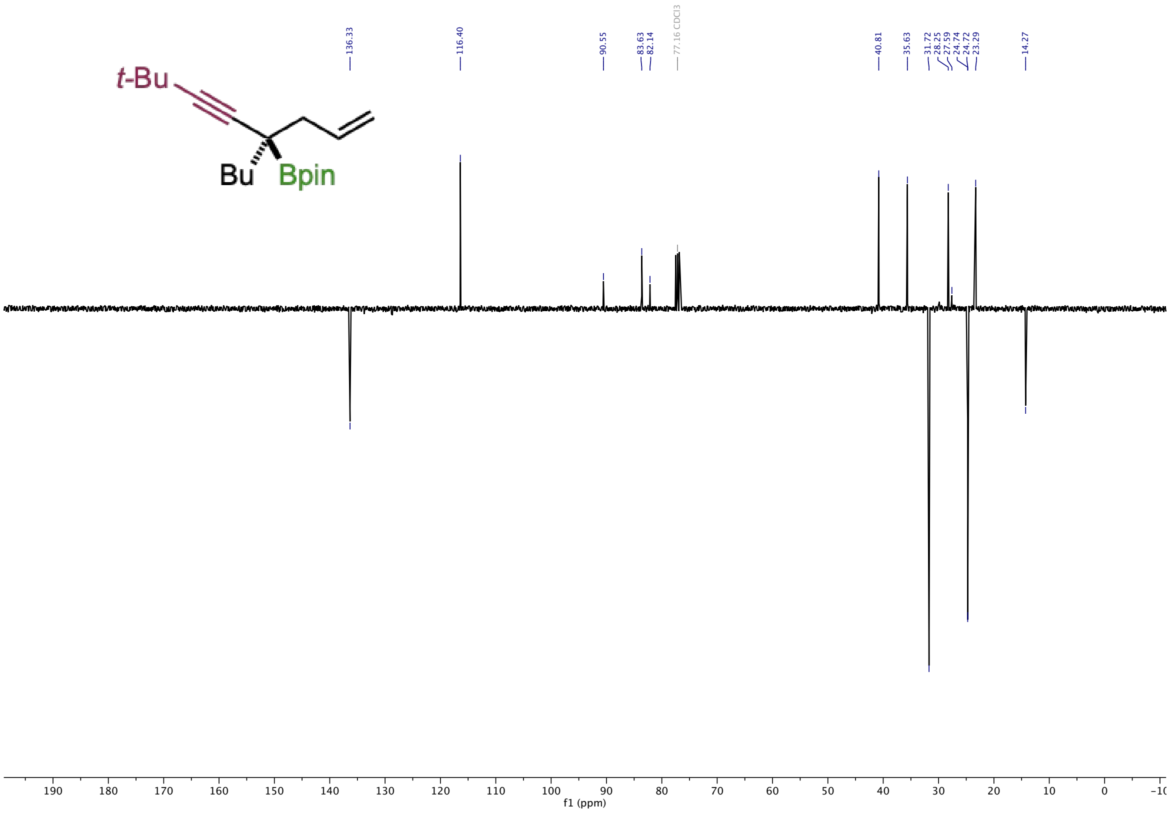

¹³C NMR (APT) spectrum (101 MHz, $CDCl_3$)

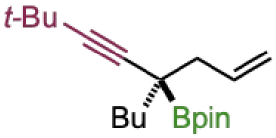

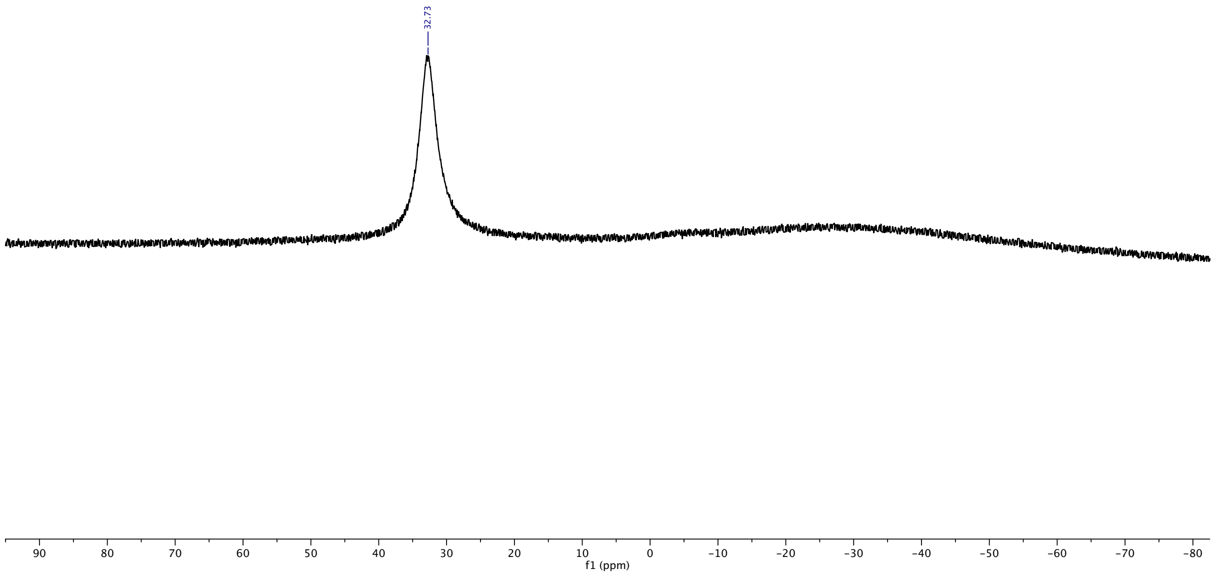

^{11}**B NMR** spectrum (128 MHz, $CDCl_3$)

2-(4-(Cyclopropylethynyl)oct-1-en-4-yl)-4,4,5,5-tetramethyl-1,3,2-dioxaborolane (6d)

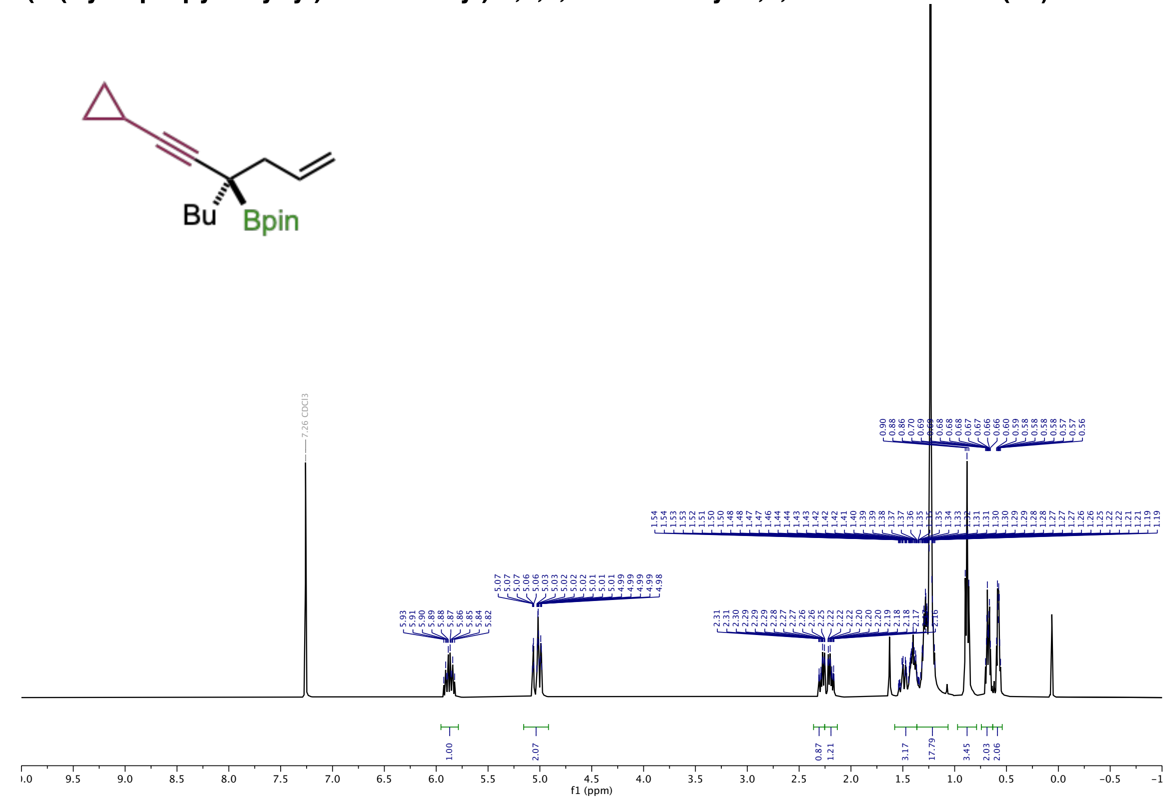

^{1}H NMR spectrum (400 MHz, $CDCl_3$)

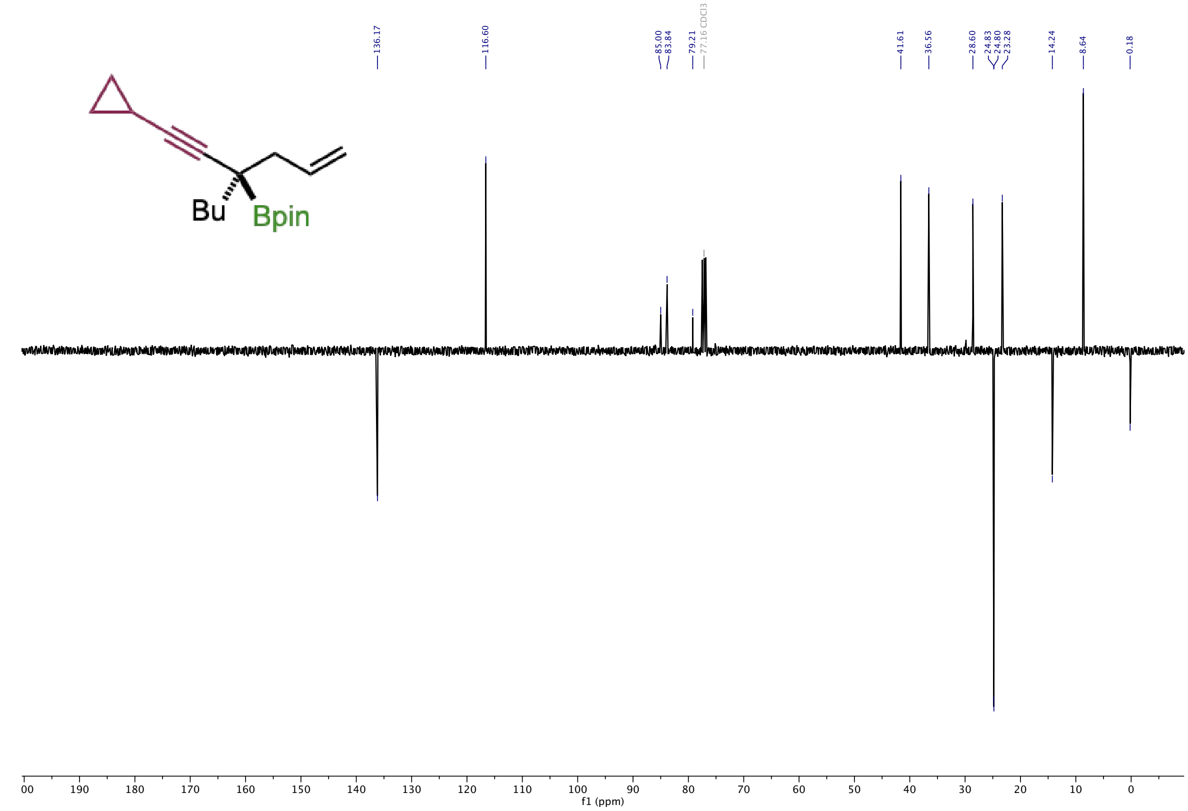

^{13}C NMR (APT) spectrum (101 MHz, $CDCl_3$)

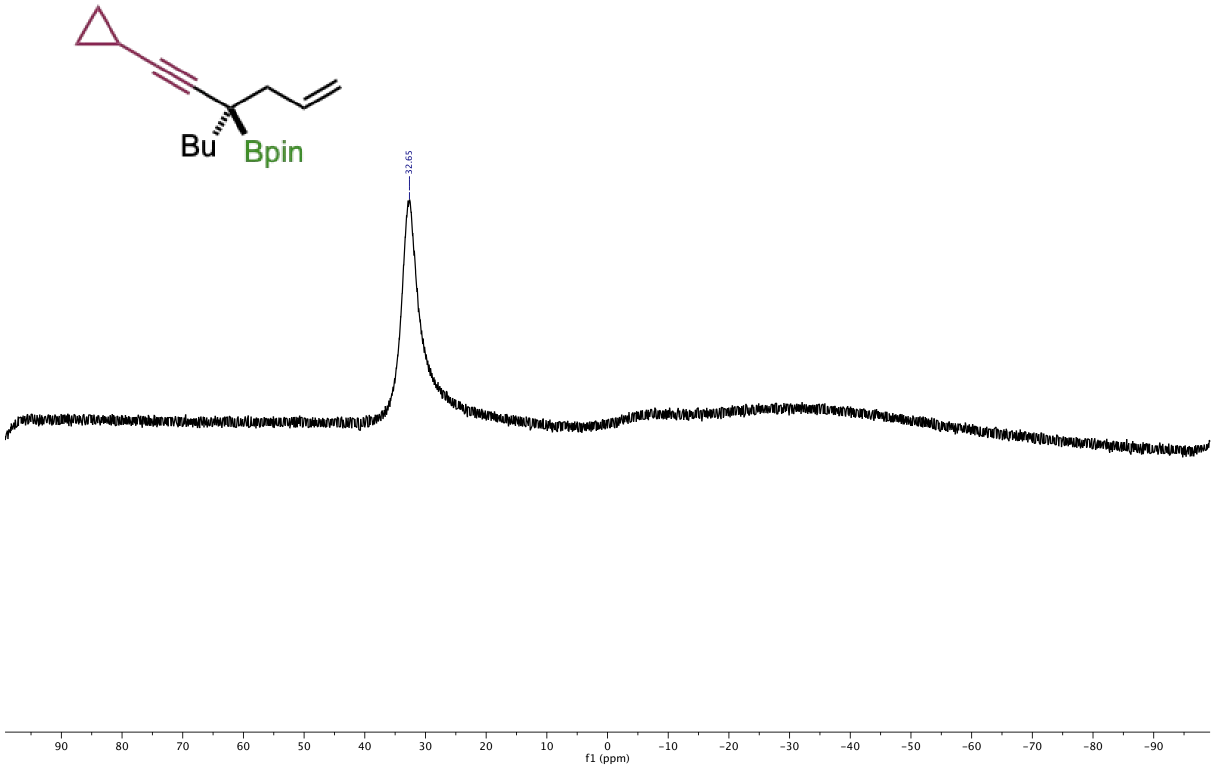

^{11}**B NMR** spectrum (128 MHz, $CDCl_3$)

2-(4-(Cyclohexylethynyl)oct-1-en-4-yl)-4,4,5,5-tetramethyl-1,3,2-dioxaborolane (6e)

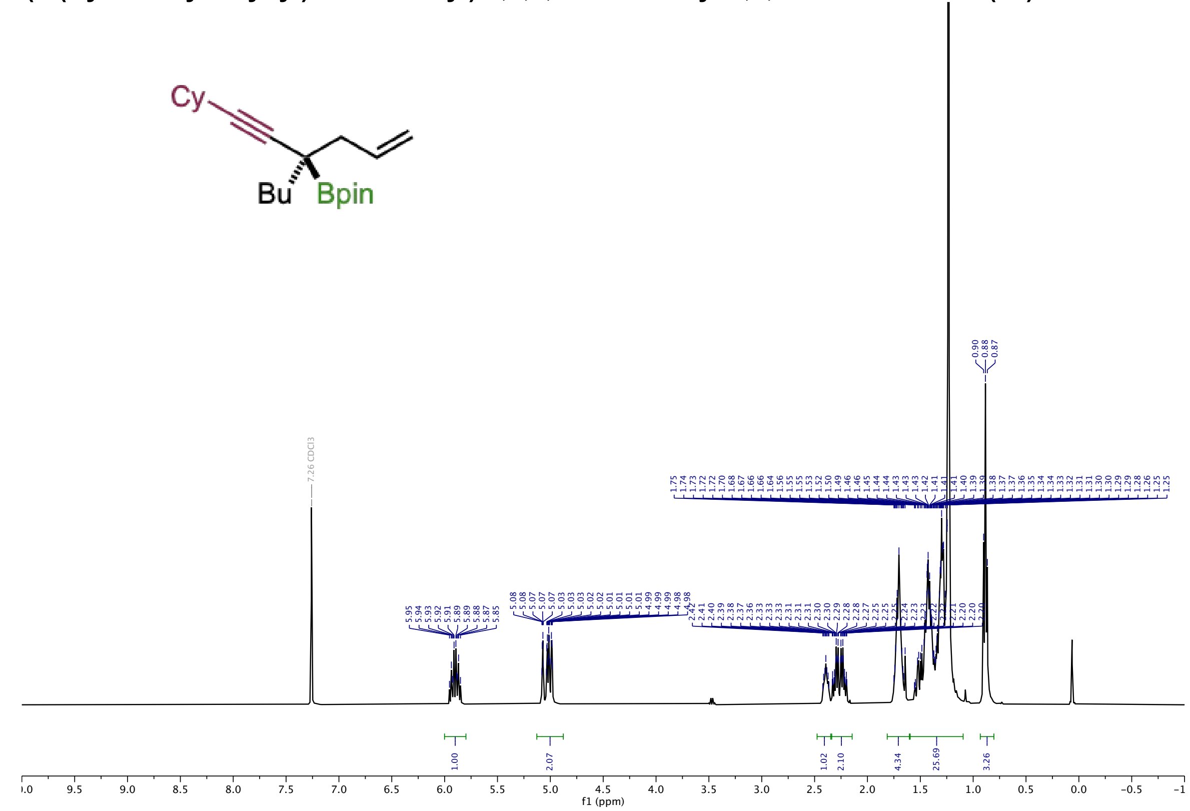

¹H NMR spectrum (400 MHz, $CDCl_3$)

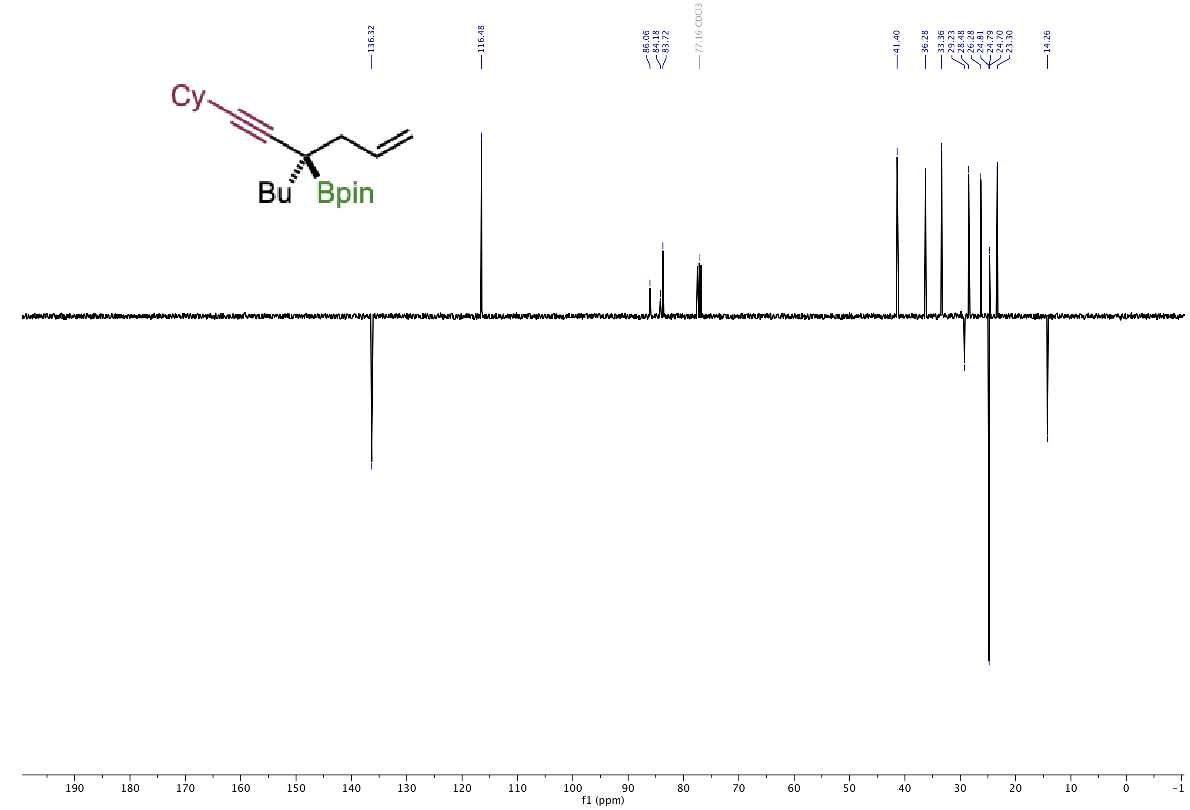

¹³C NMR (APT) spectrum (101 MHz, $CDCl_3$)

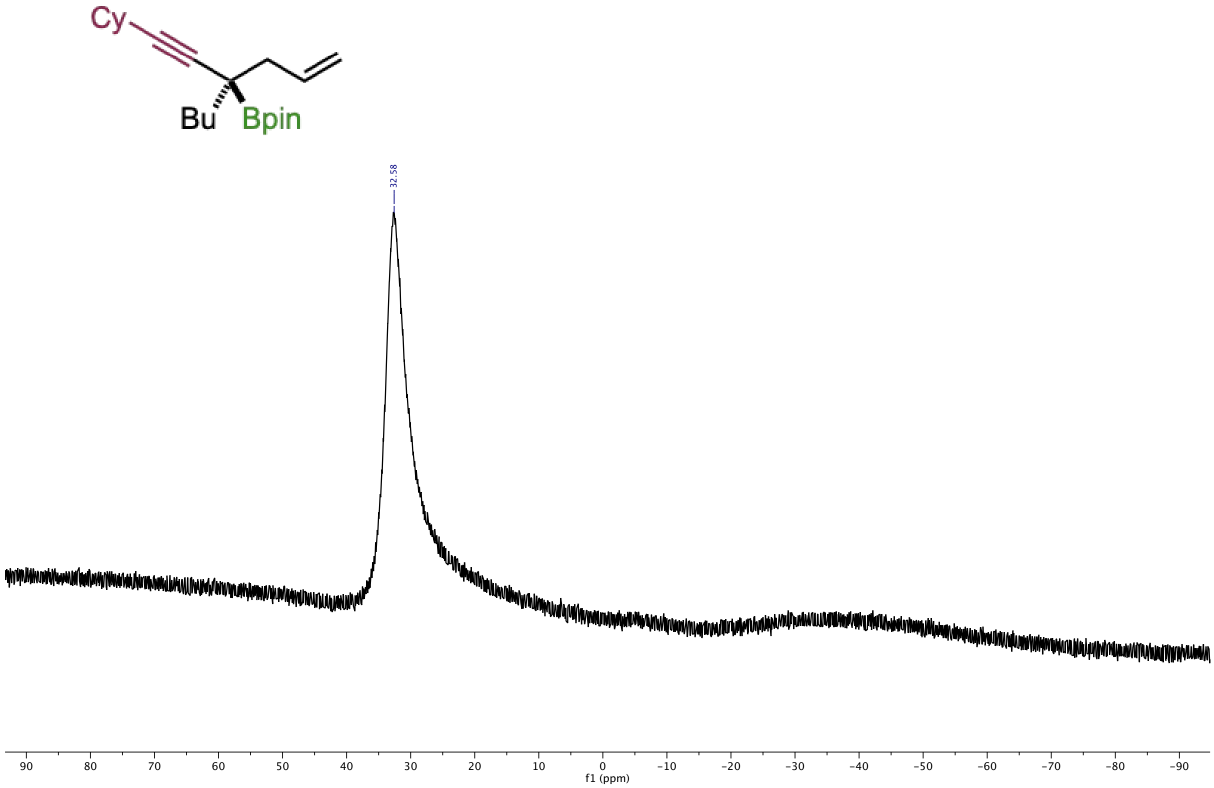

^{11}B NMR spectrum (128 MHz, $CDCl_3$)

2-(4-((*p*-Methoxyphenyl)ethynyl)oct-1-en-4-yl)-4,4,5,5-tetramethyl-1,3,2-dioxaborolane (6f)

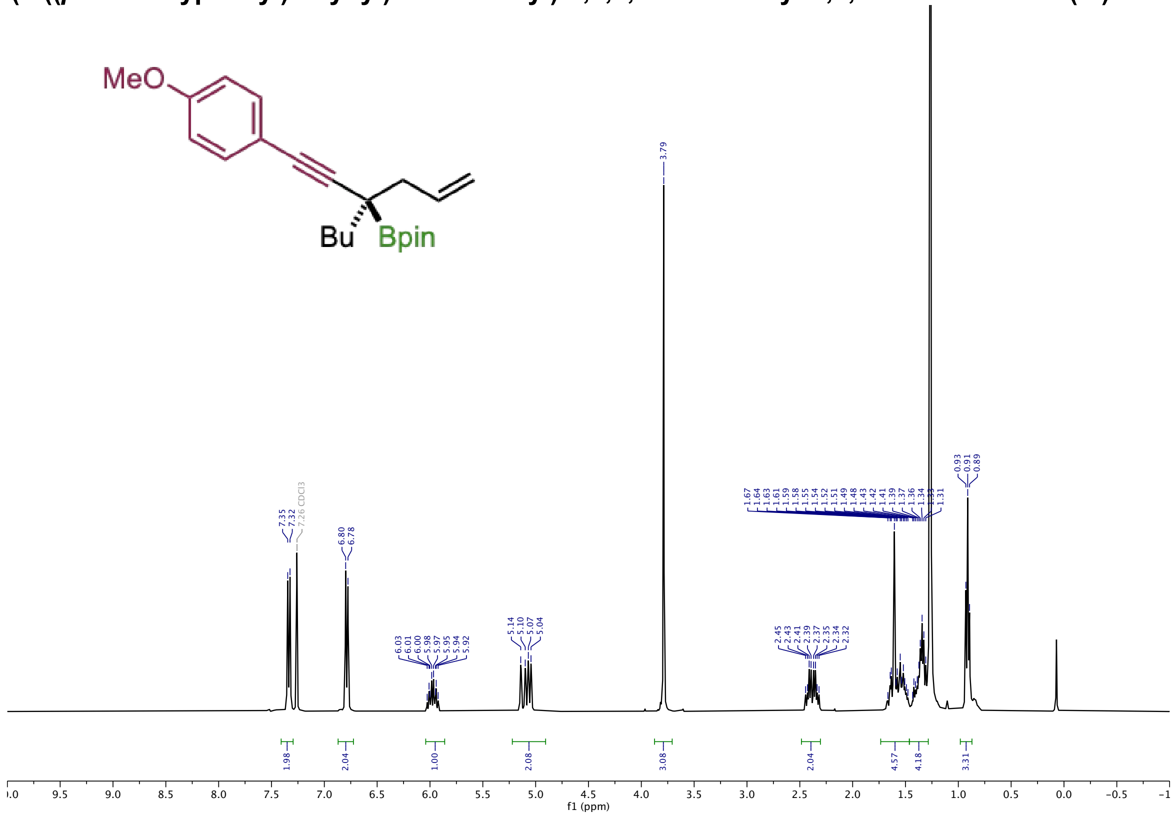

^{1}**H NMR** spectrum (400 MHz, $CDCl_3$)

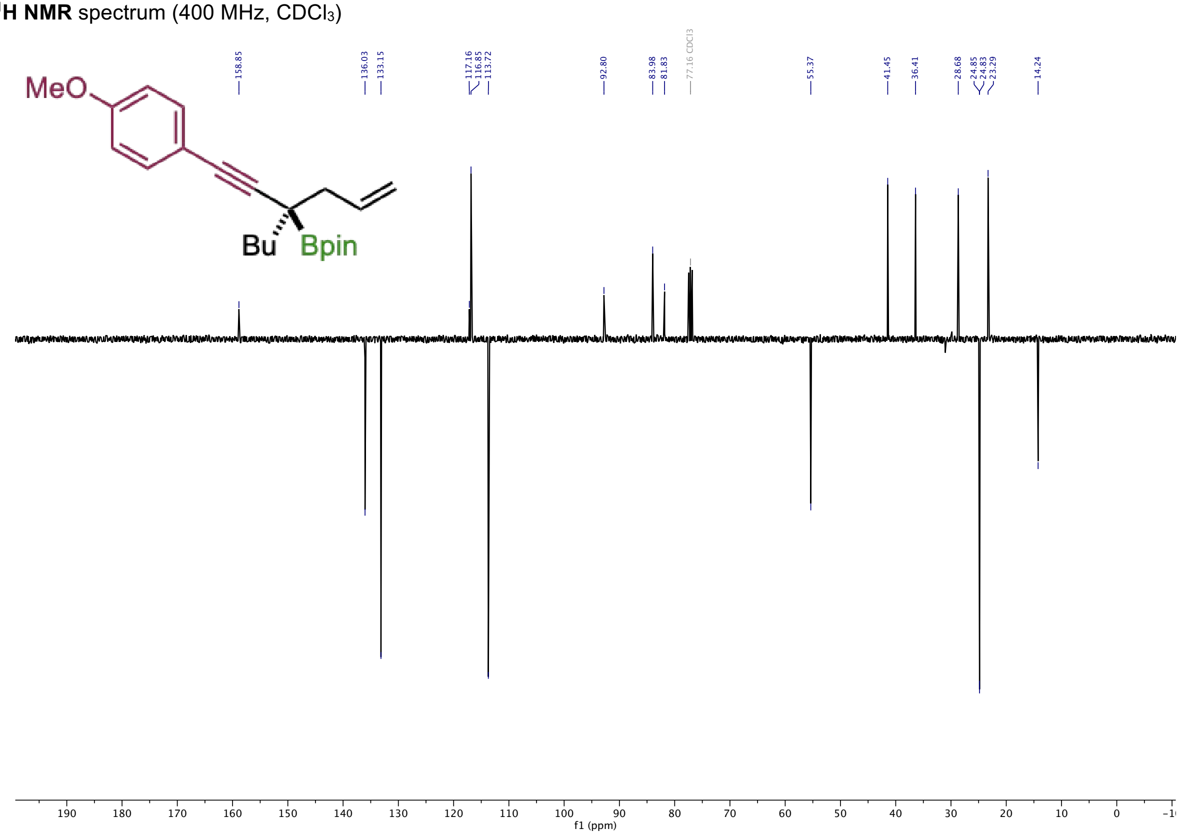

^{13}**C NMR** (APT) spectrum (101 MHz, $CDCl_3$)

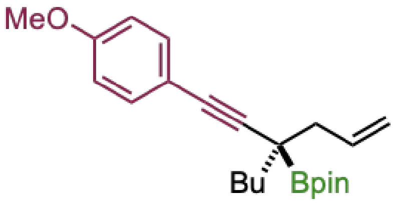

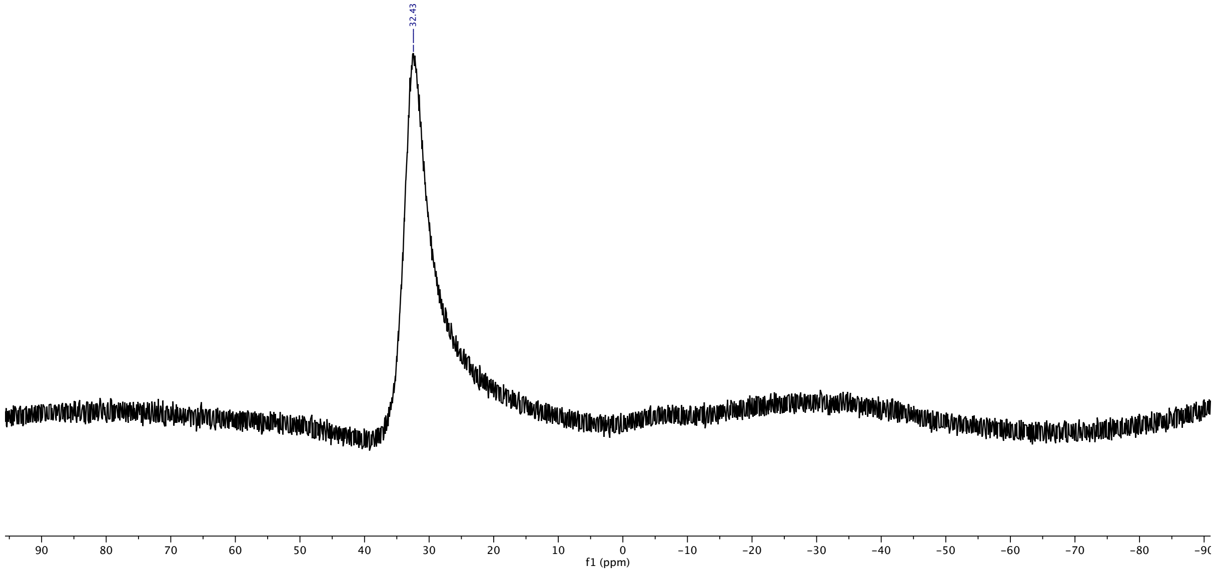

^{11}**B NMR** spectrum (128 MHz, $CDCl_3$)

4,4,5,5-Tetramethyl-2-(4-(*p*-tolylethynyl)oct-1-en-4-yl)-1,3,2-dioxaborolane (6g)

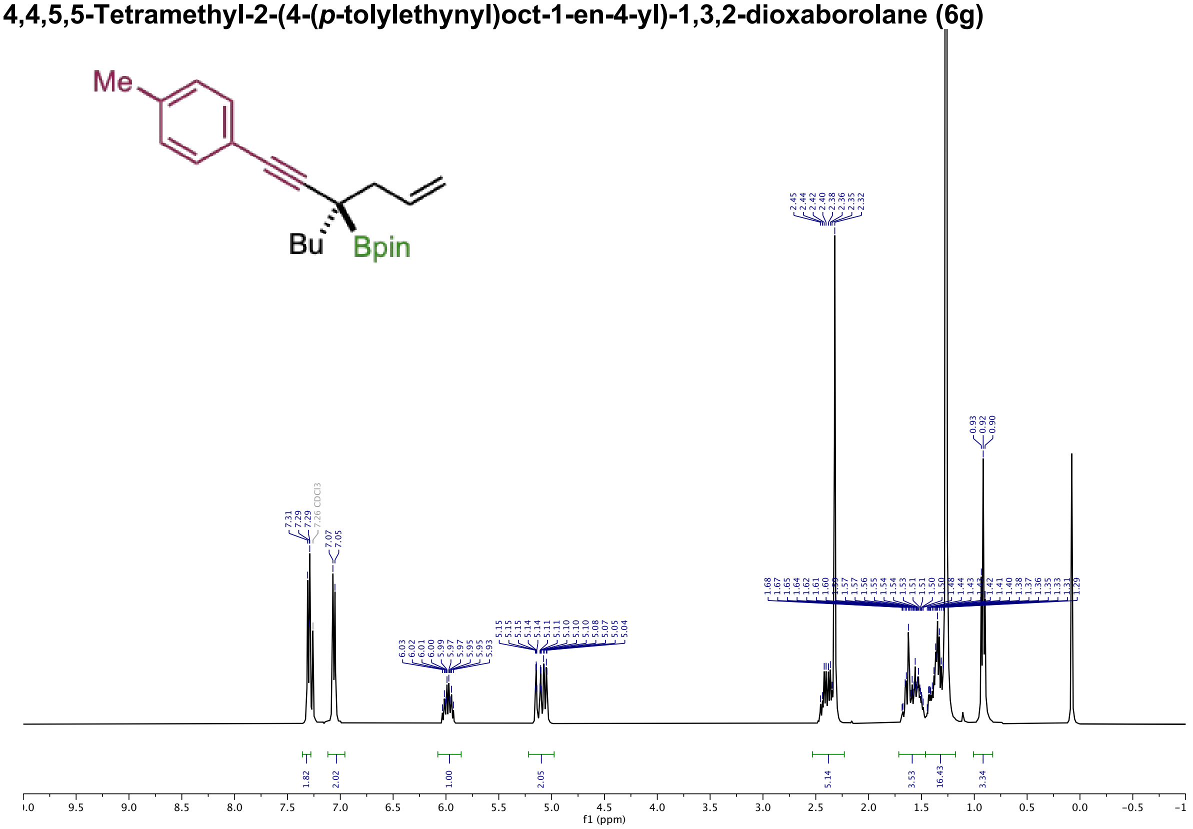

[1]**H NMR** spectrum (400 MHz, $CDCl_3$)

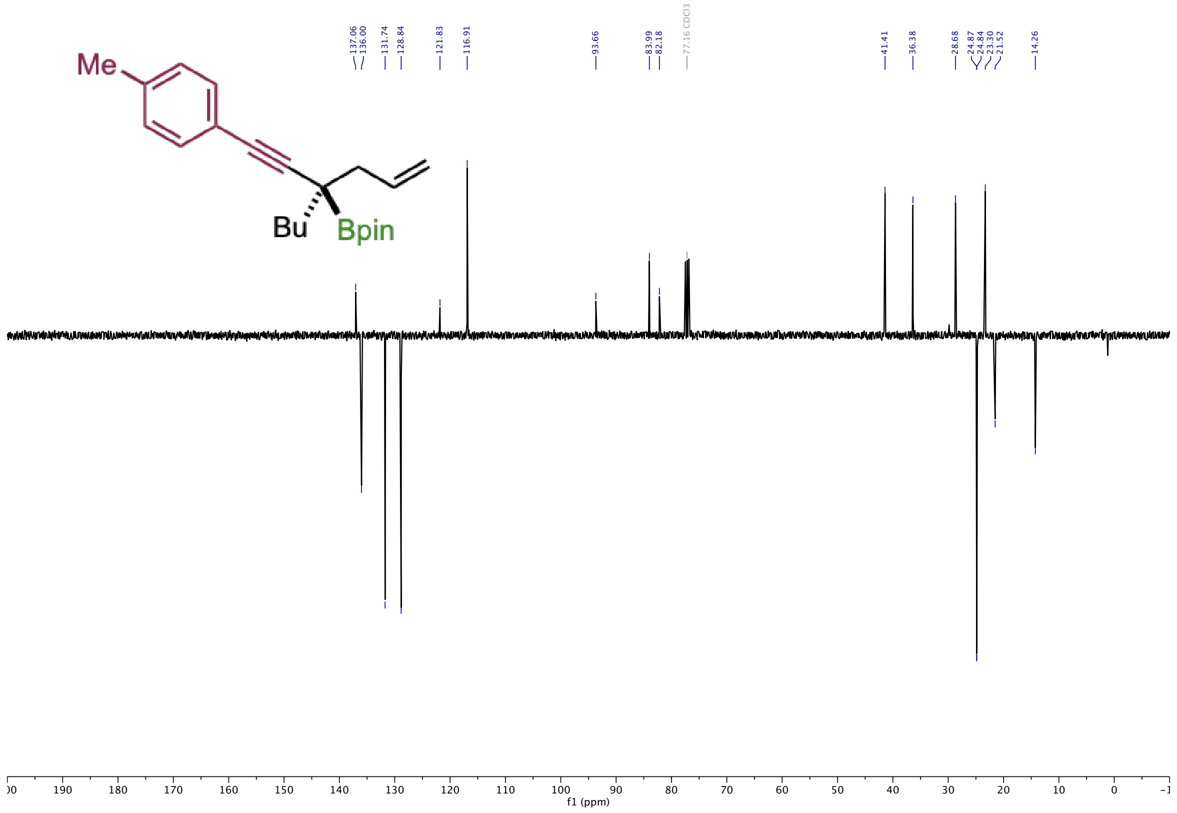

[13]**C NMR** (APT) spectrum (101 MHz, $CDCl_3$)

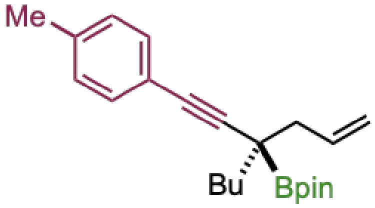

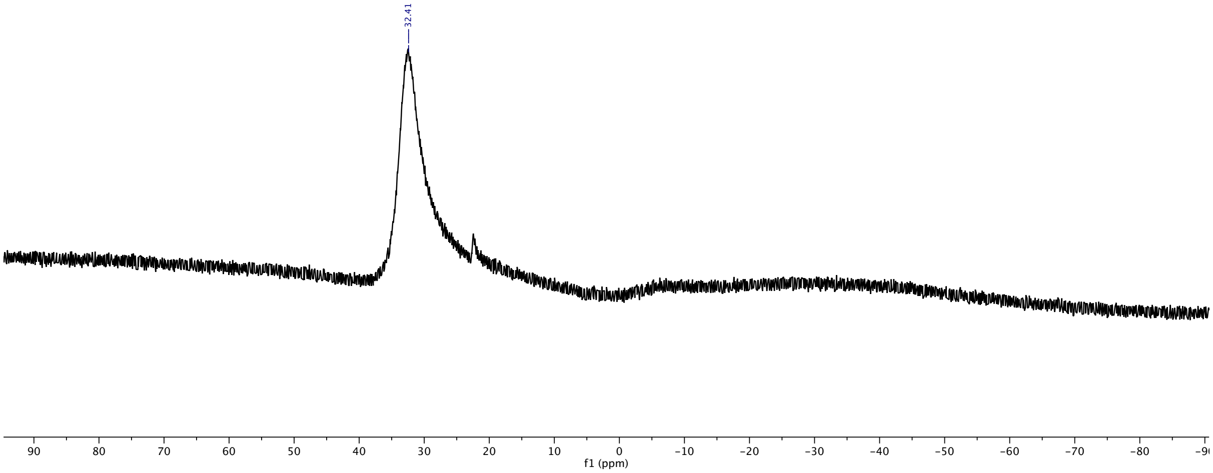

^{11}B NMR spectrum (128 MHz, $CDCl_3$)

4,4,5,5-Tetramethyl-2-(4-((trimethylsilyl)ethynyl)oct-1-en-4-yl)-1,3,2-dioxaborolane (6h)

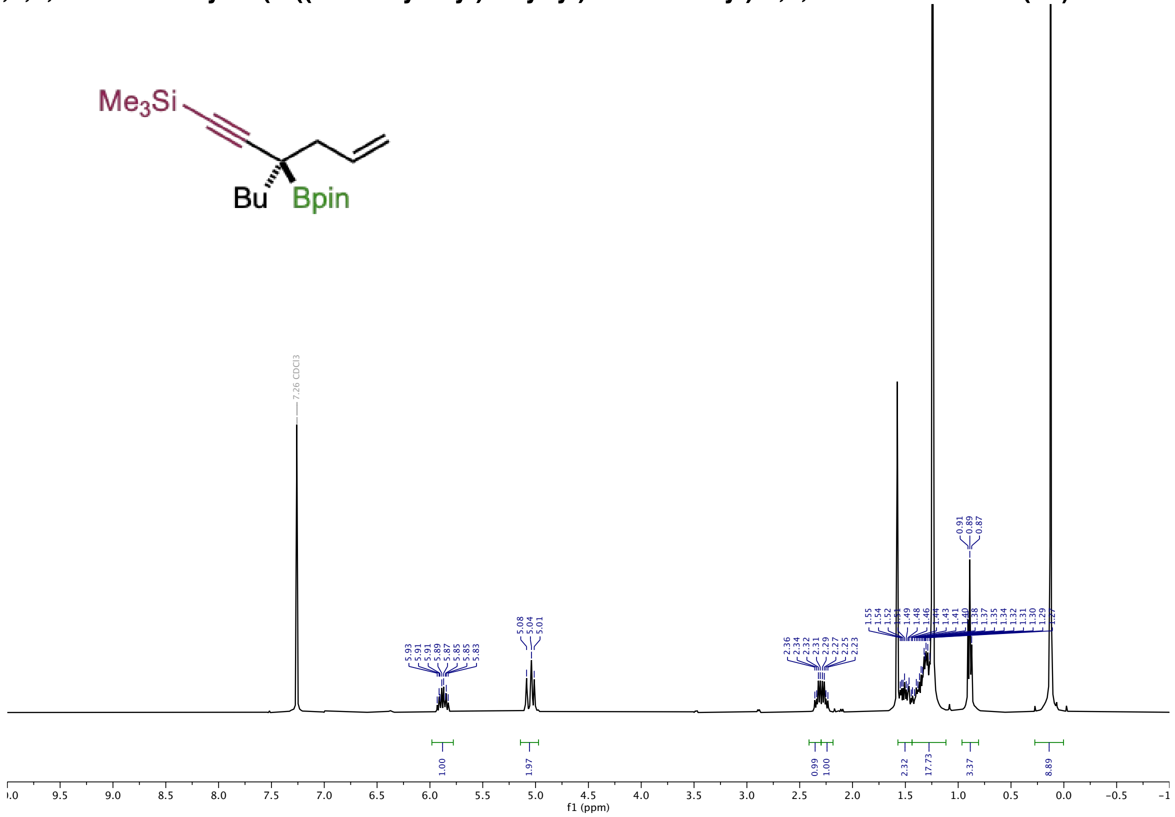

^{1}H NMR spectrum (400 MHz, $CDCl_3$)

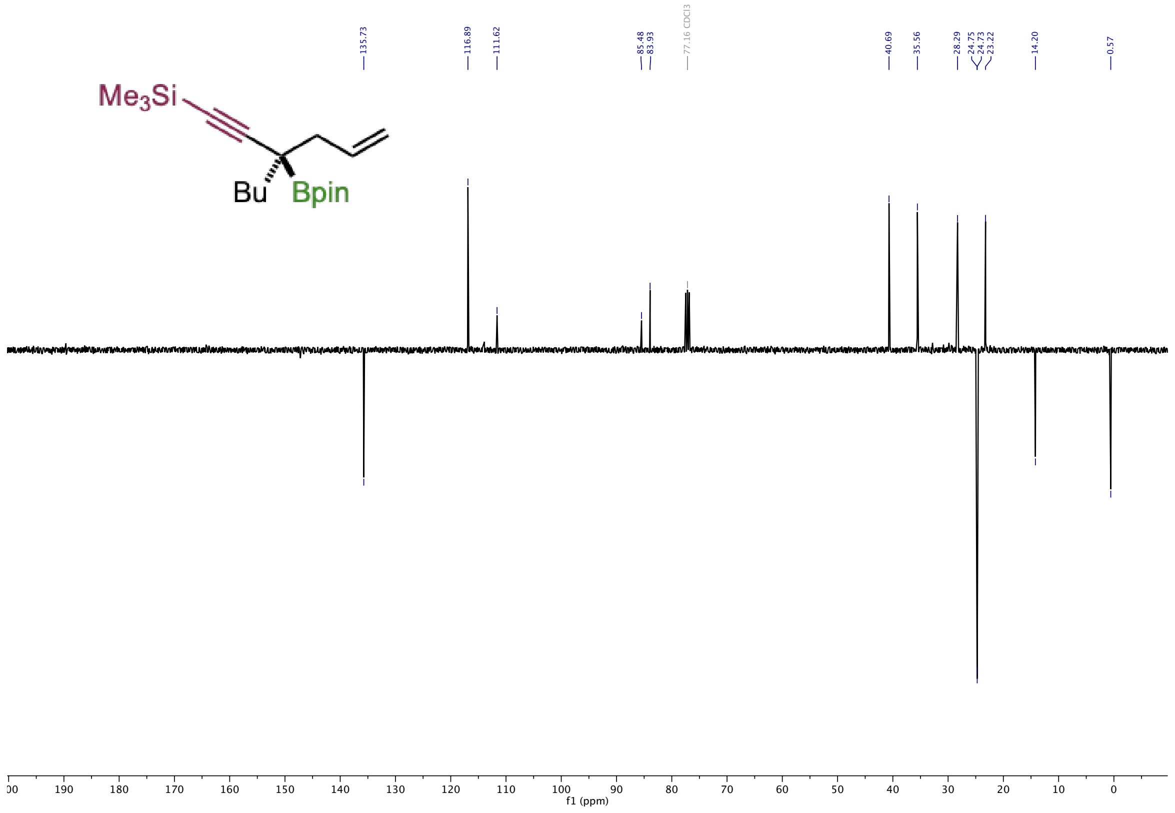

^{13}C NMR (APT) spectrum (101 MHz, $CDCl_3$)

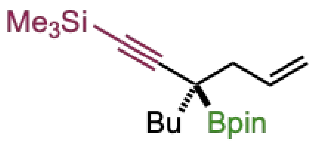

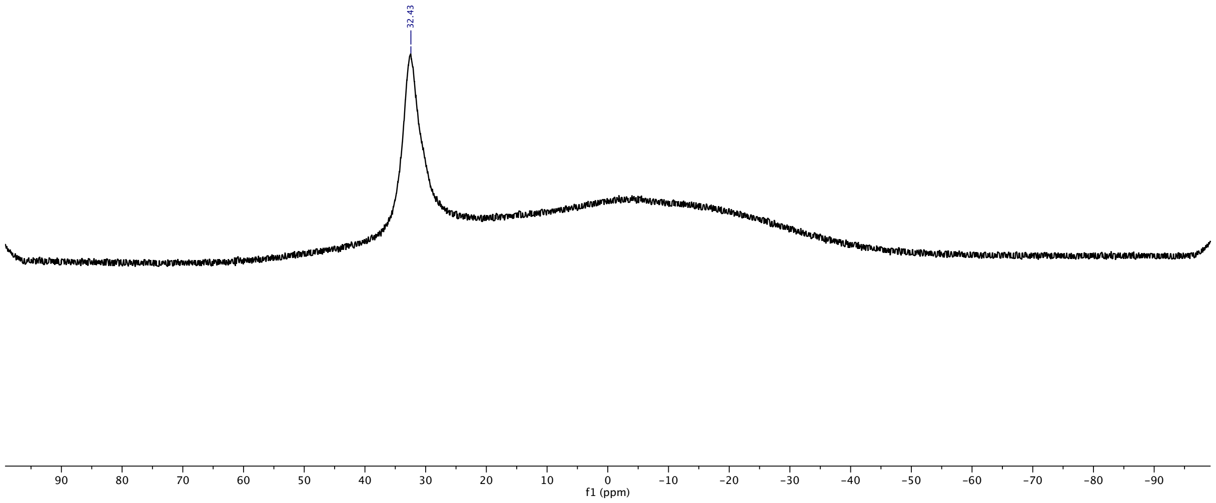

^{11}**B NMR** spectrum (128 MHz, $CDCl_3$)

4,4,5,5-Tetramethyl-2-(4-((triisopropylsilyl)ethynyl)oct-1-en-4-yl)-1,3,2-dioxaborolane (6i)

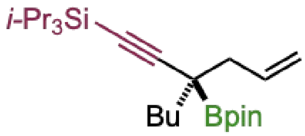

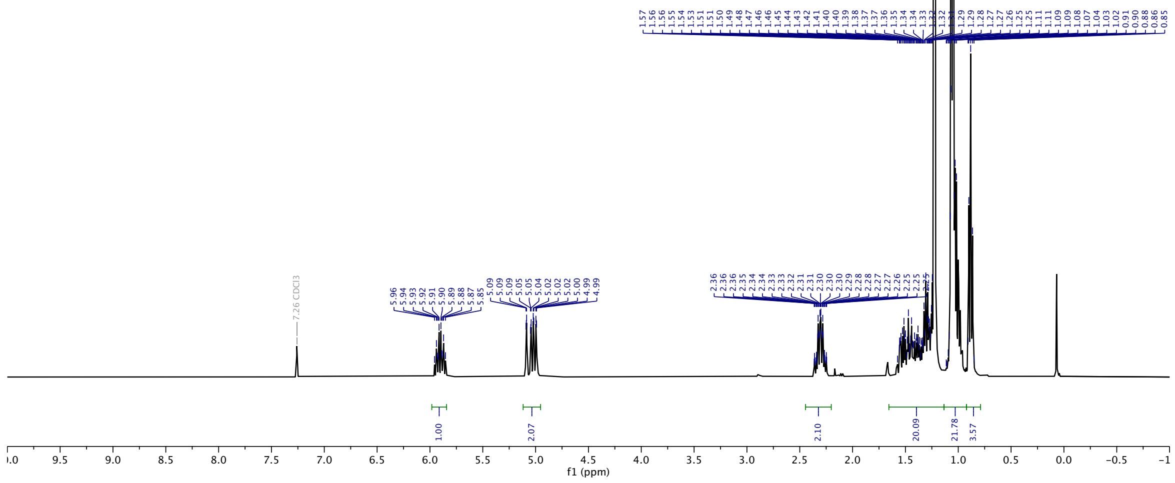

^{1}H NMR spectrum (400 MHz, $CDCl_3$)

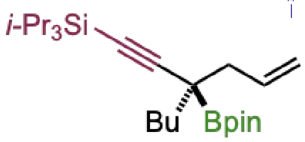

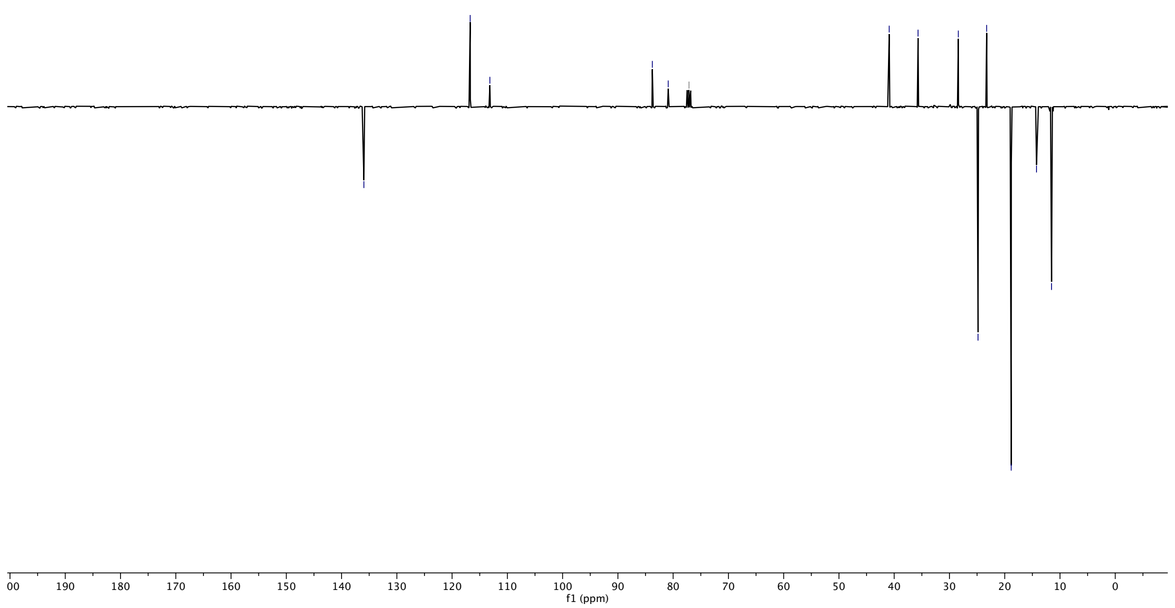

^{13}C NMR (APT) spectrum (101 MHz, $CDCl_3$)

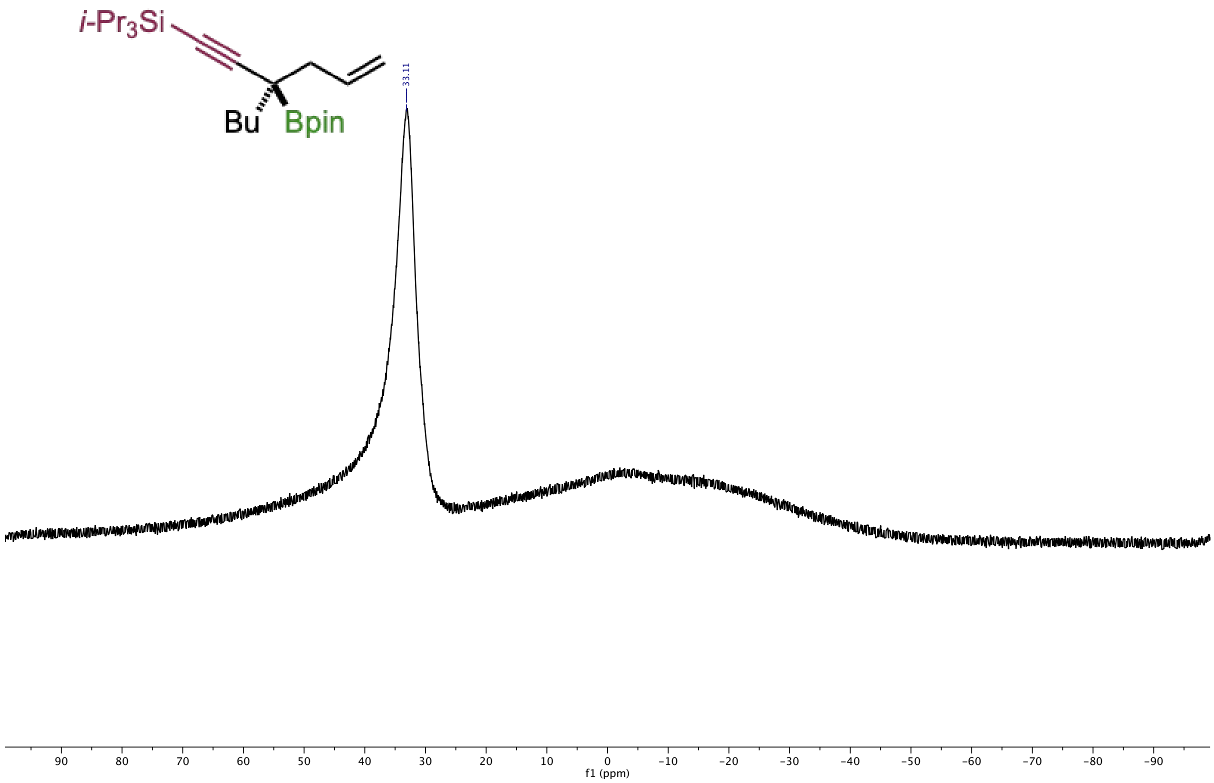

^{11}B NMR spectrum (128 MHz, $CDCl_3$)

2-(5-Allyl-10-(triisopropyl)oxydec-6-yn-5-yl)-4,4,5,5-tetramethyl-1,3,2-dioxaborolane (6j)

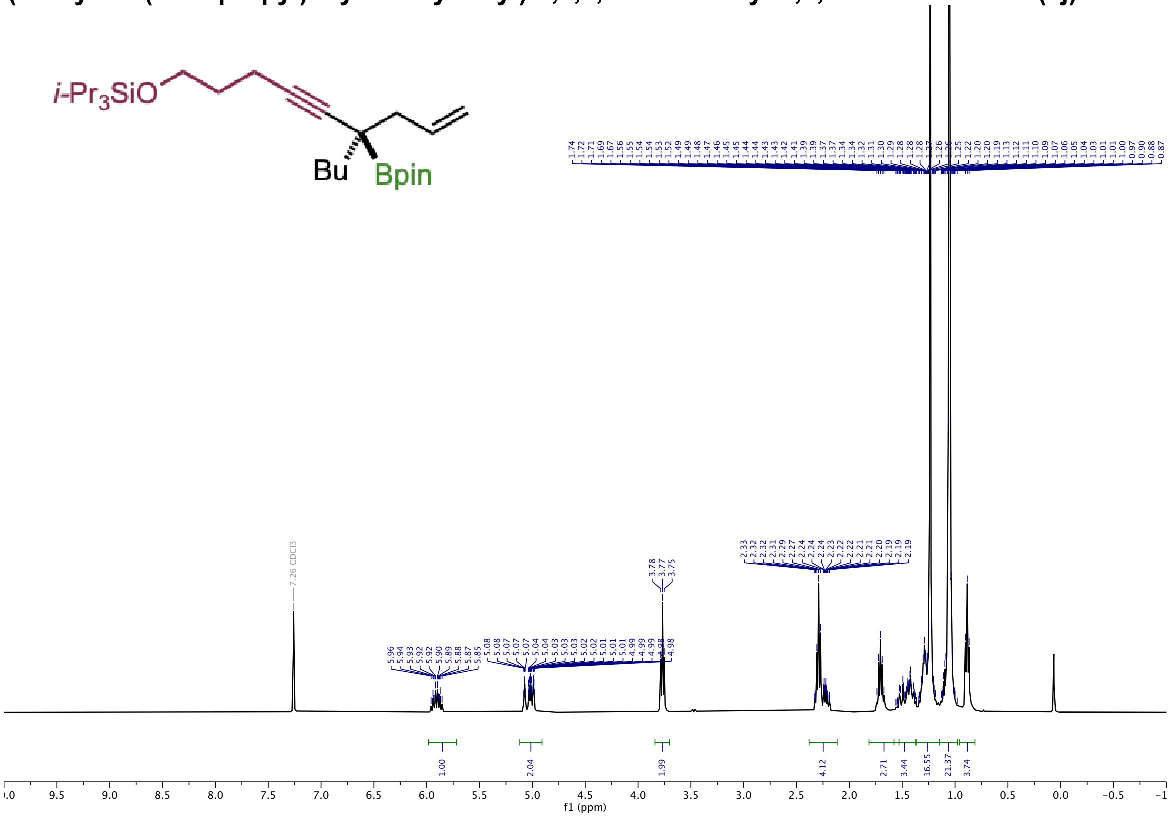

^{1}**H NMR** spectrum (400 MHz, $CDCl_3$)

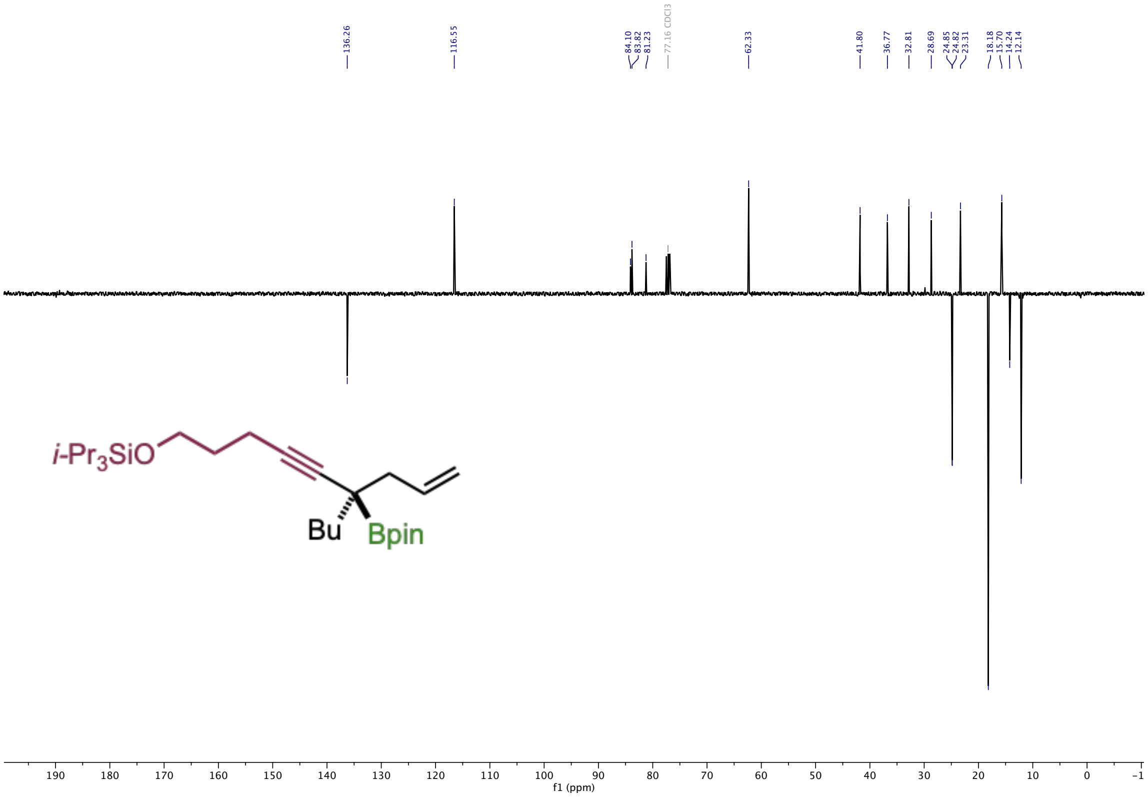

^{13}**C NMR** (APT) spectrum (101 MHz, $CDCl_3$)

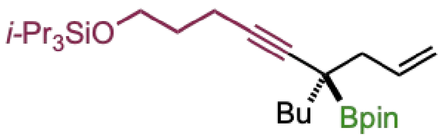

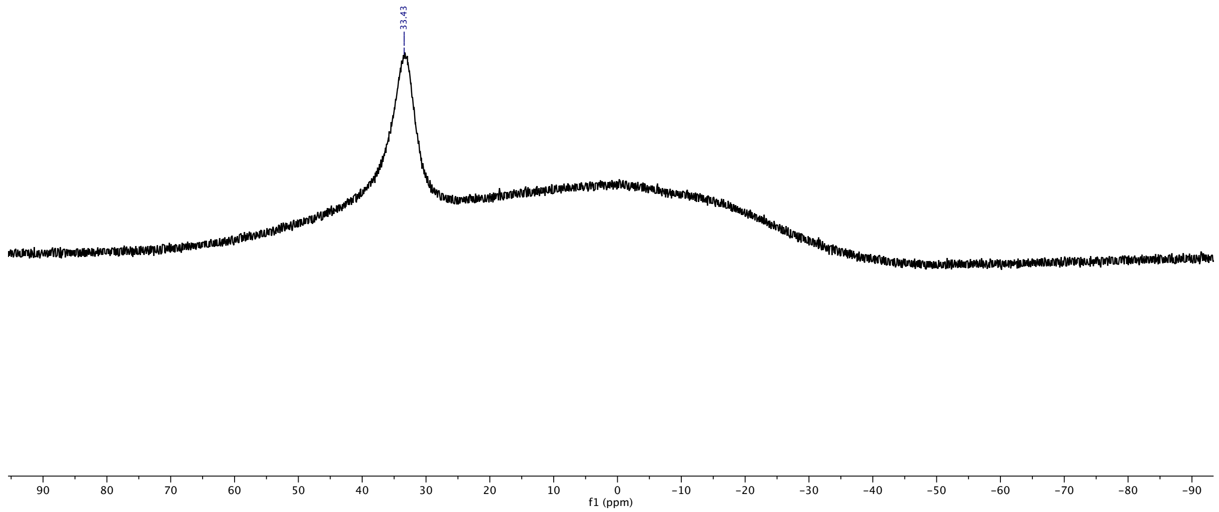

^{11}B NMR spectrum (128 MHz, $CDCl_3$)

2-(5-Allyl-10-(triisopropyl)oxyoct-6-yn-5-yl)-4,4,5,5-tetramethyl-1,3,2-dioxaborolane (6k)

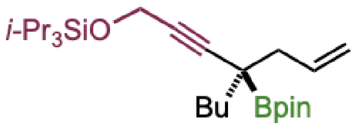

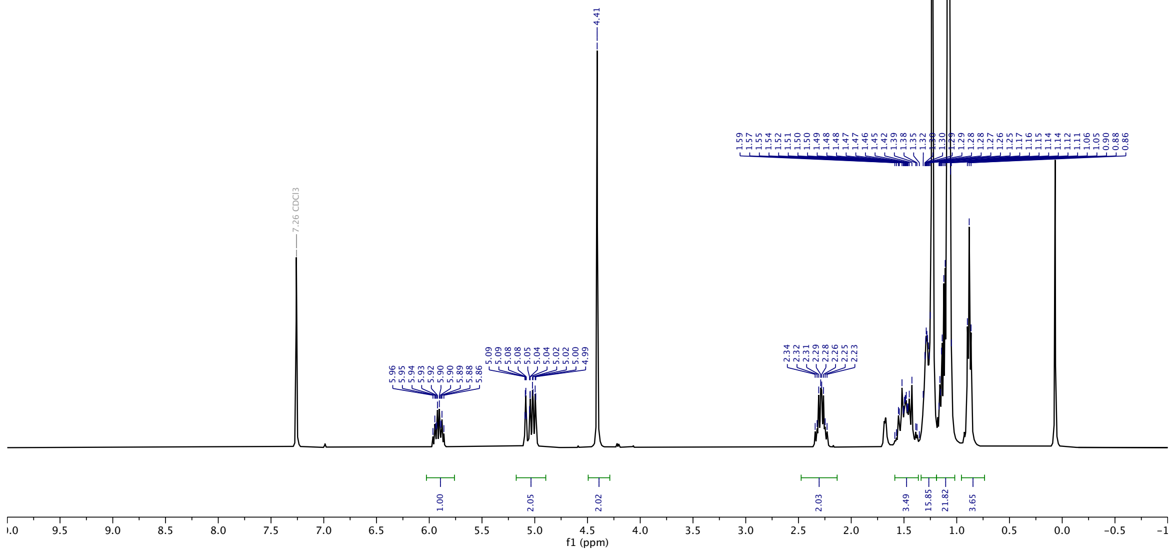

^{1}H NMR spectrum (400 MHz, $CDCl_3$)

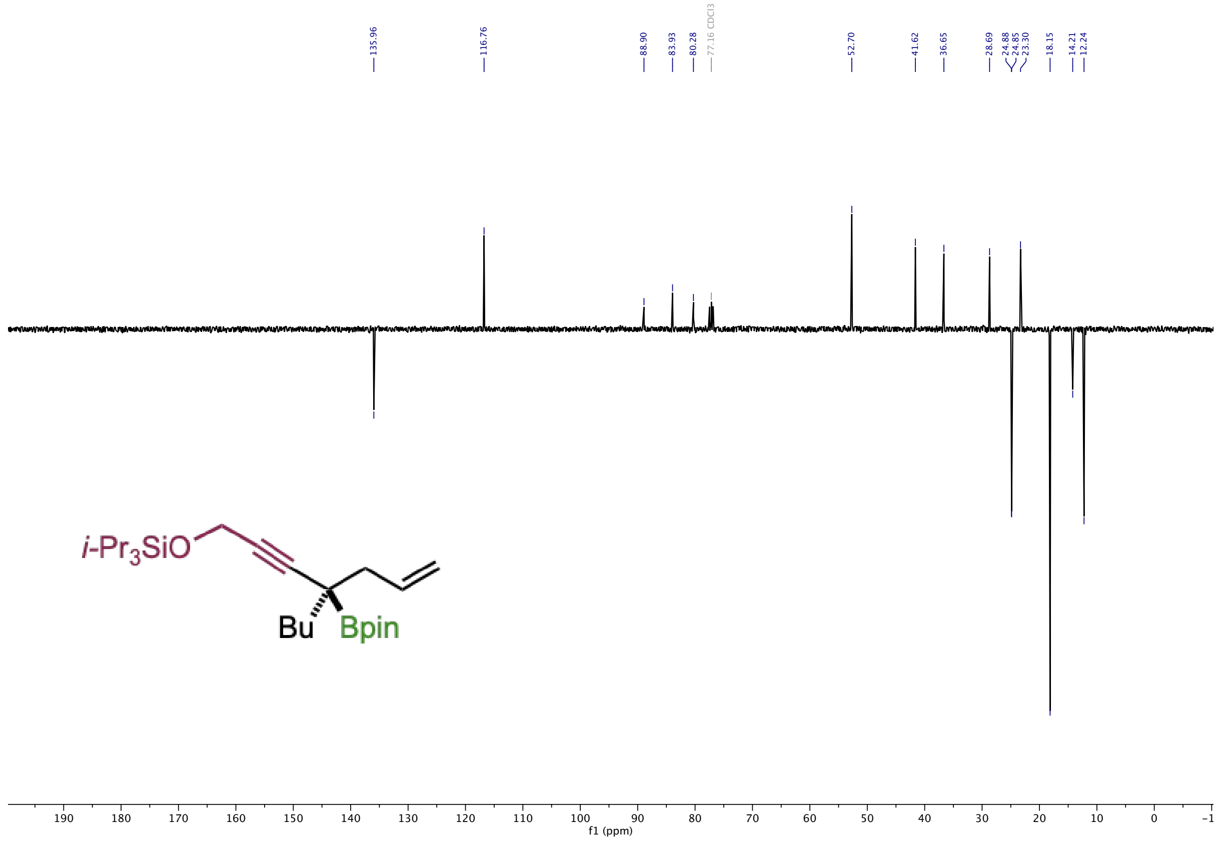

^{13}C NMR (APT) spectrum (101 MHz, $CDCl_3$)

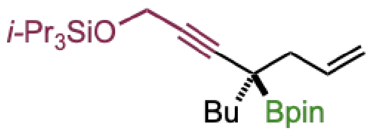

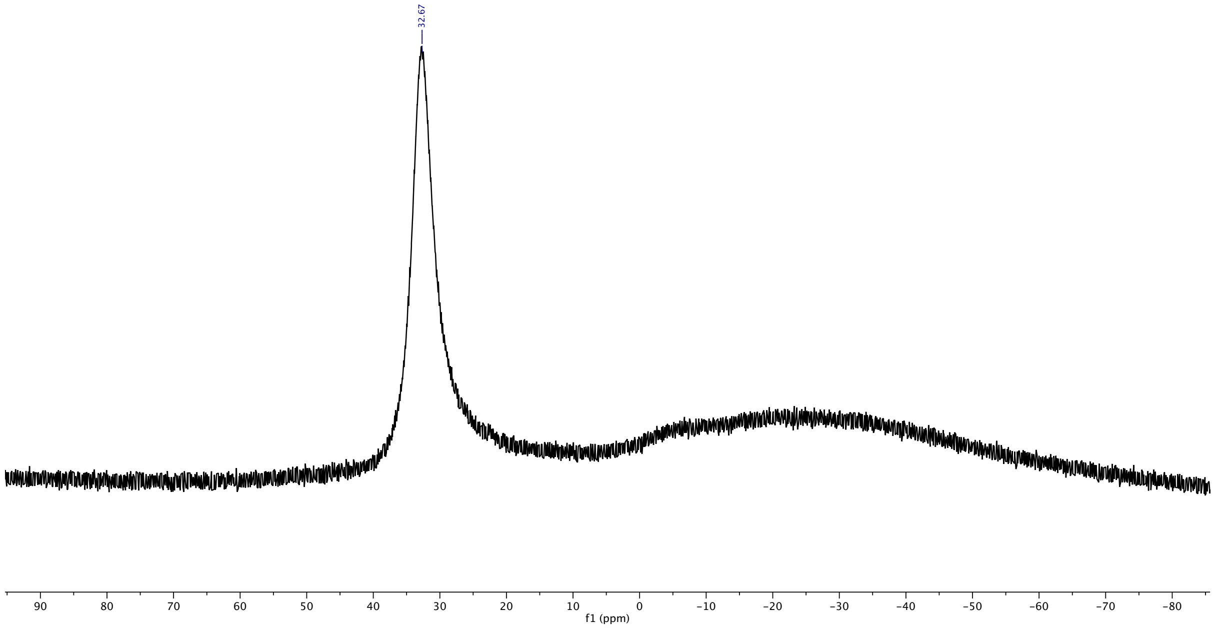

^{11}B NMR spectrum (128 MHz, $CDCl_3$)

2-(5-Allyl-10-chlorodec-6-yn-5-yl)-4,4,5,5-tetramethyl-1,3,2-dioxaborolane (6l)

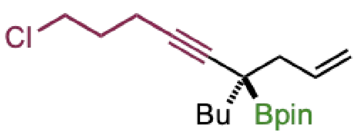

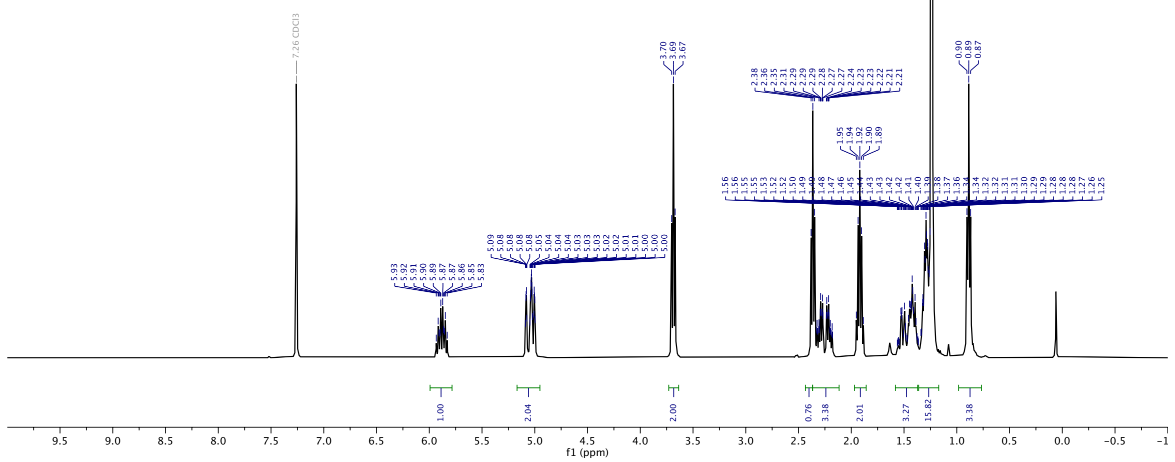

¹H NMR spectrum (400 MHz, $CDCl_3$)

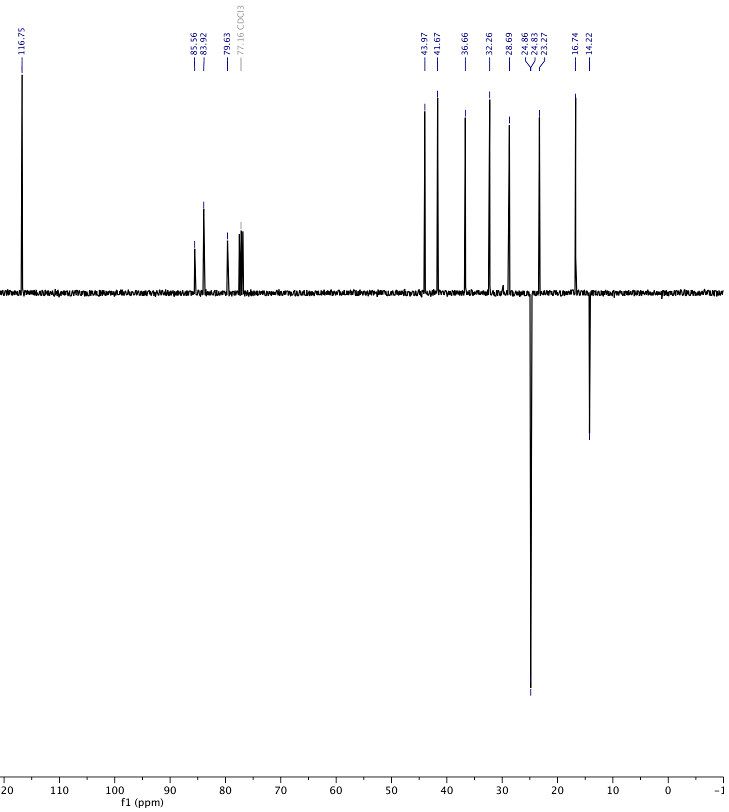

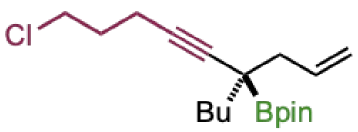

¹³C NMR (APT) spectrum (101 MHz, $CDCl_3$)

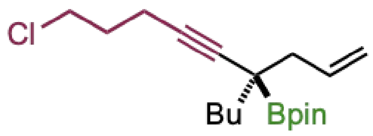

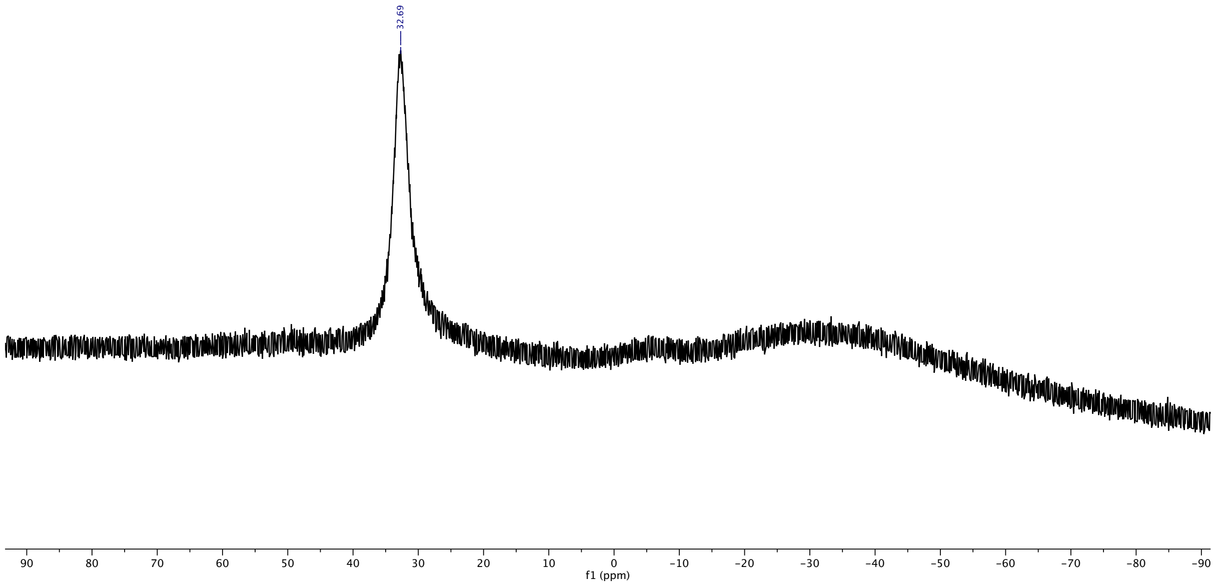

^{11}B NMR spectrum (128 MHz, $CDCl_3$)

2-(4-(3-Chloropropyl)dec-1-en-5-yn-4-yl)-4,4,5,5-tetramethyl-1,3,2-dioxaborolane (6m)

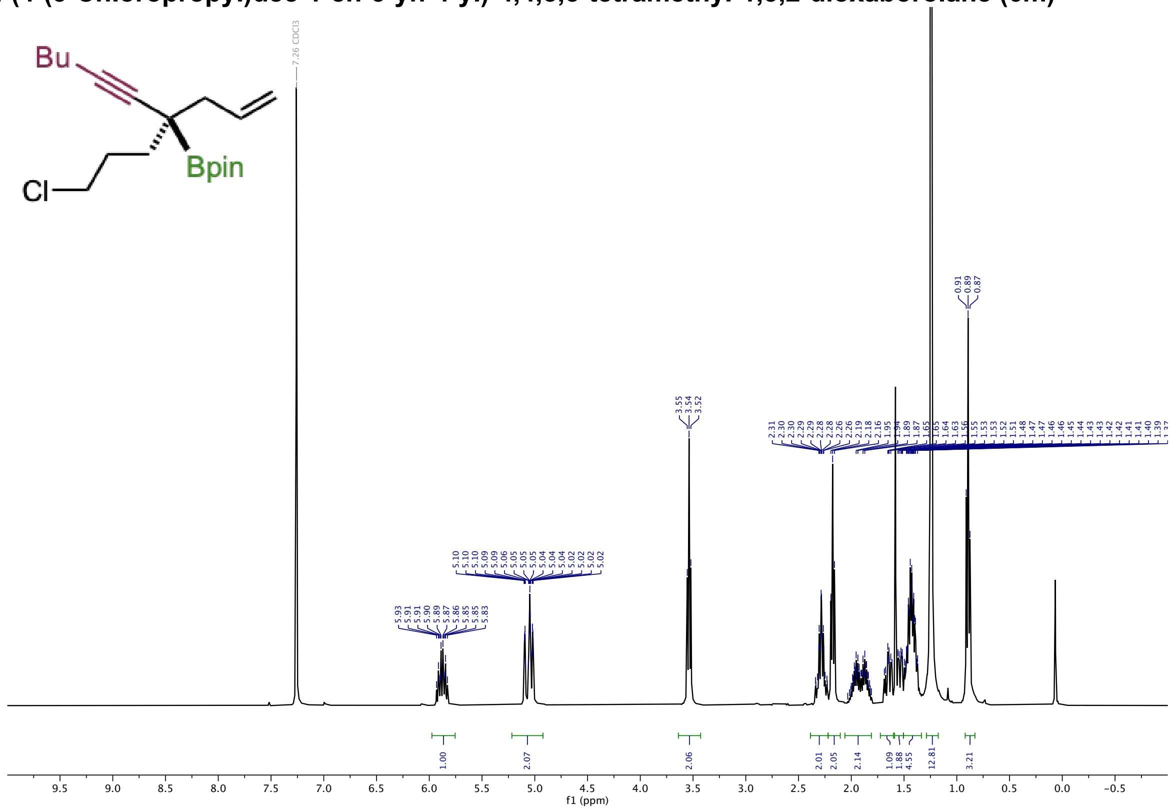

^{1}H NMR spectrum (400 MHz, $CDCl_3$)

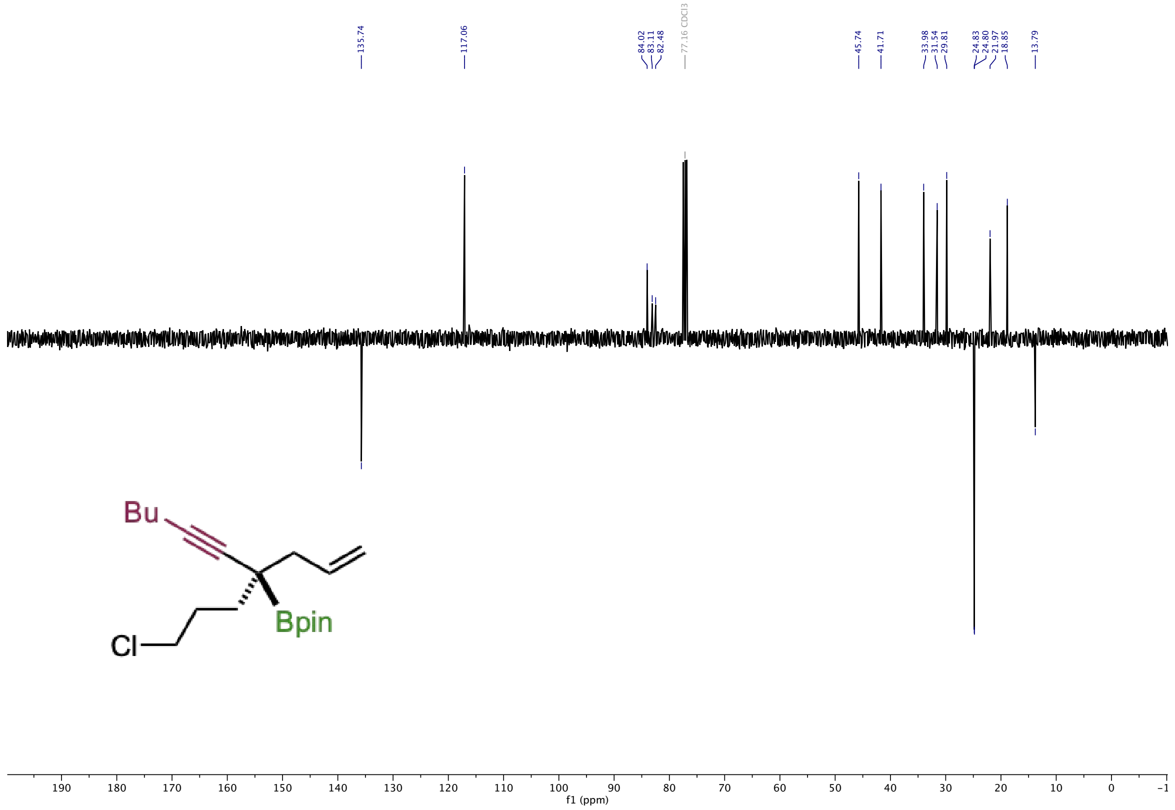

^{13}C NMR (APT) spectrum (101 MHz, $CDCl_3$)

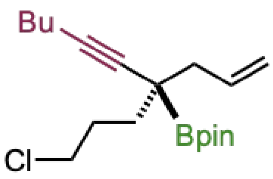

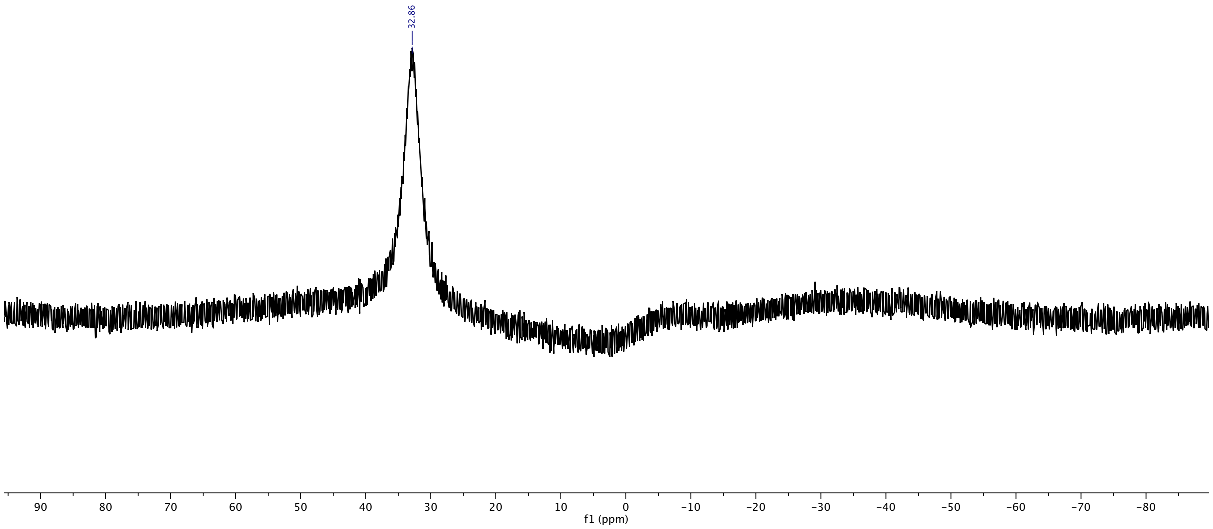

^{11}B NMR spectrum (128 MHz, $CDCl_3$)

2-((4*S**,5*S**)-5-Butyl-4-vinylundec-1-en-6-yn-5-yl)-4,4,5,5-tetramethyl-1,3,2-dioxaborolane (6n)

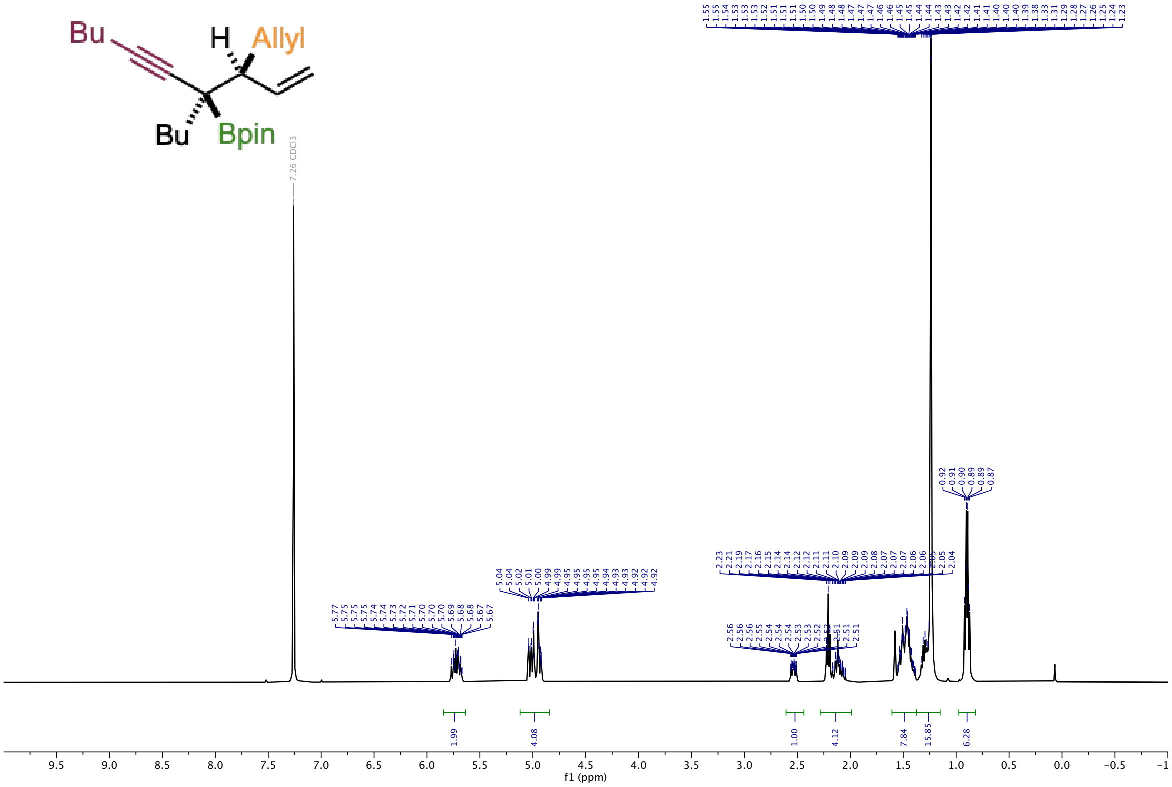

^{1}H NMR spectrum (400 MHz, $CDCl_3$)

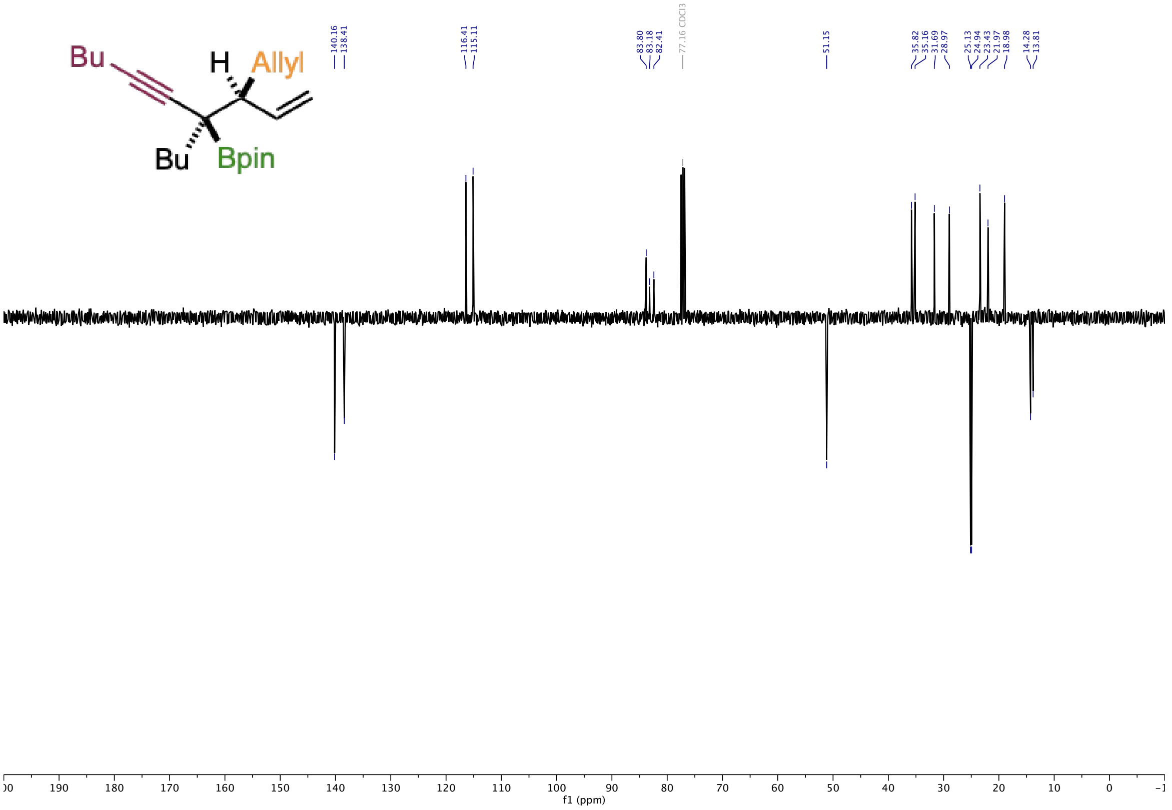

^{13}C NMR (APT) spectrum (101 MHz, $CDCl_3$)

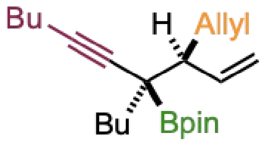

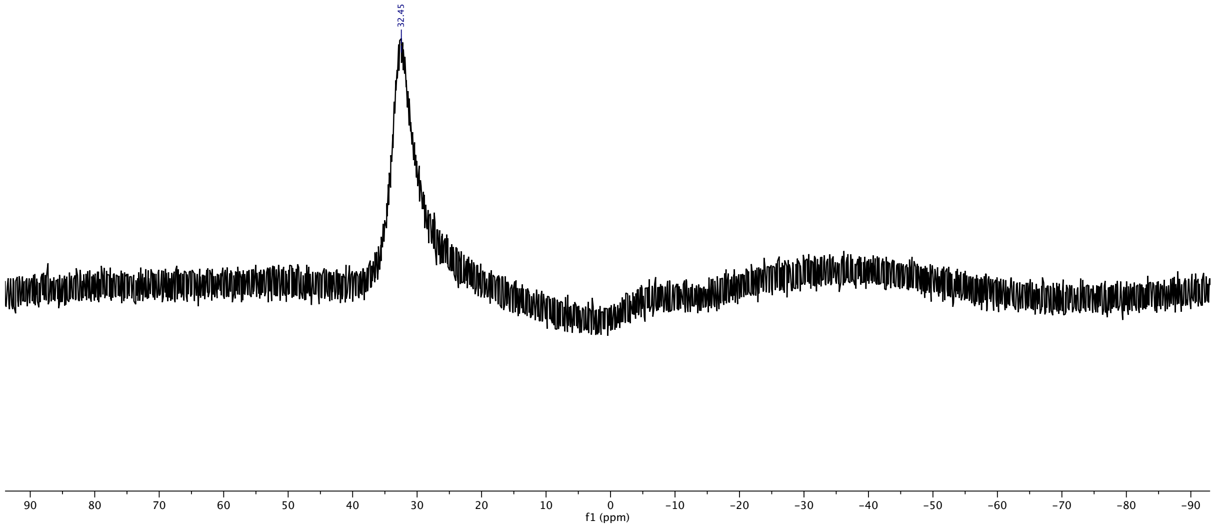

^{11}**B NMR** spectrum (128 MHz, $CDCl_3$)

2-((4*S,5*S**)-5-(cyclohexylethynyl)-4-vinylnon-1-en-5-yl)-4,4,5,5-tetramethyl-1,3,2-dioxaborolane (6o)**

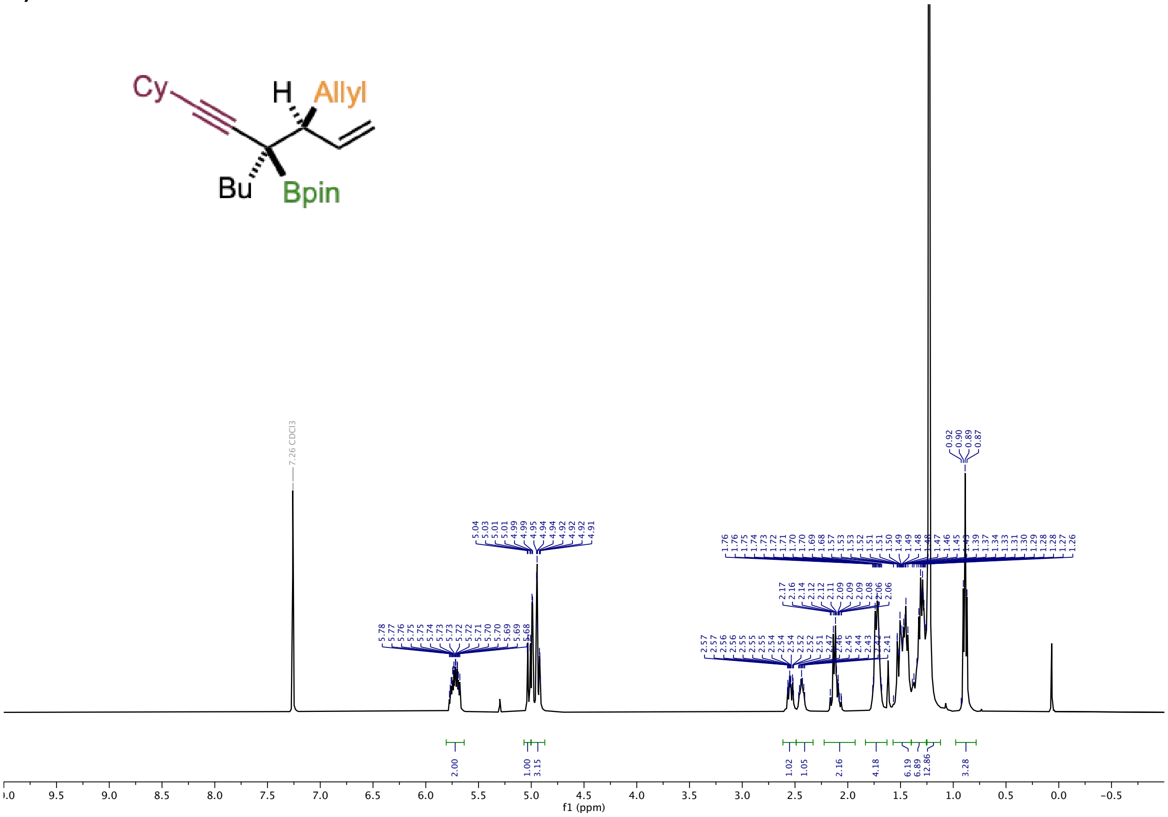

^{1}H NMR spectrum (400 MHz, CDCl$_3$)

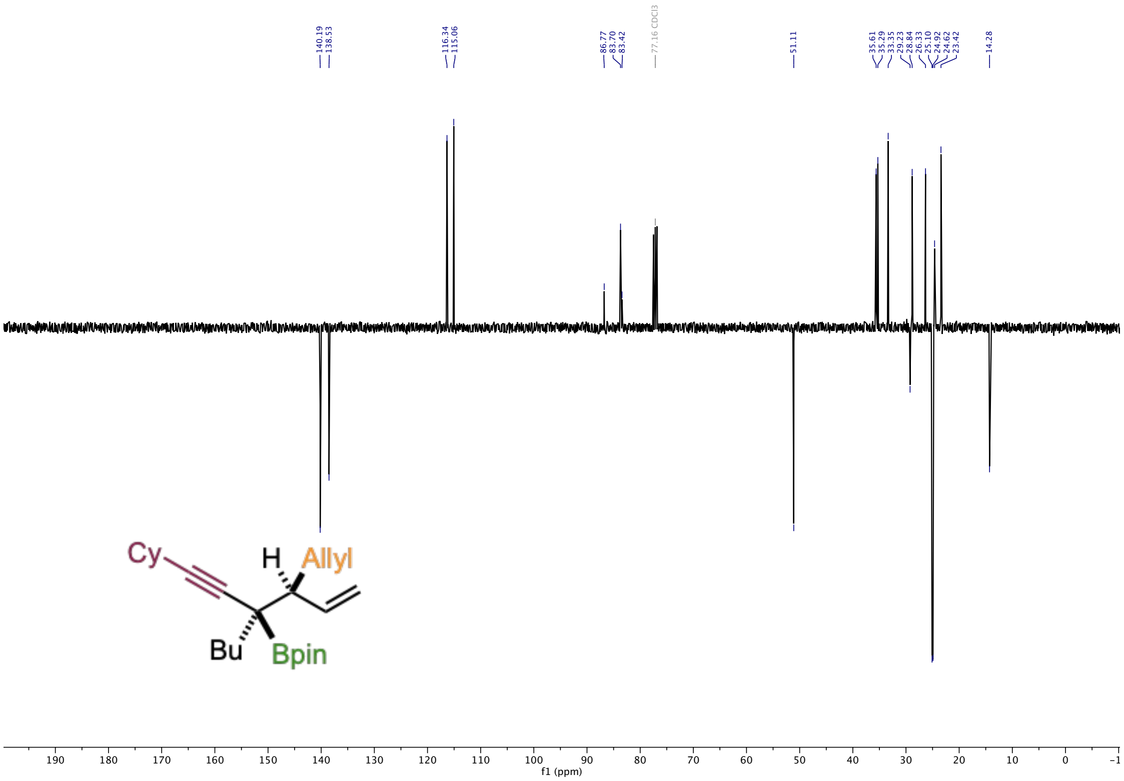

^{13}C NMR (APT) spectrum (101 MHz, CDCl$_3$)

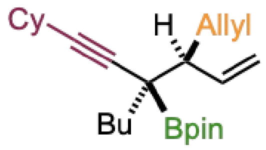

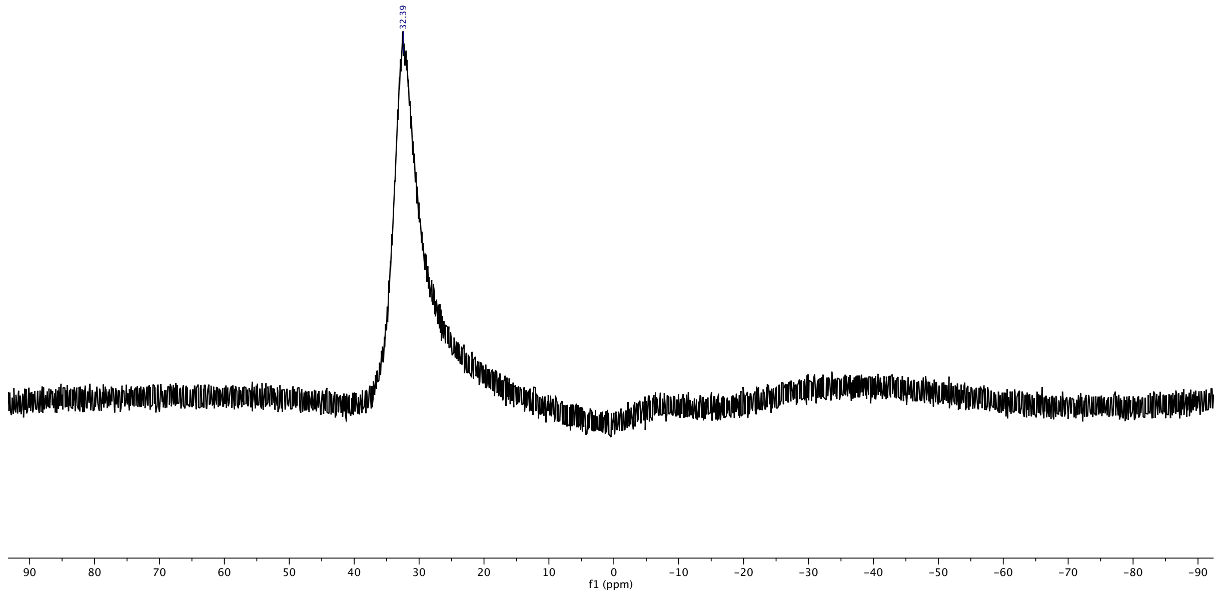

^{11}B NMR spectrum (128 MHz, $CDCl_3$)

2-((4*S,5*S**)-5-Butyl-8,8-dimethyl-4-vinylnon-1-en-6-yn-5-yl)-4,4,5,5-tetramethyl-1,3,2-dioxaborolane (6p)**

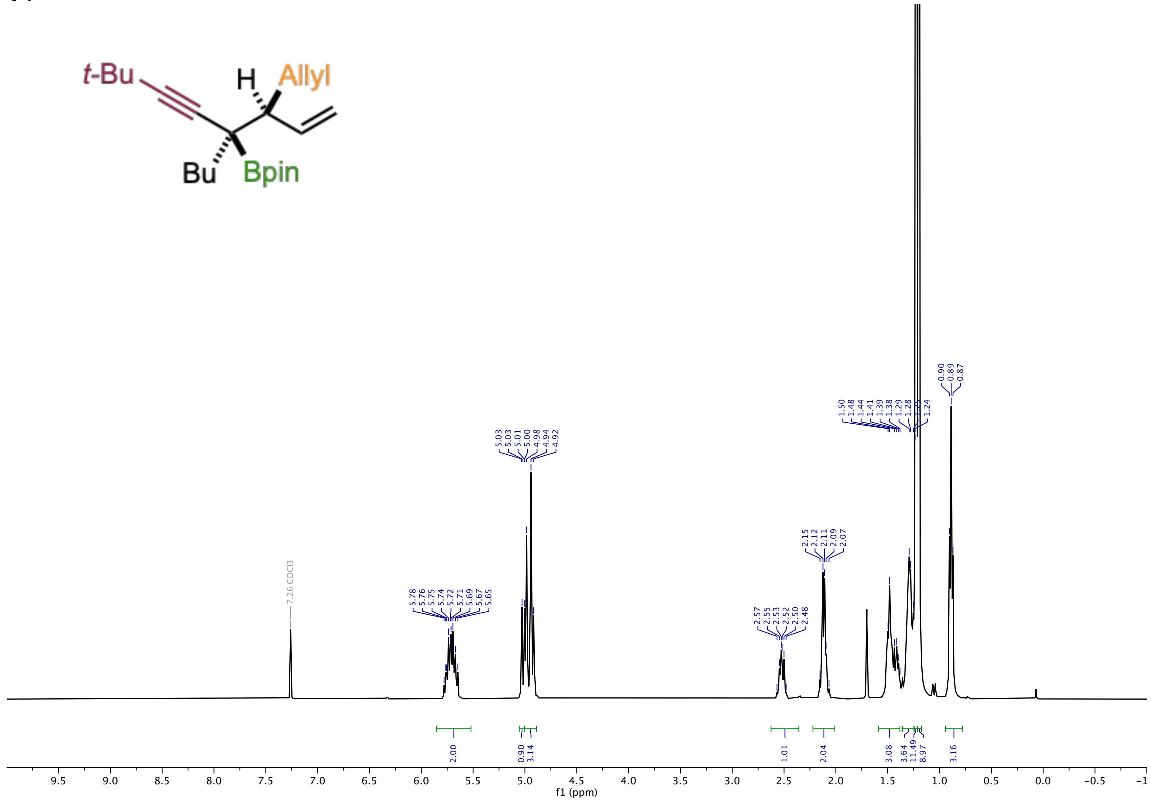

^{1}H NMR spectrum (400 MHz, $CDCl_3$)

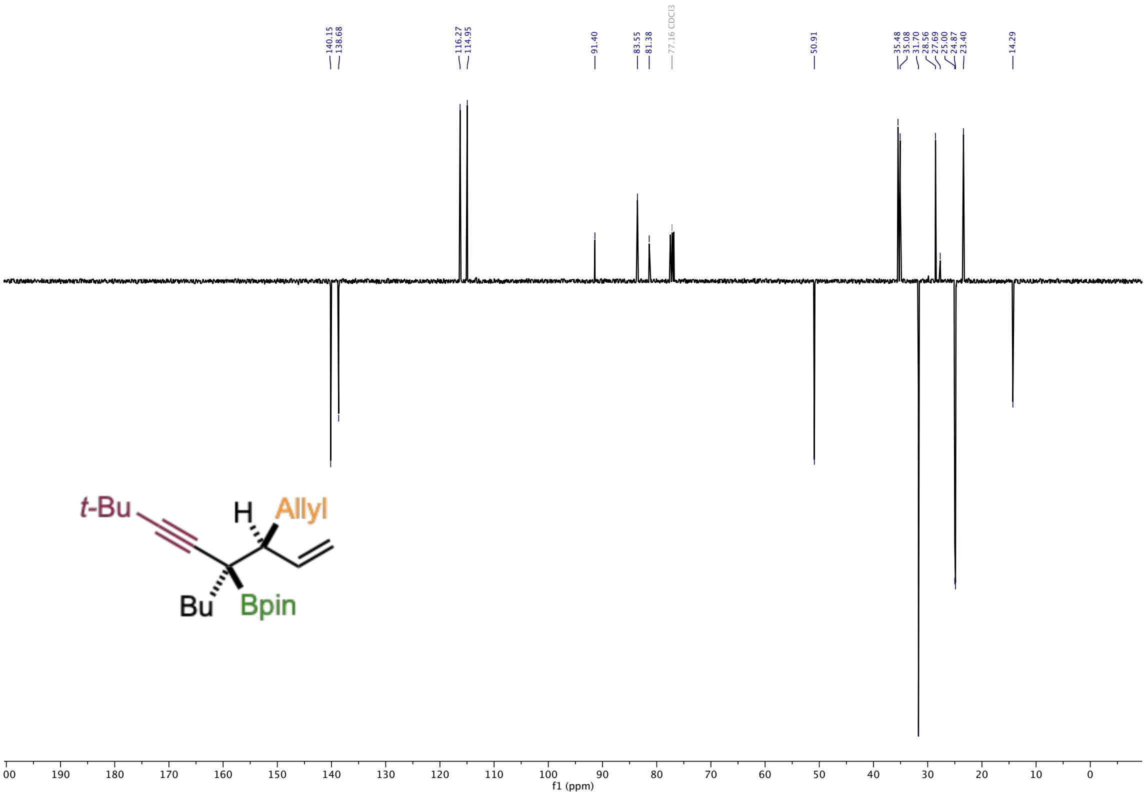

^{13}C NMR (APT) spectrum (101 MHz, $CDCl_3$)

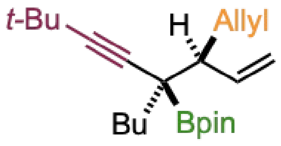

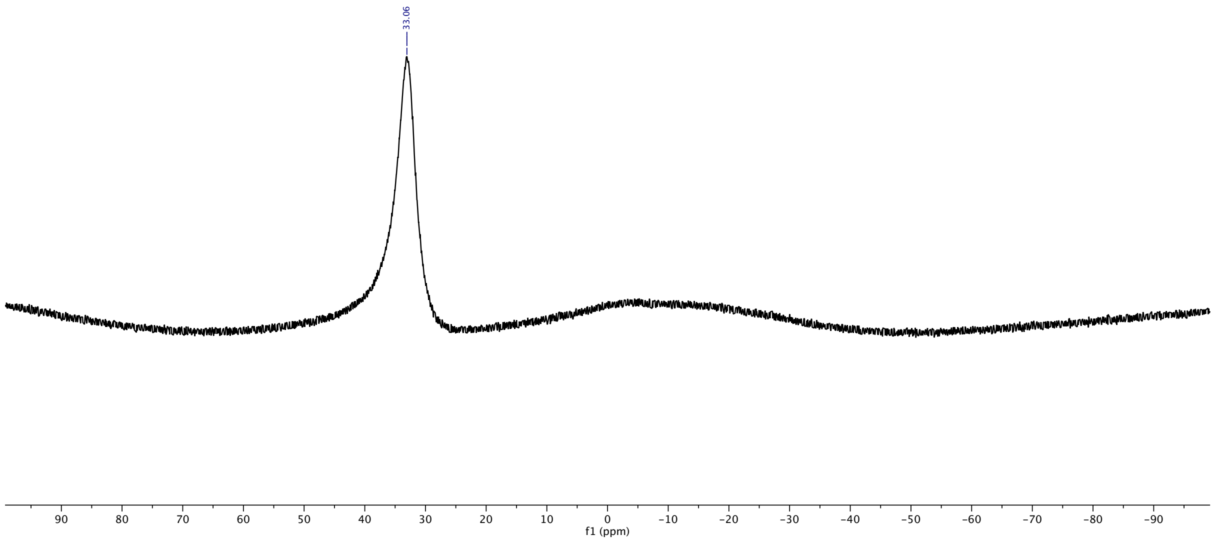

^{11}B NMR spectrum (128 MHz, $CDCl_3$)

4,4,5,5-Tetramethyl-2-((4*S,5*S**)-5-(prop-1-yn-1-yl)-4-vinylnon-1-en-5-yl)-1,3,2-dioxaborolane (6q)**

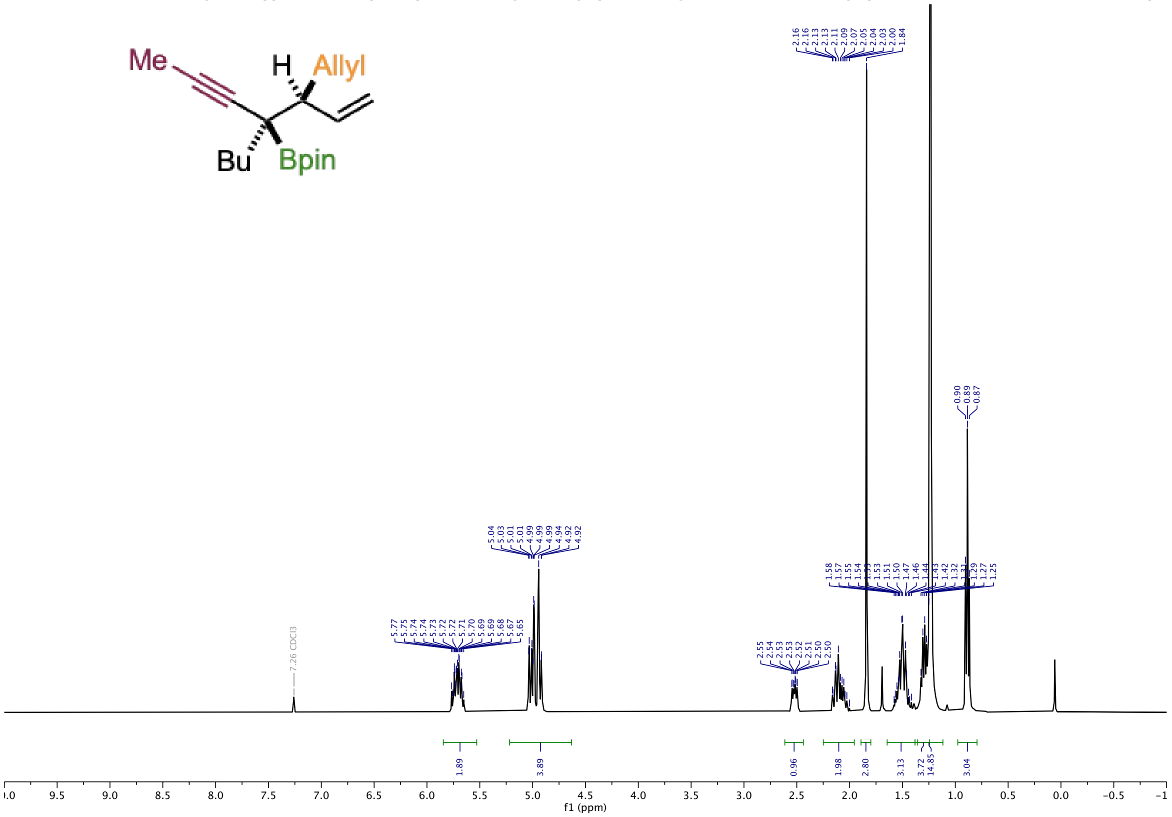

^{1}**H NMR** spectrum (400 MHz, CDCl$_3$)

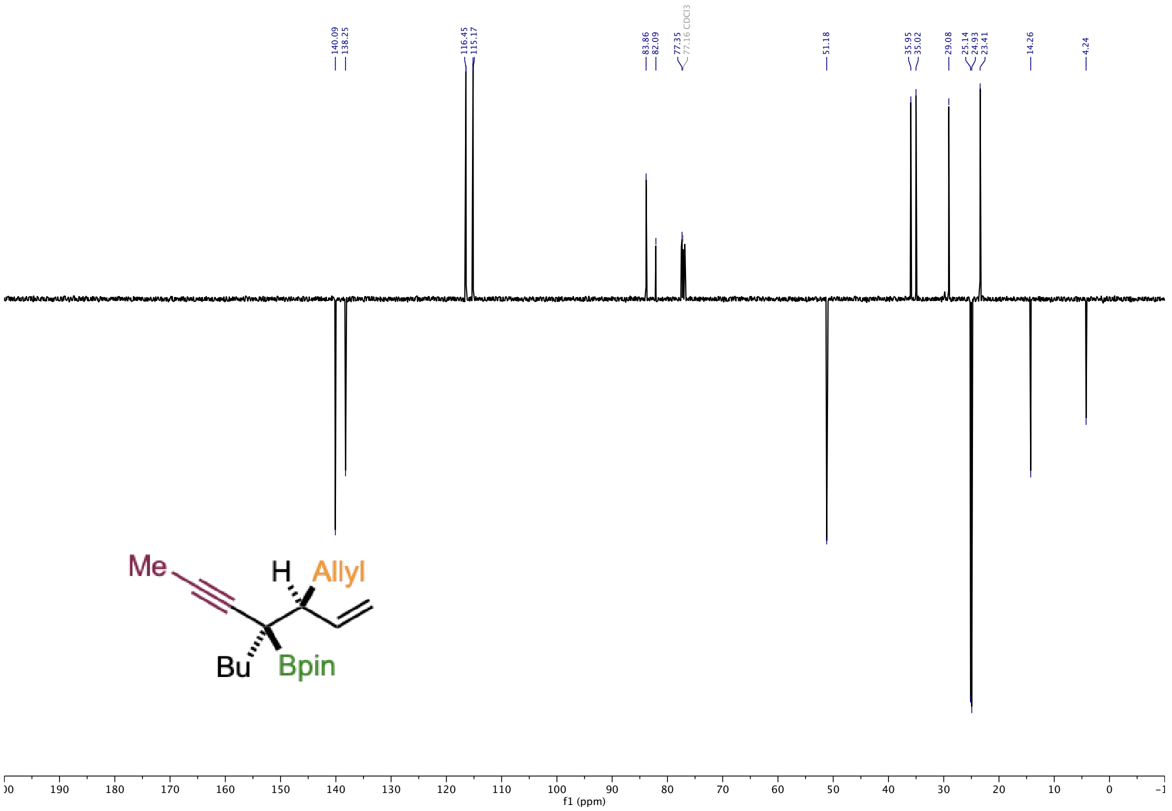

^{13}**C NMR** (APT) spectrum (101 MHz, CDCl$_3$)

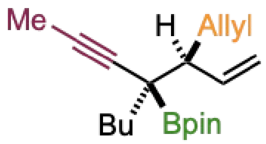

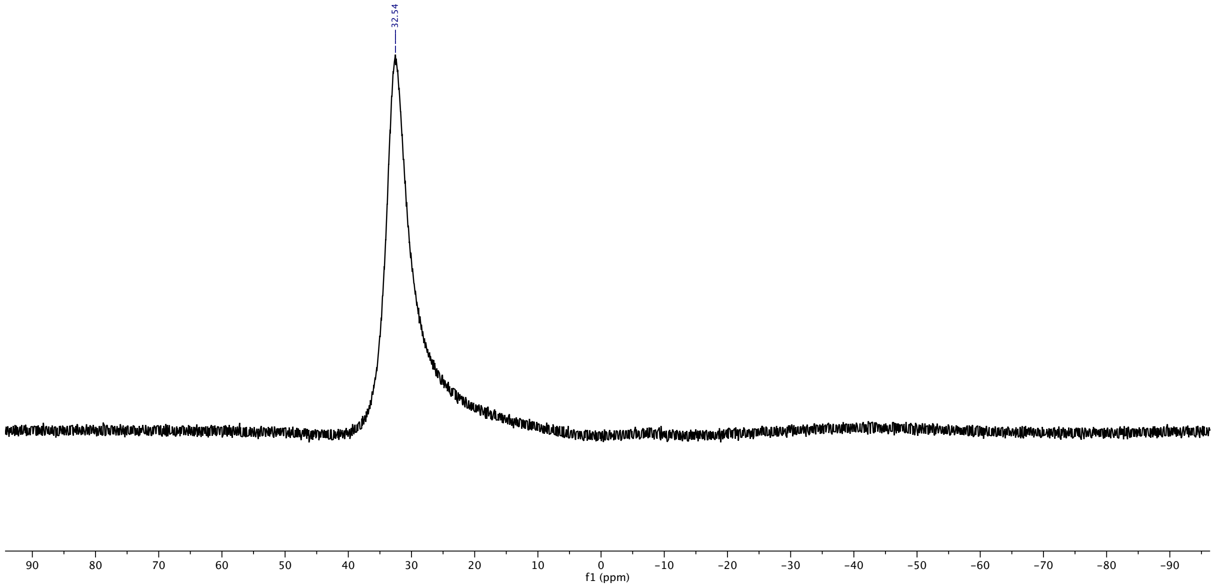

^{11}B NMR spectrum (128 MHz, $CDCl_3$)

4,4,5,5-Tetramethyl-2-((4*S,5*S**)-5-(phenylethynyl)-4-vinylnon-1-en-5-yl)-1,3,2-dioxaborolane (6r)**

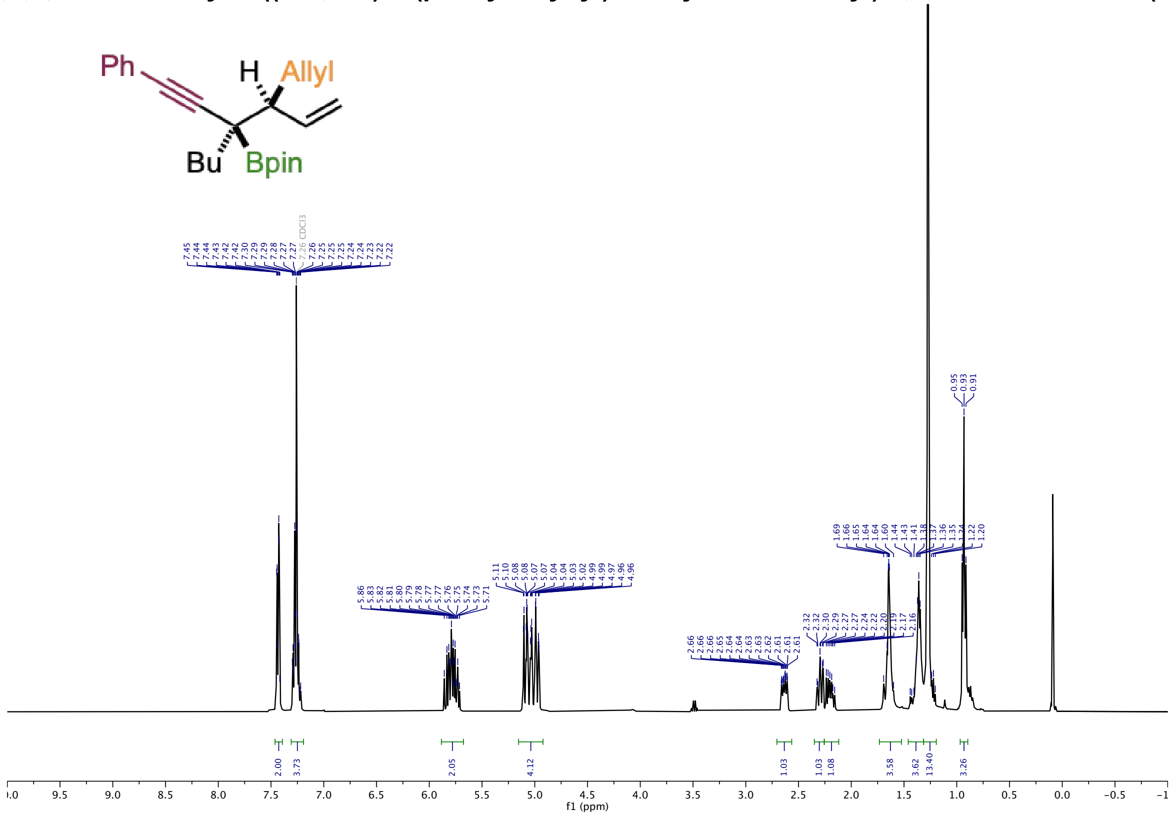

¹H NMR spectrum (400 MHz, CDCl$_3$)

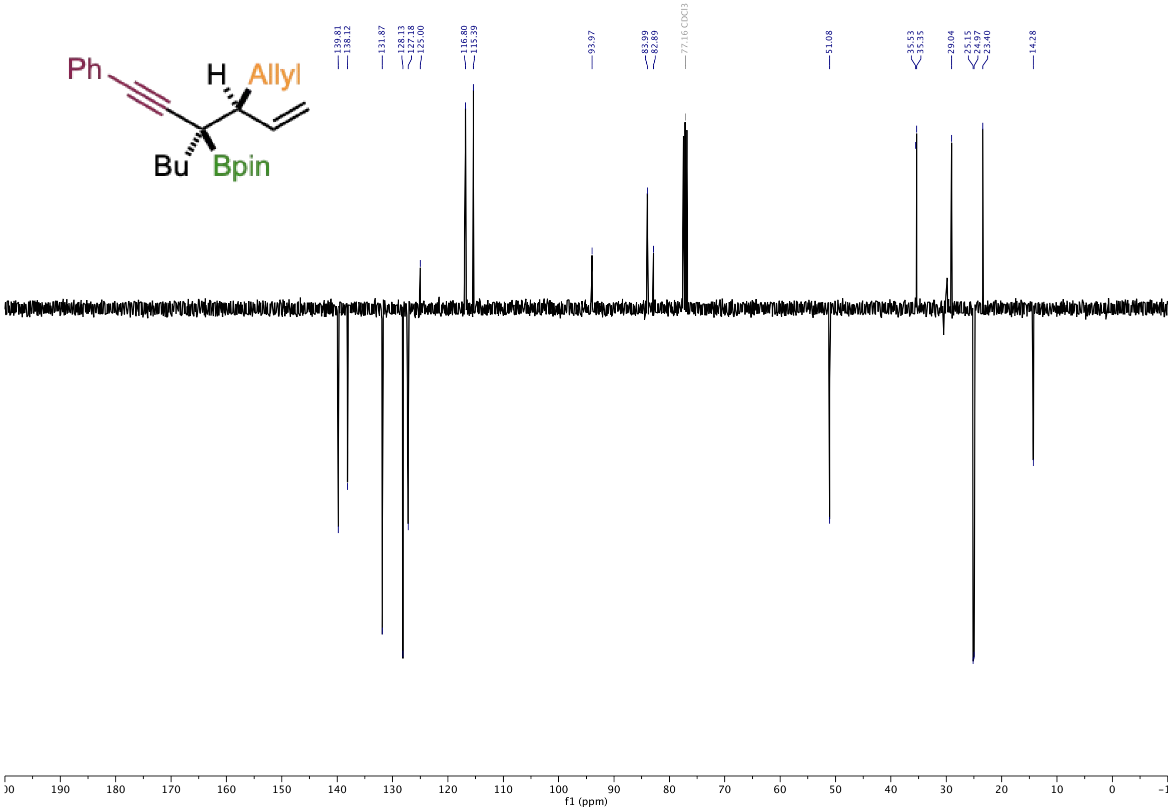

¹³C NMR (APT) spectrum (101 MHz, CDCl$_3$)

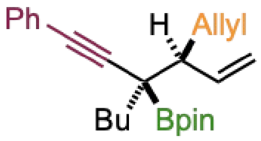

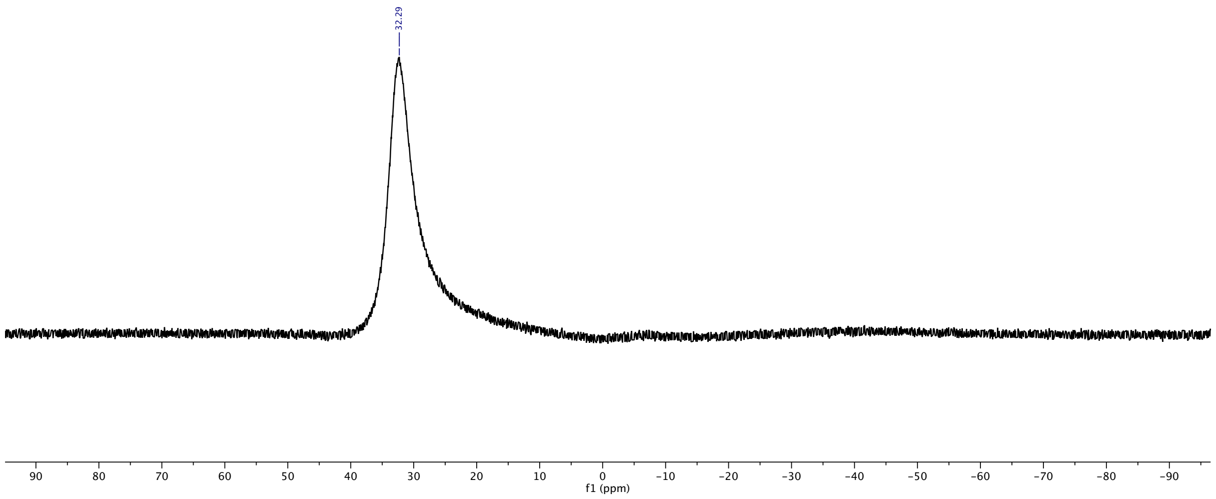

^{11}B NMR spectrum (128 MHz, $CDCl_3$)

4,4,5,5-Tetramethyl-2-((4*S,5*S**)-5-(triisopropylsilyl(ethynyl))-4-vinylnon-1-en-5-yl)-1,3,2-dioxaborolane (6s)**

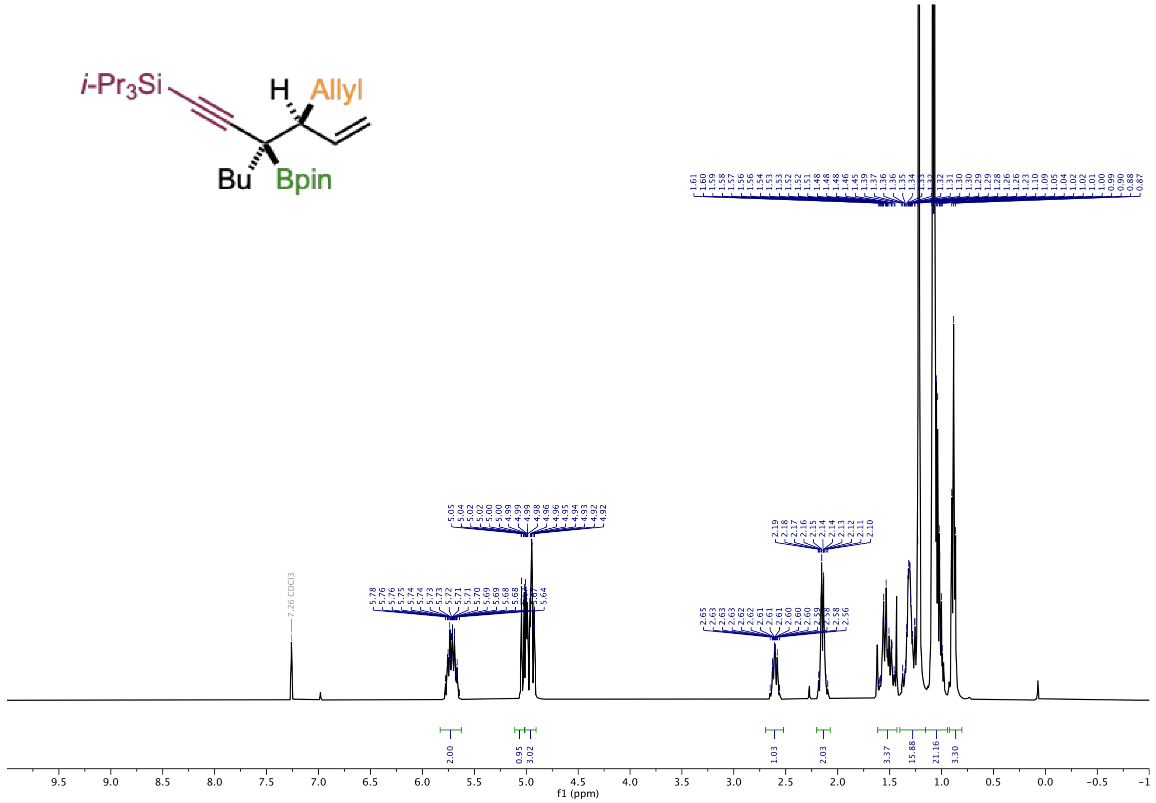

^{1}**H NMR** spectrum (400 MHz, $CDCl_3$)

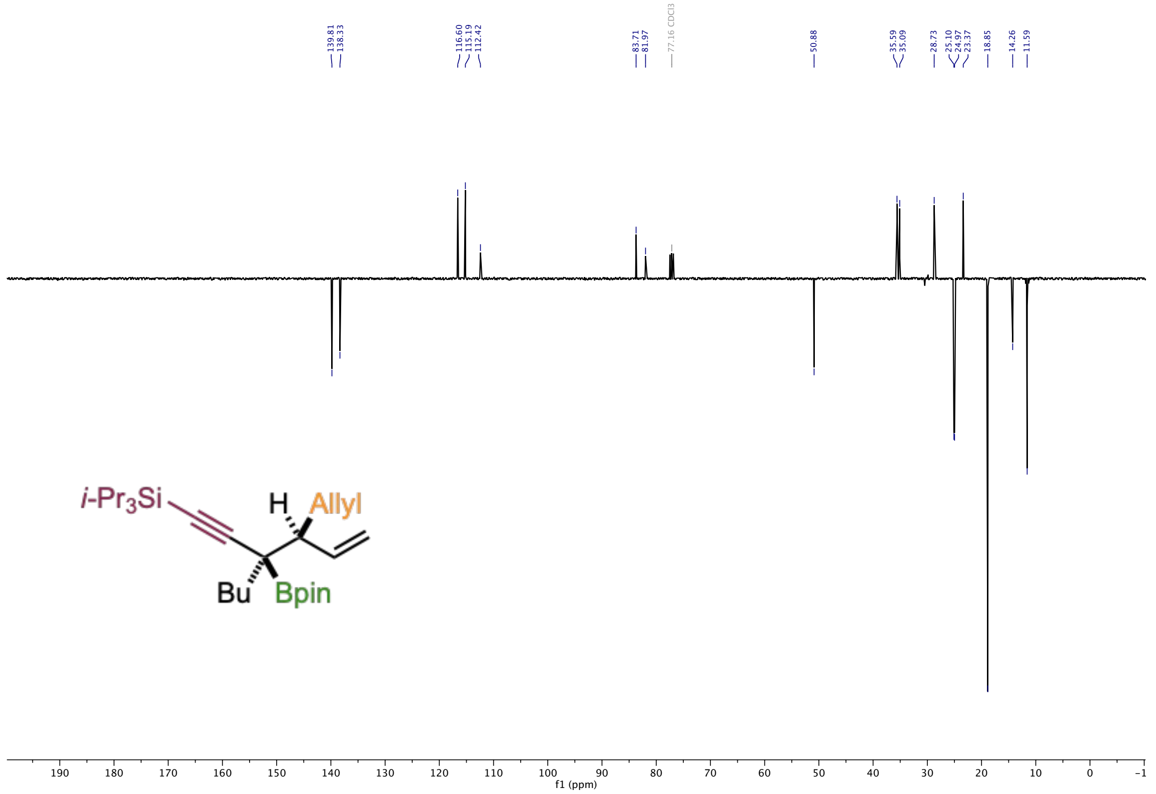

^{13}**C NMR** (APT) spectrum (101 MHz, $CDCl_3$)

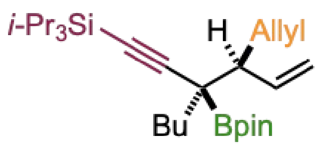

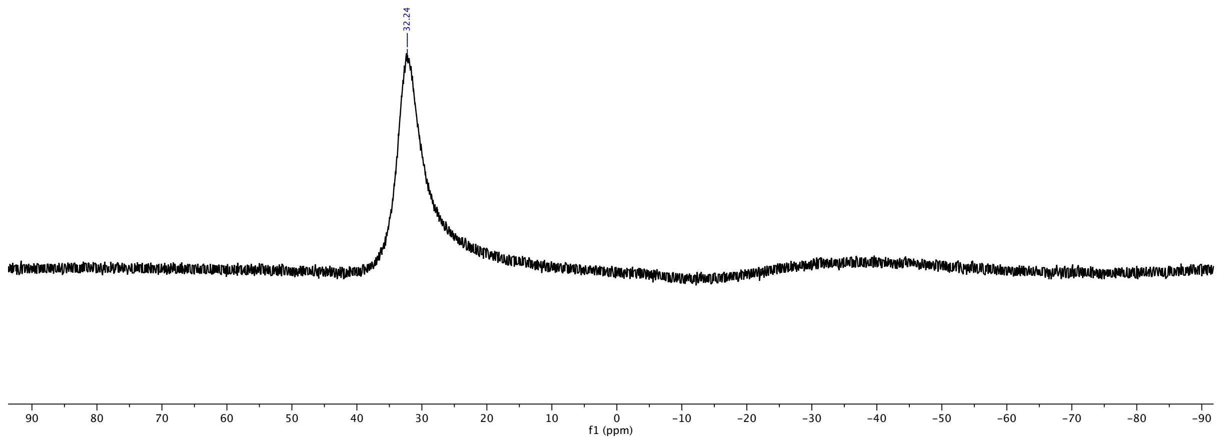

^{11}B NMR spectrum (128 MHz, $CDCl_3$)

2-(3,4-Dimethyldec-1-en-5-yn-4-yl)-4,4,5,5-tetramethyl-1,3,2-dioxaborolane (6t)

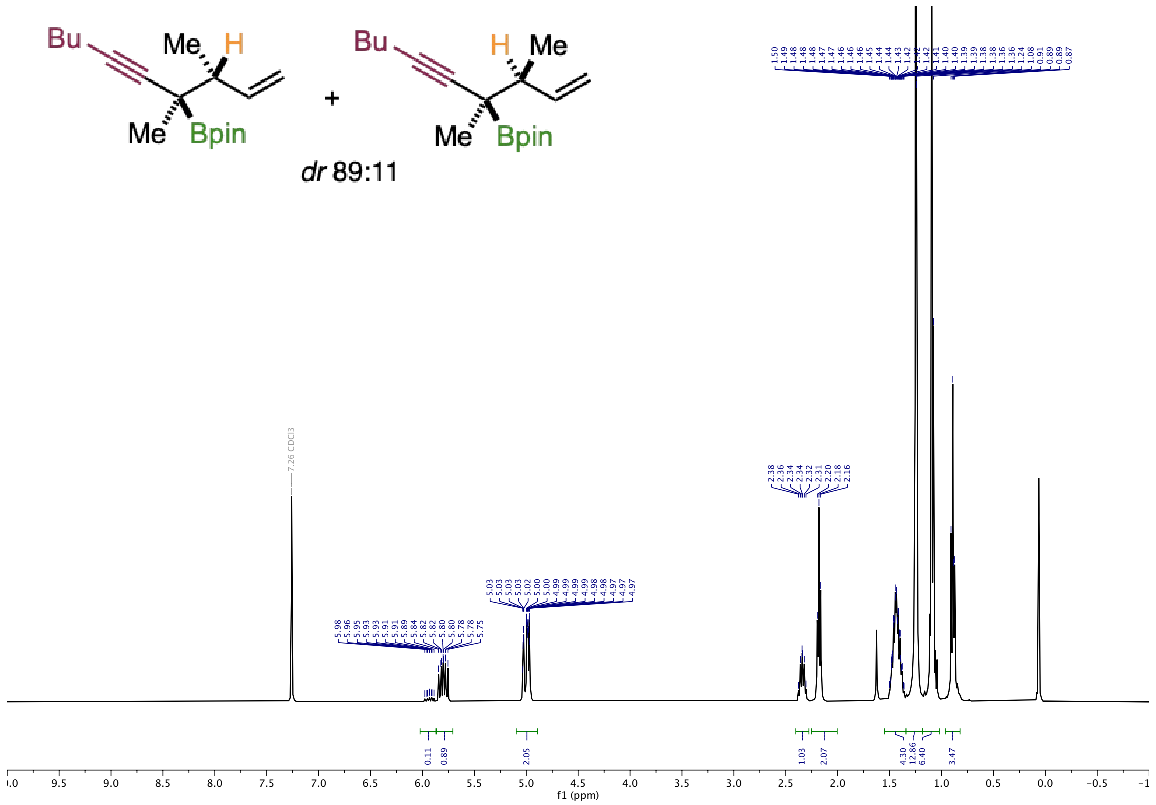

^{1}H NMR spectrum (400 MHz, $CDCl_3$)

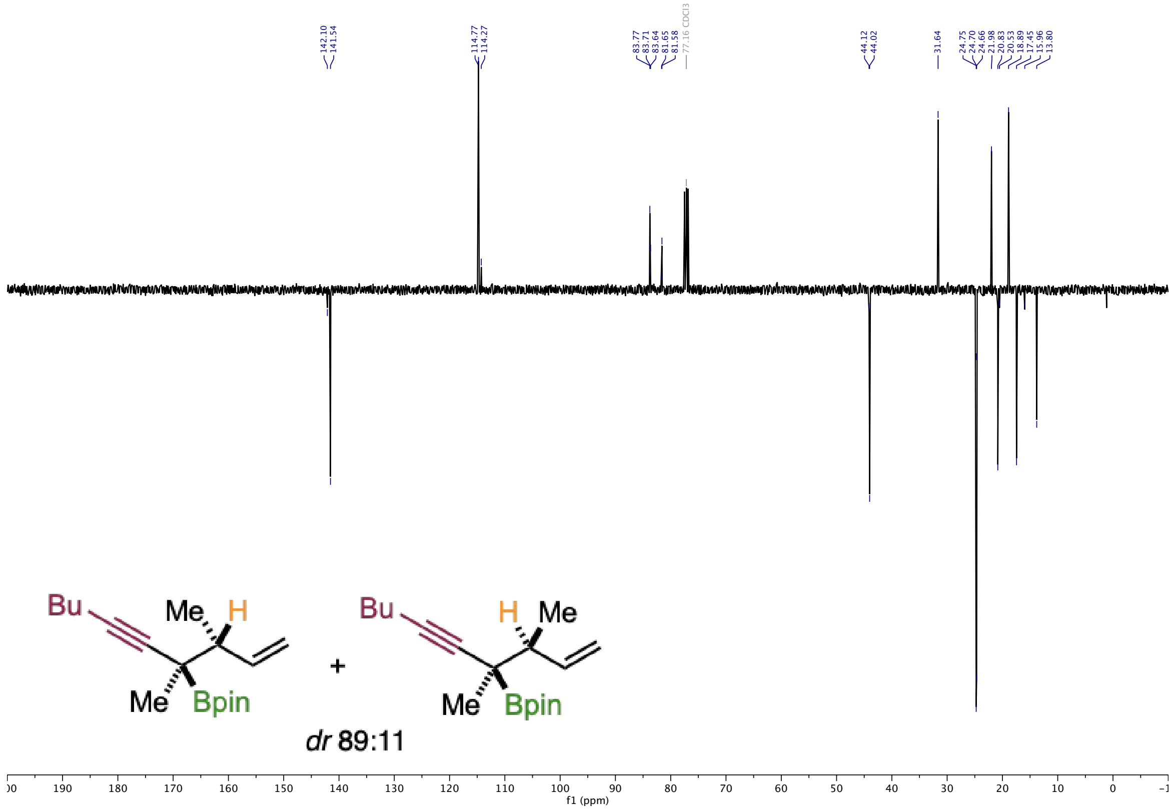

^{13}C NMR (APT) spectrum (101 MHz, $CDCl_3$)

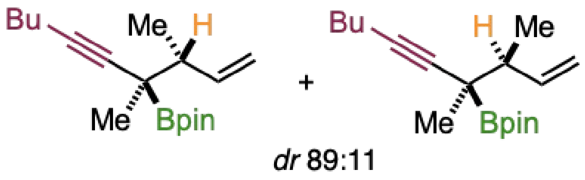

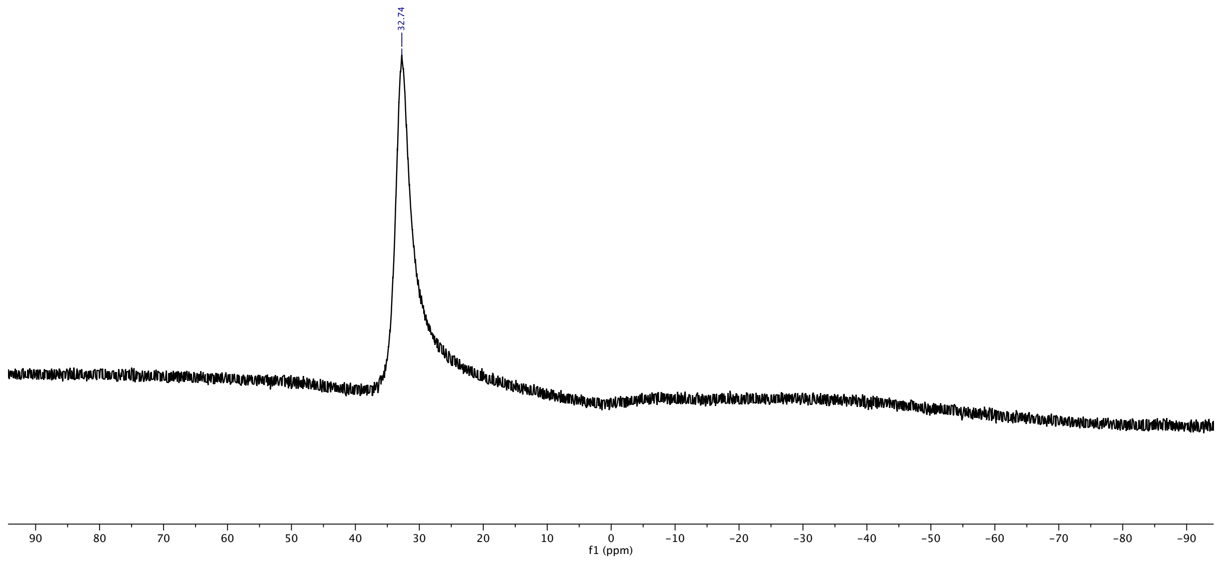

11B NMR spectrum (128 MHz, $CDCl_3$)

2-((3*R**,4*S**)-4-ethyl-3-phenyldec-1-en-5-yn-4-yl)-4,4,5,5-tetramethyl-1,3,2-dioxaborolane (6u)

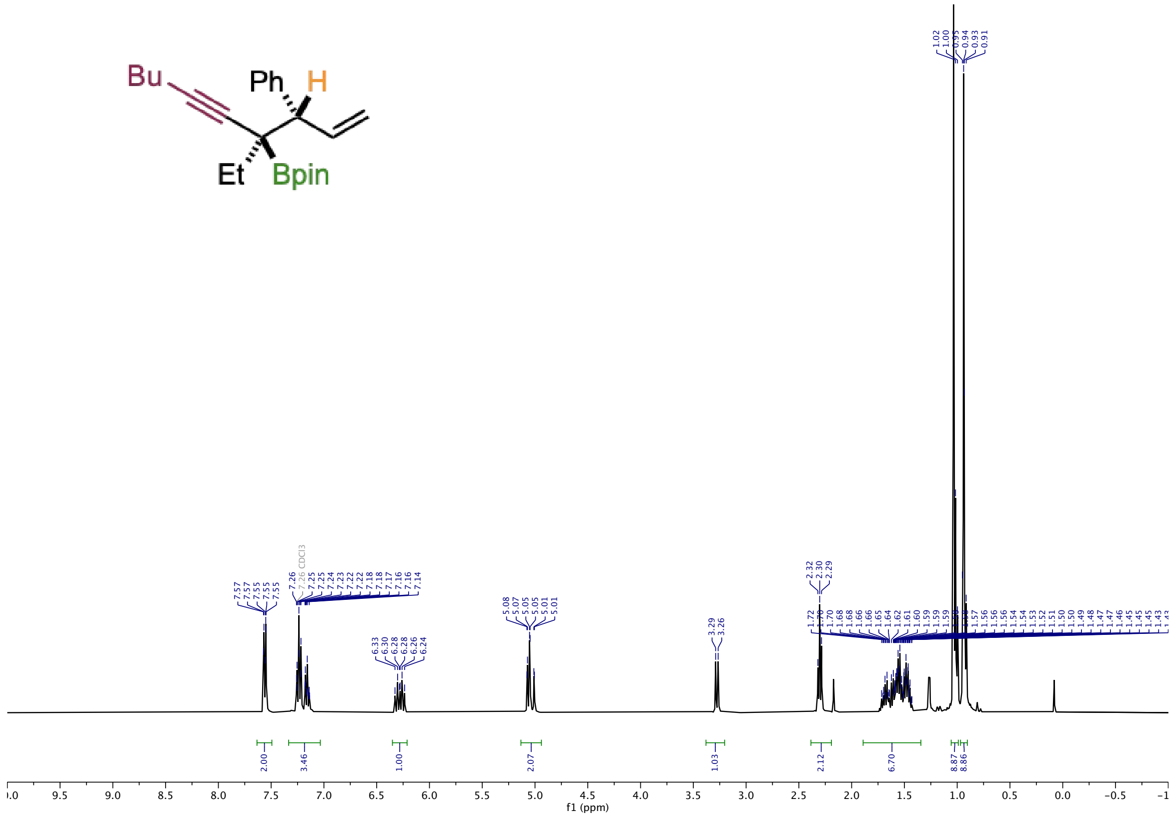

[1]**H NMR** spectrum (400 MHz, $CDCl_3$)

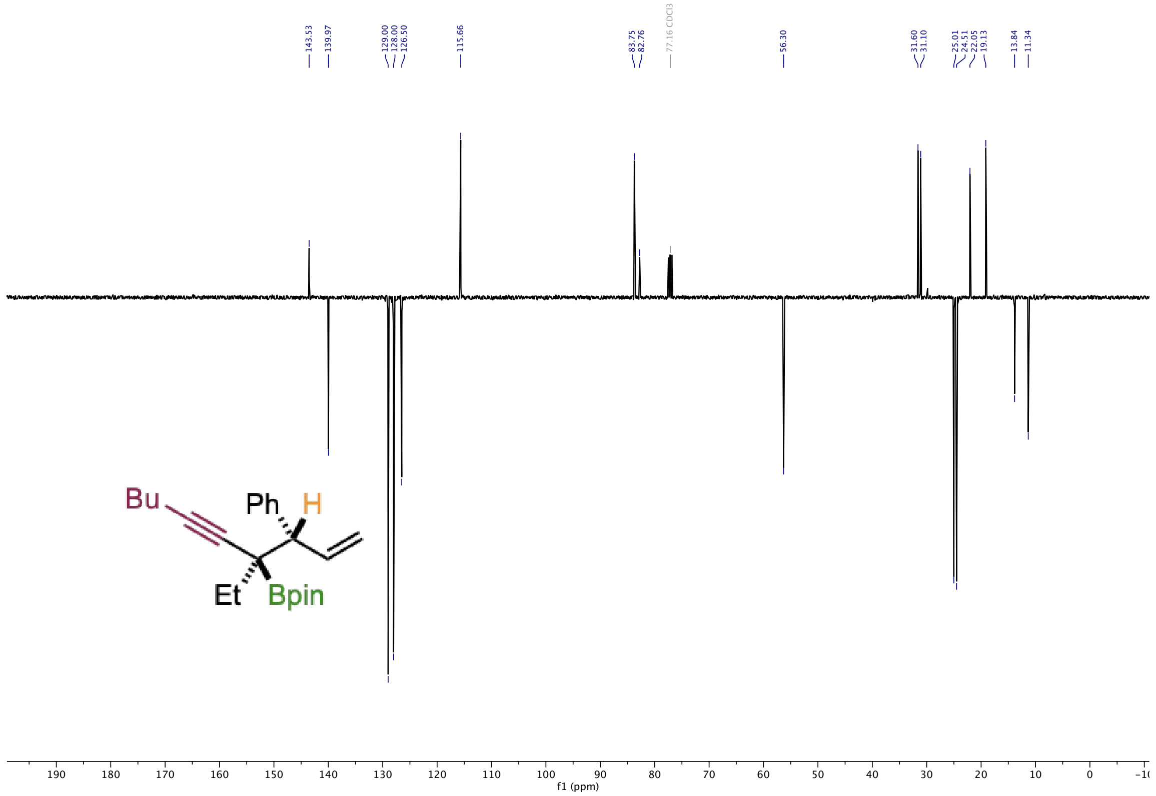

[13]**C NMR** (APT) spectrum (101 MHz, $CDCl_3$)

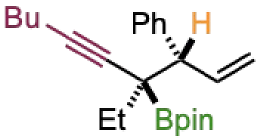

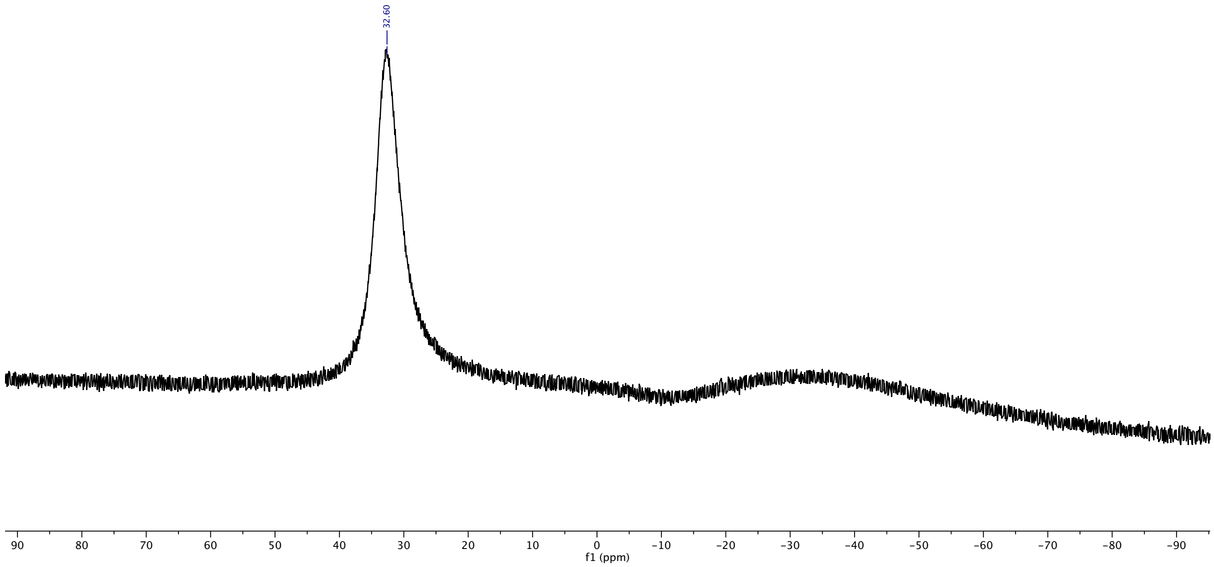

^{11}B NMR spectrum (128 MHz, $CDCl_3$)

2-((3*R*,*4*S)-3,4-Diethyldec-1-en-5-yn-4-yl)-4,4,5,5-tetramethyl-1,3,2-dioxaborolane (6v)**

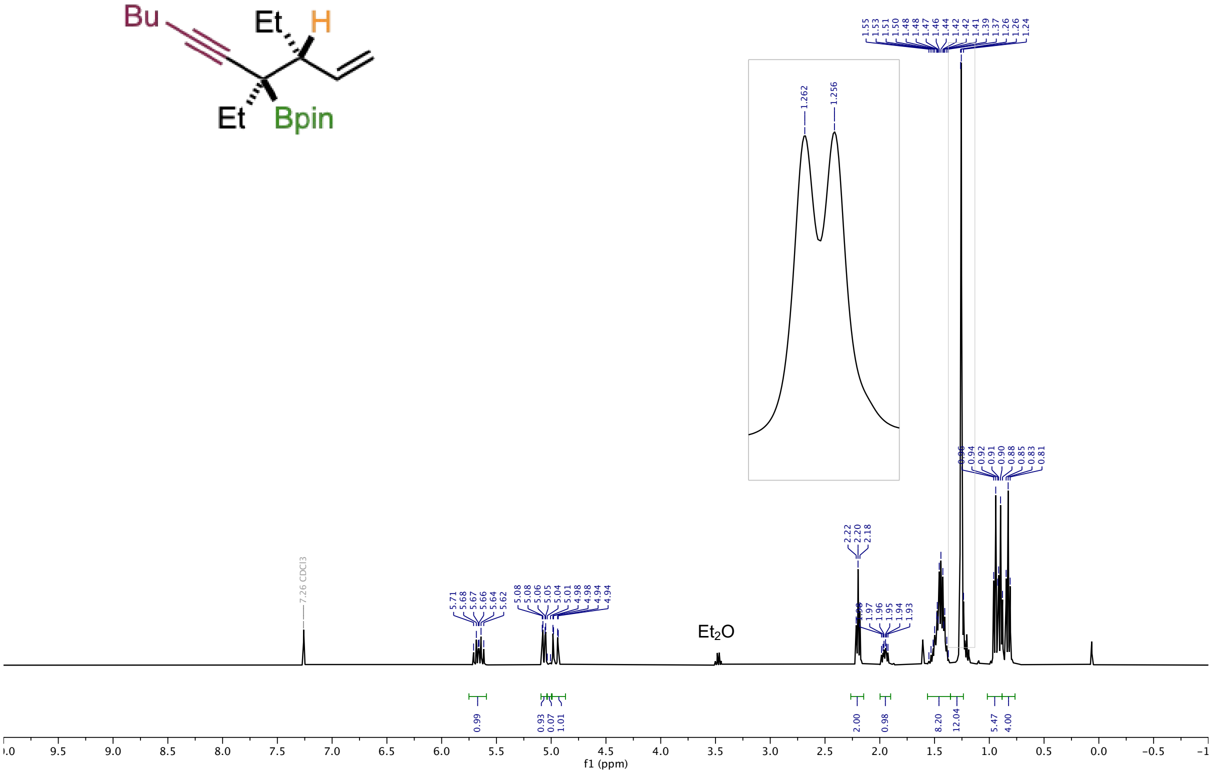

^{1}H NMR spectrum (400 MHz, CDCl$_3$)

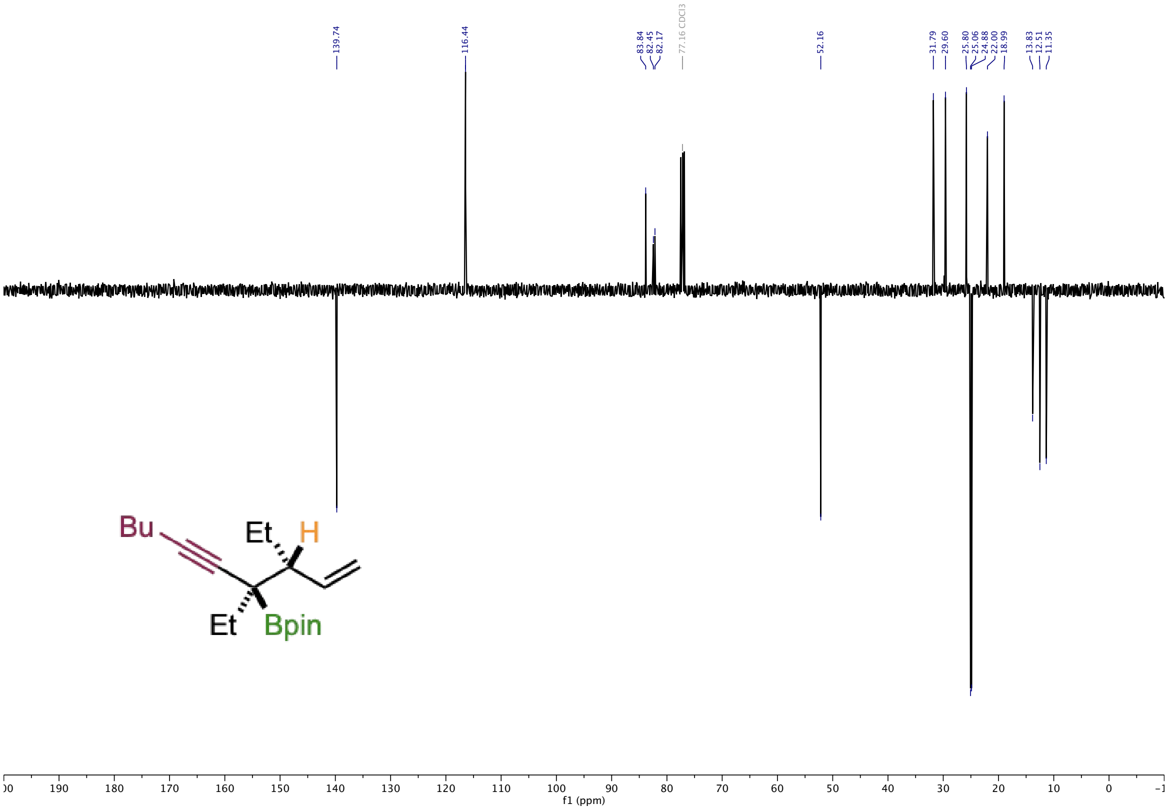

^{13}C NMR (APT) spectrum (101 MHz, CDCl$_3$)

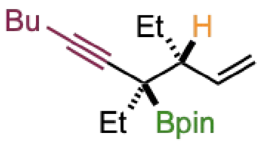

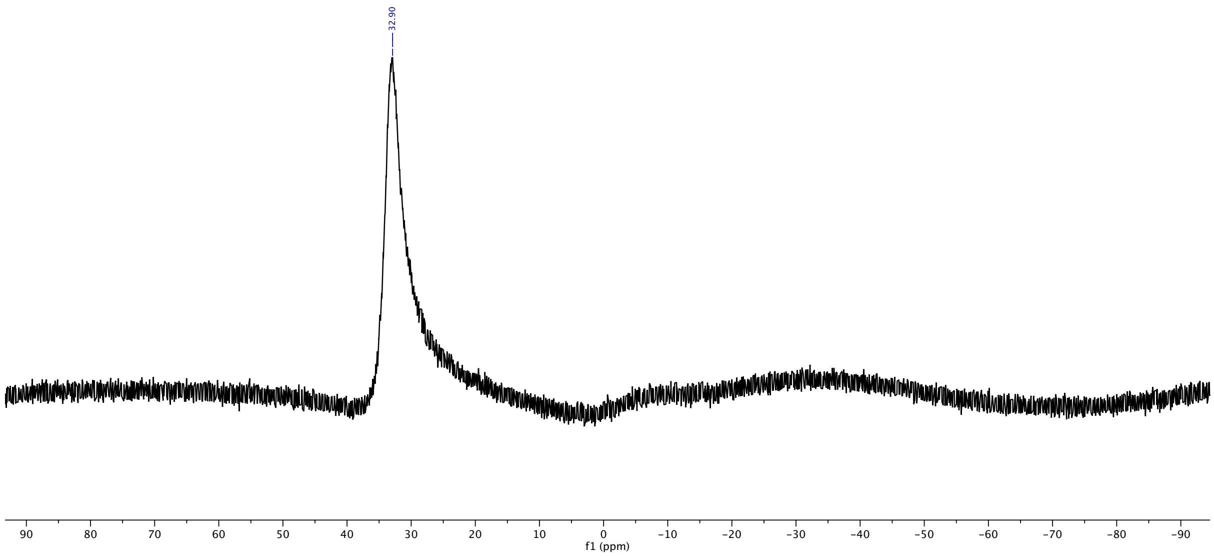

^{11}B NMR spectrum (128 MHz, $CDCl_3$)

2-((3*R,4*S**)-3,4-Diethyl-7,7-dimethyloct-1-en-5-yn-4-yl)-4,4,5,5-tetramethyl-1,3,2-dioxaborolane (6w)**

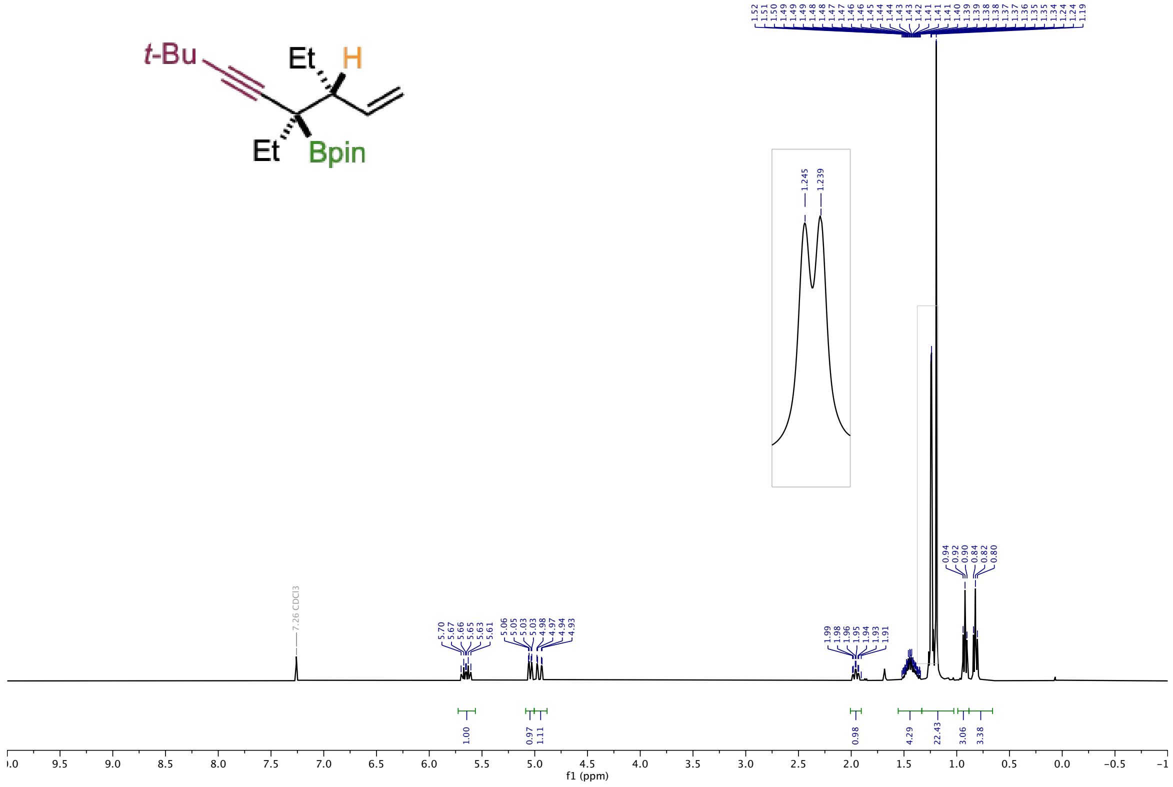

^{1}H NMR spectrum (400 MHz, $CDCl_3$)

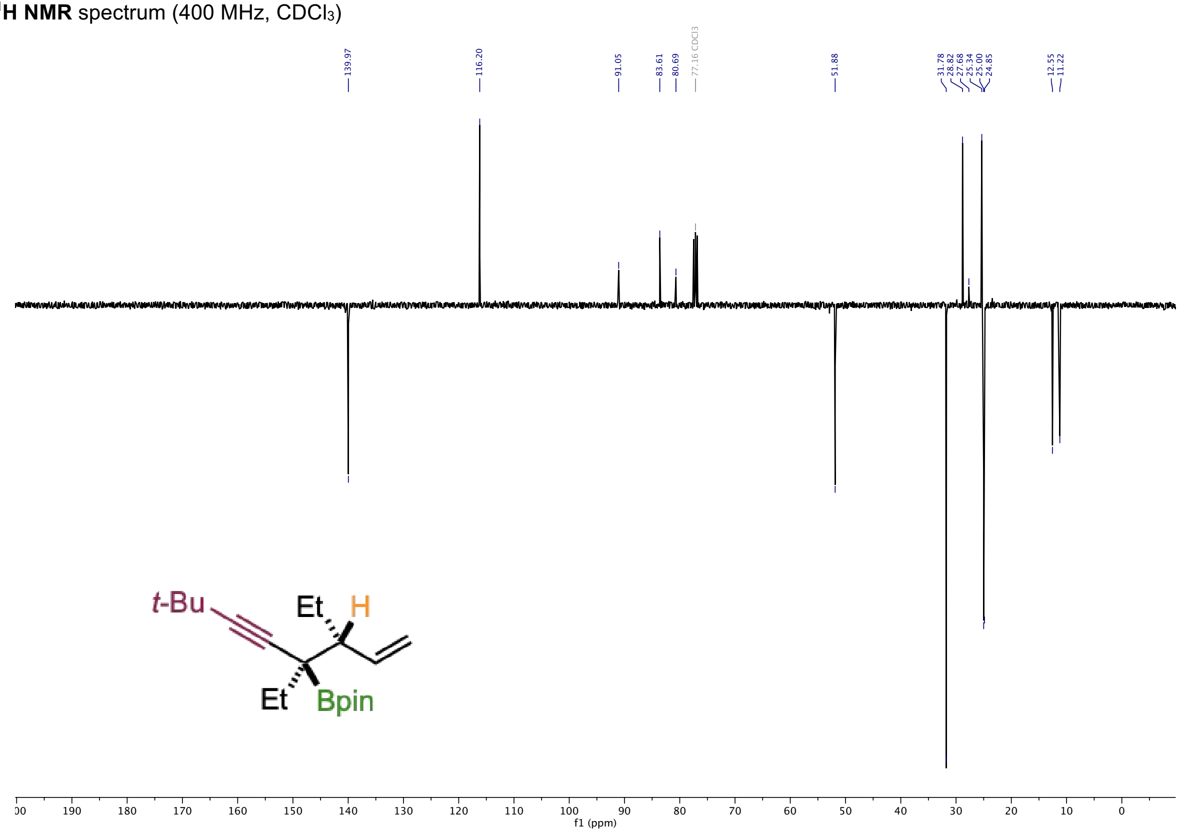

^{13}C NMR (APT) spectrum (101 MHz, $CDCl_3$)

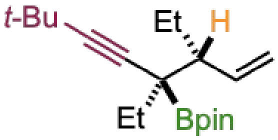

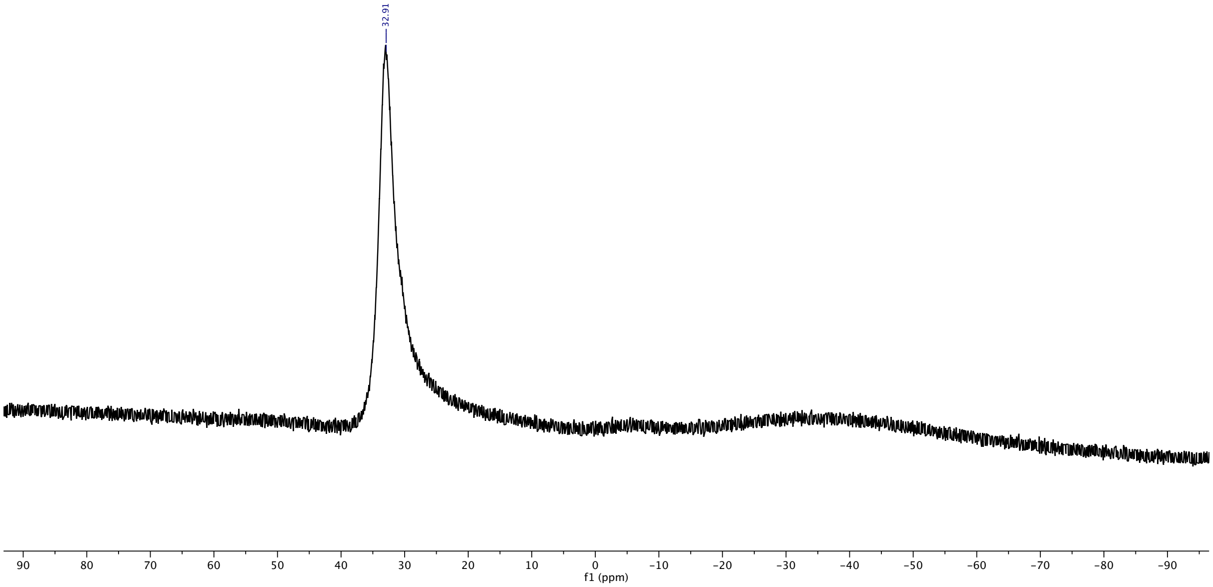

^{11}B NMR spectrum (128 MHz, $CDCl_3$)

2-(3,4-Diethyl-1-phenylhex-5-en-1-yn-3-yl)-4,4,5,5-tetramethyl-1,3,2-dioxaborolane (6x)

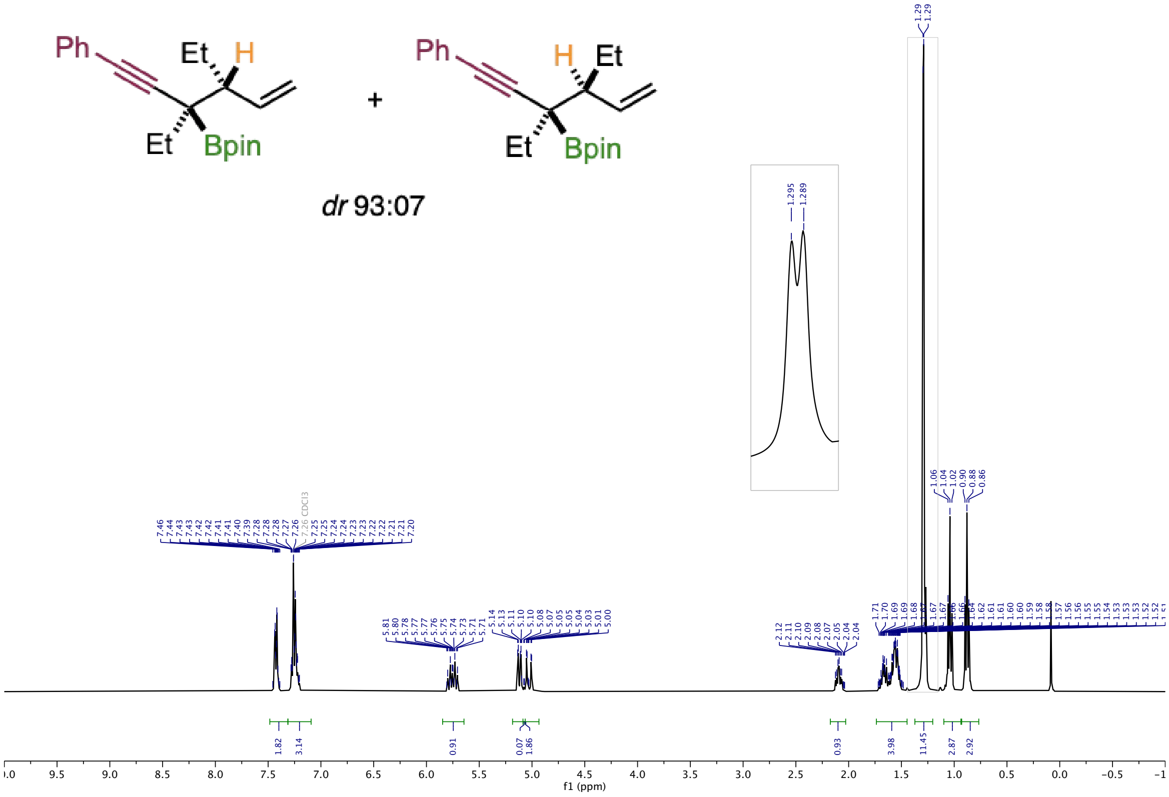

^{1}H NMR spectrum (400 MHz, $CDCl_3$)

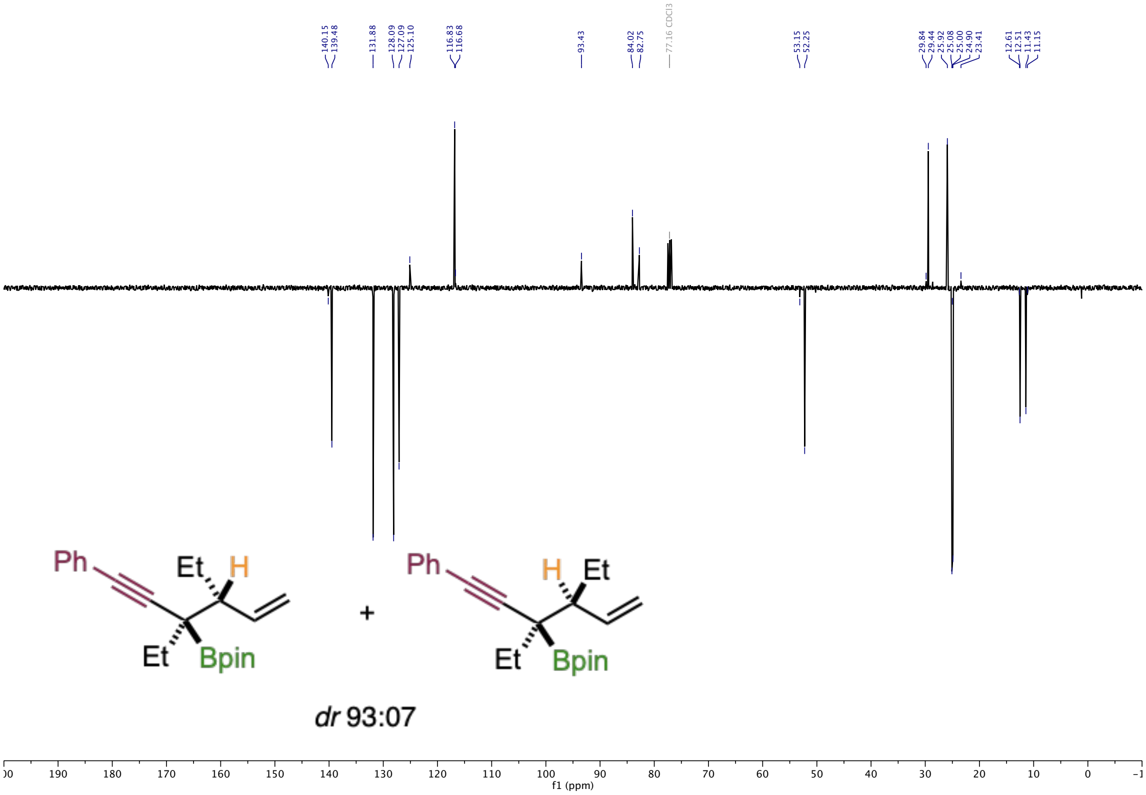

^{13}C NMR (APT) spectrum (101 MHz, $CDCl_3$)

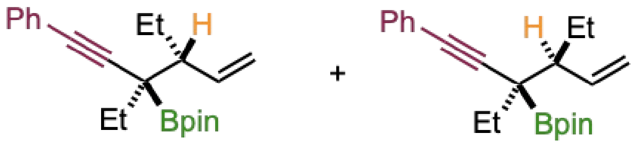

dr 93:07

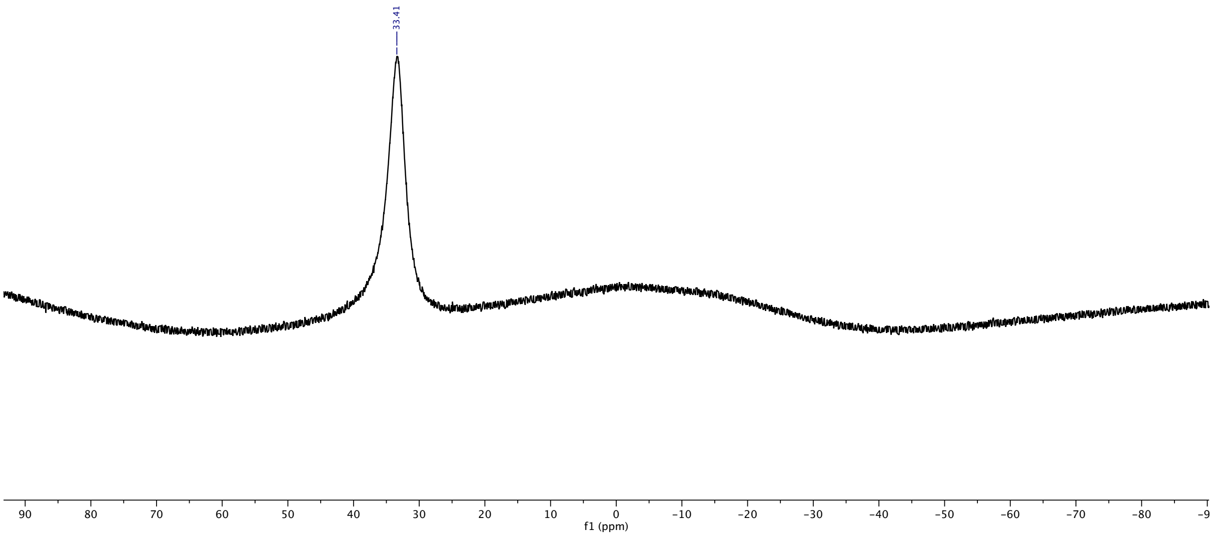

^{11}B NMR spectrum (128 MHz, $CDCl_3$)

2-(3,4-Diethyl-1-(triisopropylsilyl-5-en-1-yn-3-yl)-4,4,5,5-tetramethyl-1,3,2-dioxaborolane (6y)

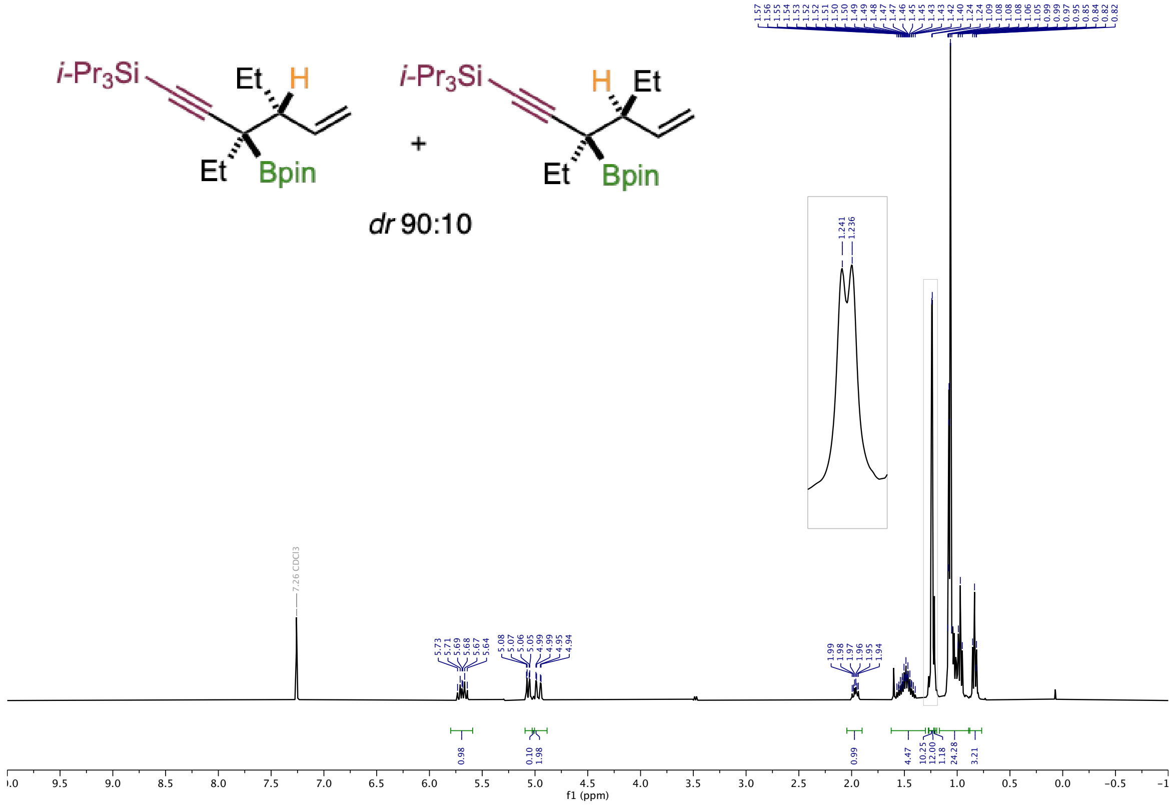

^{1}H NMR spectrum (400 MHz, $CDCl_3$)

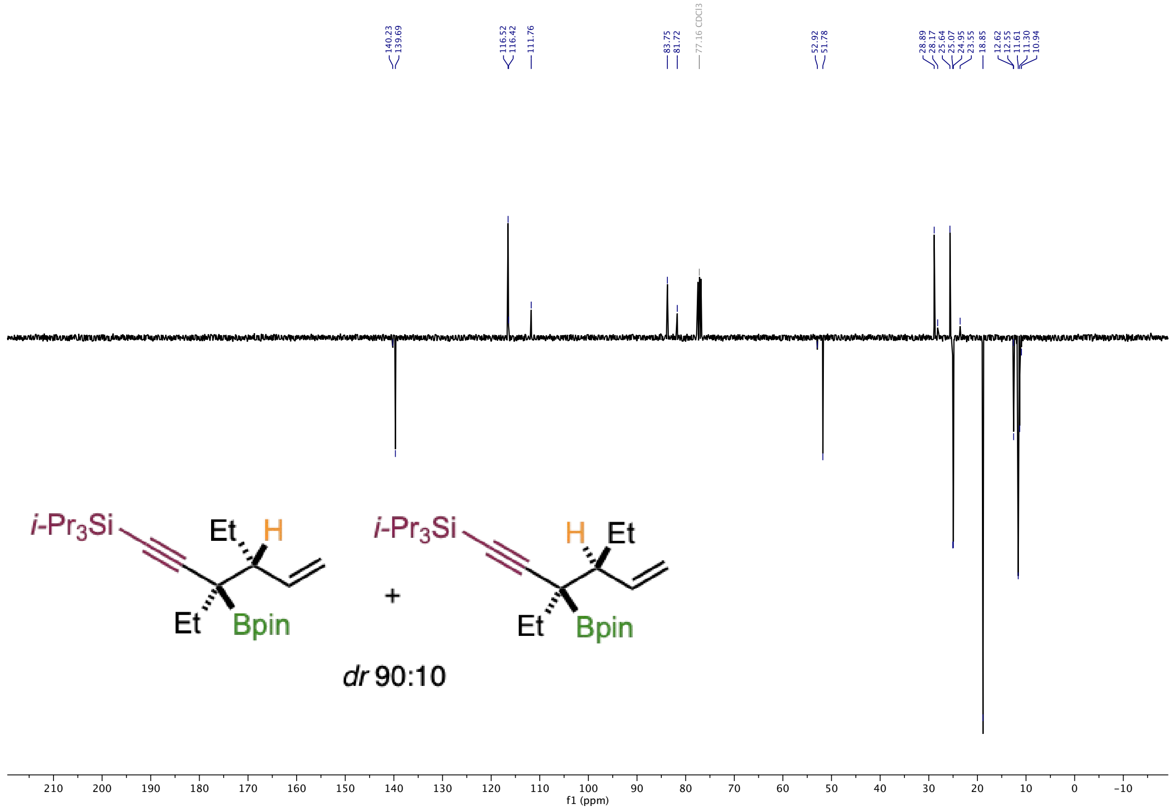

^{13}C NMR (APT) spectrum (101 MHz, $CDCl_3$)

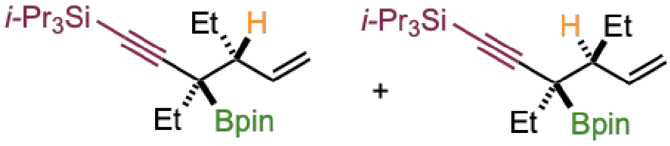

dr 90:10

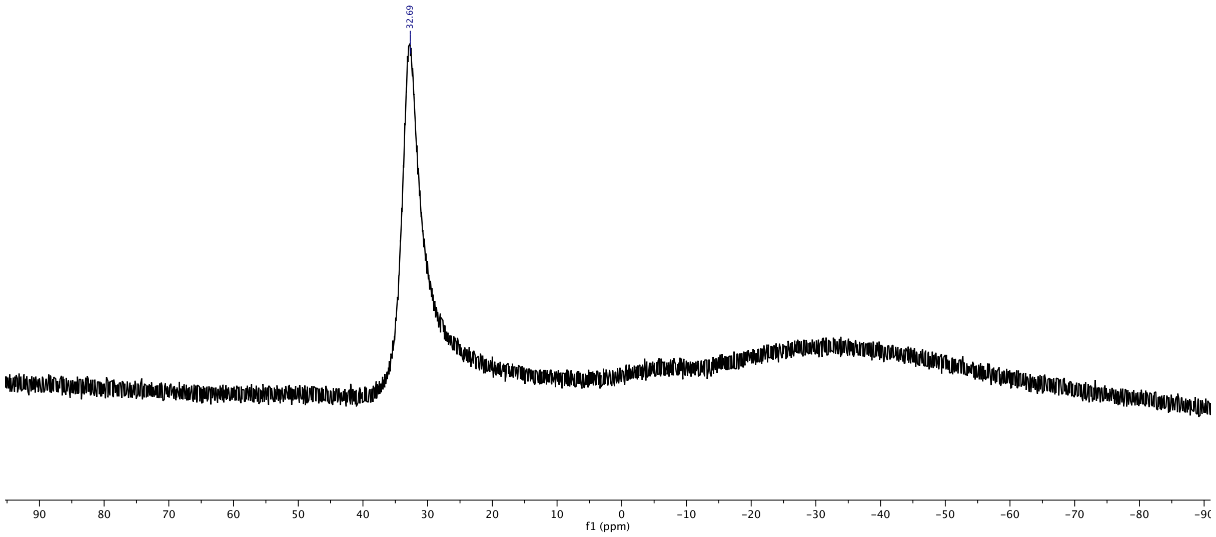

^{11}B NMR spectrum (128 MHz, $CDCl_3$)

2-(1-Cyclohexyl-3,4-diethylhex-5-en-1-yn-3-yl)-4,4,5,5-tetramethyl-1,3,2-dioxaborolane (6z)

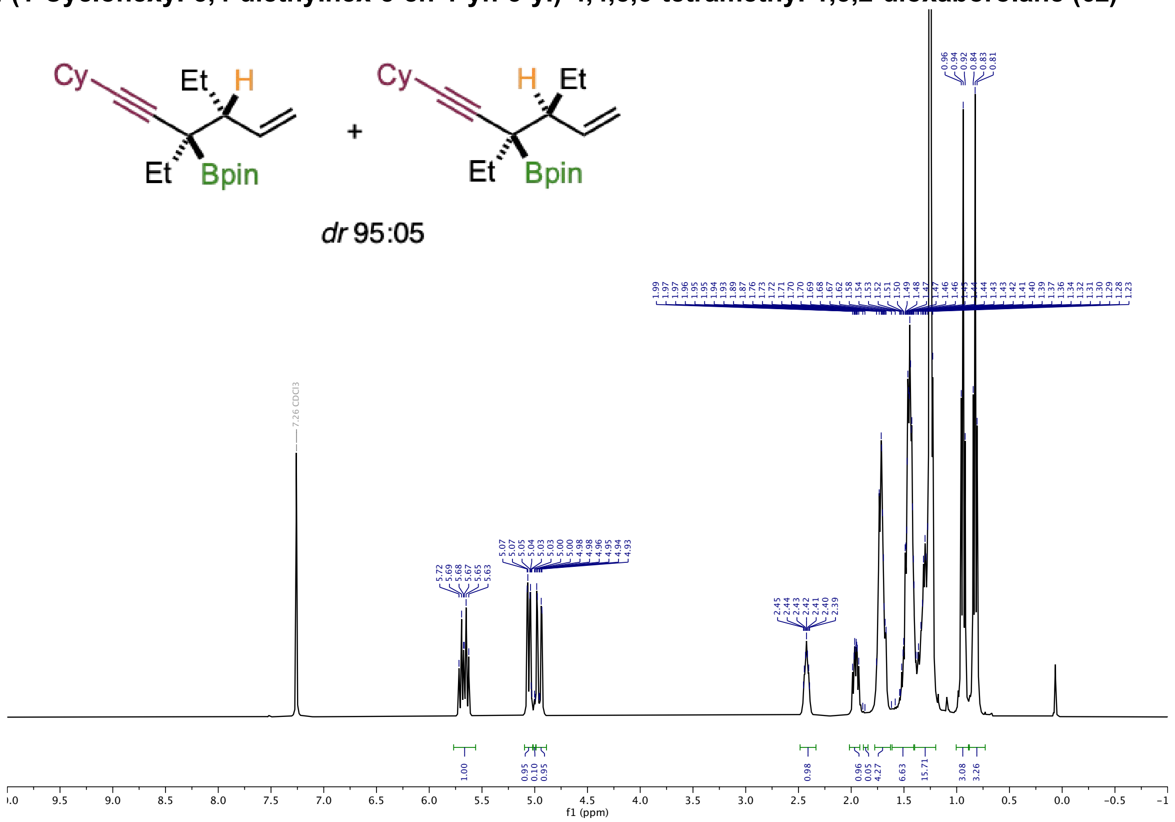

^{1}H NMR spectrum (400 MHz, $CDCl_3$)

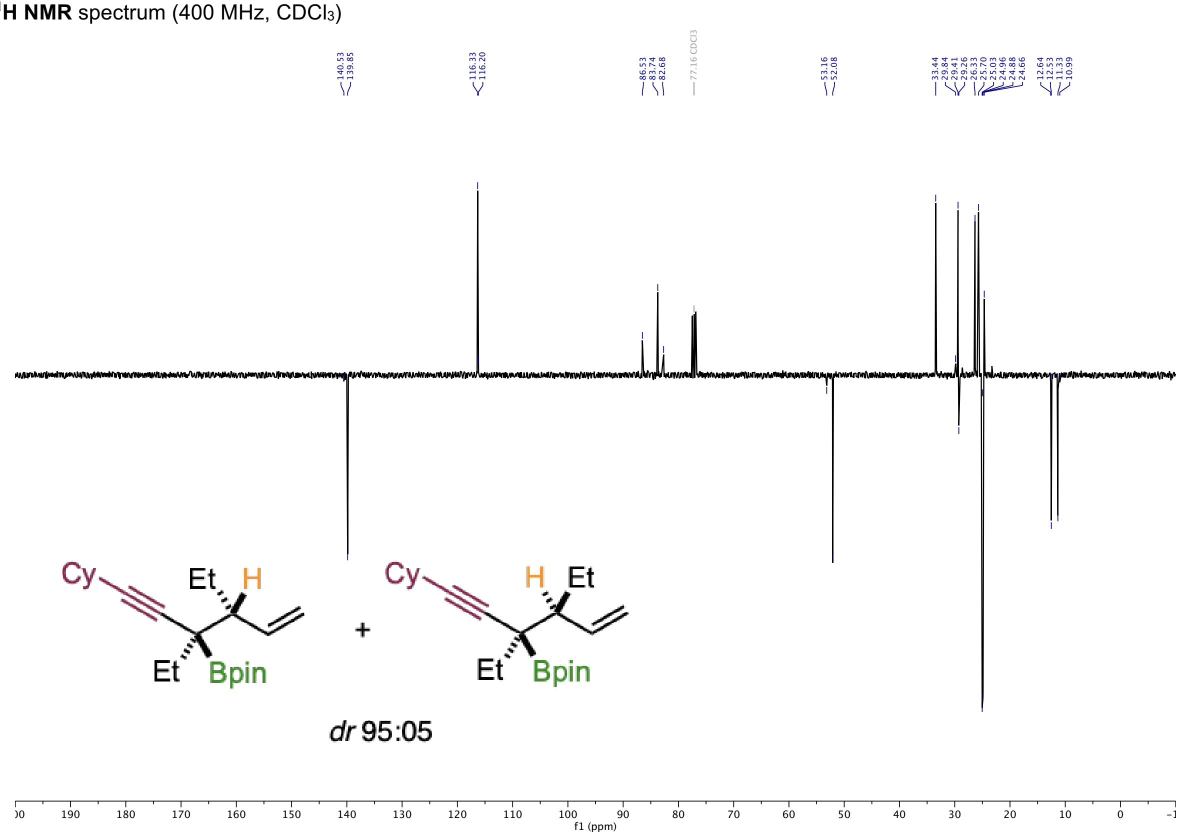

^{13}C NMR (APT) spectrum (101 MHz, $CDCl_3$)

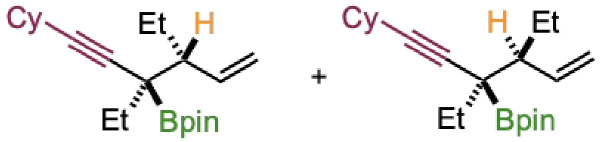

dr 95:05

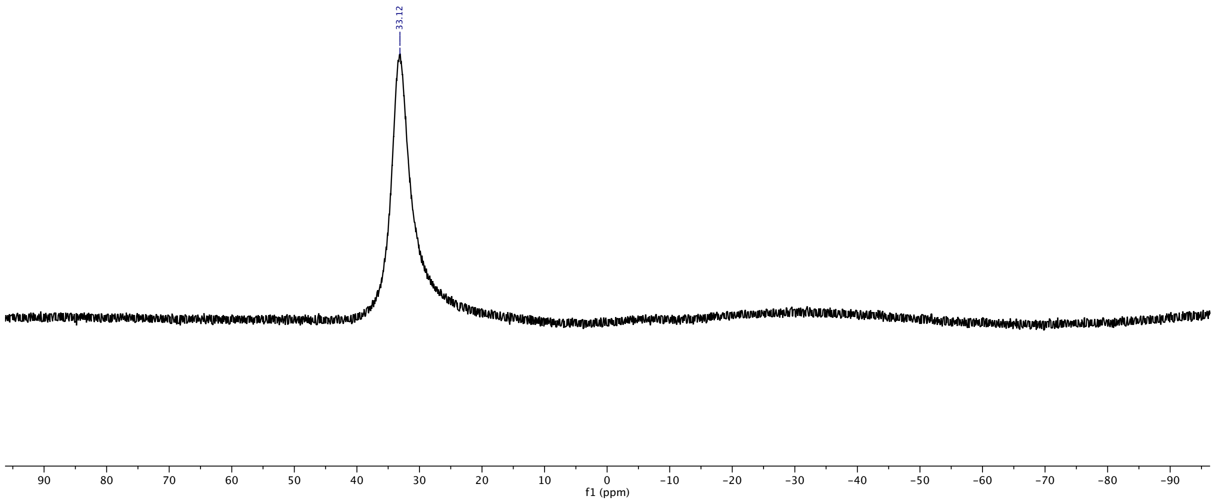

^{11}B NMR spectrum (128 MHz, $CDCl_3$)

2-((4*S**,5*S**)-4,5-Dimethyl-4-vinylundec-1-en-6-yn-5-yl)-4,4,5,5-tetramethyl-1,3,2-dioxaborolane (6aa)

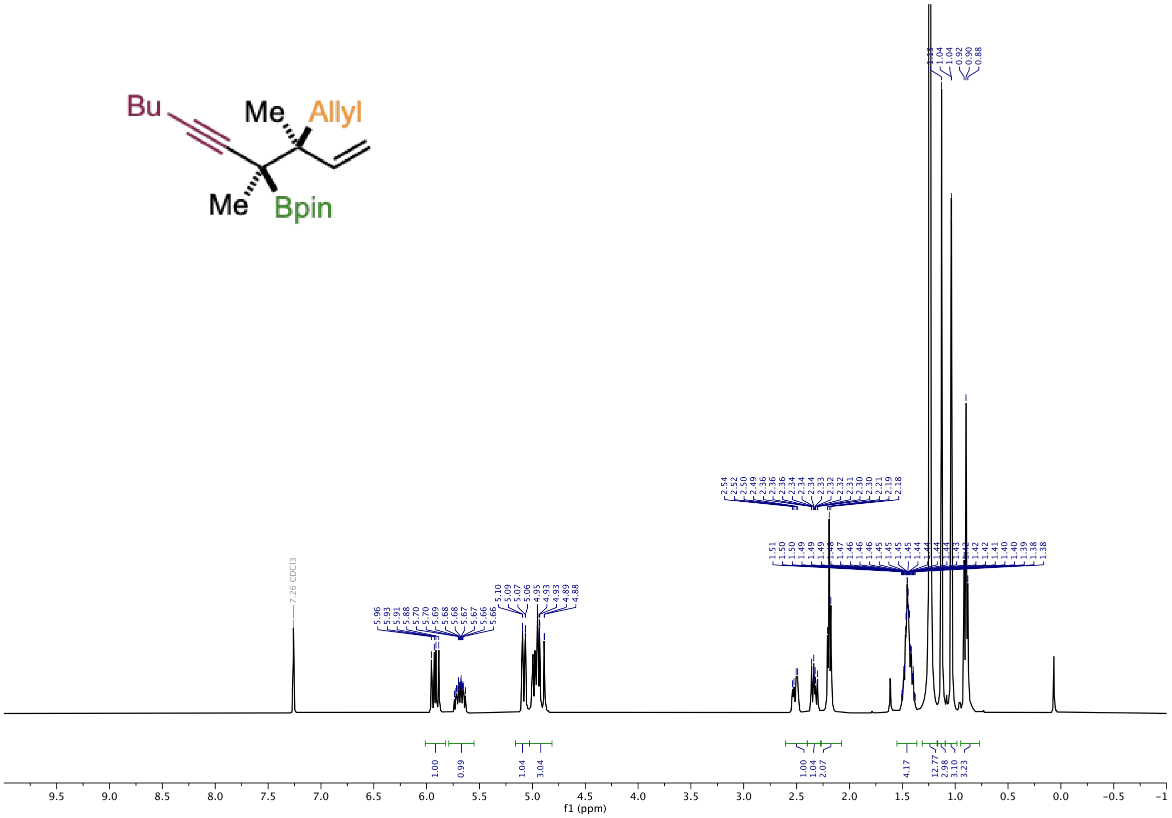

^{1}H NMR spectrum (400 MHz, $CDCl_3$)

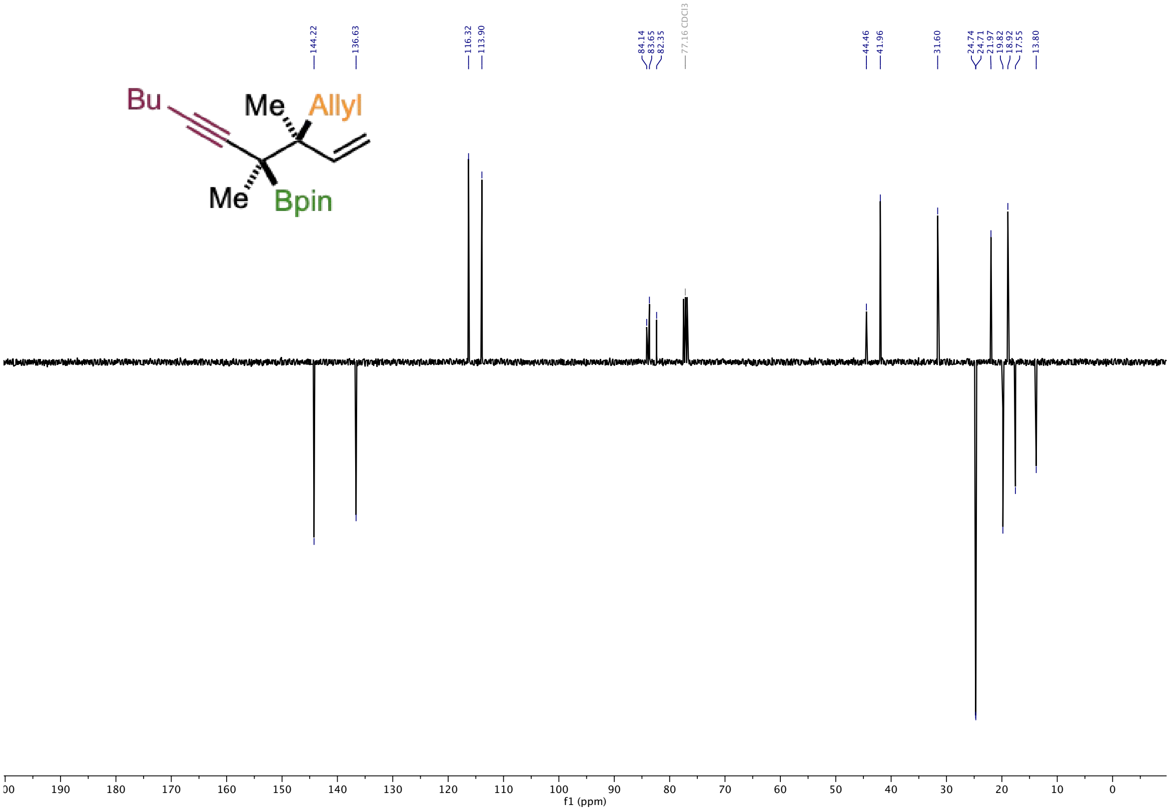

^{13}C NMR (APT) spectrum (101 MHz, $CDCl_3$)

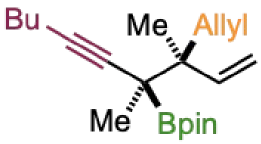

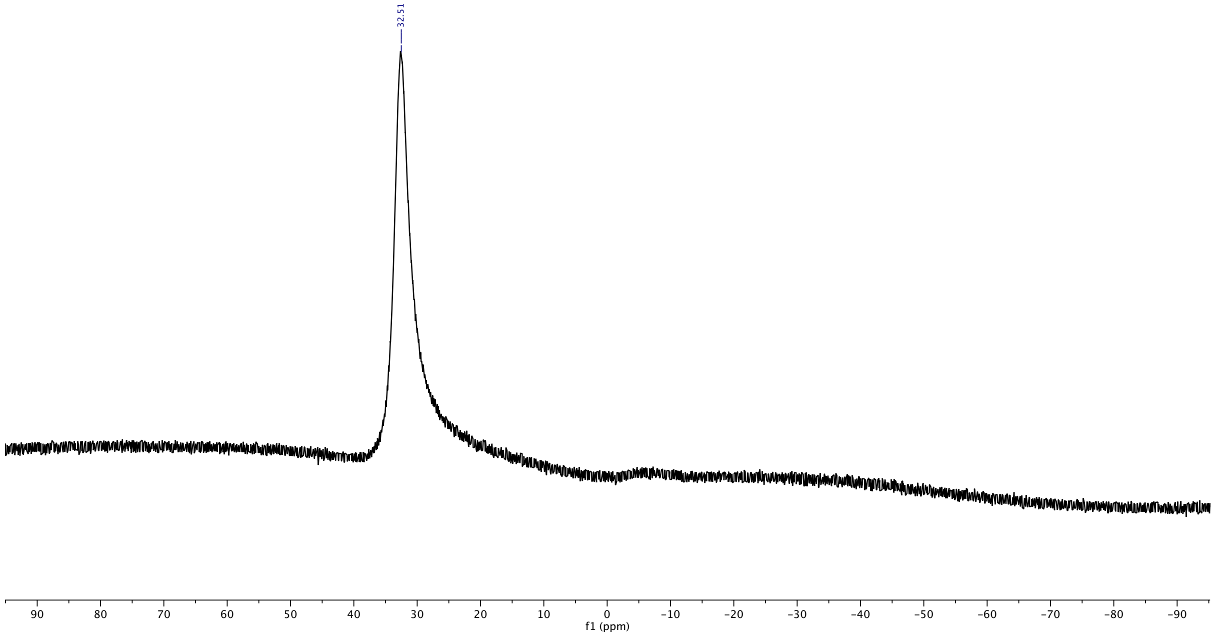

^{11}B NMR spectrum (128 MHz, $CDCl_3$)

2-((3*R,4*S**)-3,4-Dimethyl-7,7-dimethyloct-1-en-5-yn-4-yl)-4,4,5,5-tetramethyl-1,3,2-dioxaborolane (6ab)**

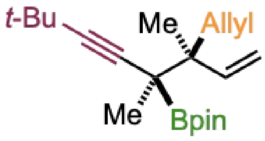

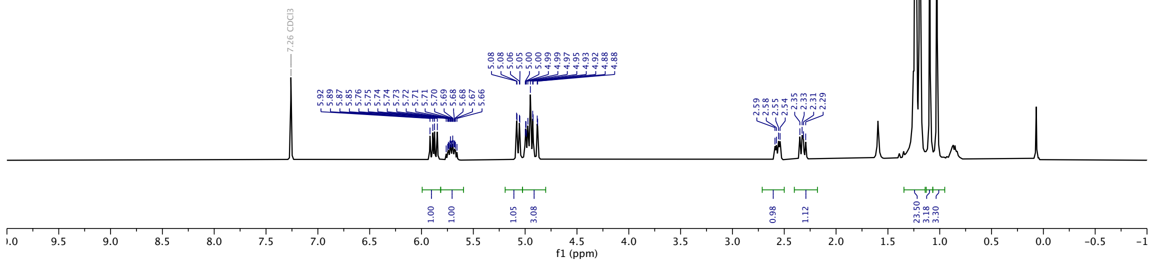

¹H NMR spectrum (400 MHz, CDCl₃)

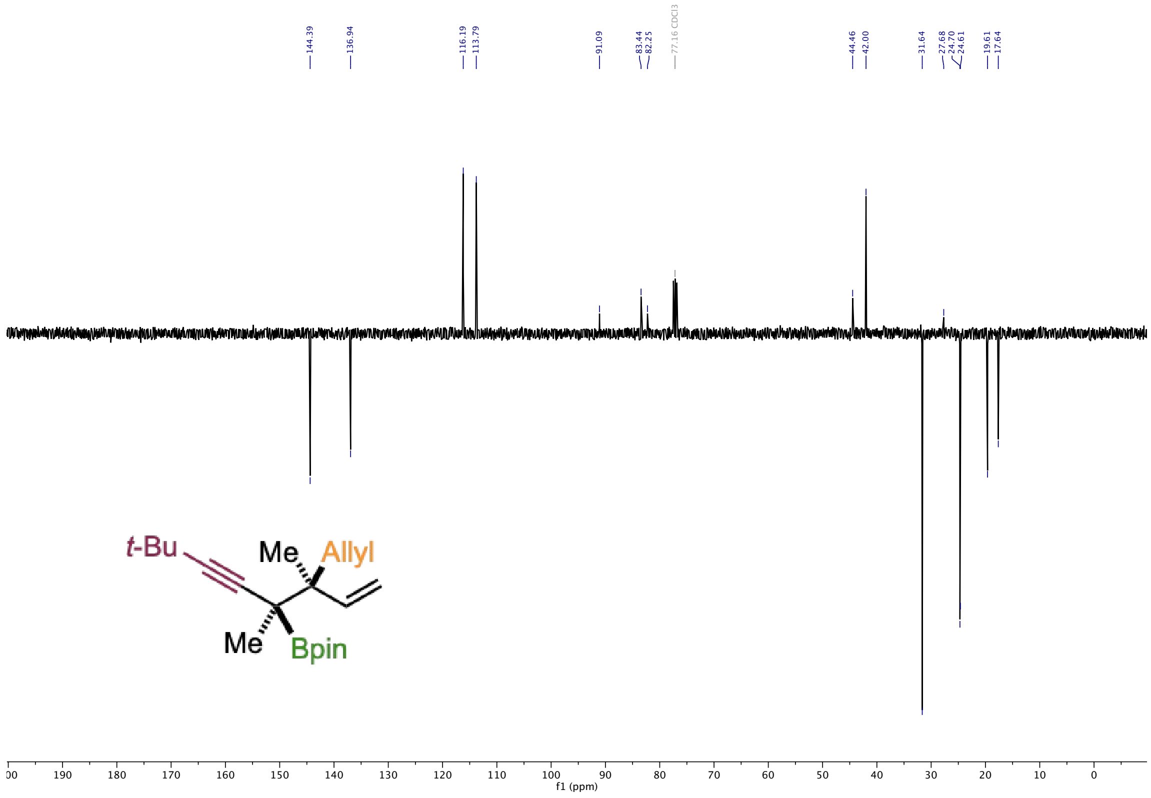

¹³C NMR (APT) spectrum (101 MHz, CDCl₃)

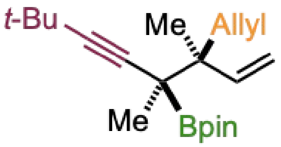

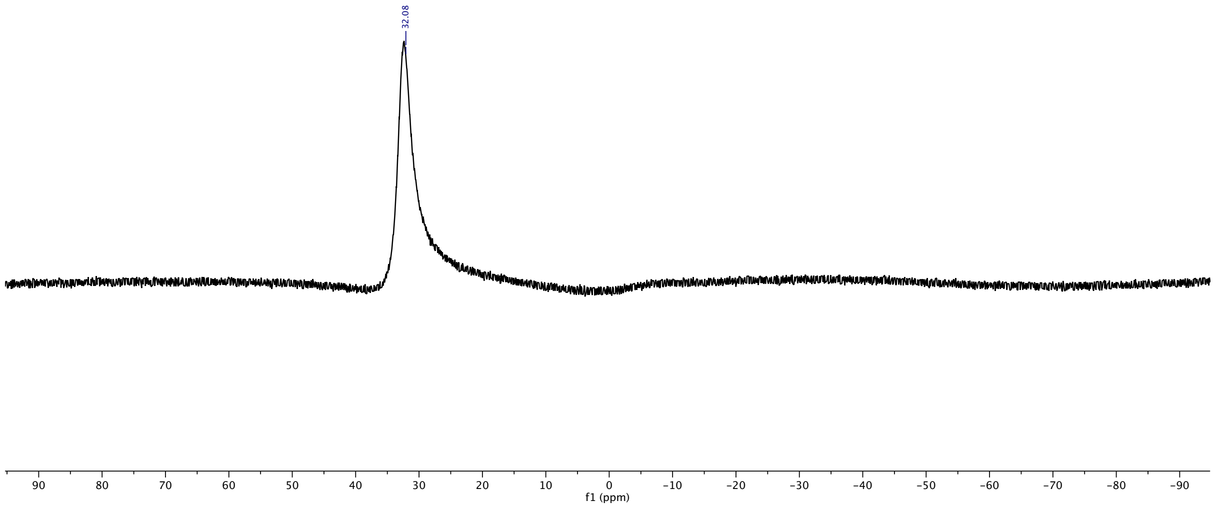

^{11}B NMR spectrum (128 MHz, $CDCl_3$)

2-((3*S**,4*S**)-1-Cyclohexyl-3,4-dimethyl-4-vinylhept-6-en-1-yn-3-yl)-4,4,5,5-tetramethyl-1,3,2-dioxaborolane (6ac)

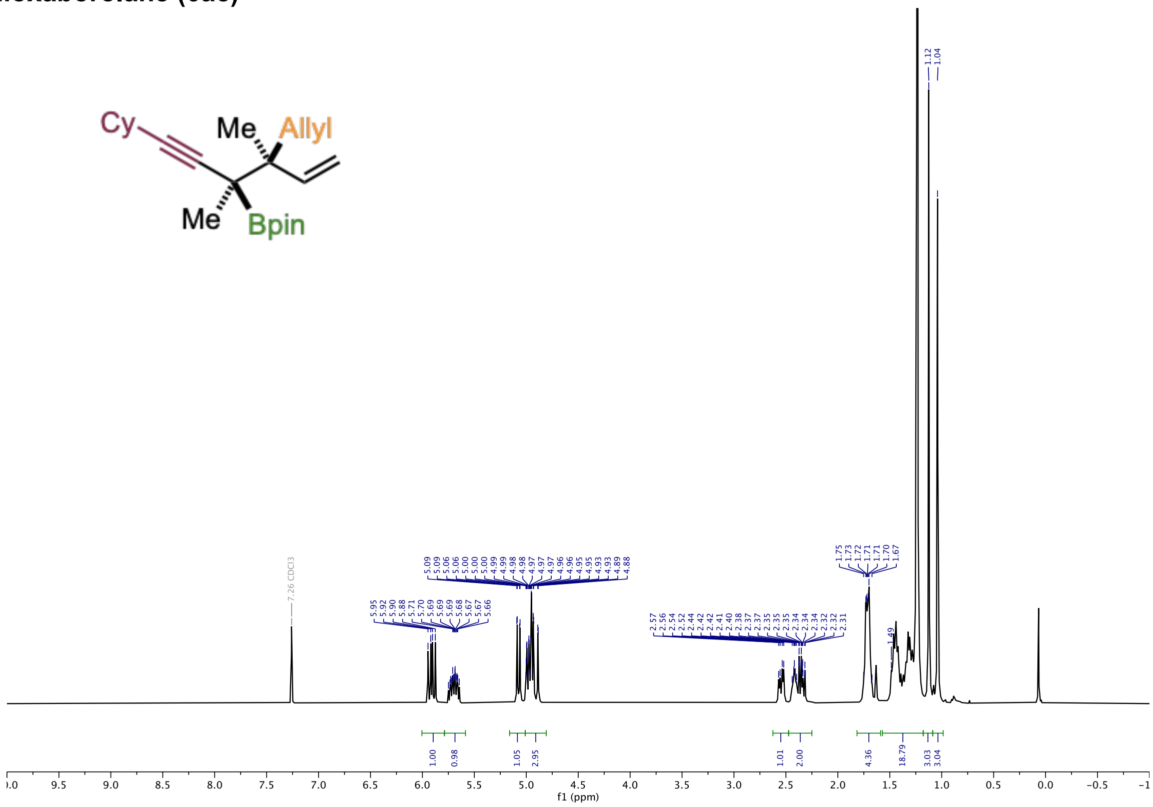

^{1}H NMR spectrum (400 MHz, $CDCl_3$)

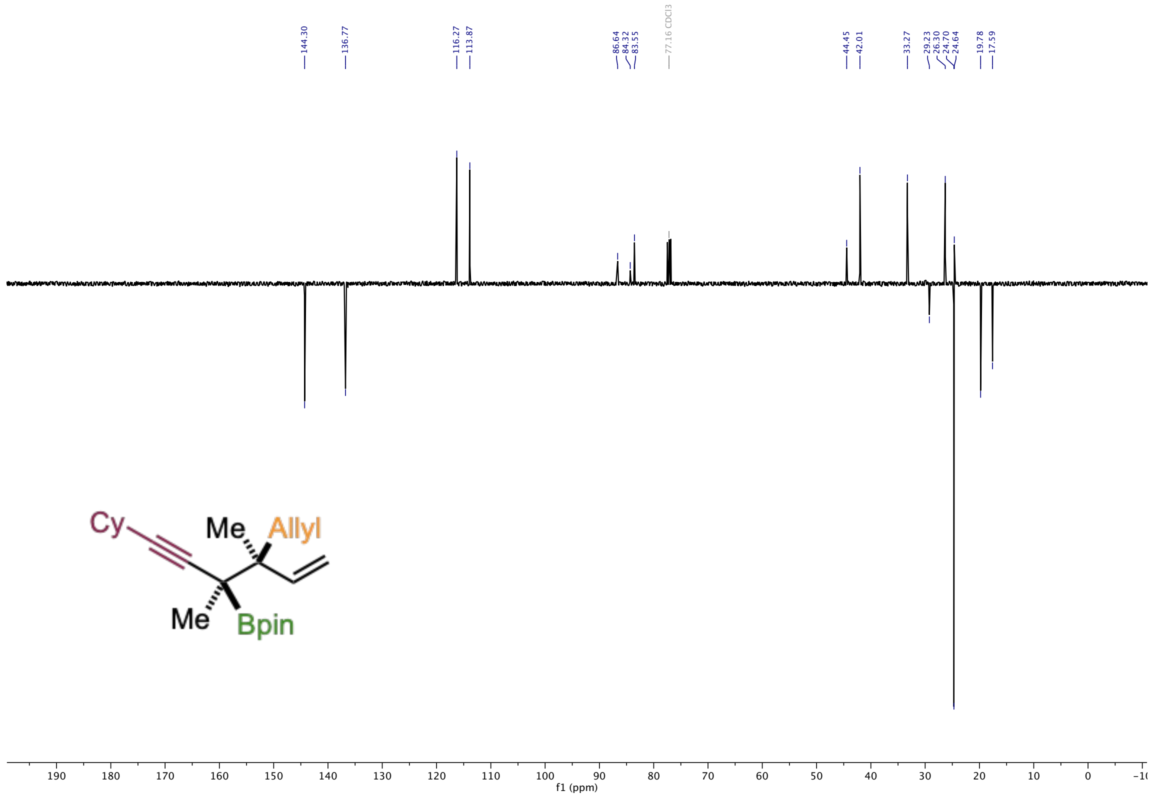

^{13}C NMR (APT) spectrum (101 MHz, $CDCl_3$)

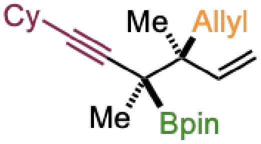

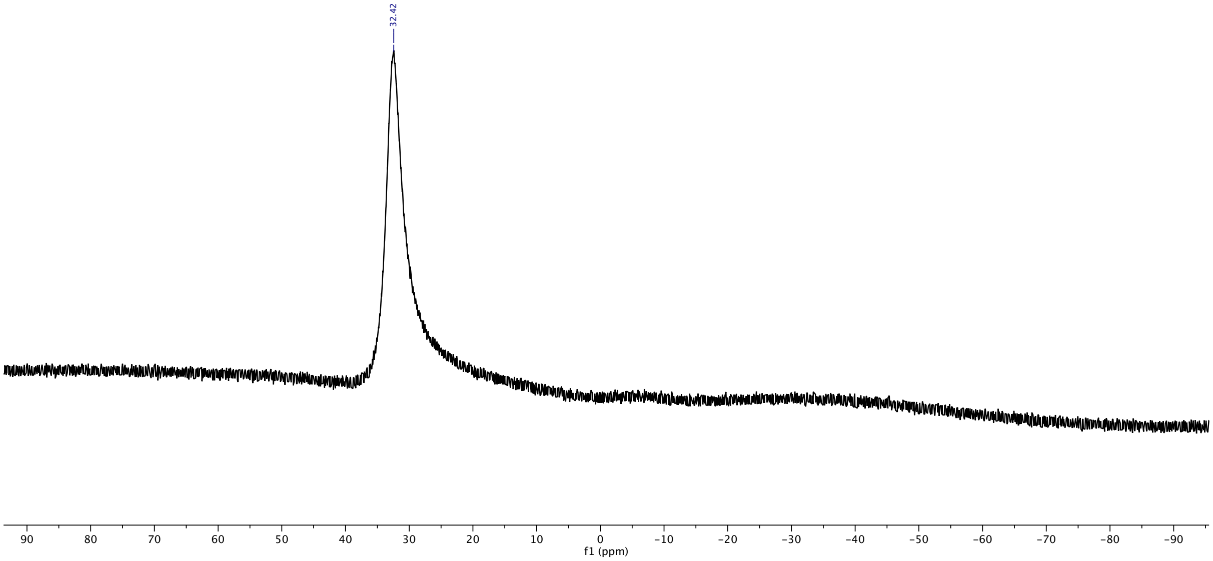

^{11}B NMR spectrum (128 MHz, $CDCl_3$)

2-((3*S,4*S**)-3,4-Dimethyl-1-phenyl-4-vinylhept-6-en-1-yn-3-yl)-4,4,5,5-tetramethyl-1,3,2-dioxaborolane (6ad)**

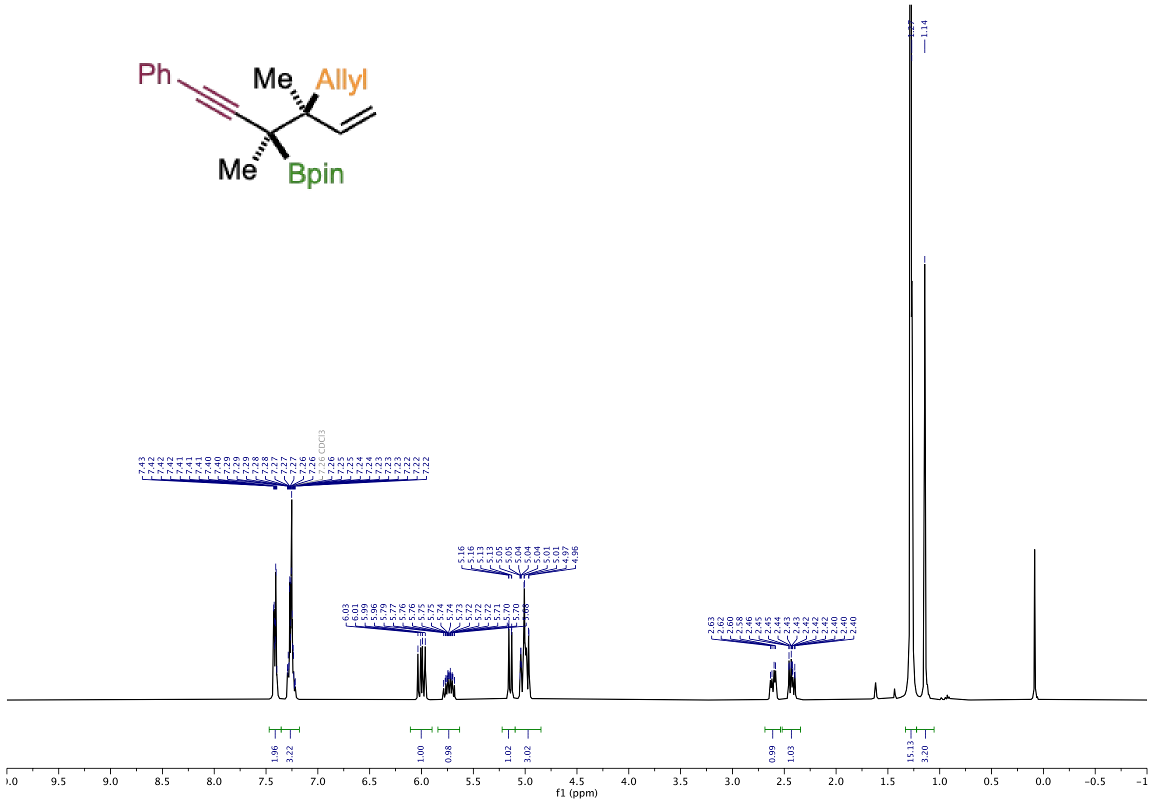

^{1}H NMR spectrum (400 MHz, $CDCl_3$)

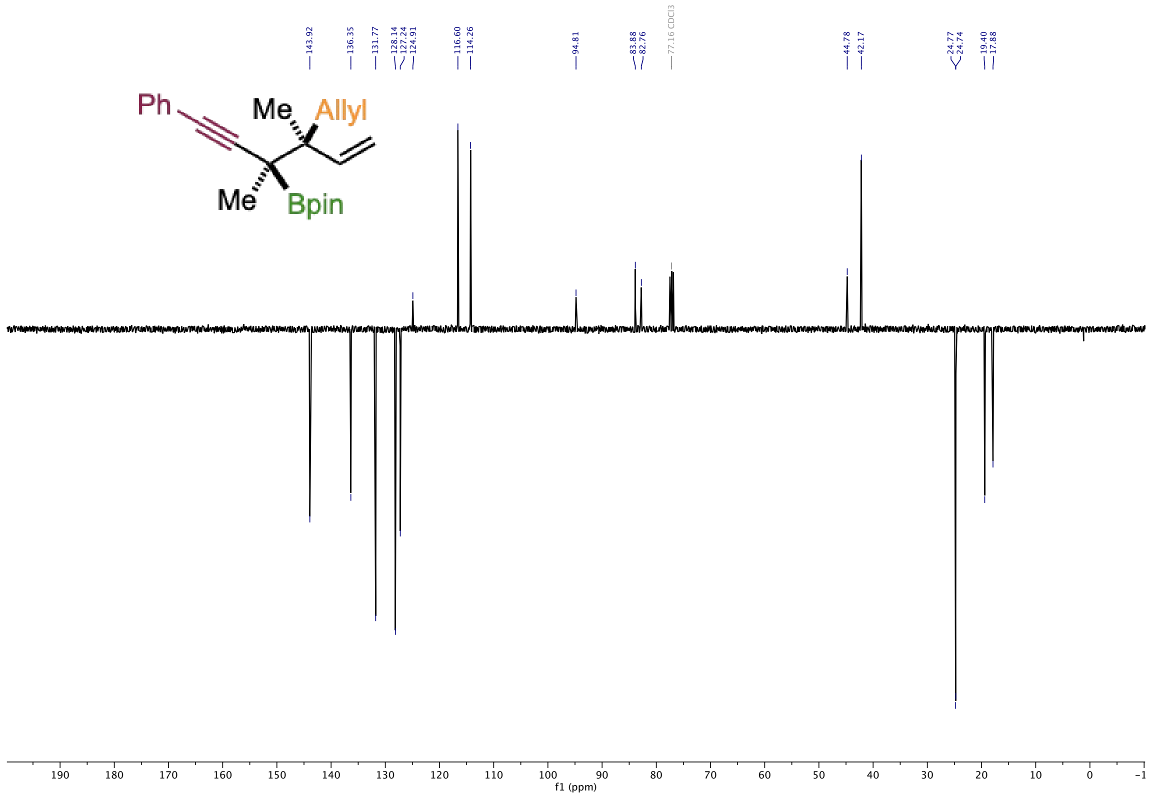

^{13}C NMR (APT) spectrum (101 MHz, $CDCl_3$)

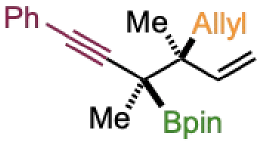

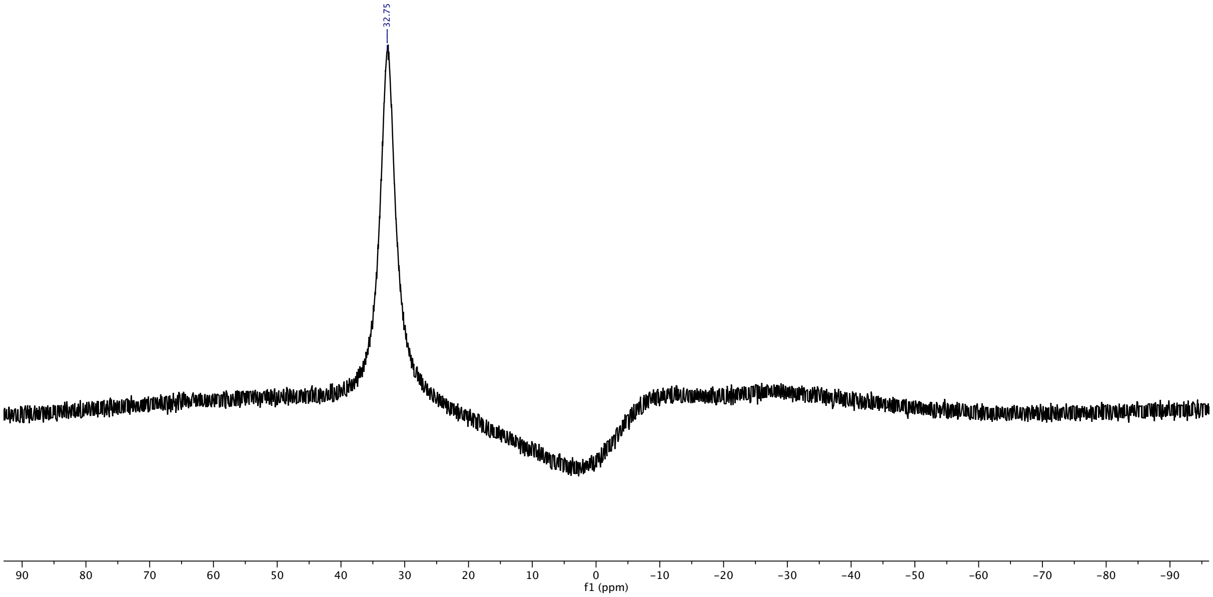

^{11}B NMR spectrum (128 MHz, $CDCl_3$)

2-((3*S,4*S**)-3,4-Dimethyl-1-triisopropylsilyl-4-vinylhept-6-en-1-yn-3-yl)-4,4,5,5-tetramethyl-1,3,2-dioxaborolane (6ae)**

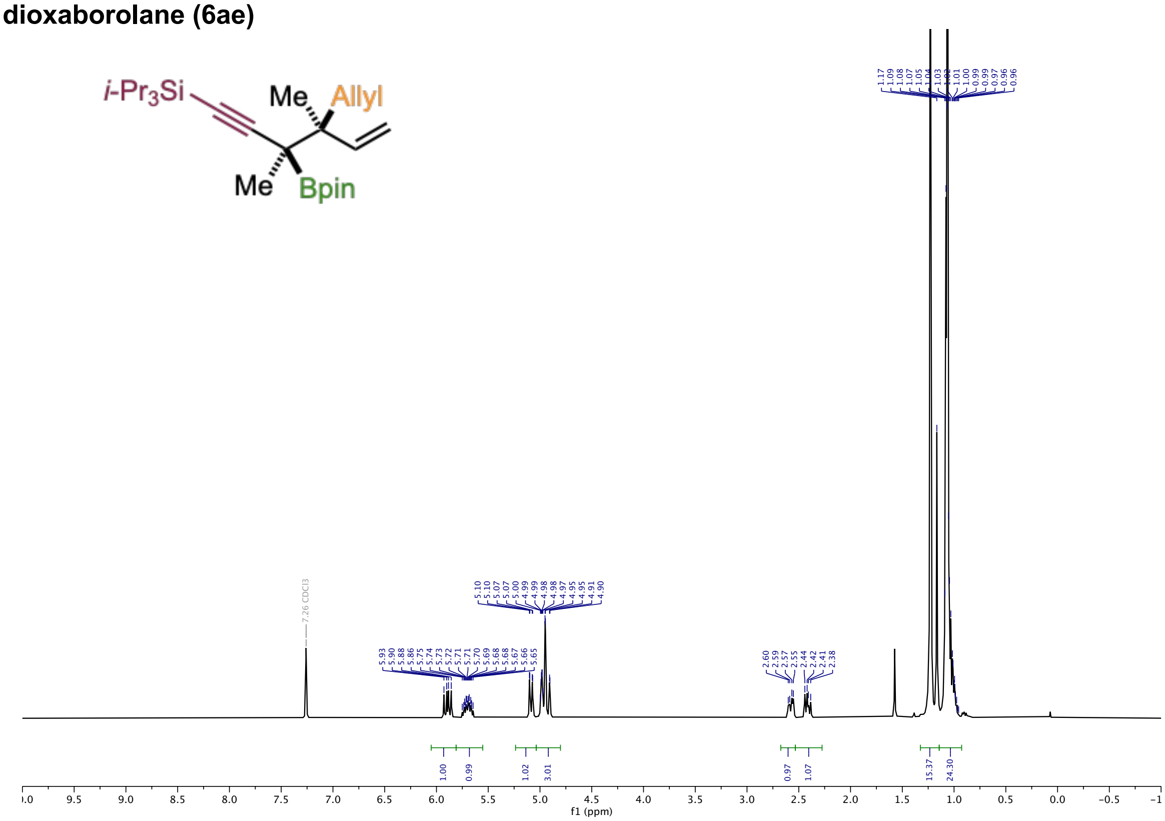

^{1}H NMR spectrum (400 MHz, $CDCl_3$)

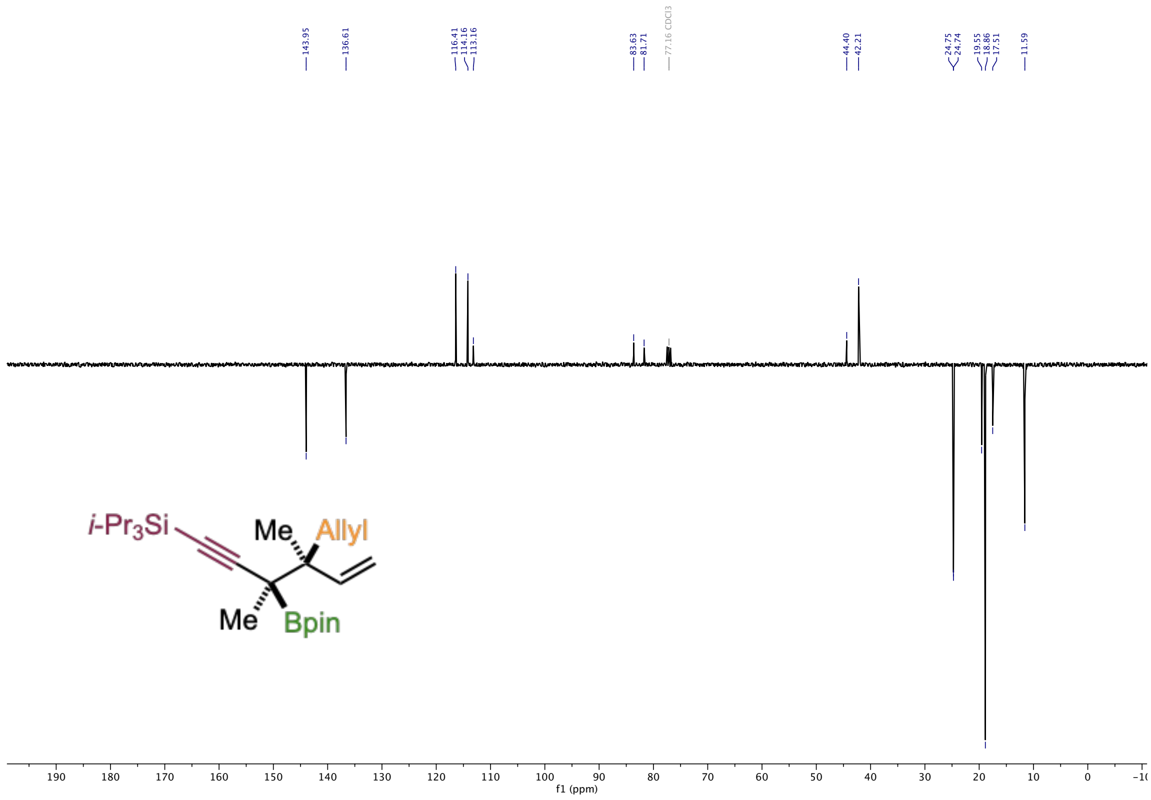

^{13}C NMR (APT) spectrum (101 MHz, $CDCl_3$)

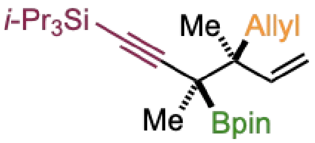

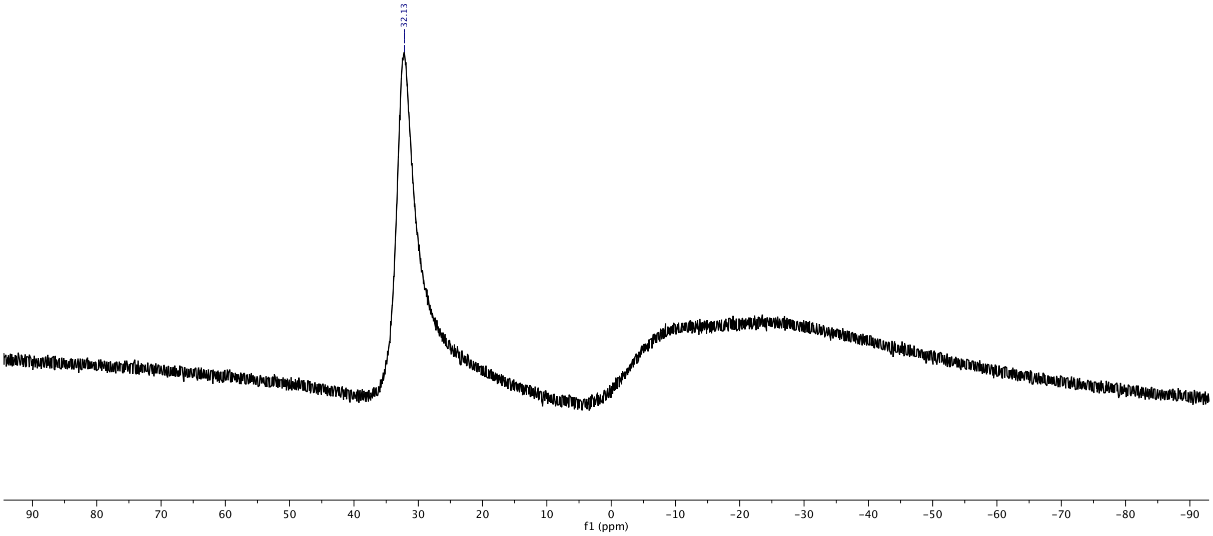

^{11}B NMR spectrum (128 MHz, $CDCl_3$)

5-Allylundec-6-yn-5-ol (8a)

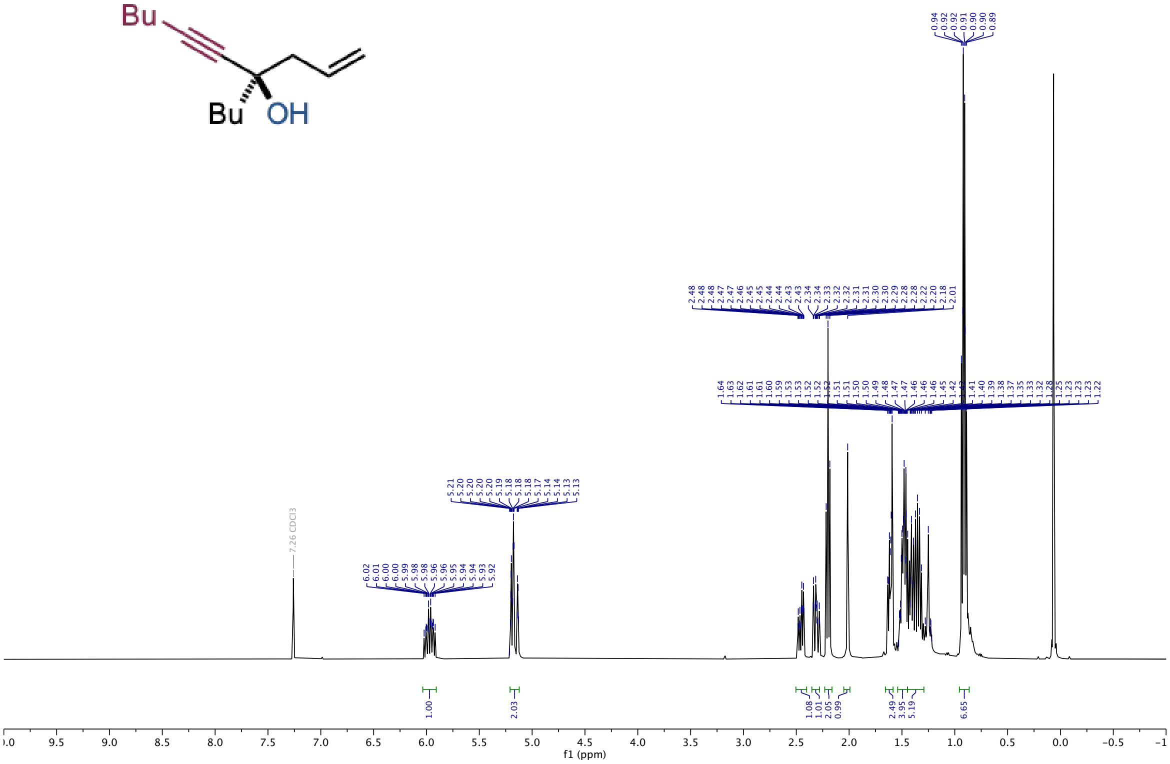

^{1}H NMR spectrum (400 MHz, $CDCl_3$)

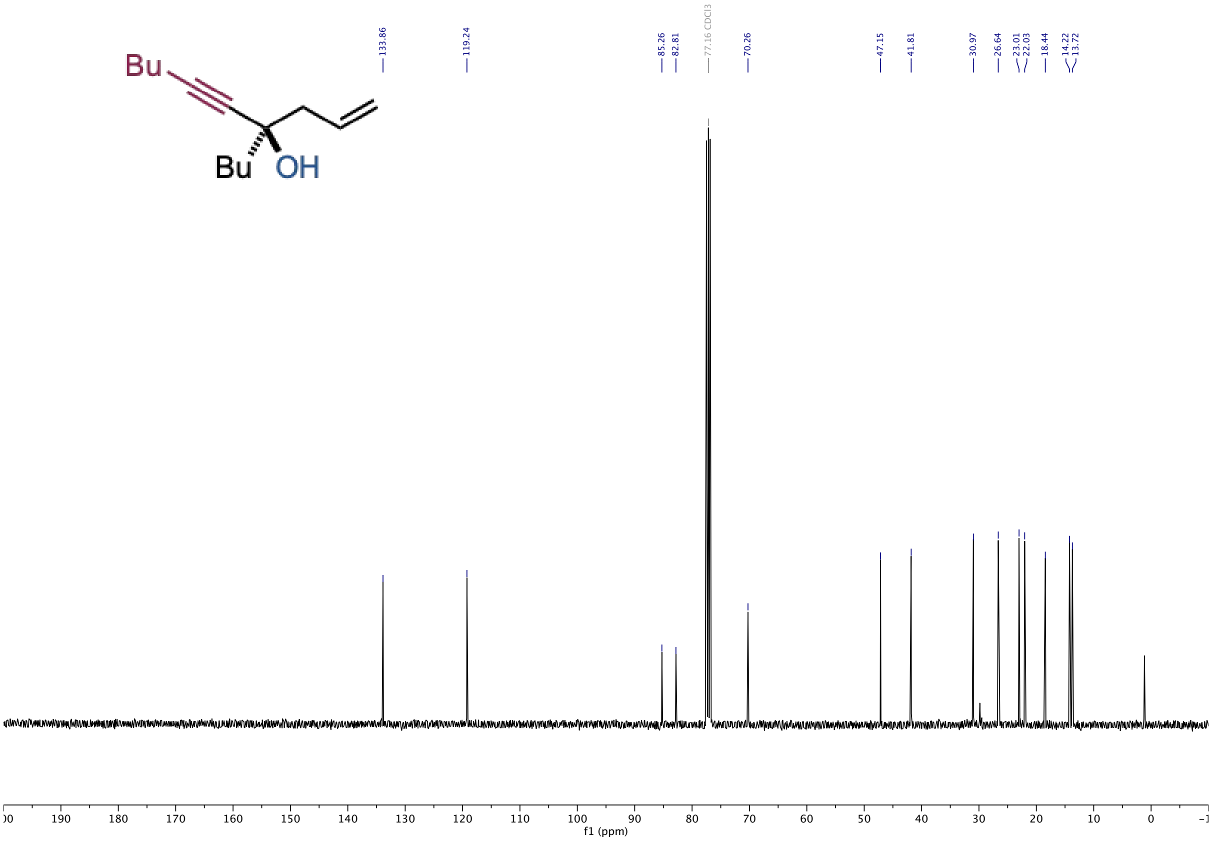

^{13}C NMR spectrum (101 MHz, $CDCl_3$)

4-(Phenylethynyl)oct-1-en-4-ol (8b)

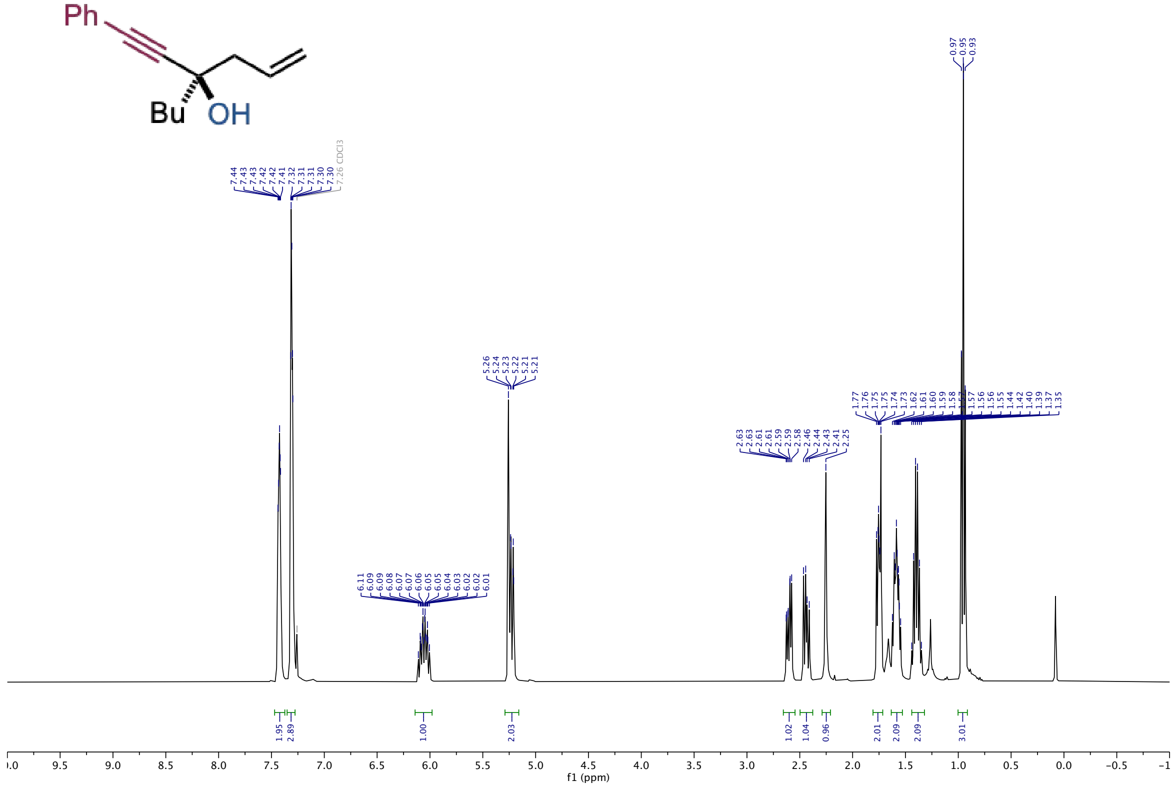

^{1}H NMR spectrum (400 MHz, CDCl$_3$)

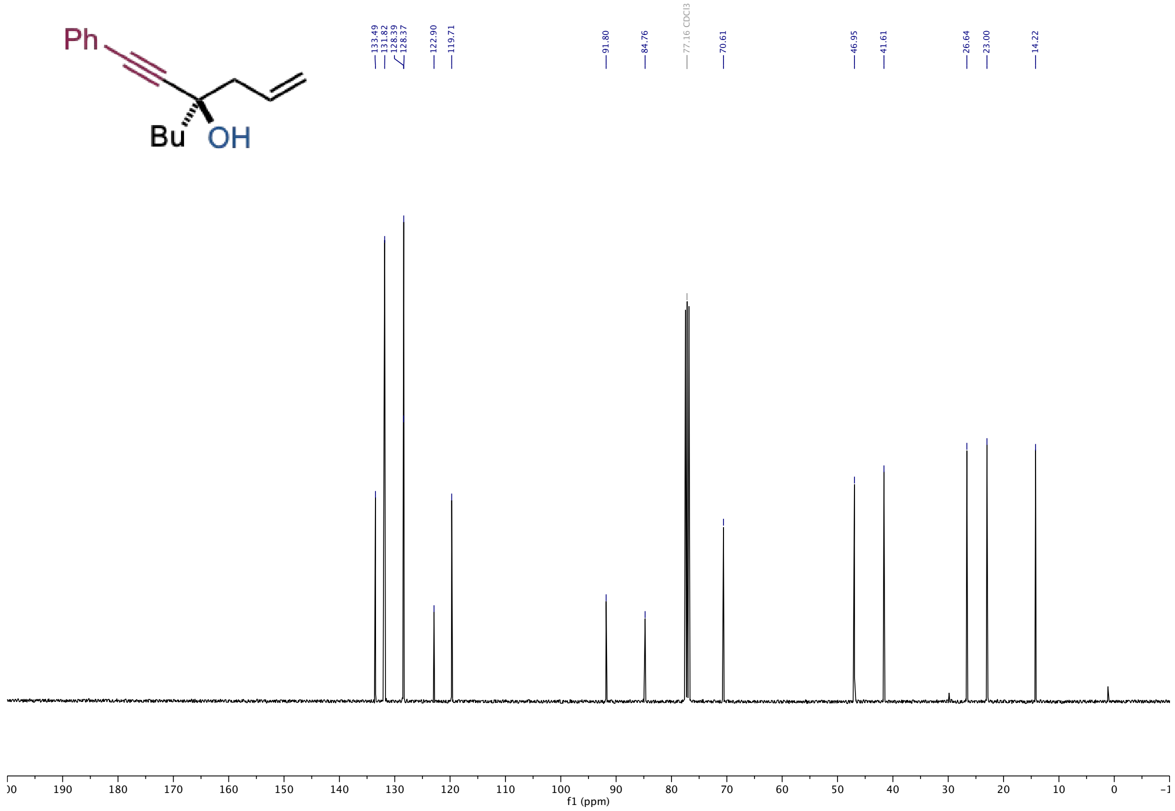

^{13}C NMR spectrum (101 MHz, CDCl$_3$)

4-((Trimethylsilyl)ethynyl)oct-1-en-4-ol (8c)

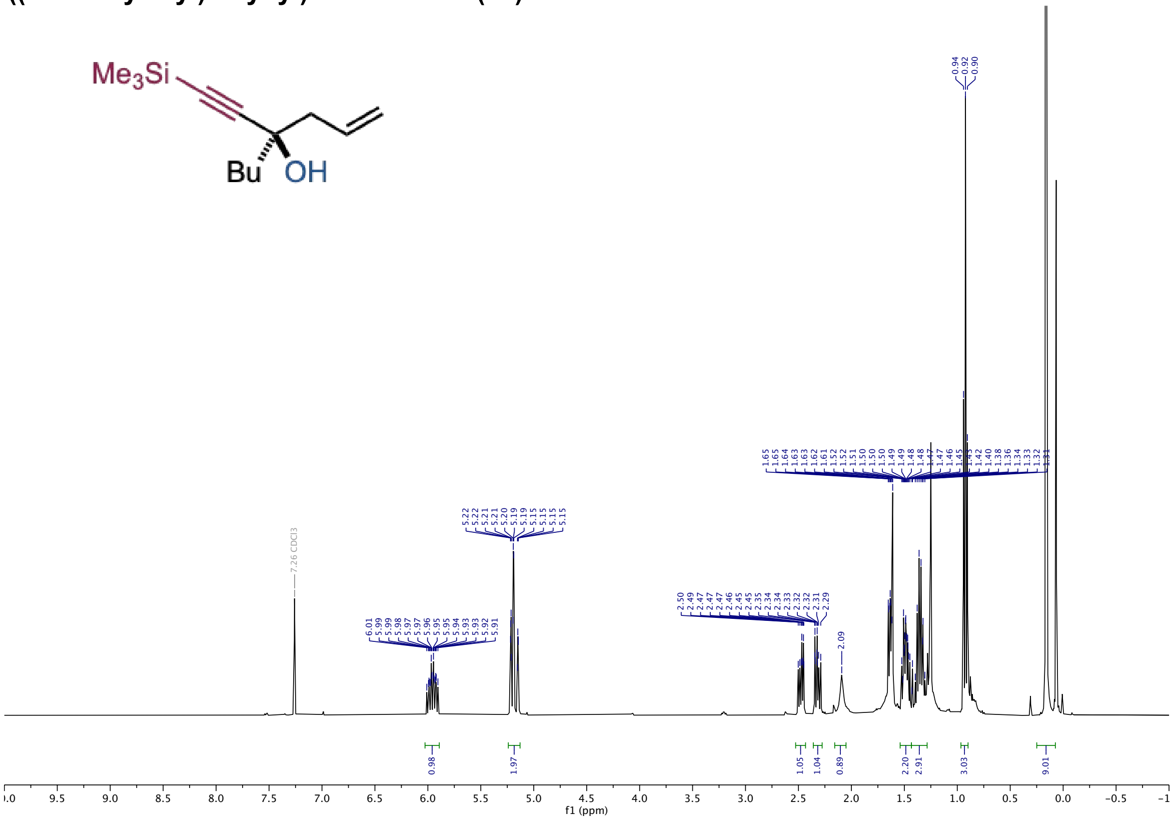

^{1}H NMR spectrum (400 MHz, $CDCl_3$)

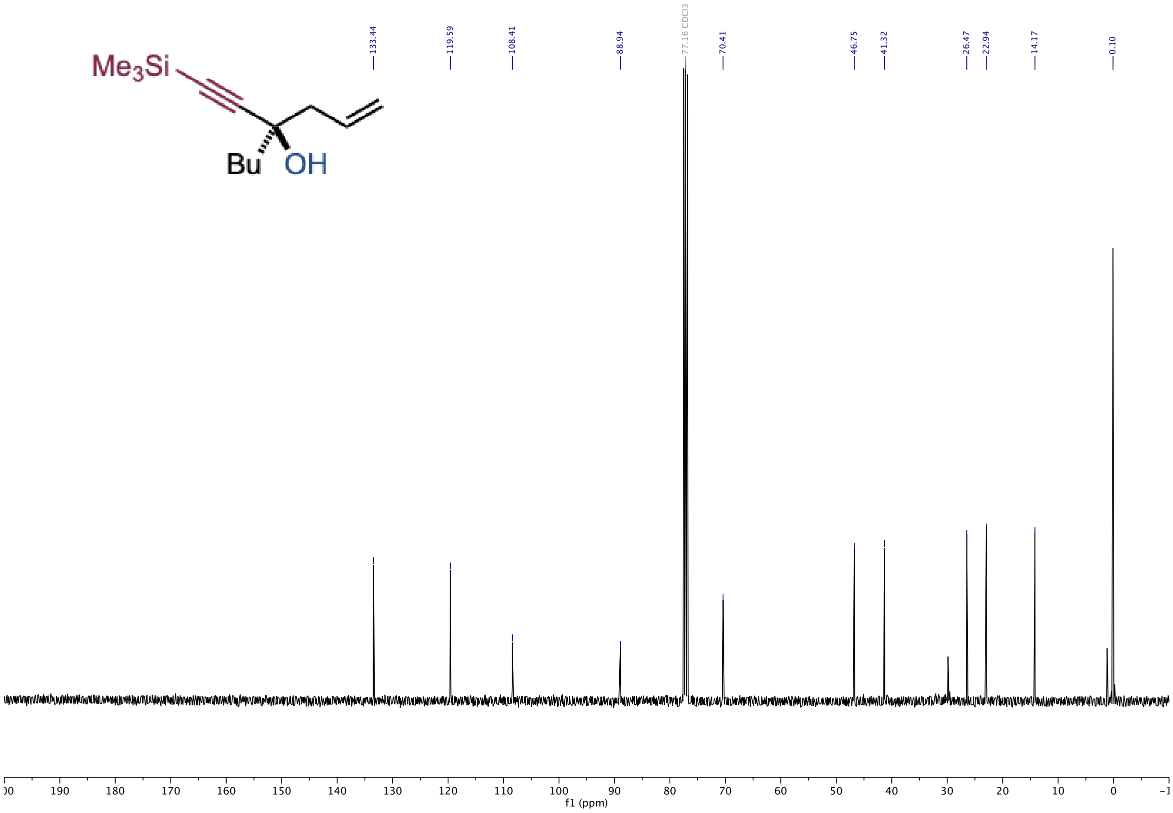

^{13}C NMR spectrum (101 MHz, $CDCl_3$)

4-((Triisopropylsilyl)ethynyl)oct-1-en-4-ol (8d)

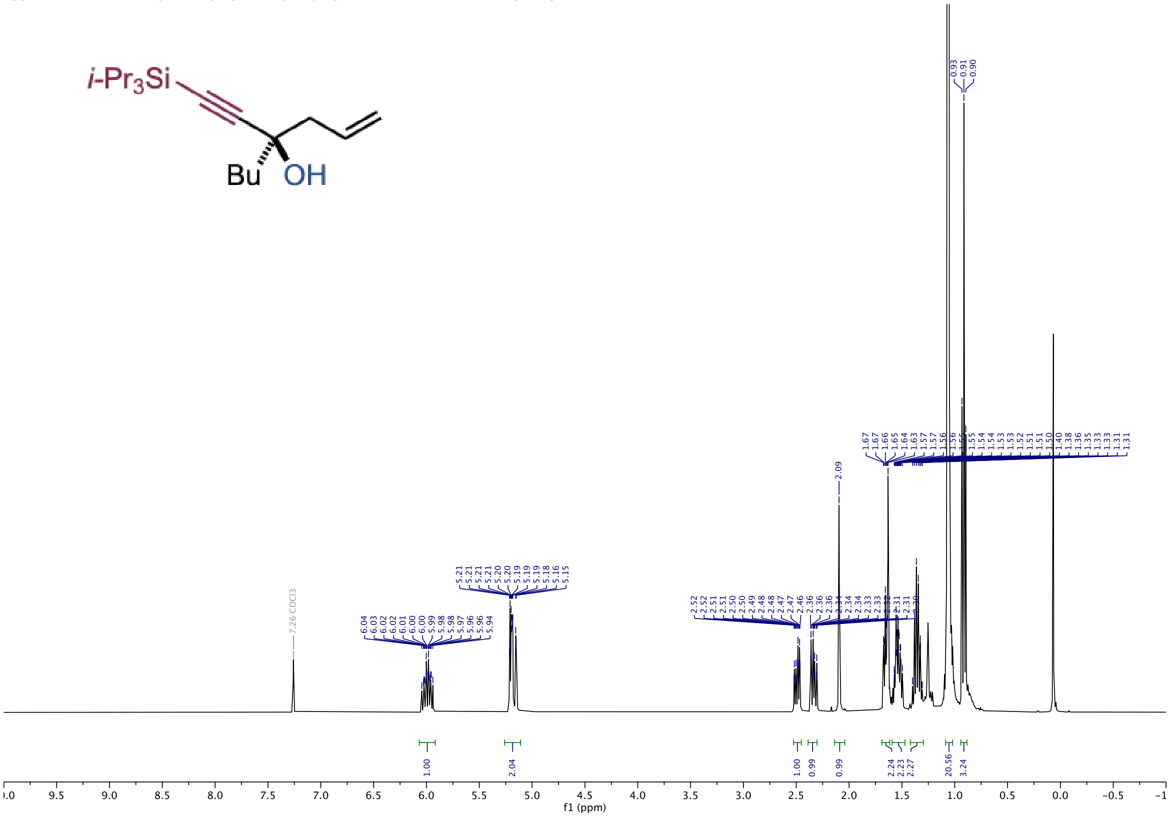

^{1}H NMR spectrum (400 MHz, $CDCl_3$)

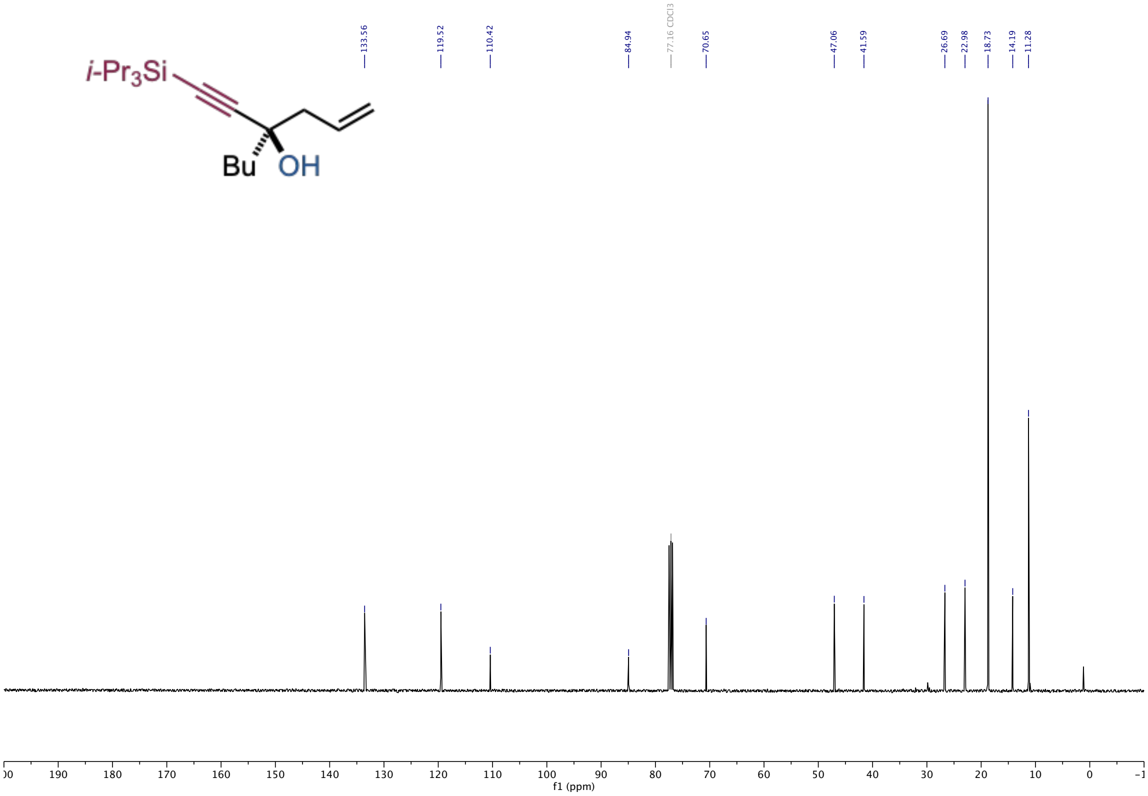

^{13}C NMR spectrum (101 MHz, $CDCl_3$)

4-((4-(Trifluoromethyl)phenyl)ethynyl)oct-1-en-4-ol (8e)

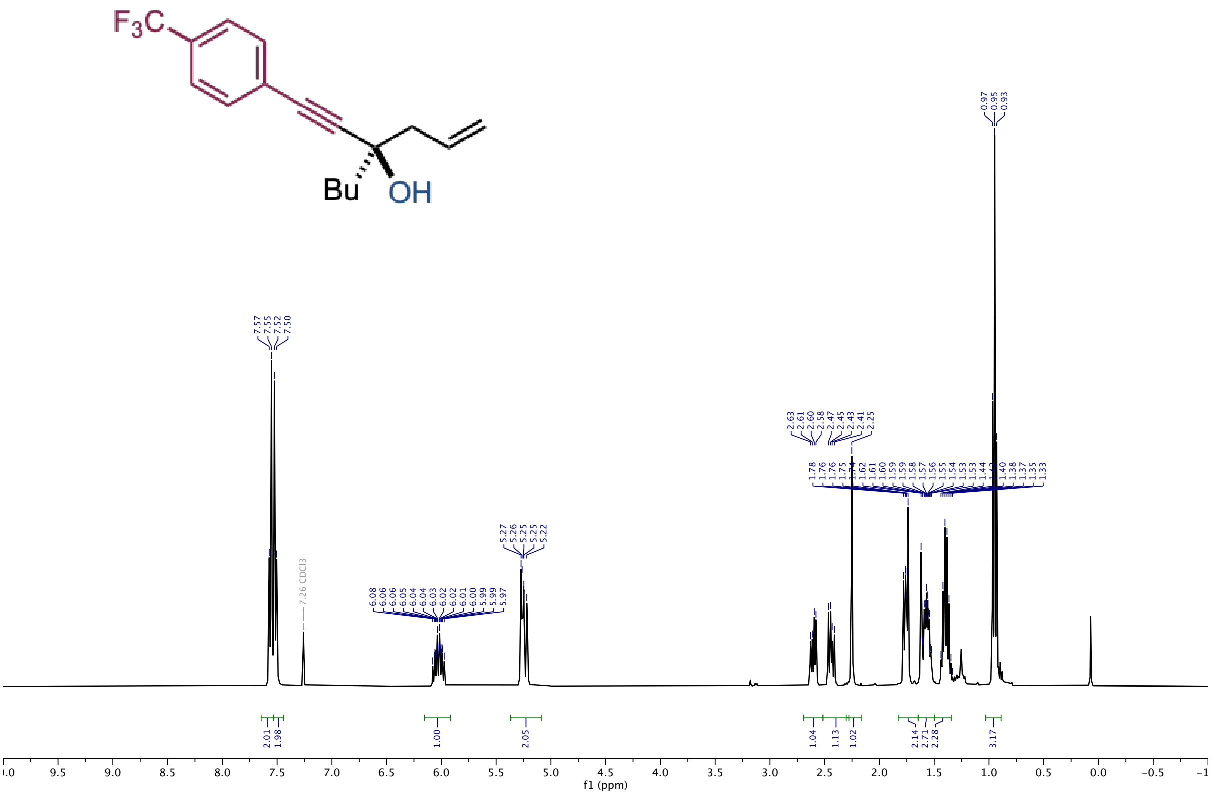

¹H NMR spectrum (400 MHz, $CDCl_3$)

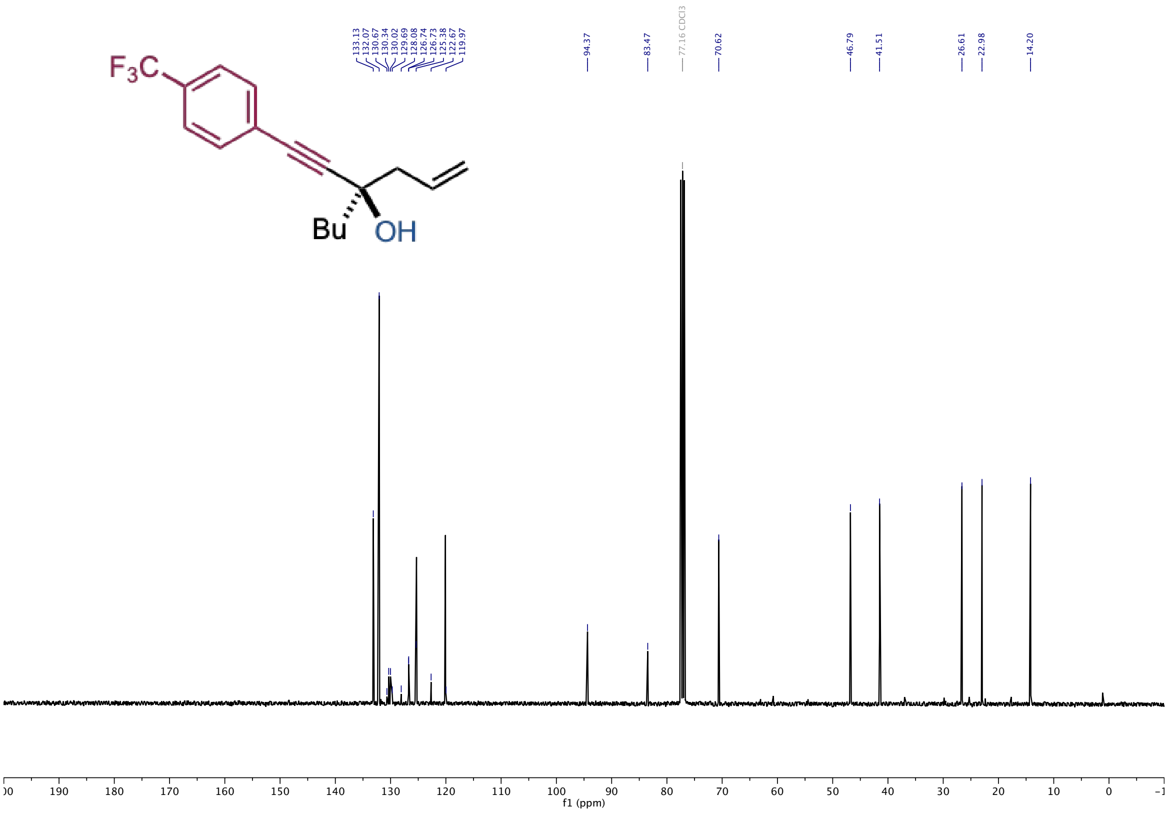

¹³C NMR spectrum (101 MHz, $CDCl_3$)

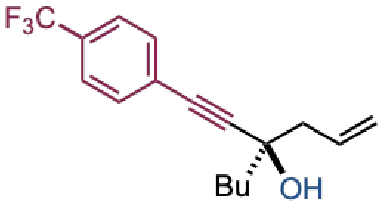

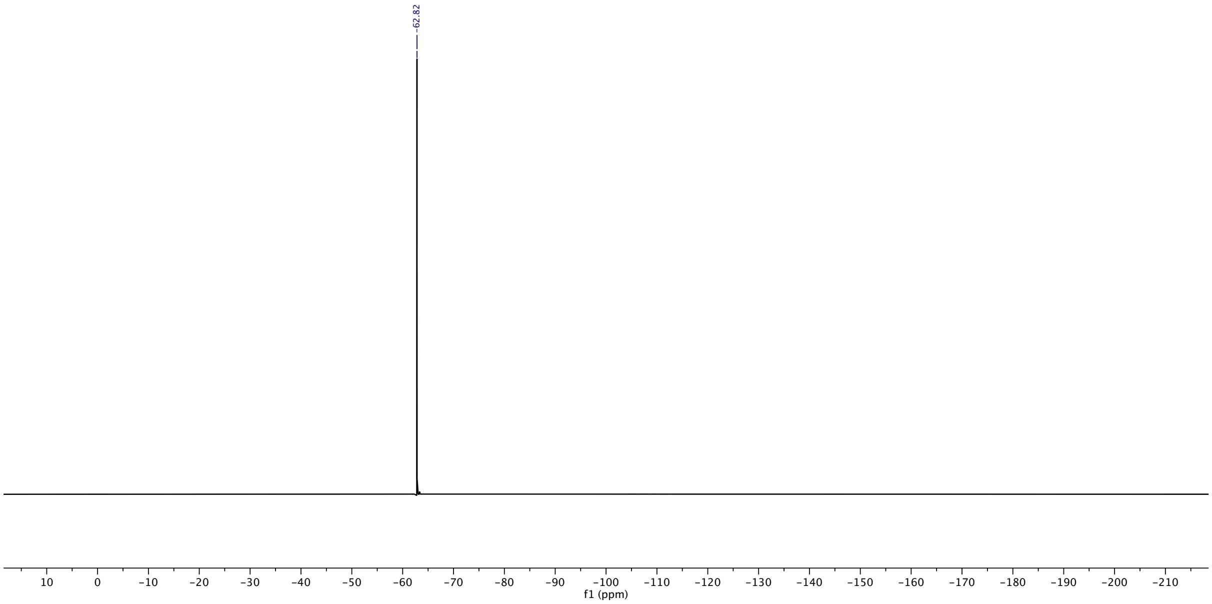

^{19}F NMR spectrum (377 MHz, $CDCl_3$)

1-(3-Allyl-hepta-1,2-dien-1-yl)-4-(trifluoromethyl)benzene (S2)

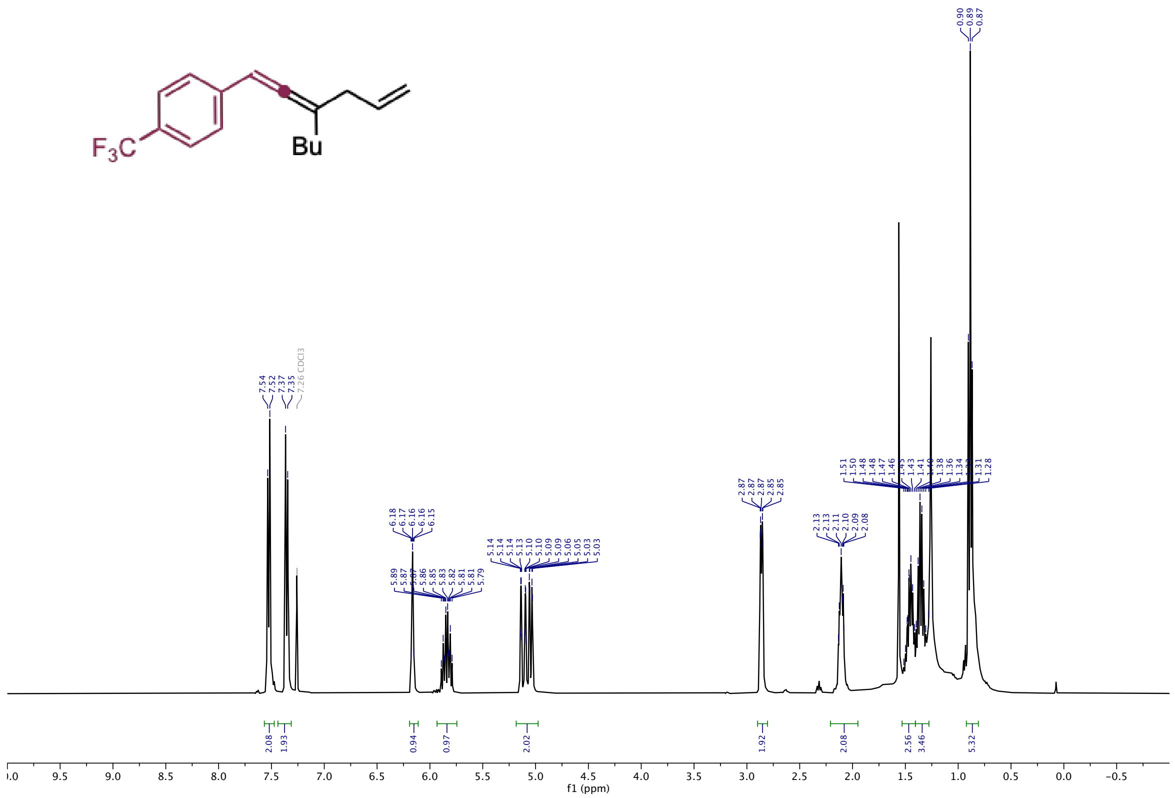

¹H NMR spectrum (400 MHz, $CDCl_3$)

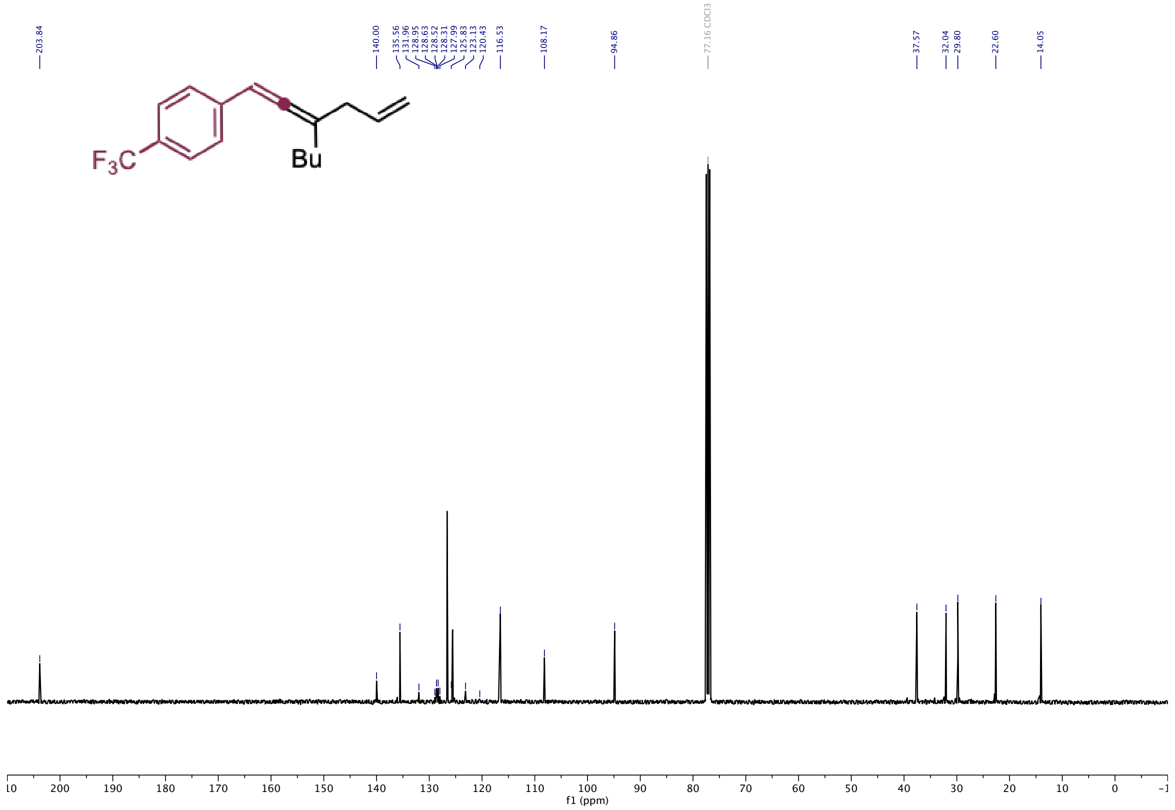

¹³C NMR spectrum (101 MHz, $CDCl_3$)

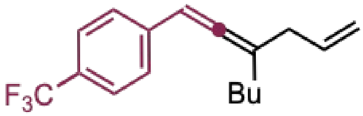

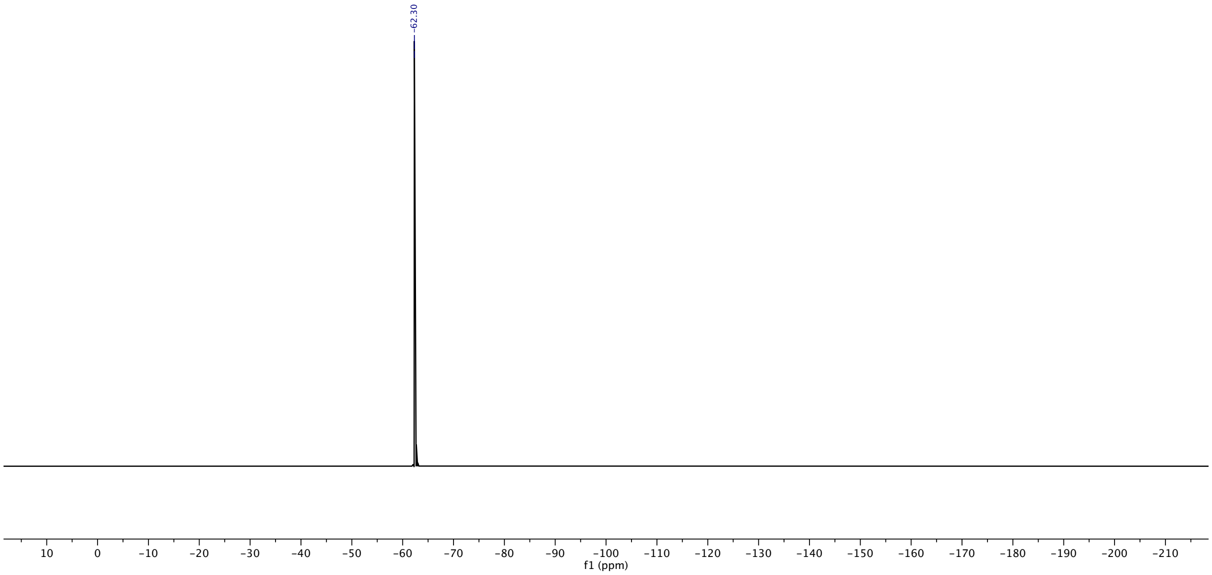

19F NMR spectrum (377 MHz, $CDCl_3$)

3,4-Diethyldec-1-en-5-yn-4-ol (8f)

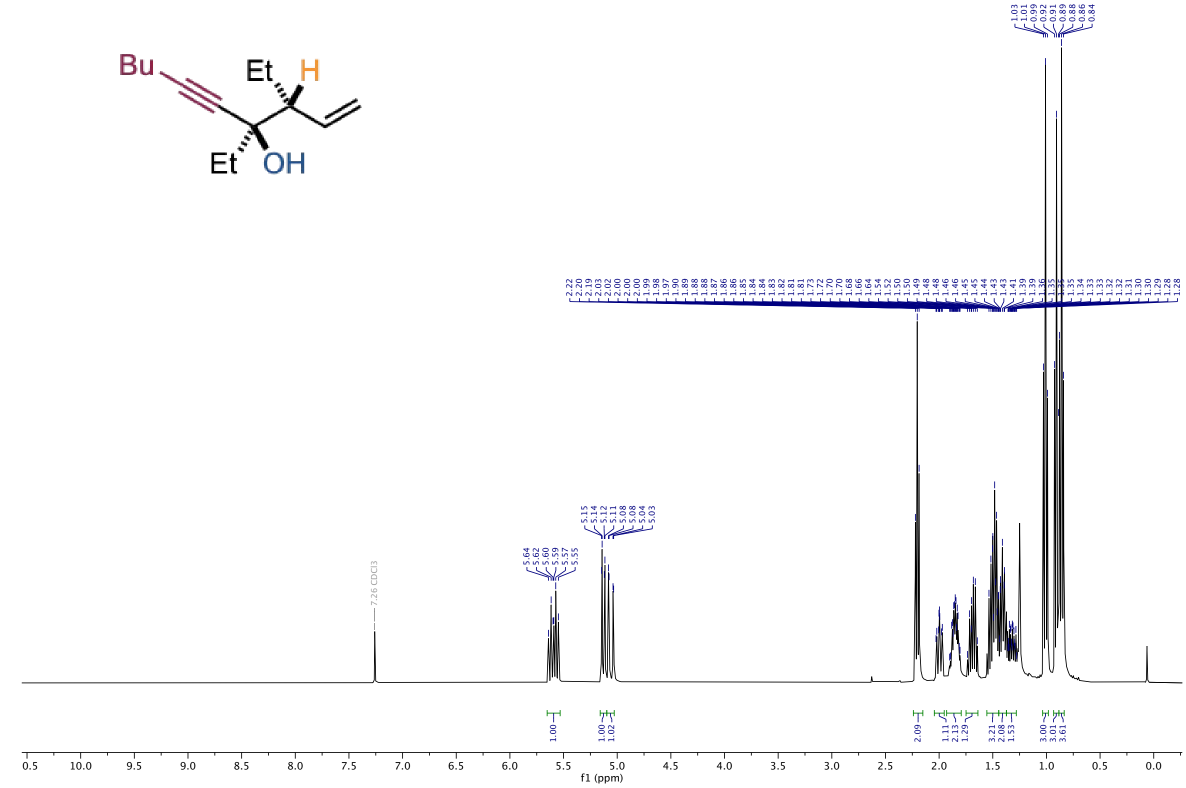

^{1}H NMR spectrum (400 MHz, $CDCl_3$)

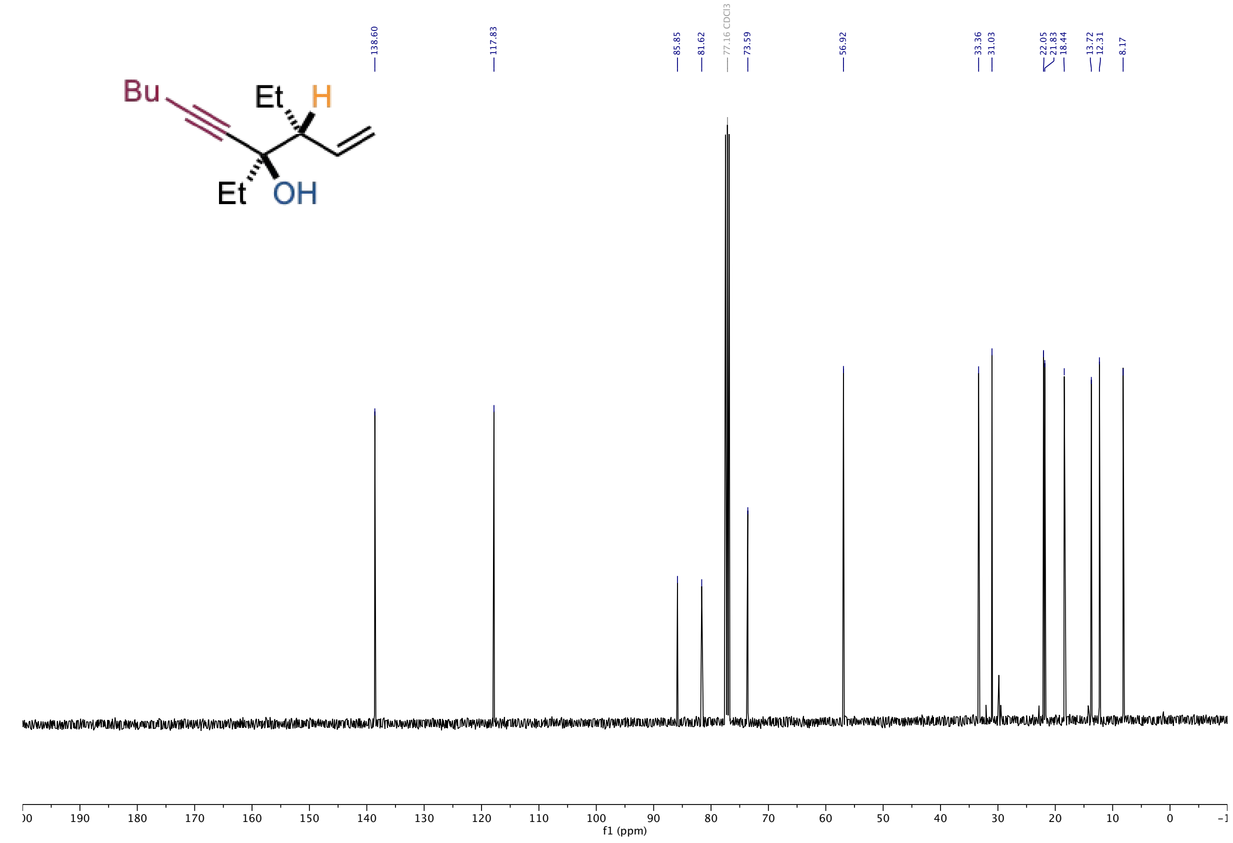

^{13}C NMR spectrum (101 MHz, $CDCl_3$)

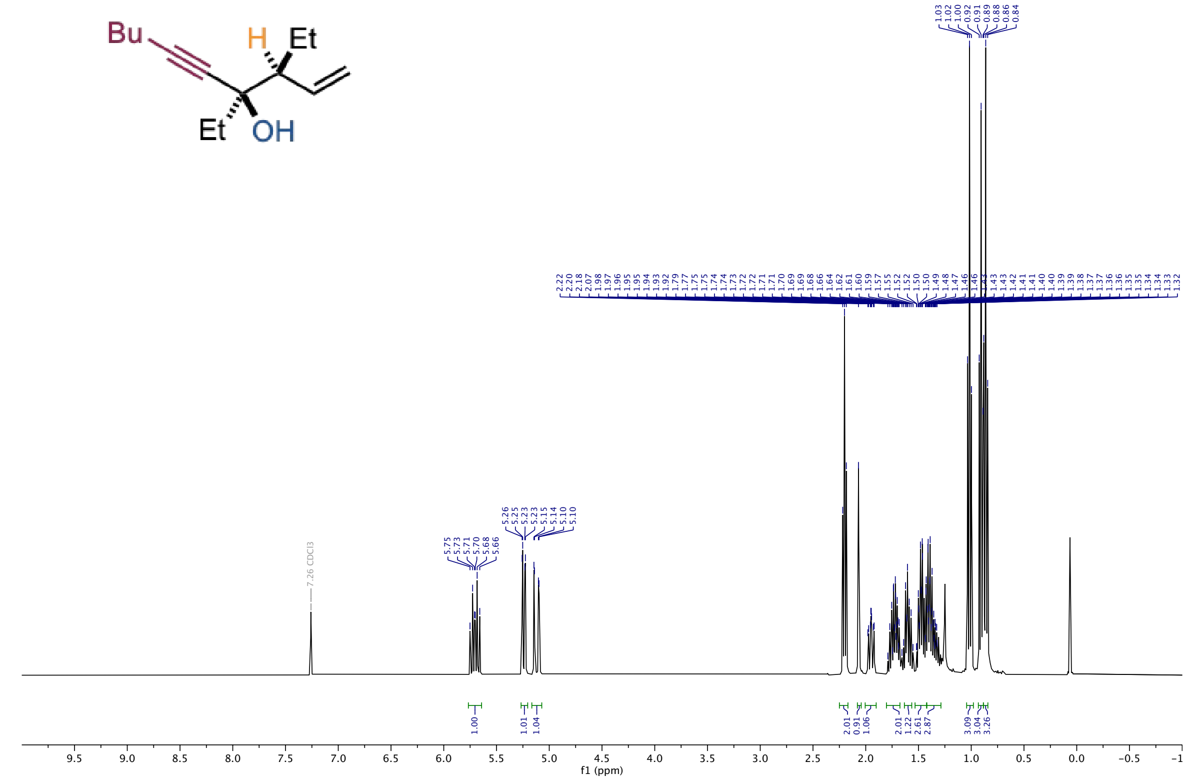

¹H NMR spectrum (400 MHz, $CDCl_3$)

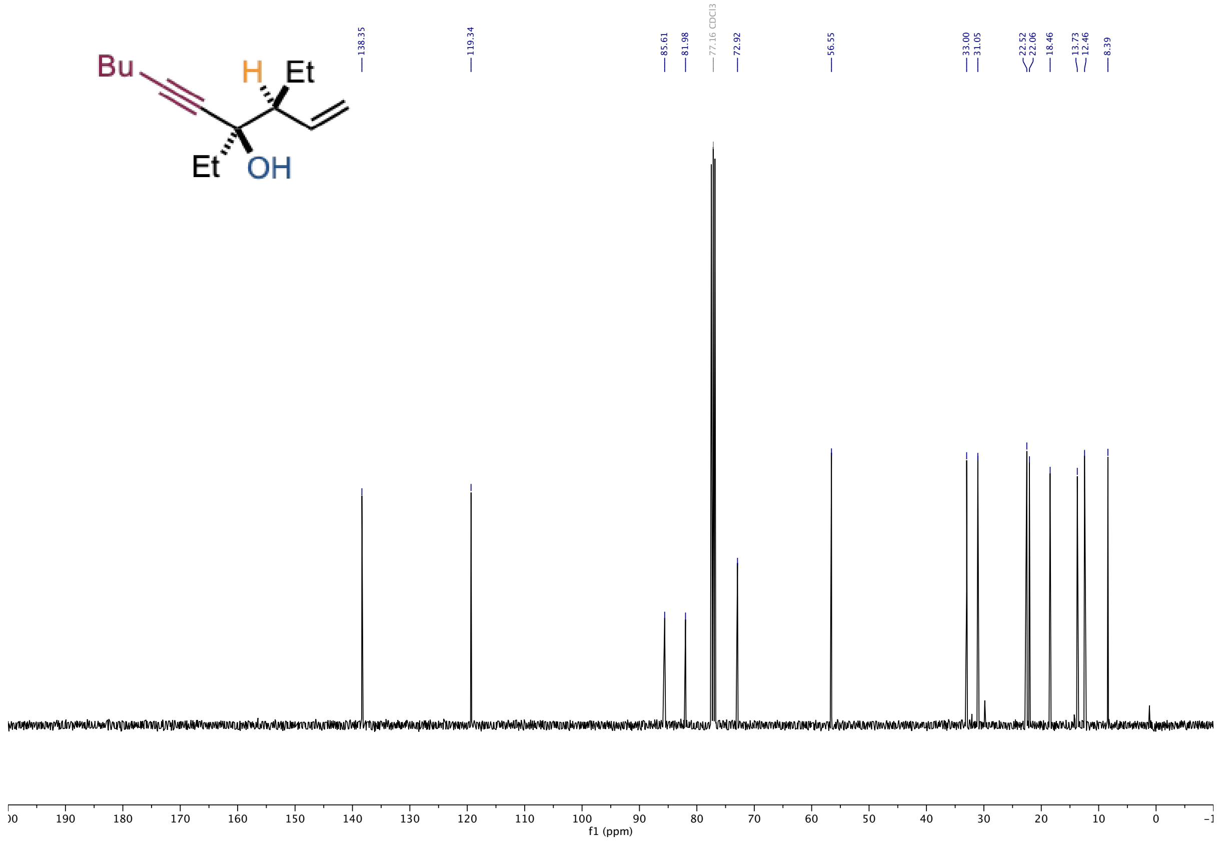

¹³C NMR spectrum (101 MHz, $CDCl_3$)

(4*S**,5*R**)-5-Butyl-4-vinylundec-1-en-6-yn-5-ol (8g)

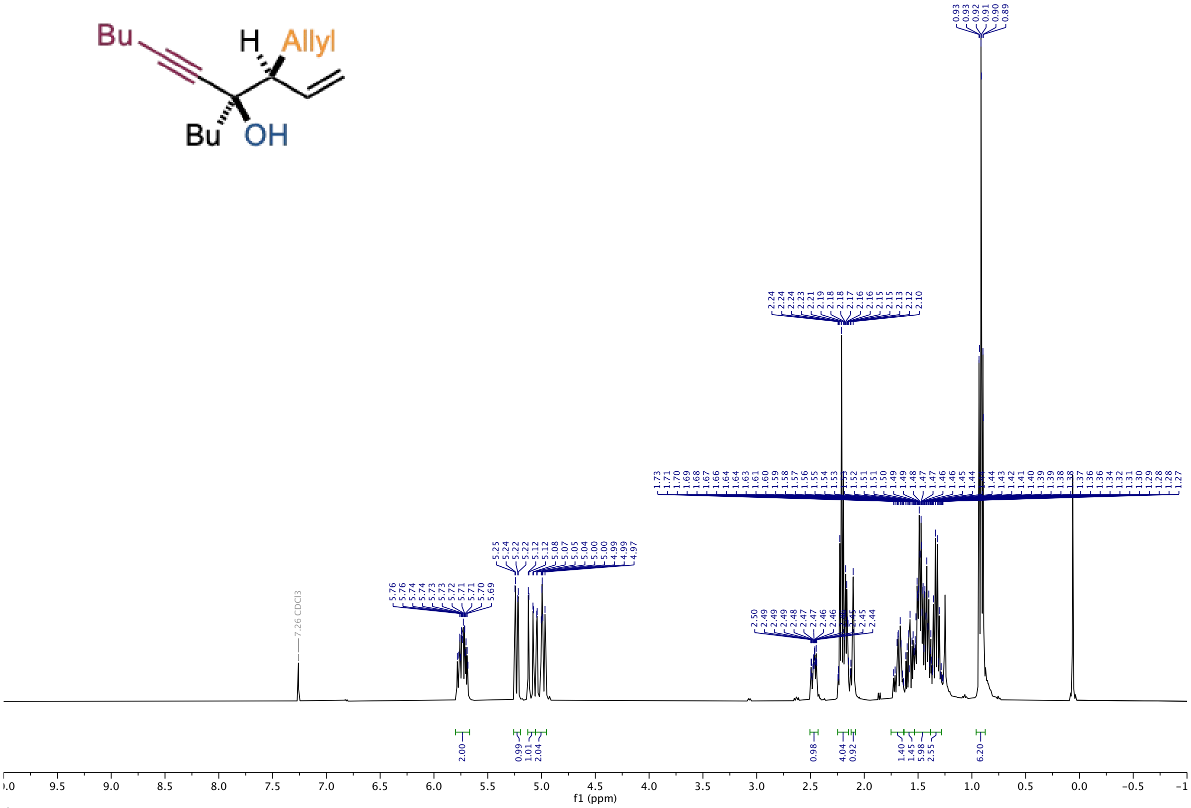

^{1}H NMR spectrum (400 MHz, $CDCl_3$)

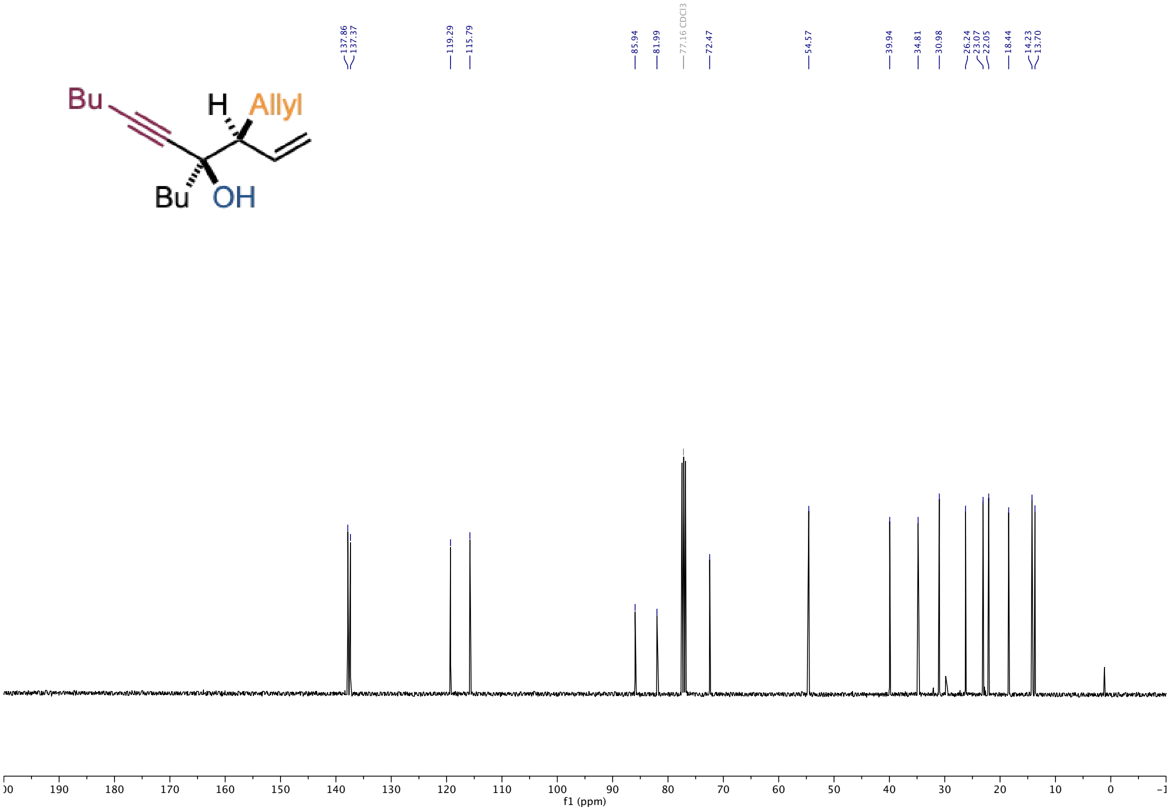

^{13}C NMR spectrum (101 MHz, $CDCl_3$)

(4*S**,5*R**)-5-((Trimethylsilyl)ethynyl)-4-vinylnon-1-en-5-ol (8h)

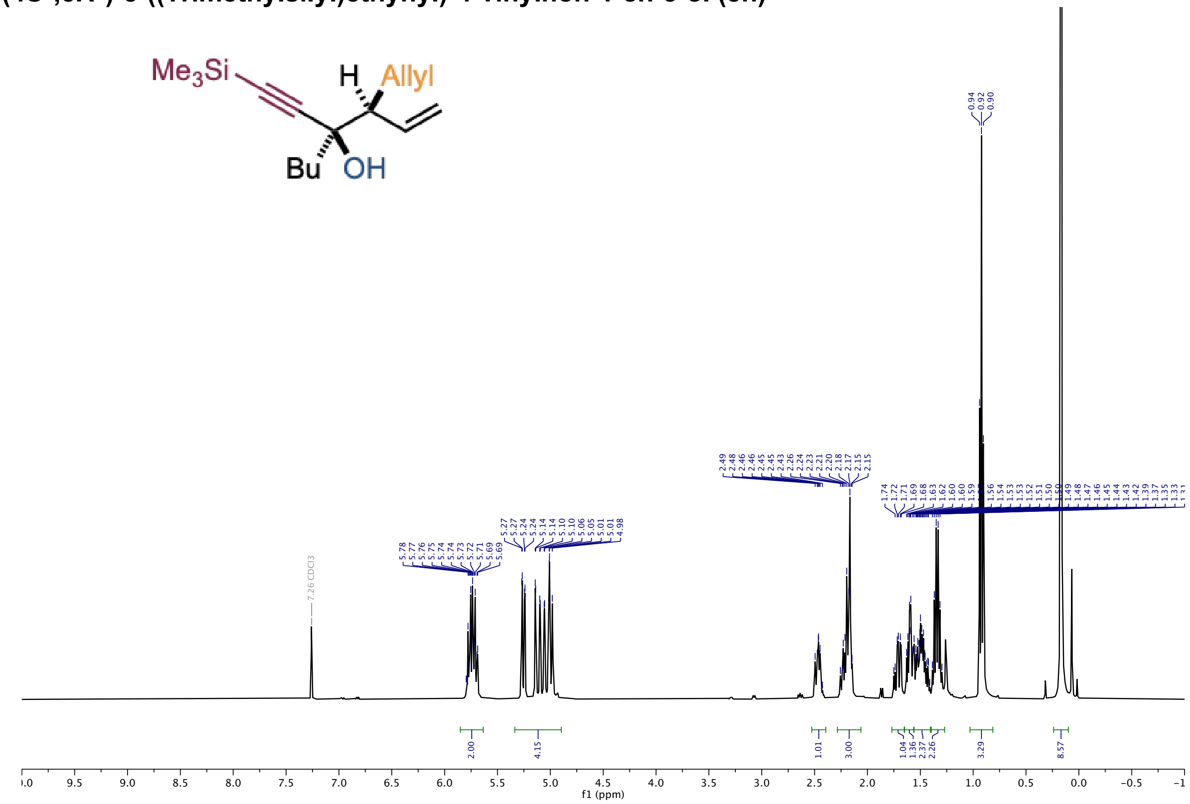

^{1}**H NMR** spectrum (400 MHz, $CDCl_3$)

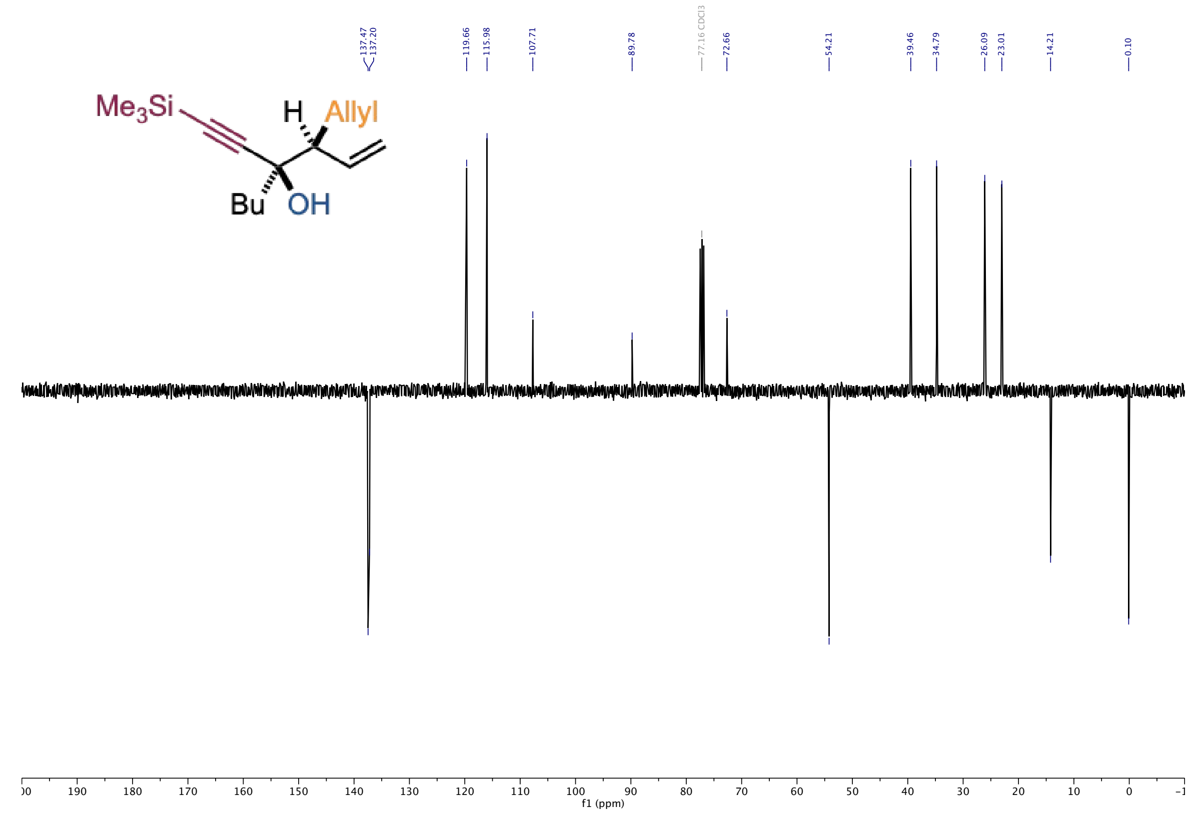

^{13}**C NMR** (APT) spectrum (101 MHz, $CDCl_3$)

(4*S**,5*R**)-5-(Phenylethynyl)-4-vinylnon-1-en-5-ol (8i)

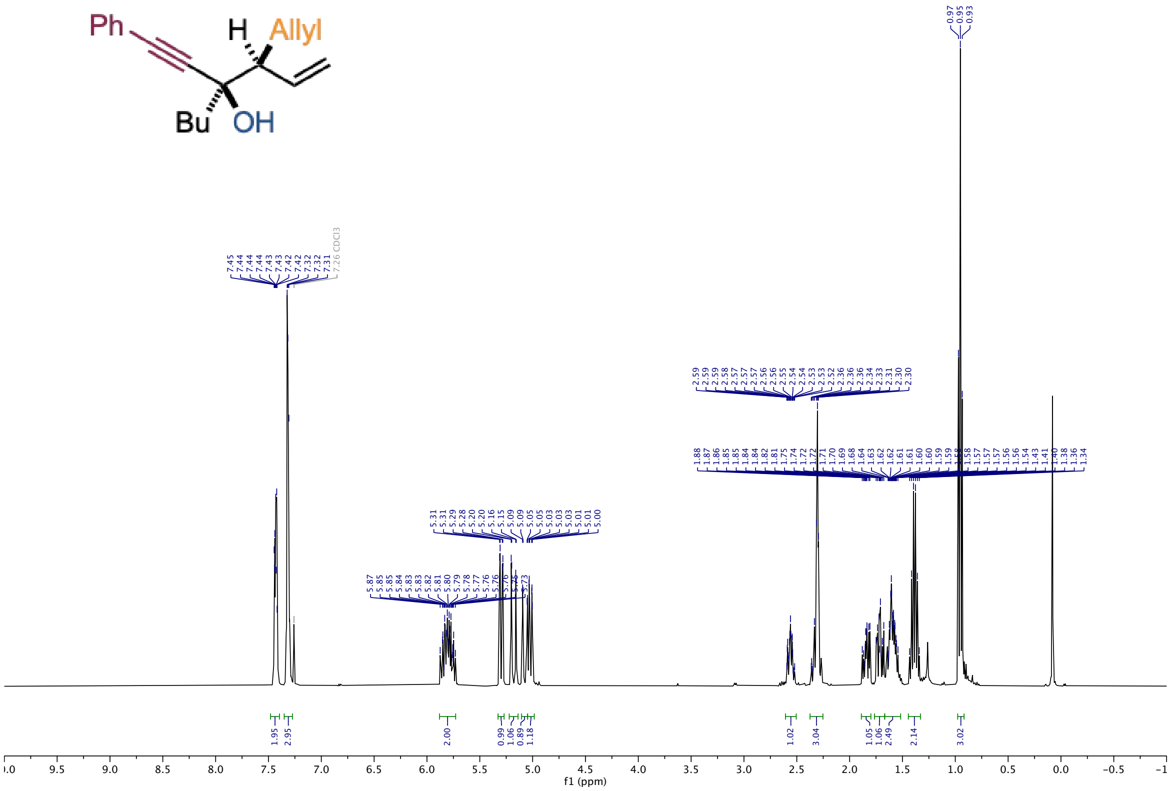

^{1}H NMR spectrum (400 MHz, $CDCl_3$)

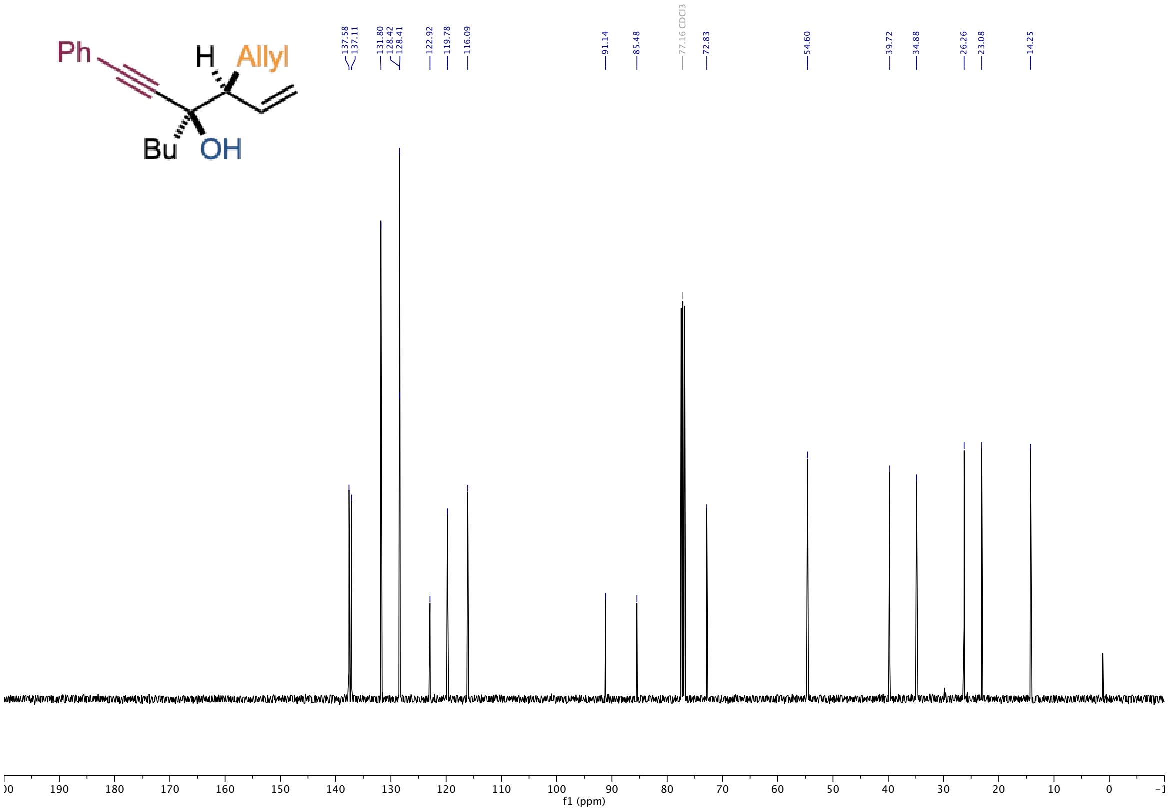

^{13}C NMR spectrum (101 MHz, $CDCl_3$)

(3-butyl-4-vinylhepta-1,2,6-trien-1-yl)benzene (S3)

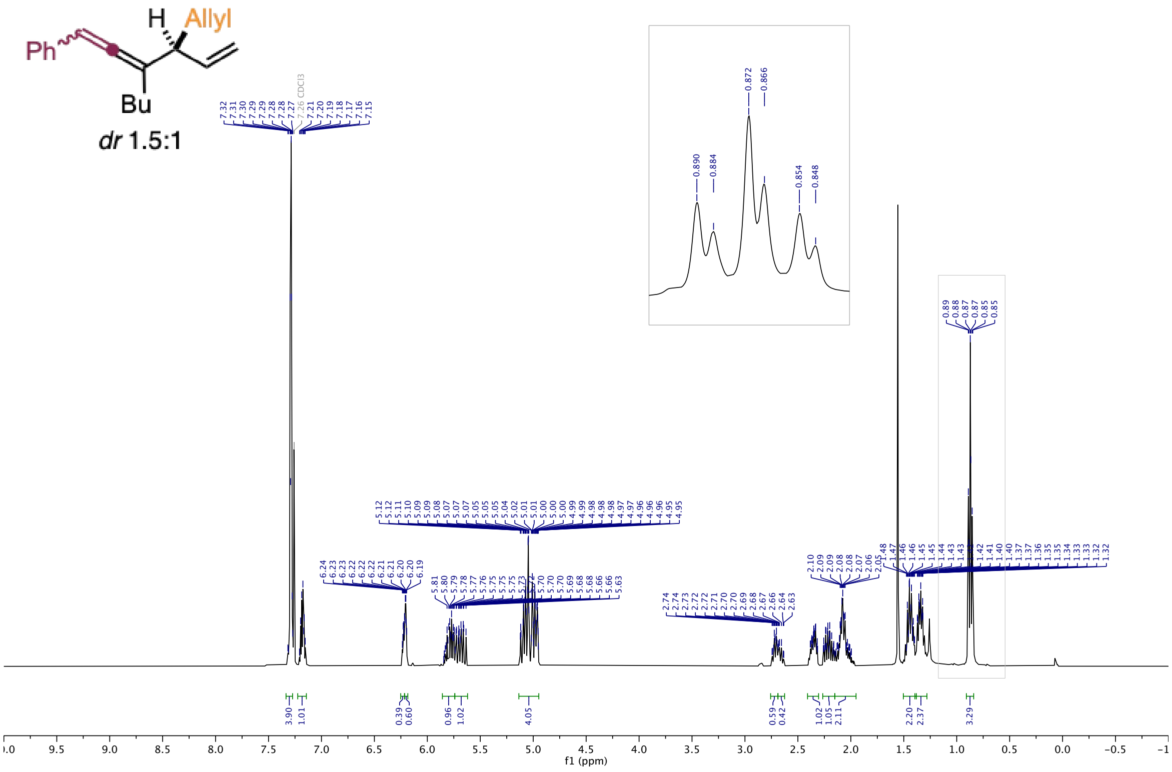

^{1}H NMR spectrum (400 MHz, $CDCl_3$)

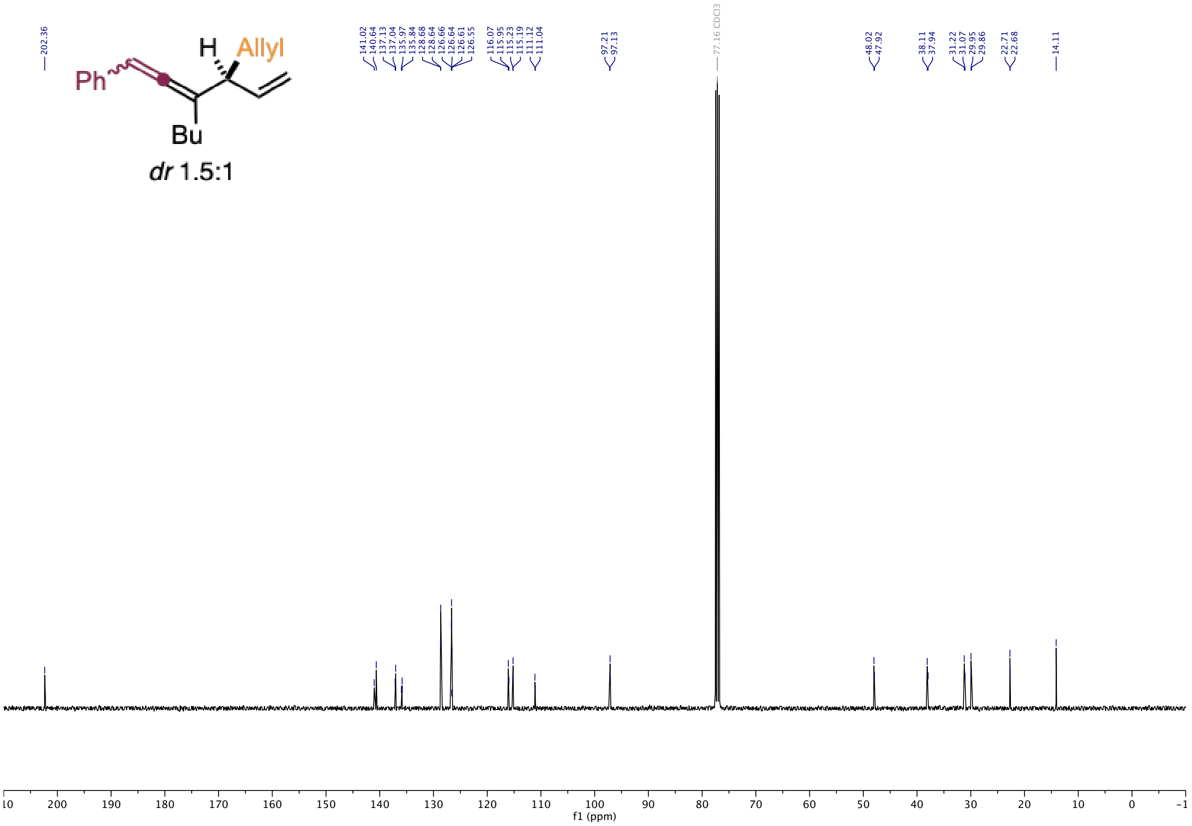

^{13}C NMR spectrum (101 MHz, $CDCl_3$)

(4*S**,5*R**)-5-benzyl-8,8-dimethyl-4-vinylnon-1-en-6-yn-5-ol (8j)

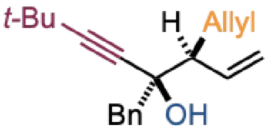

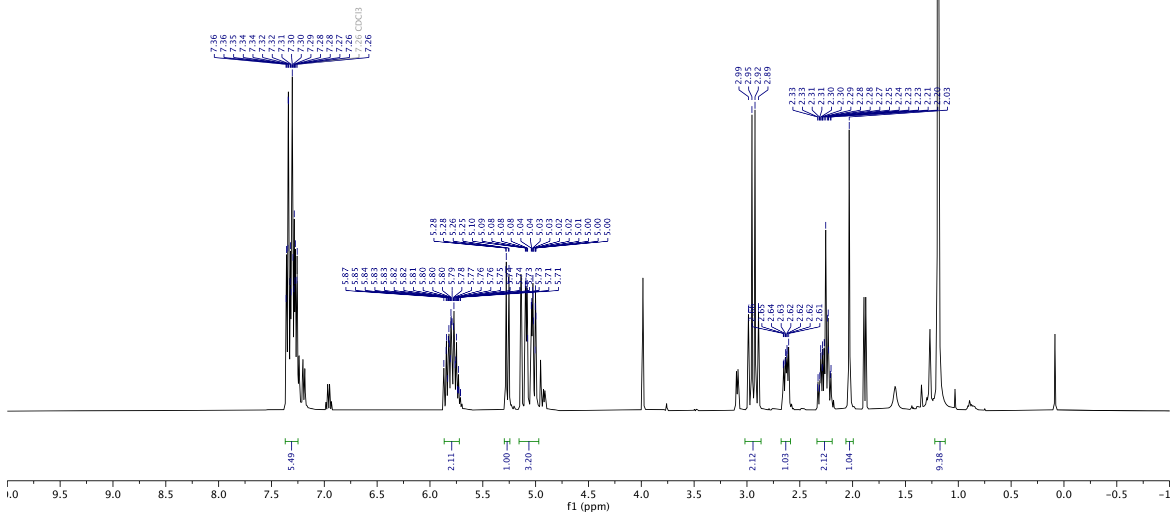

^{1}H NMR spectrum (400 MHz, $CDCl_3$)

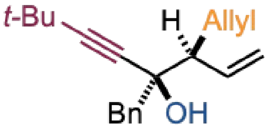

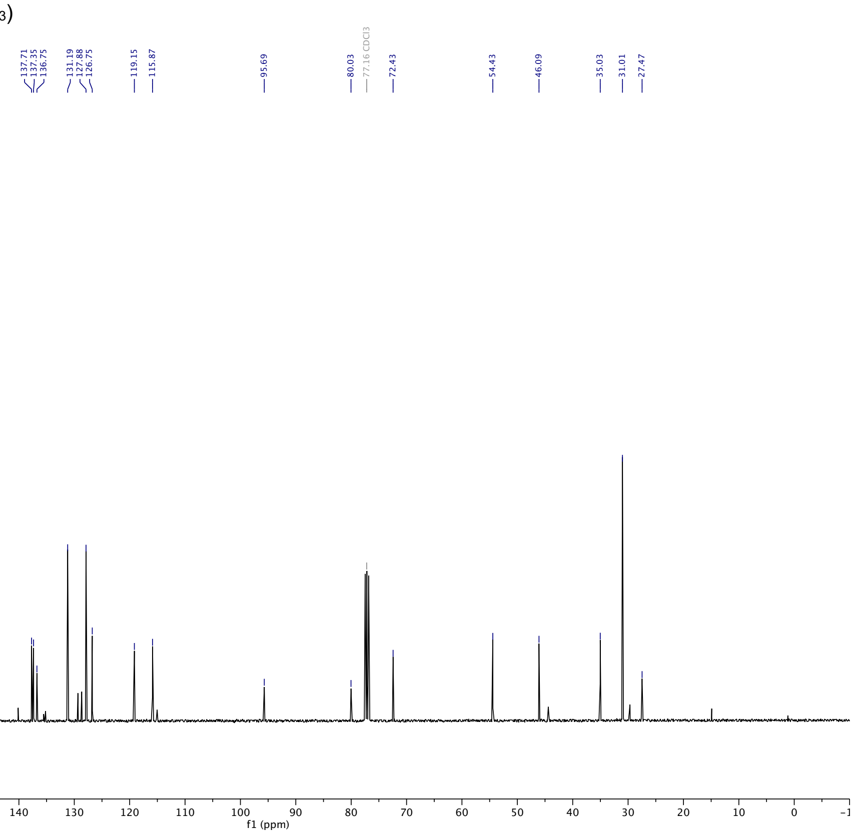

^{13}C NMR spectrum (101 MHz, $CDCl_3$)

(3*R,4*S**)-3-benzyl-1-cyclohexyl-4-vinylhept-6-en-1-yn-3-ol (8k)**

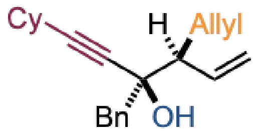

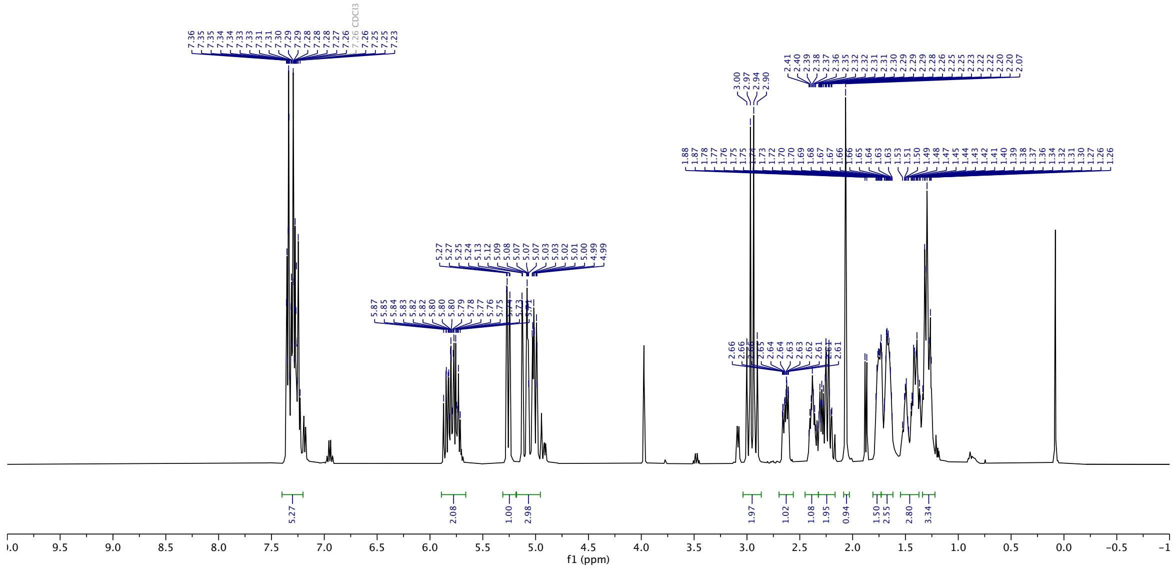

^{1}H NMR spectrum (400 MHz, $CDCl_3$)

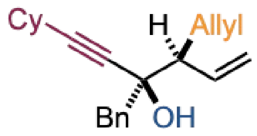

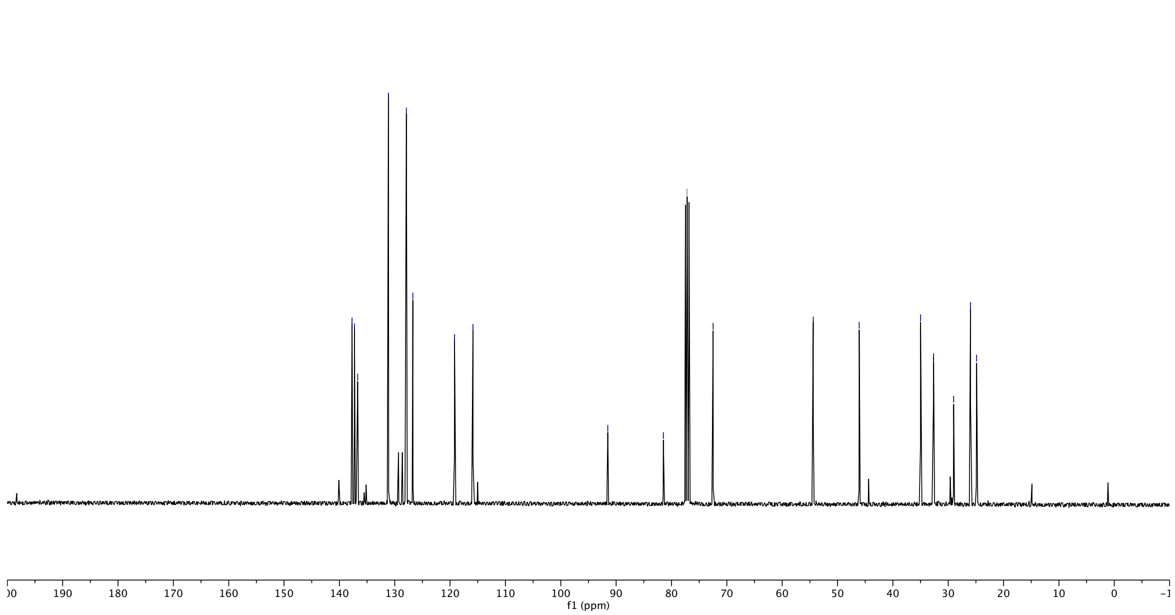

^{13}C NMR spectrum (101 MHz, $CDCl_3$)

((2*S**,4*S**)-3-butyl-4-vinylhepta-1,2,6-trien-1-yl)cyclohexane (9a)

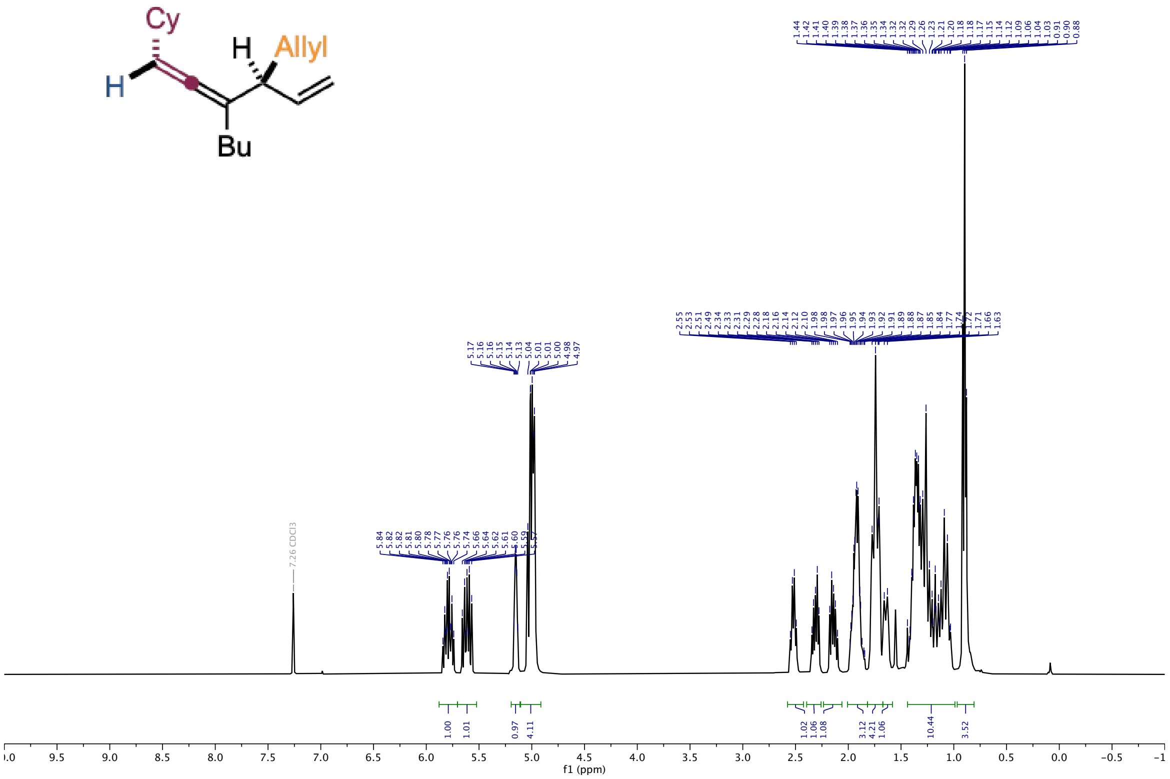

^{1}H NMR spectrum (400 MHz, CDCl$_3$)

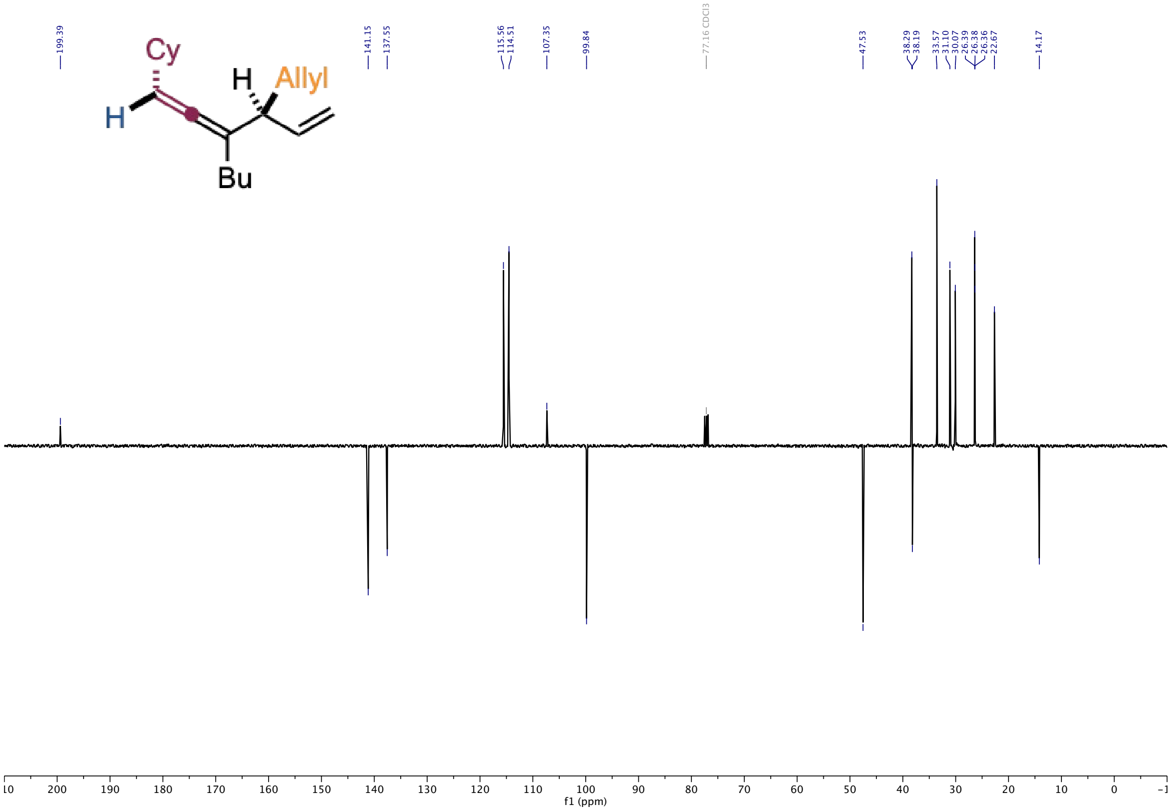

^{13}C NMR (APT) spectrum (101 MHz, CDCl$_3$)

((2*R**,4*S**)-3-Butyl-1-cyclohexyl-4-vinylhepta-1,2,6-trien-1-yl)benzene (9b)

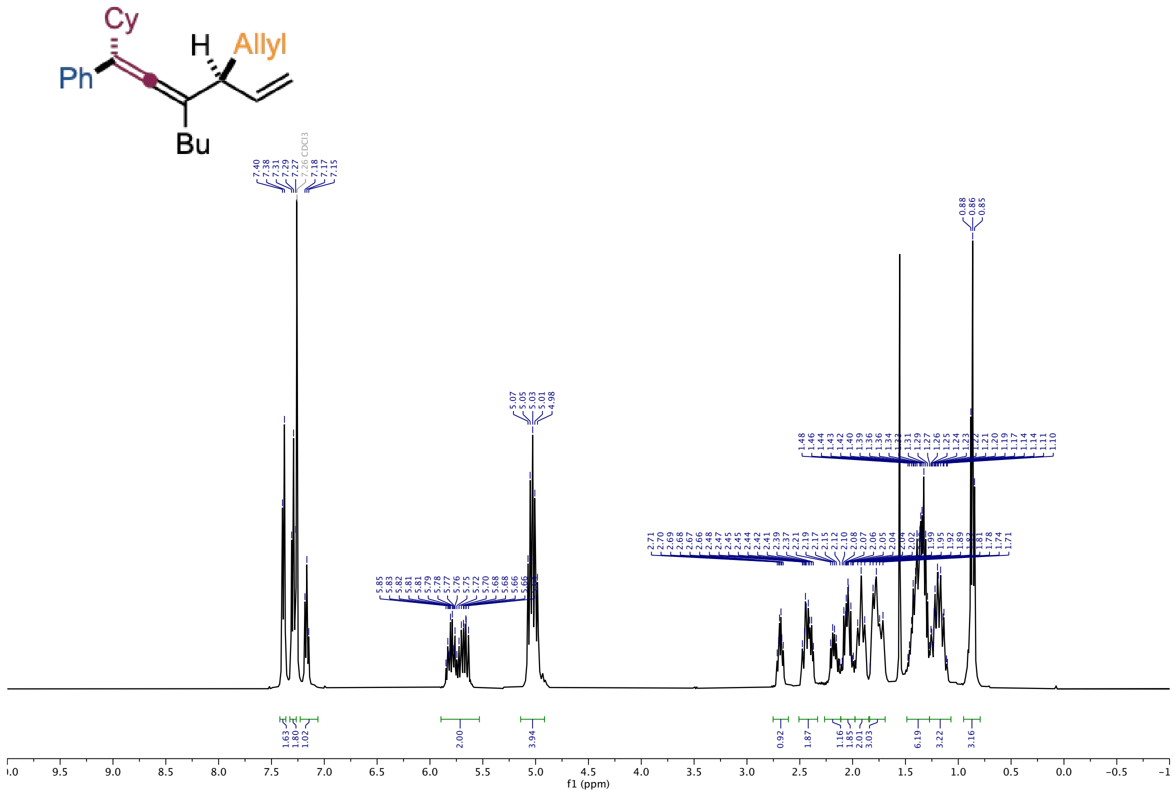

^{1}**H NMR** spectrum (400 MHz, $CDCl_3$)

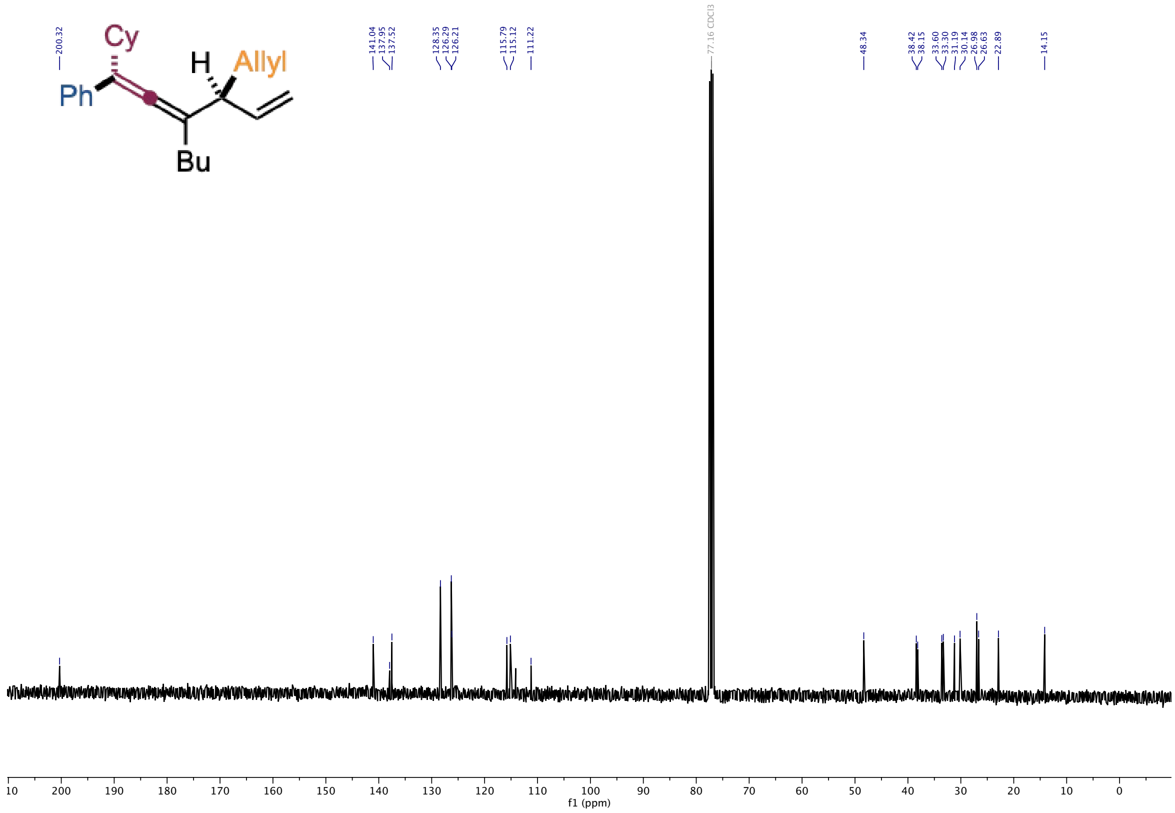

^{13}**C NMR** spectrum (101 MHz, $CDCl_3$)

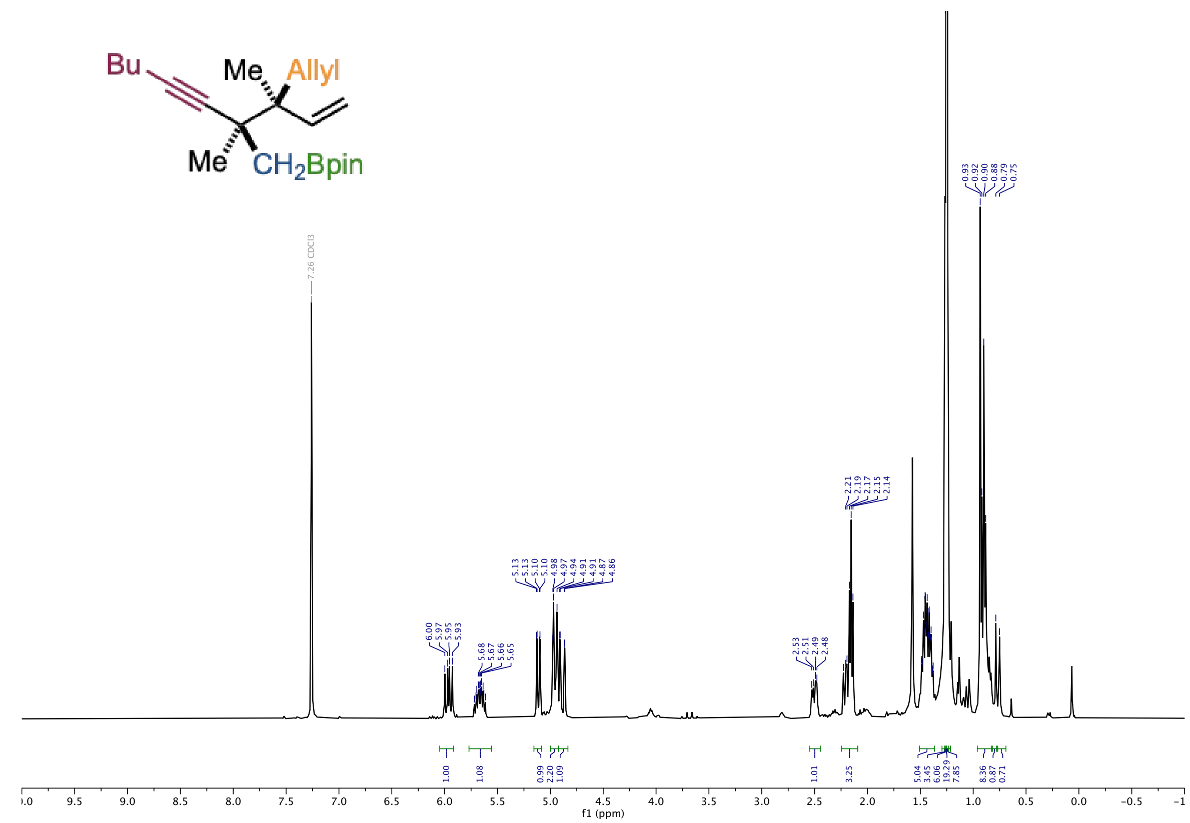

1H NMR spectrum (400 MHz, $CDCl_3$)

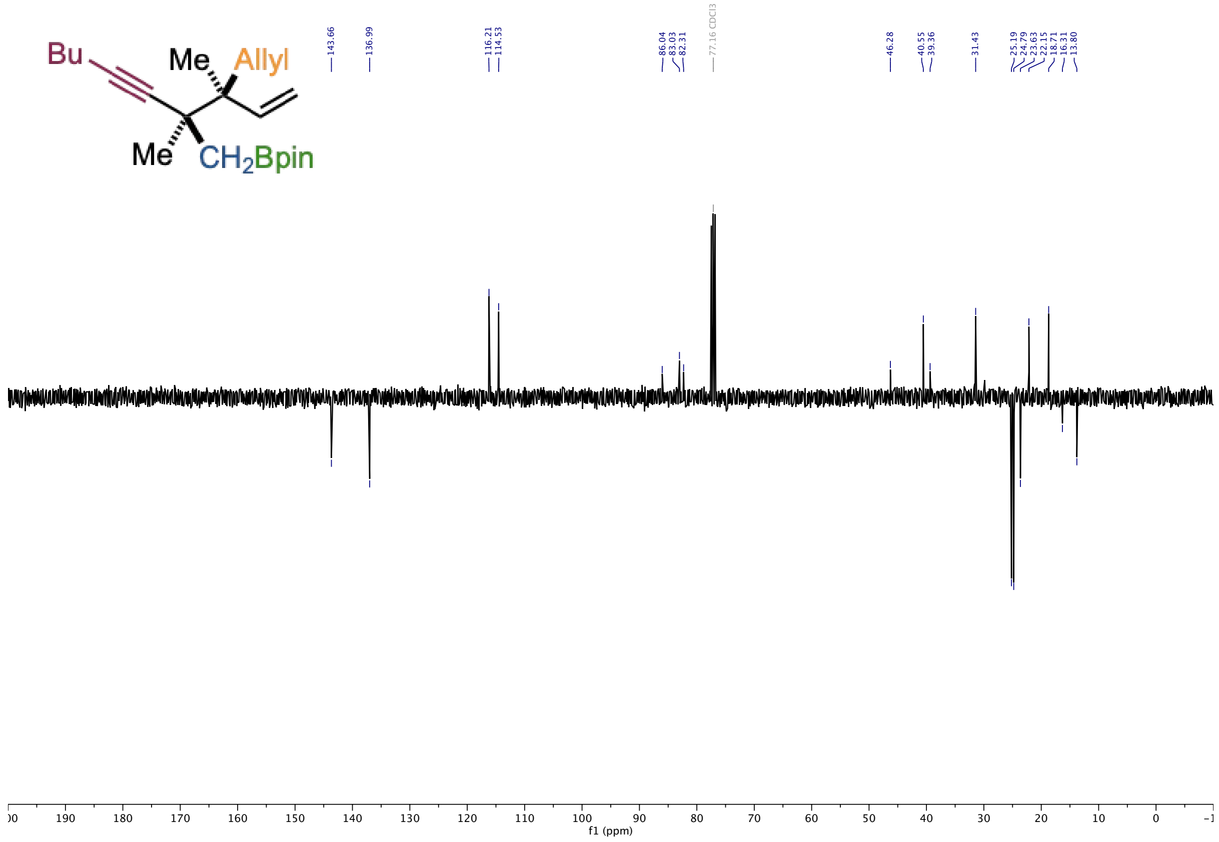

13C NMR (APT) spectrum (101 MHz, $CDCl_3$)

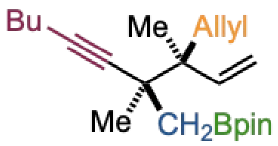

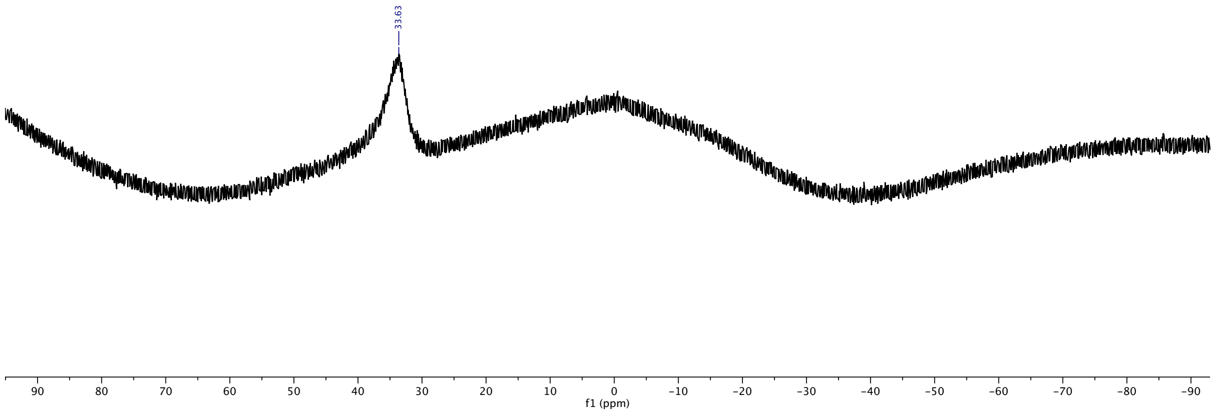

^{11}B NMR spectrum (128 MHz, $CDCl_3$)

5. References

(1) (a) Petiniot, N.; Anciaux, A. J.; Noels, A. F.; Hubert, A. J.; Teyssié, P. Rhodium catalysed cyclopropenation of acetylenes. *Tetrahedron Lett.* **1978**, *19*, 1239-1242. (b) Sommer, H.; Marek, I. Diastereo- and enantioselective copper catalyzed hydroallylation of disubstituted cyclopropenes. *Chem. Sci.* **2018**, *9*, 6503-6508. (c) Zhang, F.-G.; Marek, I. Brook Rearrangement as Trigger for Carbene Generation: Synthesis of Stereodefined and Fully Substituted Cyclobutenes. *J. Am. Chem. Soc.* **2017**, *139*, 8364-8370. (d) Gensler, W. J.; Langone, J. J.; Floyd, M. B. Cyclobutenes by ring expansion of cyclopropenes. *J. Am. Chem. Soc.* **1971**, *93*, 3828-3830. (e) Delaye, P.-O.; Didier, D.; Marek, I. Diastereodivergent Carbometalation/Oxidation/Selective Ring Opening: Formation of All-Carbon Quaternary Stereogenic Centers in Acyclic Systems. *Angew. Chem. Int. Ed.* **2013**, *52*, 5333-5337.

(2) Augustin, A. U.; Di Silvio, S.; Marek, I. Borylated Cyclopropanes as Spring-Loaded Entities: Access to Vicinal Tertiary and Quaternary Carbon Stereocenters in Acyclic Systems. *J. Am. Chem. Soc.* **2022**, *144*, 16298-16302.

(3) Cormier, M.; de la Torre, A.; Marek, I. Total Synthesis of C30 Botryococcene and epi-Botryococcene by a Diastereoselective Ring Opening of Alkenylcyclopropanes. *Angew. Chem. Int. Ed.* **2018**, *57*, 13237-13241.

(4) (a) Matteson, D. S.; Mah, R. W. H. Vinylborinic and α-Bromoalkylborinic Esters1. *J. Org. Chem.* **1963**, *28*, 2171-2174. (b) Matteson, D. S.; Man, H.-W.; Ho, O. C. Asymmetric Synthesis of Stegobinone via Boronic Ester Chemistry. *J. Am. Chem. Soc.* **1996**, *118*, 4560-4566.

(5) Partridge, B. M.; Chausset-Boissarie, L.; Burns, M.; Pulis, A. P.; Aggarwal, V. K. Enantioselective Synthesis and Cross-Coupling of Tertiary Propargylic Boronic Esters Using Lithiation-Borylation of Propargylic Carbamates. *Angew. Chem. Int. Ed.* **2012**, *51*, 11795-11799.

(6) Sadhu, K. M.; Matteson, D. S. (Chloromethyl)lithium: efficient generation and capture by boronic esters and a simple preparation of diisopropyl (chloromethyl)boronate. *Organometallics* **1985**, *4*, 1687-1689.
